# Causal associations of obesity related anthropometric indicators and body compositions with knee and hip arthritis: A large-scale genetic correlation study

**DOI:** 10.3389/fendo.2022.1011896

**Published:** 2022-09-29

**Authors:** Chao Wang, Yong Zhu, Zhi Liu, Haitao Long, Zhe Ruan, Shushan Zhao

**Affiliations:** ^1^ Department of Orthopaedics, Xiangya Hospital, Central South University, Changsha, China; ^2^ National Clinical Research Center for Geriatric Disorders, Xiangya Hospital, Central South University, Changsha, China

**Keywords:** obesity, anthropometric measurement, body compositions, knee arthritis, hip arthritis, genetic correlation, Mendelian randomization, causal association

## Abstract

**Backgrounds:**

Epidemiological studies have repeatedly investigated the association between obesity related anthropometric indicators and body compositions and osteoarthritis (OA). However, the results have remained inconsistent. This work aimed to investigate the genetic correlation and causal associations of obesity related anthropometric indicators and body compositions with knee and hip OA.

**Methods:**

Single-nucleotide polymorphisms associated with the exposures were searched from the recent genome-wide association studies (GWAS) to obtain full statistics. Summary-level results of knee and hip OA were from the UK Biobank and arcOGEN. First, linkage disequilibrium score regression (LD score regression) was applied to detect the genetic correlation (rg). We further performed a series of sensitivity analyses as validation of primary mendelian randomization (MR) results and the specific evidence of potential causal effects was defined.

**Results:**

We found that genetic components in OA had significant correlation with obesity related traits, except waist-to-hip ratio. In the univariable MR analysis, with the exception of waist-to-hip ratio, obesity related anthropometric indicators were causally associated with increased risks of knee and hip OA. For obesity related body compositions, higher fat-free mass in arm, leg, and whole body increased the risk of knee OA but only fat-free mass in leg showed a significant association with hip OA. Meanwhile trunk fat mass and trunk fat percentage, were associated with knee but not with hip OA. Higher fat mass, and fat percentage in arm, leg, and whole body increased the risk of both knee and hip OA. After adjusting for BMI, the multivariable MR showed maintained results in knee OA. However, in hip OA, only fat mass and fat-free mass in arm, leg, trunk and whole body were significantly associated with the risk of hip OA.

**Conclusion:**

The present study suggests genetic evidence for certain causal associations of obesity related anthropometric indicators and body compositions with knee and hip OA, which may provide important insights for the prevention and treatment on OA.

## 1 Introduction

Osteoarthritis (OA) is a common disabling disease. With the comprehensive impacts of the aging and the increasing of obesity in the global population, an estimated 250 million people around the world are affected by OA, which brings a huge and increasing health burden to the society. Clinically, osteoarthritis most often occurs in the knee, followed by the hand and hip (1).

Genome-wide association study (GWAS) is a research method used to identify genomic variants associated with the risk of a disease or a specific trait. Recent GWAS have identified some statistically independent risk variants, which explain a small proportion of the phenotypic variance and are mainly related to osteoarthritis affecting the knee and hip joints (2). The heritability of osteoarthritis is about 50% and a total of 21 loci, spanning hip and knee OA, have been identified in previous genetic studies (3).

Obesity and osteoarthritis are two interrelated health care problems. It is estimated that by 2030, the increasing weight among population will result in nearly 1.3 billion adults being considered overweight and 573 million classified as obese (4, 5). Epidemiological studies showed that obesity was one of the main risk factors of knee OA (6, 7), and more contradictory results were found in the association of hip OA (8–11). At present, a large number of epidemiological studies explored the relationship between body mass index(BMI), a surrogate marker for adiposity, and OA, but there is relatively little evidence on the association between body compositions (fat mass and fat-free mass) and the risk of OA. What’s more, BMI is not an exclusive measure of adiposity though, but rather reflects the composite of fat, muscle and bone mass (6). Meanwhile, observational studies are commonly susceptible to confounding factors, reverse causality, limited follow-up time and small samples, which may lead to erroneous conclusions.

Although Randomized Controlled Trials (RCTs) are gold standard for causality inference, they are often prohibitively expensive or ethically restrictive (12). Recently, many robust causal inference methods have emerged, which could overcome the limitations of observational research. As one of the most popular genetic correlation analysis methods (13), linkage disequilibrium score regression (LDSC) can evaluate the genetic correlation (rg) between obesity and osteoarthritis, though it is hard to determine the causal relationship between them. Mendelian randomization (MR) analysis, which uses genetic variations as instrument variables (IVs), can be used to investigate the causal relationship between exposure and outcome. Since inheritance of genetic variants is random at conception and is accordingly unaffected by environmental factors or disease process, MR is less susceptible to bias from confounding factors and reverse causality (14). In our study, we used the genome-wide significant single nucleotide polymorphisms (SNPs) retrieved from the large-scale genome-wide association studies as IVs, aiming to investigate the genetic correlation and causal associations of obesity related anthropometric indicators and body compositions with knee and hip OA through LD score regression and two-sample MR analysis. Anthropometric indicators of obesity include: weight, BMI, obesity class (I II III), waist circumference (WC), hip circumference (HC) and waist-to-hip ratio (WHR). Body compositions includes: arm fat mass (AFM), arm fat-free mass (AFFM), arm fat percentage (AFP), leg fat mass (LFM), leg fat-free mass (LFFM), leg fat percentage (LFP), trunk fat mass (TFM), trunk fat-free mass (TFFM), trunk fat percentage (TFP), whole body fat mass (WBFM), whole body fat-free mass (WBFFM) and body fat percentage (BFP).

## 2 Materials and methods

### 2.1 Study design

**Figure 1:**
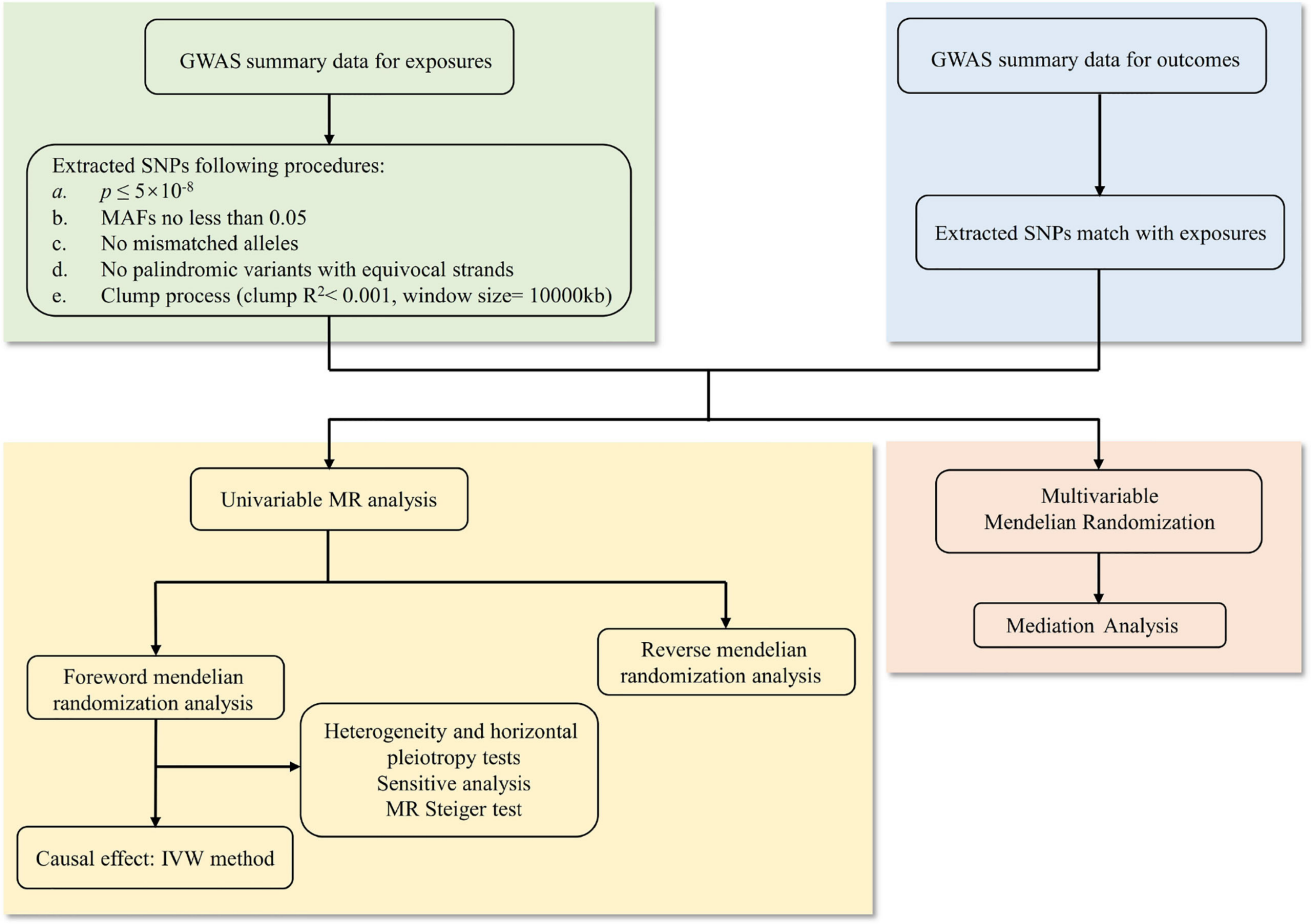
The whole workflow of MR analysis. MR, Mendelian randomization; GWAS, genome-wide association studies; SNPs, single nucleotide polymorphisms; MAFs, minor allele frequencies; IVW, inverse-variance weighted.

In this study, first, we used the cross-trait LDSC method as implemented in LDHub (15) (https://github.com/bulik/ldsc) to estimate the genetic correlation between anthropometric indicators, body compositions and knee (and/or hip) osteoarthritis. Then we used univariable two-sample MR analysis to evaluate the causal relationship between exposures and outcomes. The exposure variables were divided into anthropometric indicators and body compositions of obesity, while knee OA, hip OA and knee or hip OA were classified as outcome. In order to obtain impartial results, three hypotheses of MR must be met: (a) the genetic IVs should be highly correlated with exposure; (b) there is no confounding of the instrument-outcome association; (c) the genetic IVs affected the outcome only through exposure but not through other biological pathways (16). Therefore, the genome-wide significance SNPs (*p ≤*5×10^-8^) were used as instrumental variables, and then a series of sensitivity analyses were carried out, including four MR methods (MR-Egger, weighted median, inverse-variance weighted and weighted mode), as well as heterogeneity and horizontal pleiotropy tests (such as Cochran’s Q test, MR-Egger intercept test and MR-PRESSO test) to verify our results. Bidirectional mendelian randomization analysis and MR Steiger test were performed to estimate the potential reverse causal impact of obesity on knee and hip OA. For the purpose of assessing whether the causal impact of other obesity-related characteristics on the outcomes were mediated by BMI, we adjusted each anthropometric indicator and body composition with BMI for multivariable MR analysis by multivariable inverse-variance weighted method (MV-IVW) (17). Finally, we calculated the proportion of BMI’s effect on knee and hip OA mediated by other obesity-related traits with a mediation analysis (18). The whole workflow of MR analysis were shown in **Figure 1**.

### 2.2 Data sources

All analyses were based on publicly shared databases and no additional ethical approvals were required. Participants in the genome-wide association study were of European descent on all variables.

Anthropometric indicators of obesity were obtained from the summary statistics of the Genetic Investigation of Anthropometric Traits (GIANT) consortium. A meta-analysis of GWAS using data up to 263407 individuals of European ancestry from 51 studies participating in the GIANT consortium, subdivided indicators into obesity class I (n = 98697), class II (n = 72546) and class III (n = 50364) (19). Summary-level GWAS data for waist to hip ratio (n = 224452), waist circumference (n = 134593), and hip circumference (n = 127997) were acquired from a meta-analysis of 224459 individuals of European ancestry from the GIANT consortium (20). Summary-level data for weight (n = 73137) and BMI (n = 51852) were obtained from previous large-scale GWAS, including male and female participants from European population (21).

Genetic associations with the body compositions of obesity were obtained from summary statistics from the MRC-IEU and Neale Lab. Data of arm fat mass (n = 454757), arm fat-free mass (n = 454753), leg fat mass (n = 454846), leg fat-free mass (n = 454835), leg fat percentage (n = 454826) and body fat percentage (n = 454633) obtained from males and females of European ancestry from the MRC-IEU consortium. While statistics of arm fat percentage (n = 331249), trunk fat mass (n = 331093), trunk fat-free mass (n = 331030), trunk fat percentage (n = 331113), whole body fat mass (n = 330762) and whole-body fat-free mass (n = 331291) were acquired from the Neale Lab consortium (http://www.nealelab.is/blog/2017/7/19/rapid-gwas-of-thousands-of-phenotypes-for-337000-samples-in-the-uk-biobank).

The aggregated data on osteoarthritis was from a large meta-analysis of GWAS with 455221 individuals contained both males and females from the UK Biobank and arcOGEN (2). 24955 patients with knee OA, 15704 patients with hip OA, 39427 patients with knee or hip OA and 378169 control participants from the European population were included in the study.

Some of our exposure data among body compositions of obesity were also from the UK Biobank. Sample overlap may be biased in the direction of the observational association between the risk factor and outcome (22). However, a recent study (23) had shown that for one single large dataset, such as the UK Biobank, main two-sample MR methods can be safely used, except MR-Egger. In addition, we calculated the value of I^2^ GX (24) of these overlapped sample (**Supplementary Table 1**). Large I^2^ GX value (more than 90%) showing a strong genetic variant–exposure associations. In the case of very high variability in instrument strength of different variants, the bias from MR-Egger was greatly reduced (24). Meanwhile, we used a recently developed MR method, MRlap, that is robust to bias introduced by sample overlap, winner’s curse and weak instruments. MRlap revealed almost identical results compared to our primary analysis (**Supplementary Table 1**), suggesting sample overlap may not substantially bias estimates (25). Finally, we used MR-PRESSO (Mendelian Randomization Pleiotropy RESidual Sum and Outlier) method to detect outliers and MV-IVW for multivariable MR analysis, since they are variations based on the IVW method, it could be safely used (23, 26).

### 2.3 Genetic instruments selection

We screened the single nucleotide polymorphisms (SNPs) according to following procedures: (1) a genome-wide significance threshold was adopted (*p ≤*5×10^-8^); (2) SNPs with minor allele frequencies (MAFs) less than 0.05 were excluded; (3) SNPs with mismatched alleles (i.e., C/T and C/A) were excluded; (4) palindromic variants with equivocal strands (i.e., C/G or A/T) were excluded; (5) In Plink, lead SNPs with parameters (window size= 10000 kb, r^2^< 0.001) were clumped from the genetic variation selected above.

### 2.4 Statistical analysis

We calculated the F statistic of SNPs to measure the intensity of IVs. IVs with F statistics < 10 were excluded.

The inverse-variance weighted method (IVW) was used as the main analysis, which is considered as a method to provide the most accurate estimation when all IVs are effective. MR-Egger regression, weighted median and weighted model were used for sensitivity analysis. Cochran’s Q-test was used to test the heterogeneity of IVW estimates. The pleiotropy was detected by MR-Egger intercept test (*p* for intercept < 0.05). We also performed MR-PRESSO method, including global, outlier and distortion tests, to detect and correct pleiotropy and potential outliers (27). A recently developed MR method was performed to correct the bias introduced by sample overlap (25). Meanwhile, the direction of causality is inferred based on the Steiger test (28).

We defined the evidence of potential causal effects when the following criteria were met: after Bonferroni correction, the MR results of IVW passed the multiple comparisons adjusted *p* value <0.0025 (0.05/20); The remaining MR methods showed similar size and direction to IVW (*p <*0.05 in weighted medium and weighted mode, as well as MR-Egger showed a similar effect size); There was no evidence of heterogeneity and level pleiotropy after exclusion of potential outliers. Funnel plots and scatter plots of MR analysis were used to visually assess level pleiotropy and heterogeneity (**Supplementary Figure 1**-**120**). MR and other analysis were performed in R (version 4.0.3) using R software packages “two-sample MR”, “Mendelian Randomization”, “MRlap” and “MR-PRESSO”. *p* values were bilateral, and *p* value < 0.05 is considered to be suggestively significant for the remaining methods (except IVW).

## 3 Results

### 3.1 Genetic correlation and genetic instruments

**Figure 2:**
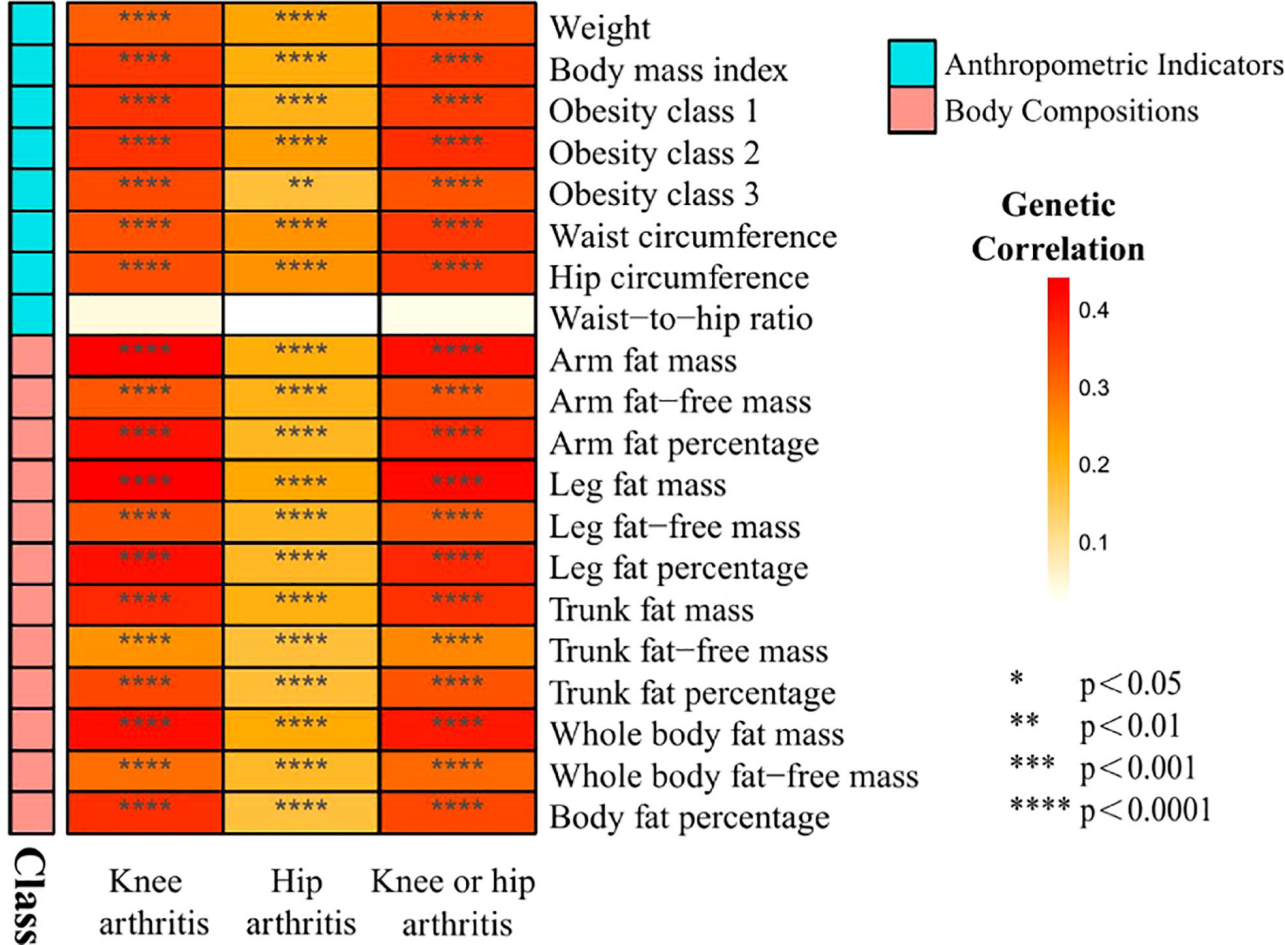
Heat map of genetic correlations between knee and hip osteoarthritis and 20 obesity traits grouped in 2 categories from GWAS consortia.

The characteristics of the SNPs for the exposures and outcomes are showed in **Tables 1**, **2**. First, the GWAS summary data of obesity related parameters and OA were used for genetic correlation analysis, and we found that osteoarthritis genetic components had significant correlation with obesity related traits (rg >0, *p ≤*0.05), except WHR (**Figure 2**; **Table 3**). Then, as genetic instruments we selected related SNPs. Summary statistics of instrumental SNPs as genetic instrumental variables were presented in **Table 1** and **Supplementary Table 2**. The average values of F statistic are shown in **Table 1**. F statistic < 10 was considered as weak IV and was excluded.

**Table 1:** Overview of genome-wide association studies used in the analyses for the exposures and characteristics of the Single Nucleotide Polymorphisms (SNPs) considered as instrumental variables.

Characteristics	Sample size	Consortium		Number of variants (before selection)	Number of variants (after selection)	F-statistic, mean
Waist circumference (WC)	134593	GIANT	19		18	60.671
Arm fat mass (AFM)	454757	MRC-IEU	428		400	63.072
Arm fat-free mass (AFFM)	454753	MRC-IEU	517		484	77.532
Arm fat percentage (AFP)	331249	Neale Lab	242		228	54.694
Obesity class 3	50364	GIANT	2		2	71.491
Obesity class 2	72546	GIANT	11		11	61.463
Obesity class 1	98697	GIANT	17		17	65.308
Body mass index (BMI)	51852	Within family GWAS consortium	11		7	55.846
Whole body fat mass (WBFM)	330762	Neale Lab	290		270	56.231
Whole body fat-free mass (WBFFM)	331291	Neale Lab	418		389	72.088
Body fat percentage (BFP)	454633	MRC-IEU	395		366	58.776
Leg fat mass (LFM)	454846	MRC-IEU	427		387	61.523
Leg fat-free mass (LFFM)	454835	MRC-IEU	513		475	78.773
Leg fat percentage (LFP)	454826	MRC-IEU	383		353	55.384
Hip circumference (HC)	127997	GIANT	18		17	59.190
Weight	73137	GIANT	11		11	51.212
Trunk fat mass (TFM)	331093	Neale Lab	290		277	55.503
Trunk fat-free mass (TFFM)	331030	Neale Lab	418		387	74.750
Trunk fat percentage (TFP)	331113	Neale Lab	244		228	52.597
Waist-to-hip ratio (WHR)	224452	GIANT	37		36	55.251

**Table 2:** Overview of genome-wide association studies used in the analyses for the outcomes.

Characteristics	Knee osteoarthritis	Hip osteoarthritis	Osteoarthritis of the hip or knee
Consortium	UK Biobank and arcOGEN	UK Biobank and arcOGEN	UK Biobank and arcOGEN
PMID	30664745	30664745	30664745
Year	2019	2019	2019
Population	European	European	European
Cases	24955	15704	39427
Control	378169	378169	378169
Sample size	403124	393873	417596

**Table 3:** Genetic correlation between obesity related anthropometric indicators and body compositions and knee and hip arthritis.

Obesity related anthropometric indicators and body compositions	Osteoarthritis	Genetic correlation (rg)	p-value^*^
Weight	Knee arthritis	0.3149	9.54E-23
	Hip arthritis	0.2293	3.1E-12
	Knee or hip arthritis	0.3319	1.86E-29
Body mass index	Knee arthritis	0.3619	1.11E-20
	Hip arthritis	0.2122	1.08E-06
	Knee or hip arthritis	0.3569	1.96E-22
Obesity class 1	Knee arthritis	0.3667	1.23E-35
	Hip arthritis	0.2028	2.2E-08
	Knee or hip arthritis	0.356	1.03E-33
Obesity class 2	Knee arthritis	0.3694	2.5E-23
	Hip arthritis	0.2393	2.19E-09
	Knee or hip arthritis	0.3746	2.46E-26
Obesity class 3	Knee arthritis	0.3405	1.1E-10
	Hip arthritis	0.175	0.003
	Knee or hip arthritis	0.3295	2.88E-10
Waist circumference	Knee arthritis	0.3331	8.55E-30
	Hip arthritis	0.2545	5.28E-14
	Knee or hip arthritis	0.3618	2.68E-35
Hip circumference	Knee arthritis	0.335	8.89E-30
	Hip arthritis	0.2528	9.49E-14
	Knee or hip arthritis	0.3621	3.39E-35
Waist-to-hip ratio	Knee arthritis	0.047	0.2137
	Hip arthritis	0.0122	0.7526
	Knee or hip arthritis	0.0393	0.2807
Arm fat mass	Knee arthritis	0.4388	2.6E-102
	Hip arthritis	0.2133	5.13E-22
	Knee or hip arthritis	0.4112	1.9E-105
Arm fat-free mass	Knee arthritis	0.3271	2.24E-32
	Hip arthritis	0.2049	2.79E-19
	Knee or hip arthritis	0.3281	1.42E-40
Arm fat percentage	Knee arthritis	0.4104	2.64E-78
	Hip arthritis	0.194	3.89E-15
	Knee or hip arthritis	0.3831	3.18E-79
Leg fat mass	Knee arthritis	0.4432	2.1E-106
	Hip arthritis	0.2233	3.05E-23
	Knee or hip arthritis	0.4185	3.3E-109
Leg fat-free mass	Knee arthritis	0.3293	3E-34
	Hip arthritis	0.1955	3.11E-17
	Knee or hip arthritis	0.3243	3.5E-40
Leg fat percentage	Knee arthritis	0.4112	5.1E-88
	Hip arthritis	0.1922	3.65E-16
	Knee or hip arthritis	0.3812	1.76E-87
Trunk fat mass	Knee arthritis	0.3827	4.57E-68
	Hip arthritis	0.2092	3.45E-21
	Knee or hip arthritis	0.3717	2.64E-78
Trunk fat-free mass	Knee arthritis	0.2536	2.37E-16
	Hip arthritis	0.1744	9.85E-12
	Knee or hip arthritis	0.2657	2.63E-21
Trunk fat percentage	Knee arthritis	0.3464	6.73E-62
	Hip arthritis	0.1764	1.58E-15
	Knee or hip arthritis	0.3295	5.72E-67
Whole body fat mass	Knee arthritis	0.4144	1.08E-80
	Hip arthritis	0.2212	8.73E-23
	Knee or hip arthritis	0.3994	2.13E-92
Whole body fat-free mass	Knee arthritis	0.296	2.18E-22
	Hip arthritis	0.1896	8.85E-14
	Knee or hip arthritis	0.3017	3.55E-28
Body fat percentage	Knee arthritis	0.3728	1.55E-79
	Hip arthritis	0.1728	2.71E-14
	Knee or hip arthritis	0.3445	1.85E-75

*Linkage disequilibrium score regression.

### 3.2 Univariable mendelian randomization

#### 3.2.1 Causal effects of anthropometric indicators and body compositions of adiposity on knee OA

**Figure 3:**
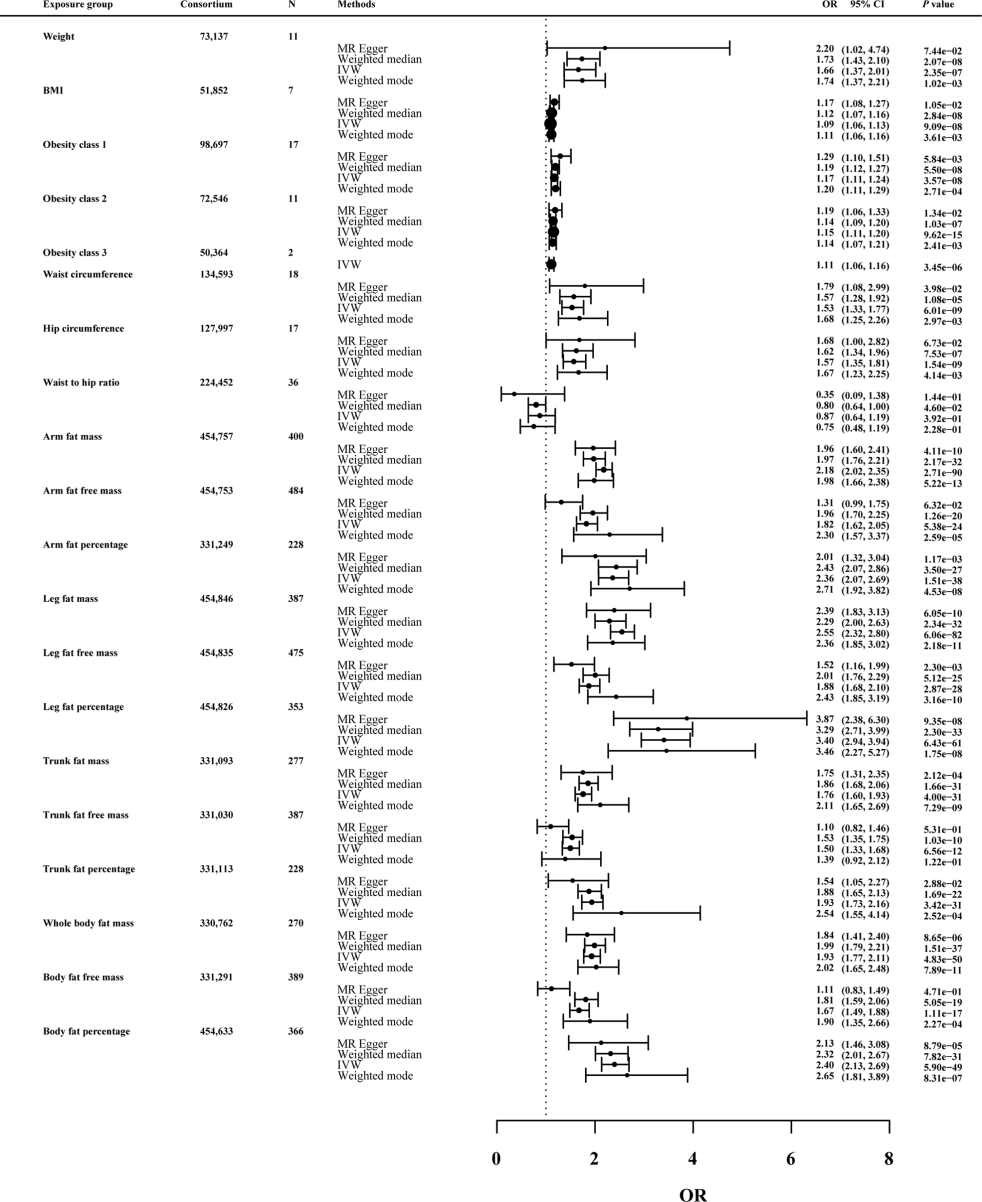
Effects of obesity-related exposures on knee OA. Results from univariable MR analyses showing the effects of genetic predicted obesity related anthropometric indicators and body compositions on knee OA with IVW, MR-Egger, weighted median, and weighted mode methods.

As shown in **Figure 3**, in the univariable MR of knee OA, the genetically predicted BMI was significantly correlated with knee OA. For the 1-SD increase in BMI level, the odds ratios were 1.09 (*p* = 9.09 × 10^-8^) for knee OA with the IVW method. The remaining MR methods showed consistent size and direction to IVW. Among other anthropometric indicators of obesity, genetic prediction of weight (OR: 1.66; *p* value: 2.35×10^-7^), obesity class I (OR: 1.17; *p* value: 3.57×10^-8^), obesity class II (OR: 1.15; *p* value: 9.62×10^-15^) and obesity class III (OR: 1.11; *p* value: 3.45×10^-6^), WC (OR: 1.53; *p* value: 6.01×10^-9^) and HC (OR: 1.57; *p* value: 1.54×10^-9^) were associated with the risk of knee OA, except WHR (OR: 0.87; *p* value: 0.39).

MRlap were performed since the sample overlap. **Supplementary Table 1** showed the preliminary analysis results of genetic prediction levels of body compositions of adiposity (increased 1-SD) and knee OA risk. In the MRlap analysis, genetically raised AFM (OR: 2.17; *p* value: 2.81×10^-65^), AFFM (OR: 1.82; *p* value: 6.91×10^-16^), AFP (OR: 2.33; *p* value: 3.77×10^-26^), LFM (OR: 2.54; *p* value: 3.03×10^-54^), LFFM (OR: 1.87; *p* value: 8.41×10^-18^), LFP (OR: 3.38; *p* value: 2.52×10^-49^), TFM (OR: 1.75; *p* value: 1.02×10^-19^), TFP (OR: 1.92; *p* value: 2.78×10^-21^), WBFM (OR: 1.92; *p* value: 1.37×10^-34^), WBFFM (OR: 1.67; *p* value: 9.08×10^-13^) and BFP (OR: 2.39; *p* value: 1.09×10^-37^) were associated with increased knee OA risk.

#### 3.2.2 Causal effects of anthropometric indicators and body compositions of adiposity on hip OA

**Figure 4:**
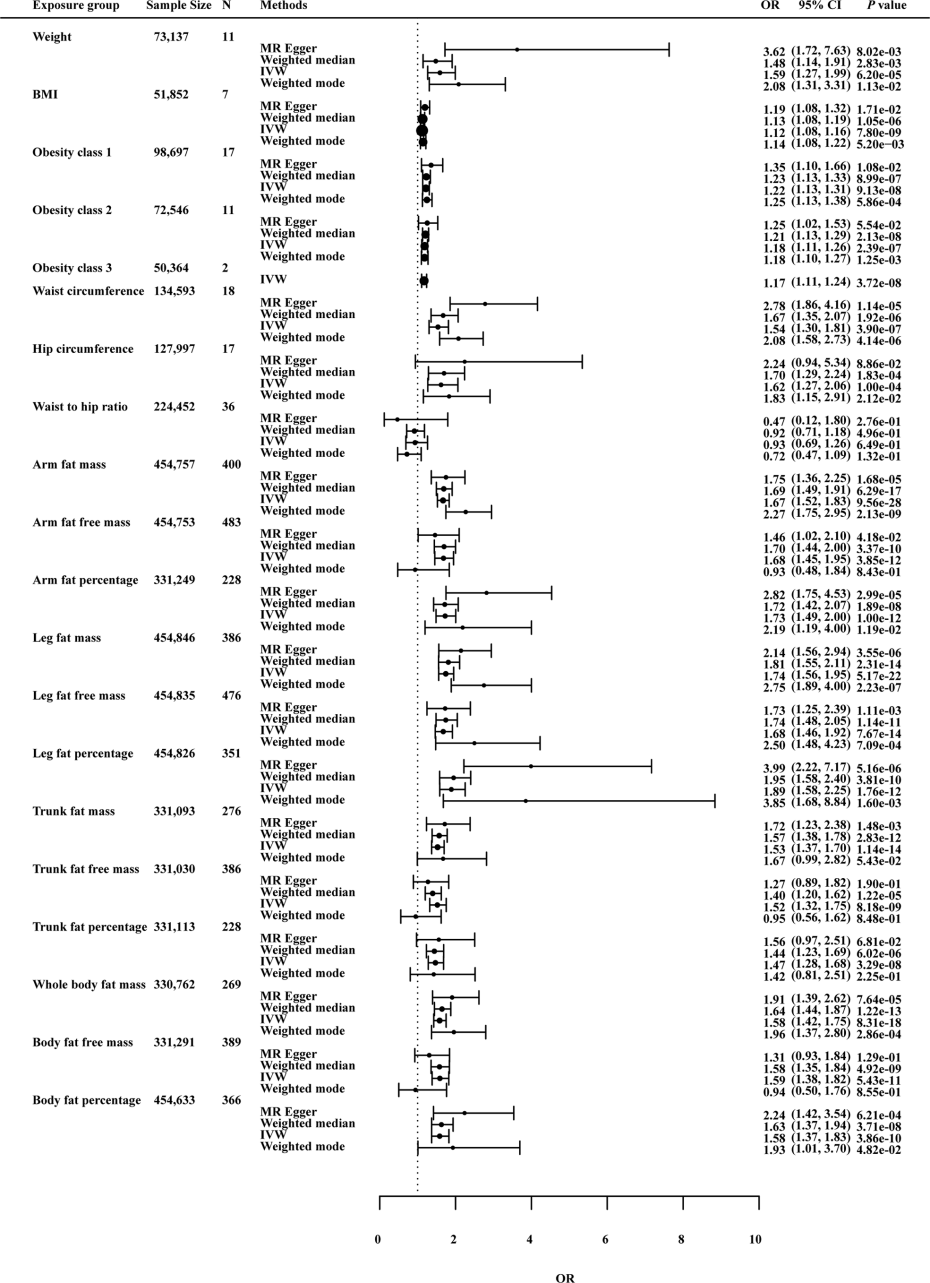
Effects of obesity-related exposures on hip OA. Results from univariable MR analyses showing the effects of genetic predicted obesity related anthropometric indicators and body compositions on hip OA with IVW, MR-Egger, weighted median, and weighted mode methods.

In **Figure 4**, the genetically predicted BMI was predicted to increase risk of hip OA (OR: 1.12; *p* value: 7.79×10^-9^). Consistent results were also observed for other obesity-related measures, such as waist circumference (OR: 1.54; *p* value: 3.90×10^-7^) and hip circumference (OR: 1.62; *p* value: 1.00×10^-4^).

For body compositions of adiposity, AFM (OR: 1.66; *p* value: 8.39×10^-24^), AFP (OR: 1.71; *p* value: 5.51×10^-11^), LFM (OR: 1.74; *p* value: 3.62×10^-19^), LFFM (OR: 1.68; *p* value: 3.39×10^-11^), LFP (OR: 1.88; *p* value: 5.43×10^-10^), WBFM (OR: 1.57; *p* value: 1.48×10^-14^), BFP (OR: 1.58; *p* value: 7.98×10^-8^) all showed significant associations with risk of hip OA in MRlap analysis. Higher levels of these factors were associated with increased risk of hip OA.

#### 3.2.3 Causal effects of anthropometric indicators and body compositions of adiposity on knee or hip OA

**Figure 5:**
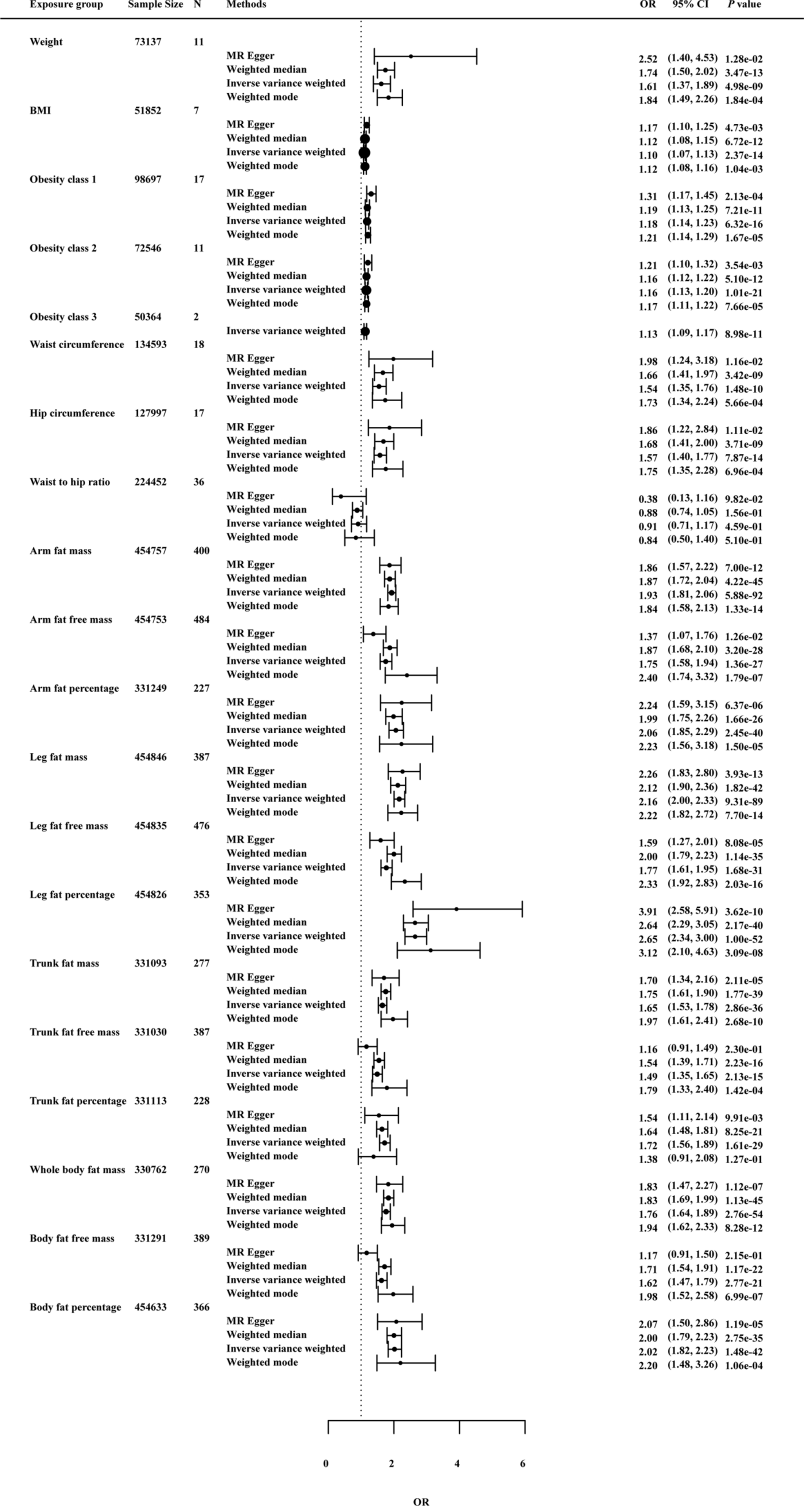
Effects of obesity-related exposures on knee or hip OA. Results from univariable MR analyses showing the effects of genetic predicted obesity related anthropometric indicators and body compositions on knee or hip OA with IVW, MR-Egger, weighted median, and weighted mode methods.

In **Figure 5**, the IVW method determined that BMI is associated with knee or hip OA risk (OR 1.10; *p* = 2.37 × 10^-14^), suggesting that each 1-SD increment in body mass index was predicted to increase risk of knee or hip OA by 1.10. Except for TFP and WHR, the other exposures showed significant correlation with knee or hip OA risk.

#### 3.2.4 Sensitivity analysis

As a further test of the robustness of our findings across MR methodologies, we performed sensitivity analysis and the results were shown in **Figures 3**–**5** and **Supplementary Table 2**. MRlap revealed almost identical results compared to our primary analysis (**Supplementary Table 1**). The results from MR sensitivity analysis methods were consistent with those of IVW (*p* < 0.05 in weighted median and weighted mode, and MR-Egger showed a similar effect size). Although the MR-Egger method in hip circumference in knee OA (OR: 1.68; *p* value: 6.72×10^-2^), weight in knee OA (OR: 2.20; *p* value: 7.44×10^-2^), hip circumference in hip OA (OR: 2.24; *p* value: 8.86×10^-2^) and obesity class 2 in hip OA (OR: 1.25; *p* value: 5.54×10^-2^) was not statistically significant, this method is mainly used to detect possible pleiotropic effects in our study.

On knee OA, possible heterogeneity and pleiotropic effects were detected (**Supplementary Table 3**). Cochran’s Q-test showed that the MR results of AFM, AFFM, AFP, LFM, LFFM, LFP, WBFM, WBFFM, BFP, TFP, TFM, TFFM, weight and WHR were heterogeneous (*p <*0.05). Except AFFM, WBFFM and TFFM, there was no horizontal pleiotropy as assessed by the MR-Egger intercept test (*p* intercept > 0.05). Outliers were detected by the MR-PRESSO method, but no significant differences between the casual estimates before and after correction for outliers were found, and *p* values were >0.05 for distortion tests. On hip OA, these association were maintained after Bonferroni correction for multiple testing (*p* < 0.05/20 = 2.5 × 10^−3^). MR-PRESSO identified outliers and provided revised estimates. After removing the abnormal SNPs, the recalculated MR estimates were similar to the results above (**Supplementary Table 3**). On knee or hip OA, results remained consistent after MR-PRESSO correction for outliers (**Supplementary Table 3**). Funnel plots and scatter plots of MR analysis were used to visually assess level pleiotropy and heterogeneity (**Supplementary Figures 1**-**120**).

#### 3.2.5 Bidirectional mendelian randomization

In the other direction, with genetic liability for knee and hip OA as exposures, we performed MR to explore the causal effect of knee and hip OA on obesity-related traits. After the multiple comparisons adjusted *p* value (0.05/20), across IVW method, we found no evidence of causal relationships of knee and hip OA with obesity-related traits (**Supplementary Table 4**). Besides, no evidence of reverse causality across the analyses in the MR Steiger test (**Supplementary Table 4**).

#### 3.2.6 The Venn diagram

**Figure 6:**
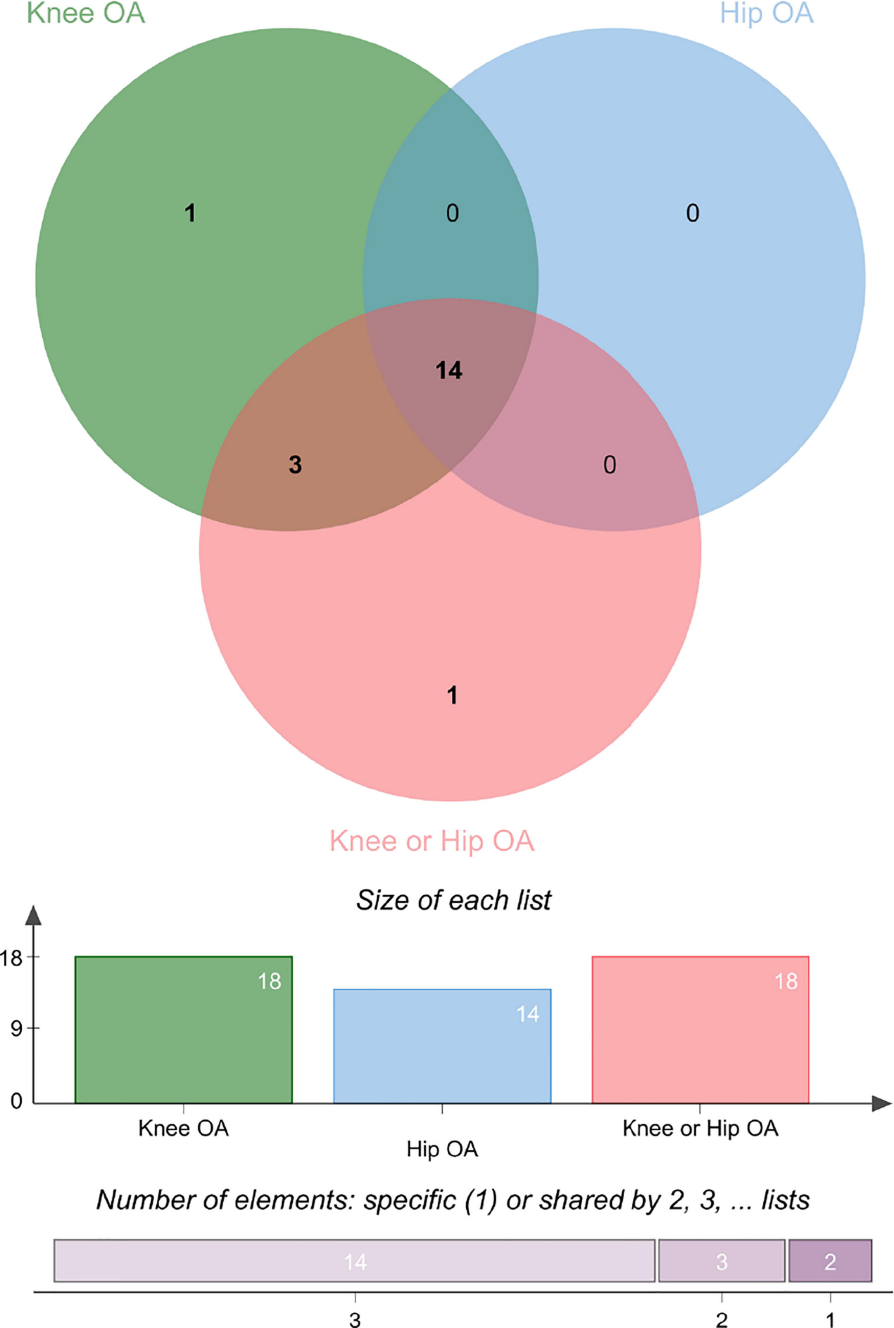
The Venn diagram of the univariable MR on knee and hip OA. See also **Table S5**.

The Venn diagram of the univariable MR was shown in **Figure 6** and **Supplementary Table 5**. The results showed that TFP was an independent risk factor for knee OA. BMI, weight, obesity class (I II III), WC, HC, AFM, AFP, LFM, LFFM, LFP, WBFM and BFP were common risk factors for knee and hip OA.

### 3.3 Multivariable mendelian randomization and mediation analysis

**Figure 7:**
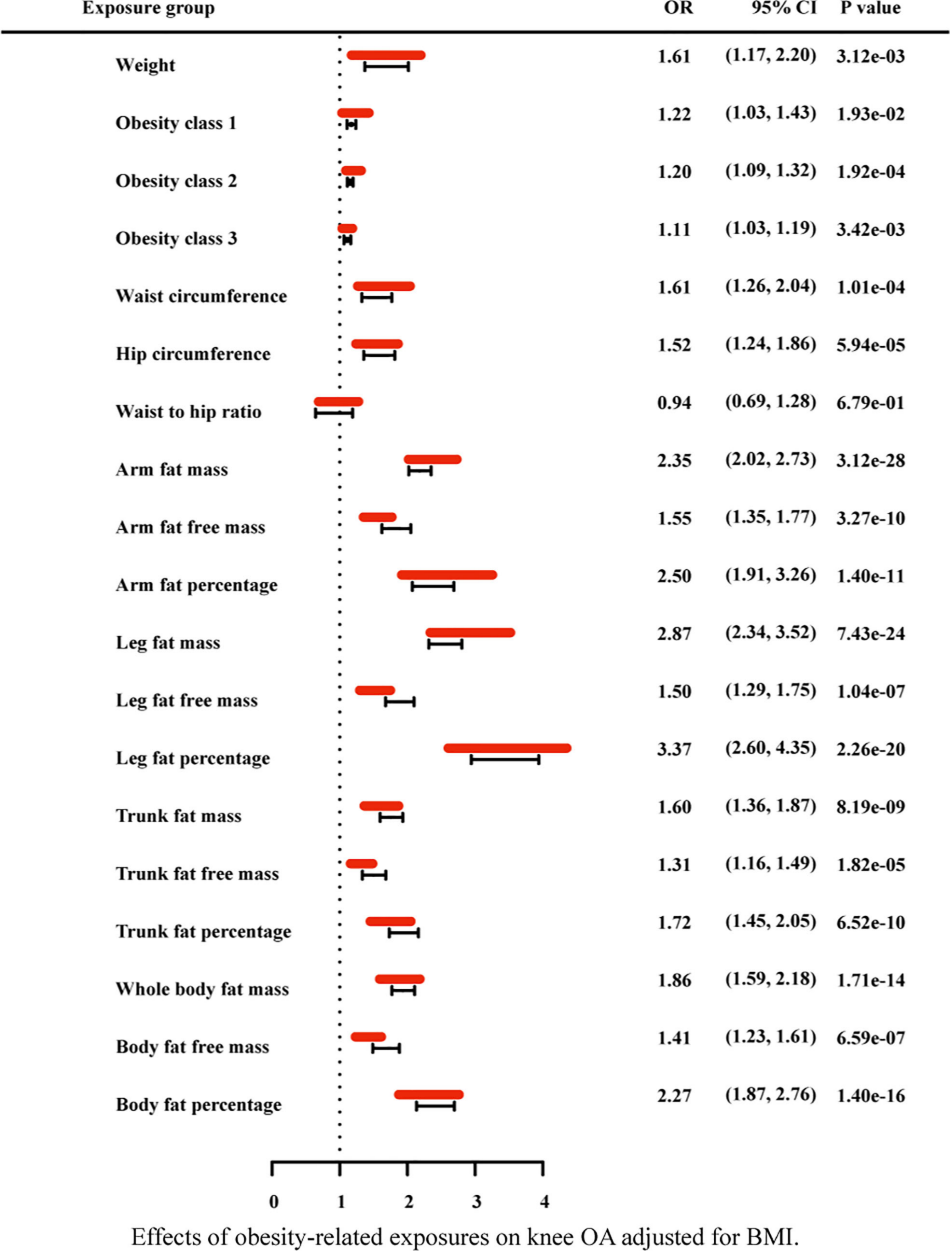
Effects of obesity-related exposures on knee OA adjusted for BMI. Results from multivariable MR analyses showing the effects of genetic predicted obesity related anthropometric indicators and body compositions on knee OA after adjusting for the effect of BMI, represented by red line.

**Figure 8:**
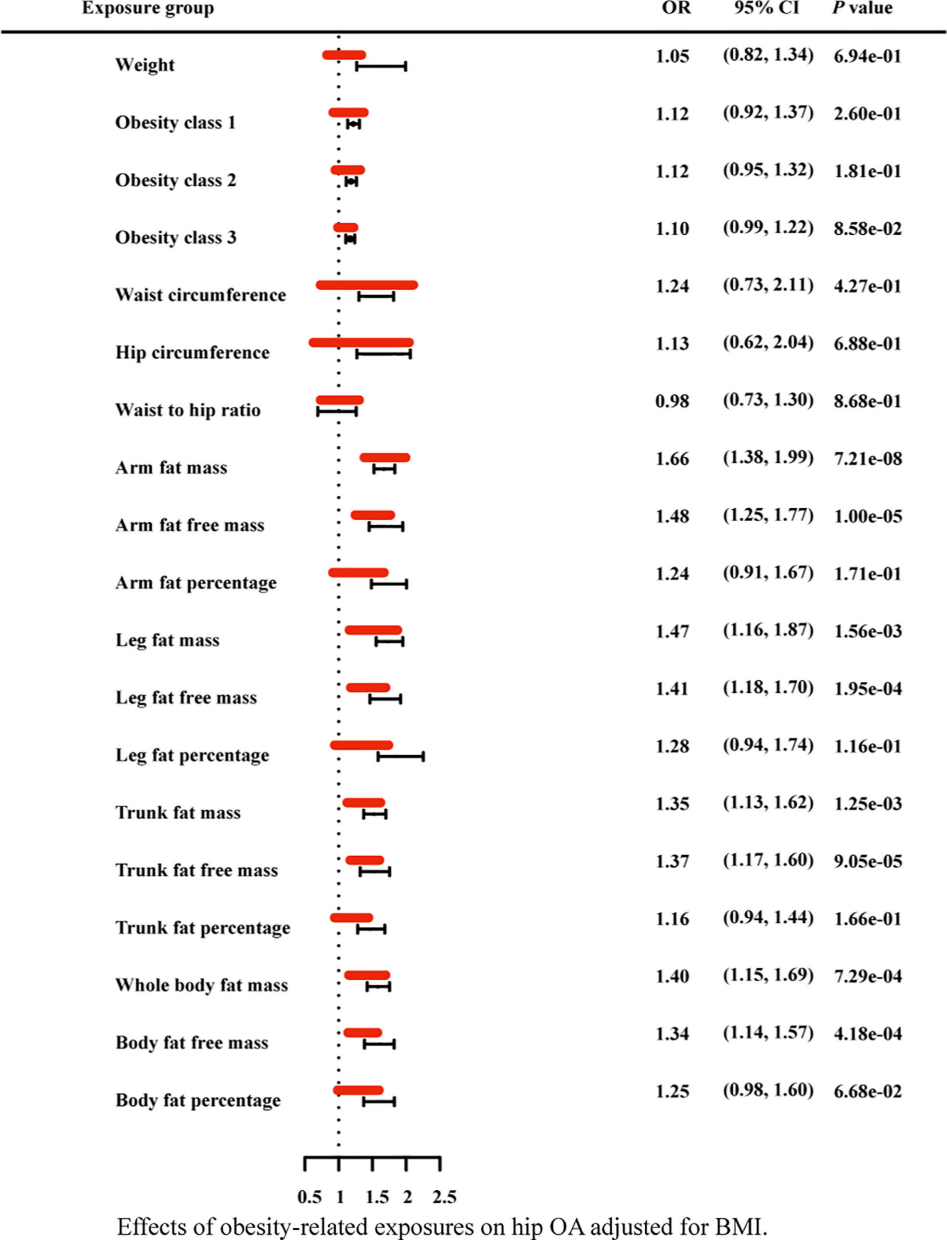
Effects of obesity-related exposures on hip OA adjusted for BMI. Results from multivariable MR analyses showing the effects of genetic predicted obesity related anthropometric indicators and body compositions on hip OA after adjusting for the effect of BMI, represented by red line.

**Figure 9:**
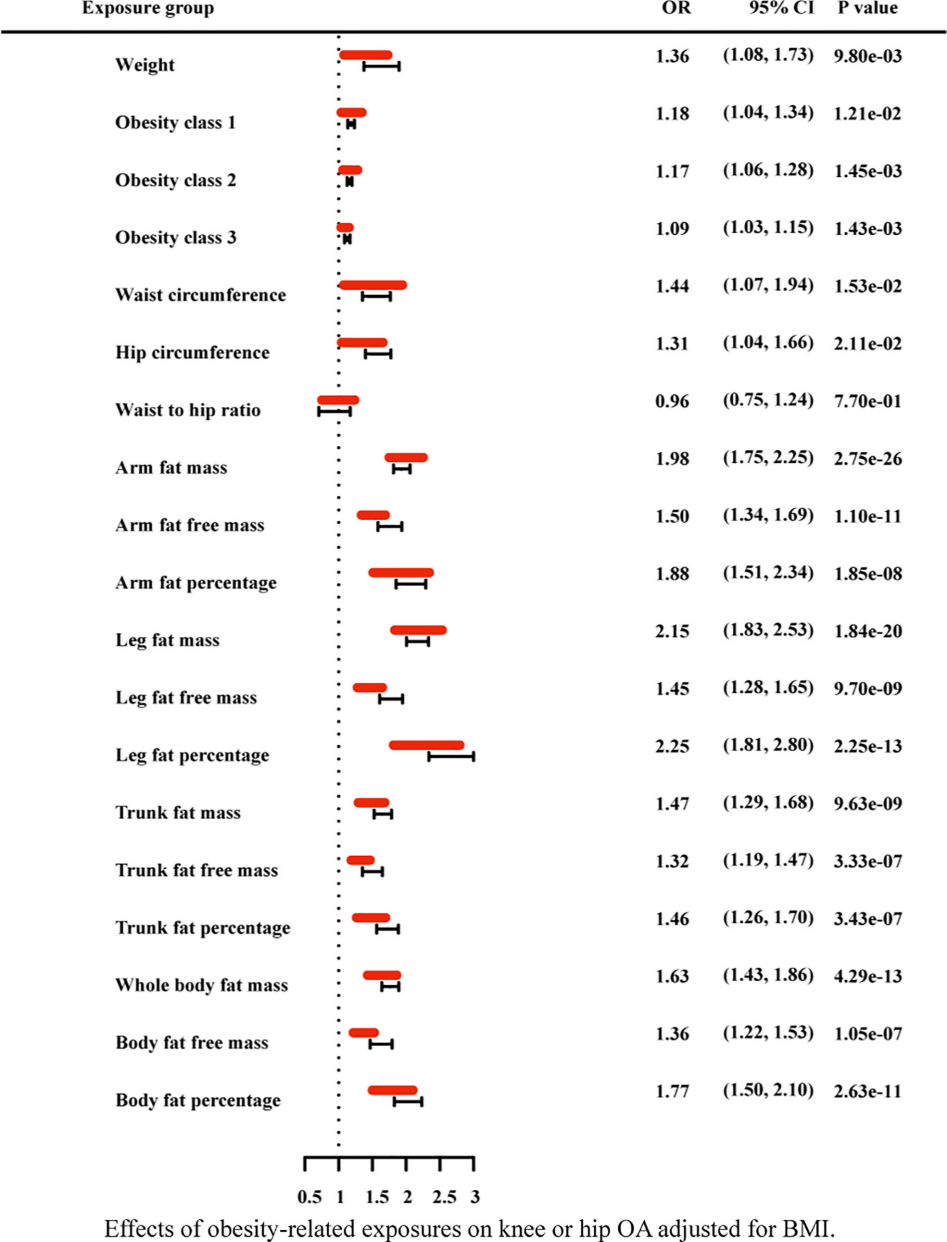
Effects of obesity-related exposures on knee or hip OA adjusted for BMI. Results from multivariable MR analyses showing the effects of genetic predicted obesity related anthropometric indicators and body compositions on knee or hip OA after adjusting for the effect of BMI, represented by red line.

Due to the high relevance of the included obesity-related traits with BMI, we further used multivariable MR analysis to adjust the effects of these traits for BMI (**Figures 7**–**9**). The conditional F-statistics were shown in **Supplementary Table 6**. All the F-statistics were >10, implicating absence of weak instrument bias. Multivariable MR demonstrated that other obesity-related traits except WHR causally influenced knee OA even after controlling for BMI, supporting the results of our univariate MR models. In hip OA, after adjusting for BMI, only AFM, AFFM, LFM, LFFM, TFM, TFFM, WBFM, WBFFM were significantly associated with the risk of hip OA. Finally, we further calculated the proportion of BMI’s effect on the outcomes mediated by other obesity-related traits by using mediation analysis. The results showed that the effect of obesity-related traits was partially mediated by BMI (**Supplementary Table 7**).

## 4 Discussion

At present, many previous studies have attributed obesity (defined by BMI) to the pathogenesis of osteoarthritis (29). However, BMI is not an exclusive measure of adiposity though, but rather reflect the composite of fat, muscle and bone mass. Using the recent large-scale GWAS dataset to date, our study performed LD score regression and two-sample MR analysis to determine the genetic correlation and causal associations of obesity related anthropometric indicators and body compositions with knee and hip OA. We investigate whether osteoarthritis genetic components are shared with obesity related traits, and then significant genetic correlation are found. Besides, as shown in **Figure 1** and **Table 3**, we find all the genetic correlations between obesity and knee osteoarthritis are higher than with hip osteoarthritis, such as BMI in knee OA (rg =0.36, *p*=1.11×10^-20^) and hip OA (rg=0.21, *p*=1.08×10^-06^).

It is well known that osteoarthritis is one of the most common clinical diseases. A number of reviews and clinical studies on the epidemiology of OA have been conducted in the past decades, which had a similar conclusion with our results that obesity has been considered as a risk factor for knee OA (29). However, the relationship between obesity defined by body mass index (BMI) and hip OA remains controversial in many cross-sectional and longitudinal studies. Some previous studies have shown that the association between obesity and hip OA is weak (1, 9, 10). However, recently a prospective cohort study has found that there is an independent association between weight gain and the diagnosis of hip OA (5), which is consistent with our result. In the subsequent studies, WC, HC and WHR were studied separately from BMI.

In addition, conflicting results have emerged regarding the relationship between the body compositions of adiposity (fat mass/muscle mass) and osteoarthritis. Where between fat mass or fat percentage and knee OA or knee cartilage, some studies reported a negative association (30, 31), other studies did not find an association (32). For fat-free mass (FFM), it accounts for a large part of muscle mass and has been shown to be related to both muscle mass and muscle strength. Therefore, FFM is a valuable representative of muscle mass (33). A previous population-based cohort study showed that low fat free mass may interact with knee OA, resulting in an additional reduction in health-related quality of life (HRQOL) in patients with knee OA (34). A cross-sectional study using the data from Korea National Health and Nutrition Examination Survey (KNHANES) showed that the low percentage of lower limb muscle mass was more correlated with knee OA in women (*p* < 0.05) (35). Another study found that the higher the total extensor muscle cross-sectional area (CSA) and vastus medialis CSA, the greater the loss of patellofemoral cartilage over time (36). In a longitudinal study registered in Sweden, the higher the knee extensor strength of adolescent men, the greater the risk of knee OA in middle age was found (37). While in the cross-sectional study of hip joint muscle strength and joints in subjects with hip OA, better self-reported physical function was found in the greater isometric muscle strength of hip joint and thigh muscle group (38). However, from the results of Venn and MR in our study, that AFFM, TFFM and WBFFM did not meet the definition of potential causal effects, while interestingly, LFFM was found to be significantly associated with both OA of knee and hip.

The difference between our results of MR analysis and epidemiological studies may be due to: (1) Small sample size of the epidemiological study; (2) Confounding factors linked to the chosen exposure and outcome in epidemiological study; (3) Some epidemiological studies only discussed FFM, but not subdivided it into AFFM, TFFM, WBFFM and LFFM; (4) There may be a reverse causal relationship in epidemiological study, for example, low muscle mass may also be a consequence of knee OA, since OA related knee pain leads to muscle abandonment.

### 4.1 Mechanisms underlying genetic causal links

Some interesting results are also obtained from this MR analysis. The MR result of BMI is consistent with most previous studies, that is, obesity defined by BMI is a risk factor for both knee and hip OA. Regarding the body compositions of adiposity, the results of MR and Venn have showed that AFM, AFP, WBFM, BFP, LFM and LFP are all risk factors for knee and hip OA. Due to a strong correlation (39), AFP and BFP can be used to measure the overall fat content, so as to replicate the results of BMI. TFP can be used to study the effect of visceral fat content on OA, while LFP represents the fat content of legs. These results suggest that the fat mass predicted by genes is highly correlated with the occurrence of OA. Obese adipose tissue seems to create a chronic inflammatory environment. Specific biological mechanisms to explore the link between obesity and OA risk are centered along metabolic and endocrine consequences of body fat accumulation. In basic research, previous studies have attributed the effect of adipose tissue to the adipokines it produces, such as leptin, resistin and adiponectin. Studies on non-weight-bearing arthritis in hands or fingers may support this hypothesis (40). Meanwhile, many studies have proved that adipose tissue can produce inflammatory cytokines such as IL-1, IL-6, and TNF-a. Elevated blood IL-6 levels were thought to be associated with decreased physical function and frailty (41), while higher levels of IL-6 were found to be associated with an increased risk of knee OA progression (42). The serum levels of IL-6 and TNF-a were correlated with articular space stenosis and cartilage loss in the elderly (43). Adipokines and inflammatory cytokines secreted by adipose tissue participate in cartilage degradation, synovitis and bone erosion, leading to OA (44), which is consistent with our study results. However, in this paper, TFP is only related to knee OA, but not hip OA. Whether this discrepancy is related to the different internal structures of knee and hip joint deserves further study. What’s more, as proxy for muscle mass, LFFM is found to be significantly correlated with knee and hip OA, and the correlation still existed after adjusting BMI by multivariable MR analysis. It indicates that genetic predicted LFFM is a risk factor for both knee and hip OA. Recently, skeletal muscle has been identified as an endocrine organ. We believe that some cytokines and inflammatory factors produced by muscle tissue may also lead to OA in knee and hip joints, such as IL-6 (45, 46).

As for the anthropometry indicators of obesity, we find an interesting phenomenon, that is, in univariable MR analysis, with the increase of obesity class (I II III), the odds ratio of both hip OA and knee OA gradually decreased (still greater than 1). In general, abnormal biomechanical loading is considered to be the main mechanism of obesity induced osteoarthritis. According to this result, we consider that, with the gradual increase of BMI, there may be some factors that weaken the effect of biomechanical loading. Of course, this does not exclude chances. Further research could explore this phenomenon. In addition, after the adjustment for BMI with multivariable MR, WC, HC, weight, obesity class (I II III) still have statistical significance in knee OA, while these causal effects disappeared in hip OA. It indicates that the other anthropometric indicators of obesity in our study, except WHR, are strongly correlated with knee OA independent from BMI.

Although the exact pathophysiological mechanism of obesity leading to articular cartilage degeneration is still unclear, mechanical factors may play a partial role in the pathogenesis of OA. With obesity, being overweight will increase the joint load, resulting in a detrimental effect on the weight-bearing joints (47). Maquet et al. (48) found that two to five times a person’s body weight is transmitted across the knee joint. Reilly et al. (49) showed that the reaction force of the entire patellofemoral joint increases from three times the weight when climbing or descending stairs to seven to eight times the weight when squatting. Gait analysis showed that each pound of weight loss could reduce the load on the knee joint by four times (50). Articular cartilage lacks regenerative ability when it bears long-term abnormal mechanical loads, which is prone to degenerative lesions to osteoarthritis. A review of mechanical loading on osteoarthritis showed that mechanical load activated multiple inflammatory pathways, such as IL-1β, TNF-α, inducing chondrocyte apoptosis, synovial inflammation and subchondral bone dysfunction, finally leading to OA (51). In conclusion, obese patients have a higher risk of osteoarthritis related outcomes due to higher mechanical forces exerted on weight-bearing joints under excessive mechanical loading conditions.

### 4.2 Strengths and limitations

In previous study, Boer et al. (52) found that there was a significant correlation between the genetic components of osteoarthritis and anthropometric traits (BMI, obesity, weight and fat mass) through LD score regression, which was consistent with our research results, but it did not study the relationship between osteoarthritis and other anthropometric traits such as waist circumference, hip circumference or waist-to-hip ratio, nor the genetic correlation between OA and fat mass or fat-free mass in different parts. Previous MR researches have suggested potential associations between BMI and OA (2, 53, 54). With regard to the relationship between other obesity related measurement indicators and OA, recent MR studies (53) indicated that some anthropometric traits such as waist circumference or hip circumference may display modest, but significant, causal associations with OA. However, few studies have adjusted for the effect of BMI through multivariable MR analysis. What is more, no known empirical research has focused on exploring relationships between OA and fat mass, fat free mass or fat mass percentage in different parts. Therefore, we have conducted a more comprehensive investigation.

This study has several strengths. First, this is a more comprehensive study used large-scale GWAS datasets to assess the genetic effect of both anthropometry indicators and body compositions of obesity on knee or hip OA through LD score regression and two-sample MR analysis. Second, given the advantages of MR analysis, it is less likely to be interfered by confounding bias and reverse causality compared with epidemiological studies. Third, we defined strict evidence of potential causal effects, robust methods enabled us to reinforce findings by considering different patterns of pleiotropy and the multivariable method allowed us to distinguish the direct effects of exposures.

However, there are certain limitations in this study. A part of sample overlaps may be biased in the direction of the observational association between risk factors and outcomes. However, a recent study had shown that for one single large dataset, such as the UK Biobank, main two-sample MR methods can be safely used, except MR-Egger, because of its bias (23). Therefore, we define a more rigorous potential causal effects to reduce the possible bias. Meanwhile, we have performed a recently developed MR method, MRlap, to correct the bias introduced by sample overlap. MRlap revealed almost identical results compared to our primary analysis, suggesting sample overlap may not substantially bias estimates. However, as MR-IVW estimates, the corrected results will also be biased if the genetic confounder, through which some of the IVs affect the exposure of interest, is existence. In our study, to avoid the genetic confounder, also called exchangeability or independence, we select the IV with a strict criterion as mentioned in the method section. What’s more, this analytical derivation hinges on a genetic architecture of the exposure, which could reduce the efficiency of the bias correction. However, the effect of the bias will decrease as the sample size increases, and for very large sample size, such as UKBB, the bias is expected to be less pronounced. In addition, since the data in the database includes both male and female, this study does not research the sex difference of obesity on OA. As we all recognized, there are differences in fat, muscle and bone mass between men and women, which may lead to changes in the results. It is also worth studying separately in the future. Finally, the population is restricted to individuals of European ancestry in this study, which limits the transferability to other ethnicities worldwide.

## 5 Conclusion

Osteoarthritis remains the leading cause of global morbidity and medical costs. Various individual and joint level risk factors are associated with the development and progression of the disease. In this paper, the results of LD score regression have showed the genetic correlation between obesity and osteoarthritis. Mendelian randomization analysis is used to minimize confounding and to avoid reverse causality, and further confirm the effect of genetic predicted anthropometry and body compositions of obesity on knee or hip OA, which may help to better understand the role of BMI, WC, HC, fat and muscle in OA, and finally prevent the occurrence of OA.

## Data availability statement

The original contributions presented in the study are included in the article/**Supplementary Material**. Further inquiries can be directed to the corresponding author.

## Ethics statement

All analyses were based on publicly shared databases and no additional ethical approvals were required.

## Author contributions

CW and YZ contributed equally to this work. CW, YZ, and SZ designed the study. SZ and ZL performed the analyses. CW, YZ, ZL, ZR, and SZ analyzed data. CW, YZ, HL, and SZ interpreted the data, wrote the manuscript, and revised the manuscript. All authors approve of the final version of the manuscript.

## Funding

This work was financially supported by the National Natural Science Foundation of China (Nos. 81902222, and Nos. 82172399) and the Natural Science Foundation of Hunan Province (2020JJ4928).

## Conflict of interest

The authors declare that the research was conducted in the absence of any commercial or financial relationships that could be construed as a potential conflict of interest.

## Publisher’s note

All claims expressed in this article are solely those of the authors and do not necessarily represent those of their affiliated organizations, or those of the publisher, the editors and the reviewers. Any product that may be evaluated in this article, or claim that may be made by its manufacturer, is not guaranteed or endorsed by the publisher.

## Supplementary material

Supplementary Table 1 Results of I^2^ GX of the overlapped sample and results of MRlap.

Supplementary Table 2 Extra information about the selected related SNPs.

Supplementary Table 3 Results of the Cochran’s Q, MR-Egger-intercept and MR-PRESSO tests for detecting horizontal and directional pleiotropy and between SNP-heterogeneity in the univariable Mendelian randomization setting and results of the MR analysis after SNPs screened by PhenoScanner V2.

Supplementary Table 4 Results of the bidirectional mendelian randomization and MR steiger test.

Supplementary Table 5 The results of Venn diagram of the univariable MR.

Supplementary Table 6 Results of the conditional F-statistics in multivariable MR.

Supplementary Table 7 Results of mediation analysis.

## Abbreviations

WC, waist circumference; HC, hip circumference; WHR, waist-to-hip ratio; BMI, body mass index; AFM, arm fat mass; AFFM, arm fat-free mass; AFP, arm fat percentage; LFM, leg fat mass; LFFM, leg fat-free mass; LFP, leg fat percentage; WBFM, whole body fat mass; WBFFM, whole body fat-free mass; BFP, body fat percentage; TFM, trunk fat mass; TFFM, trunk fat-free mass; TFP, trunk fat percentage; OA, osteoarthritis; MR, Mendelian randomization; GWAS, genome-wide association studies; SNPs, single nucleotide polymorphisms; GIANT, Genetic Investigation of Anthropometric Traits; LDSC, linkage disequilibrium score regression; IVs, instrument variables.
