# Supplementary material for: Causal associations of obesity related anthropometric indicators and body compositions with knee and hip arthritis: A large-scale genetic correlation study
Source: Front Endocrinol (Lausanne). 2022 Sep 29;13:1011896. doi: 10.3389/fendo.2022.1011896 (PMC9556900; doi:10.3389/fendo.2022.1011896)
Supplement: Supplementary file 8 [file DataSheet_1.docx]

Supplementary Figure 1: Funnel plot of the causal effect of arm fat-free mass on hip OA.


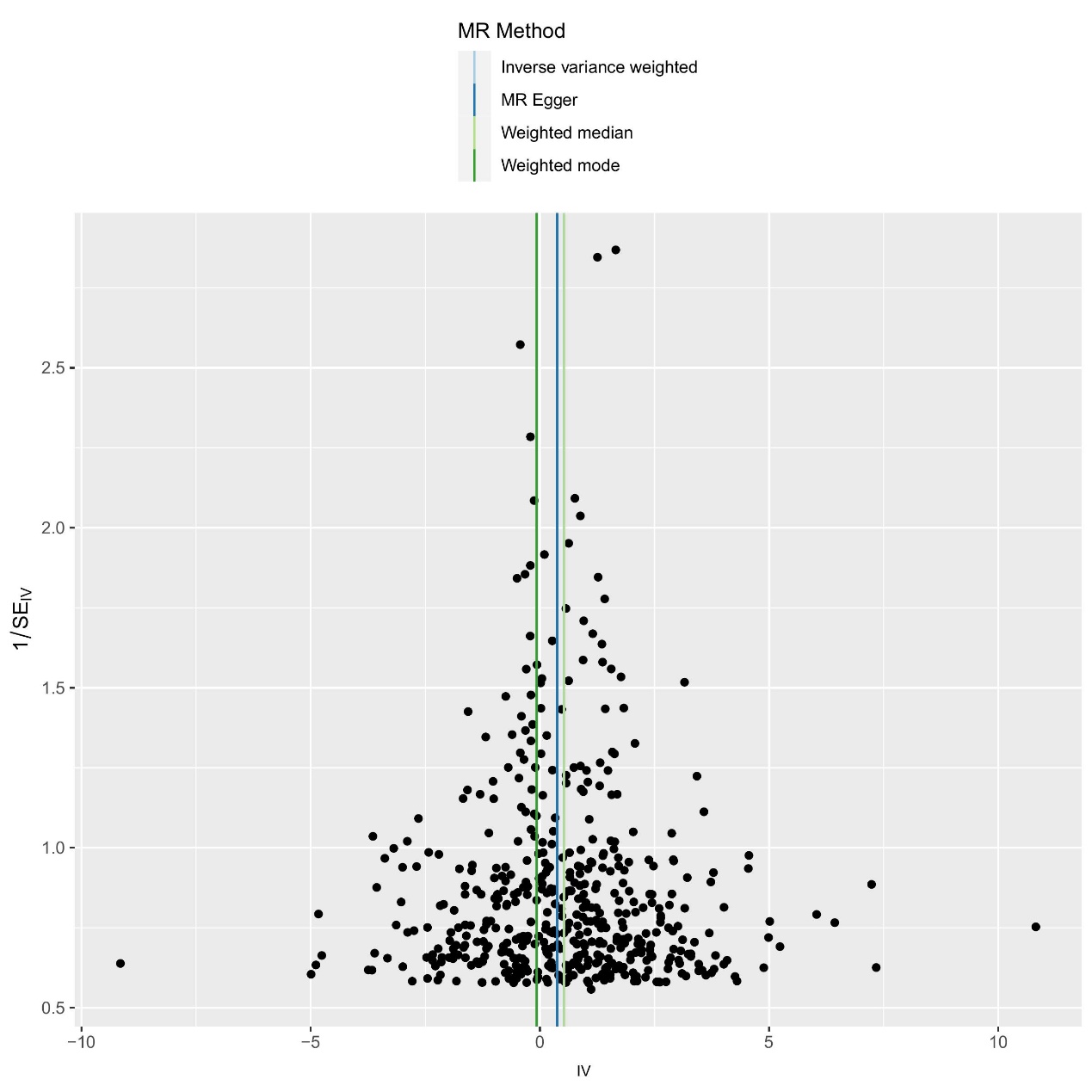


Supplementary Figure 2: Scatter plot of the causal effect of arm fat-free mass on hip OA.


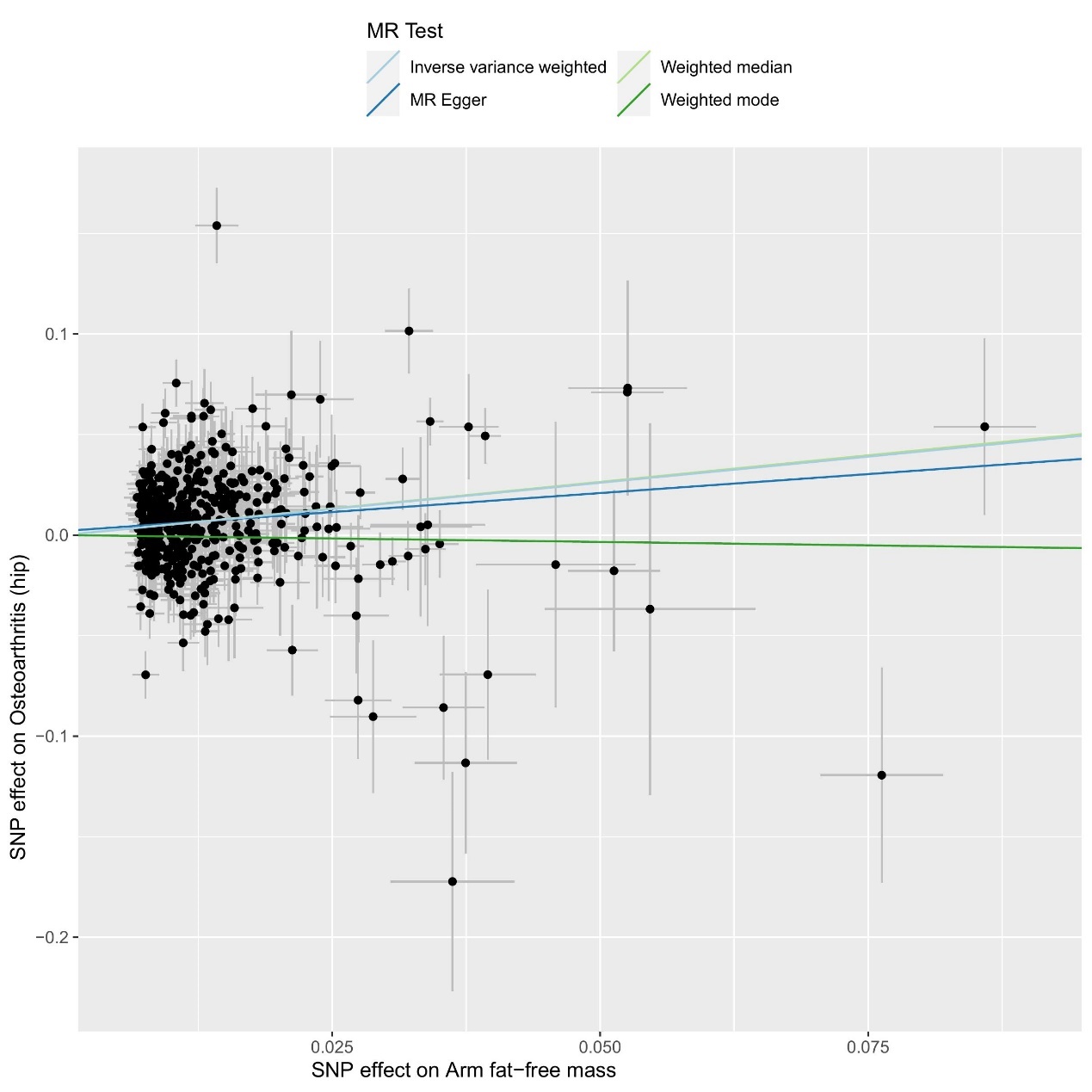


Supplementary Figure 3: Funnel plot of the causal effect of arm fat-free mass on knee or hip OA.


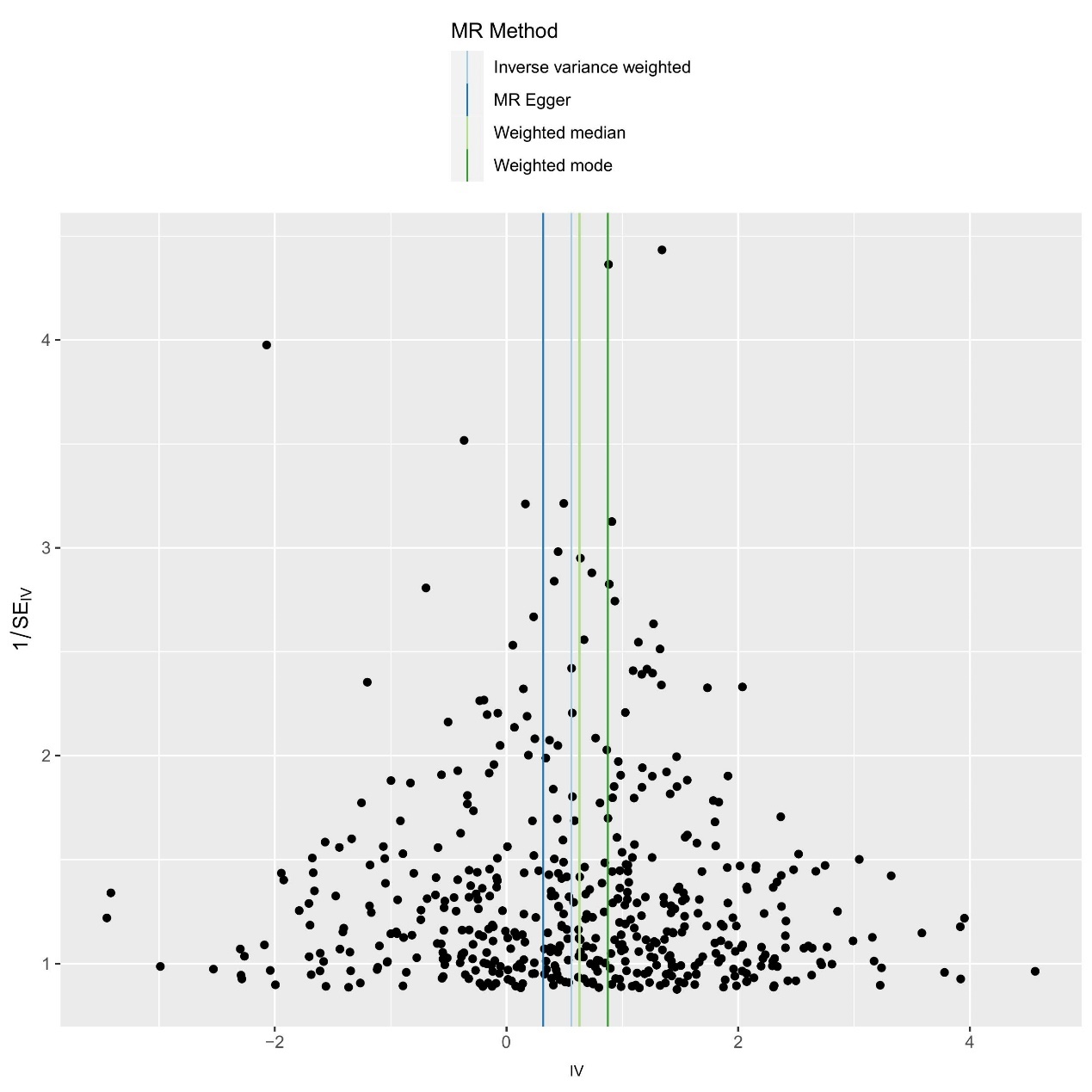


Supplementary Figure 4: Scatter plot of the causal effect of arm fat-free mass on knee or hip OA.


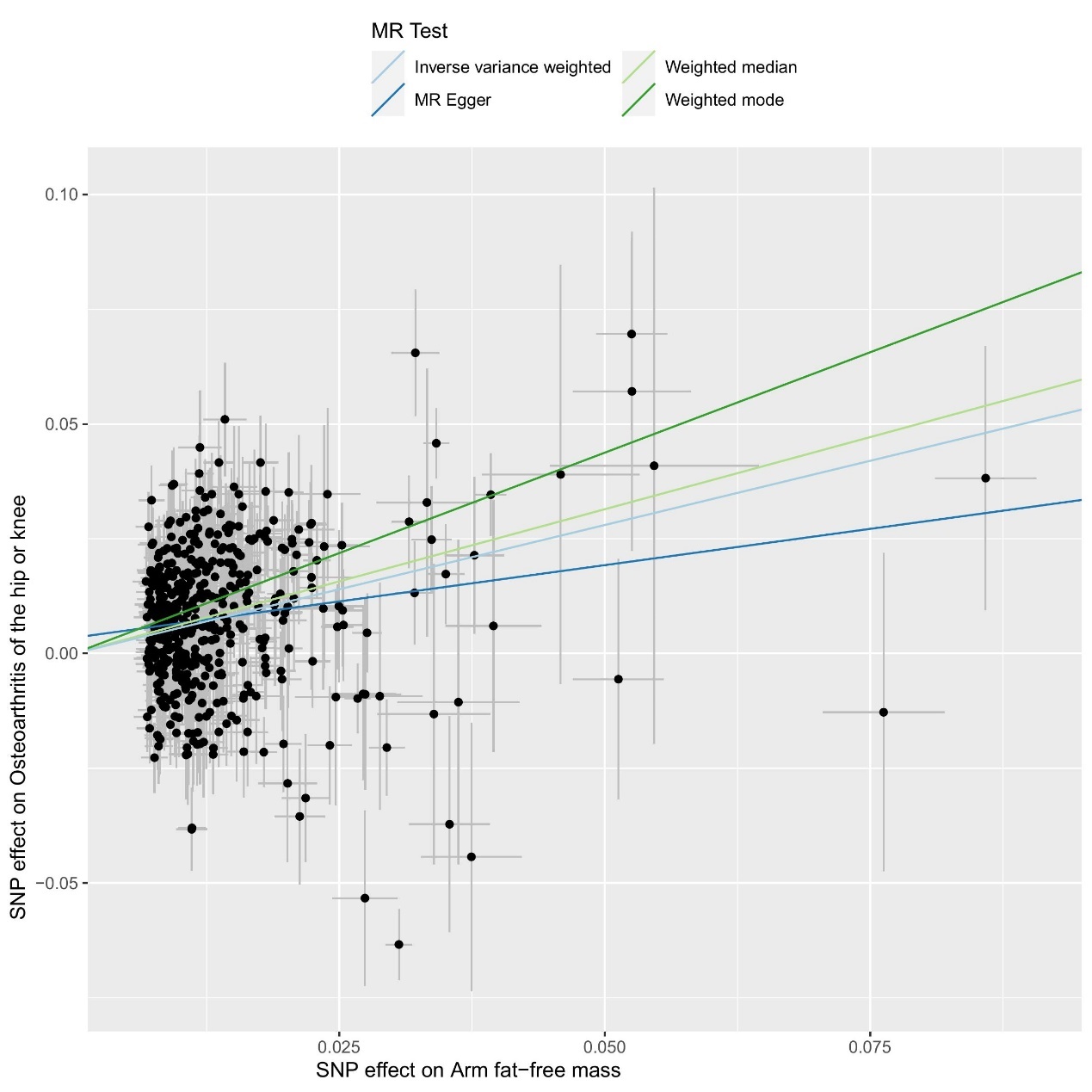


Supplementary Figure 5: Funnel plot of the causal effect of arm fat-free mass on knee OA.


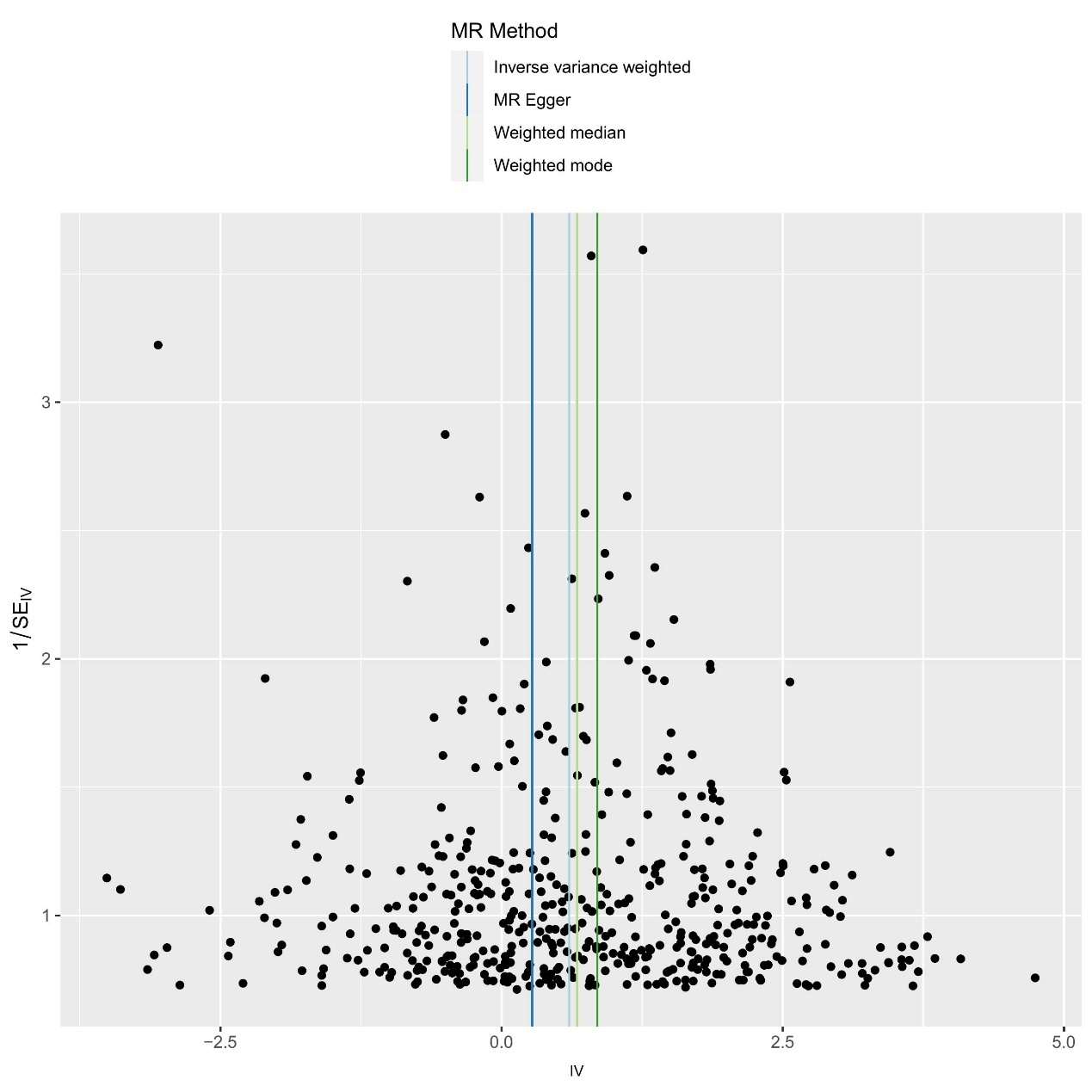


Supplementary Figure 6: Scatter plot of the causal effect of arm fat-free mass on knee OA.
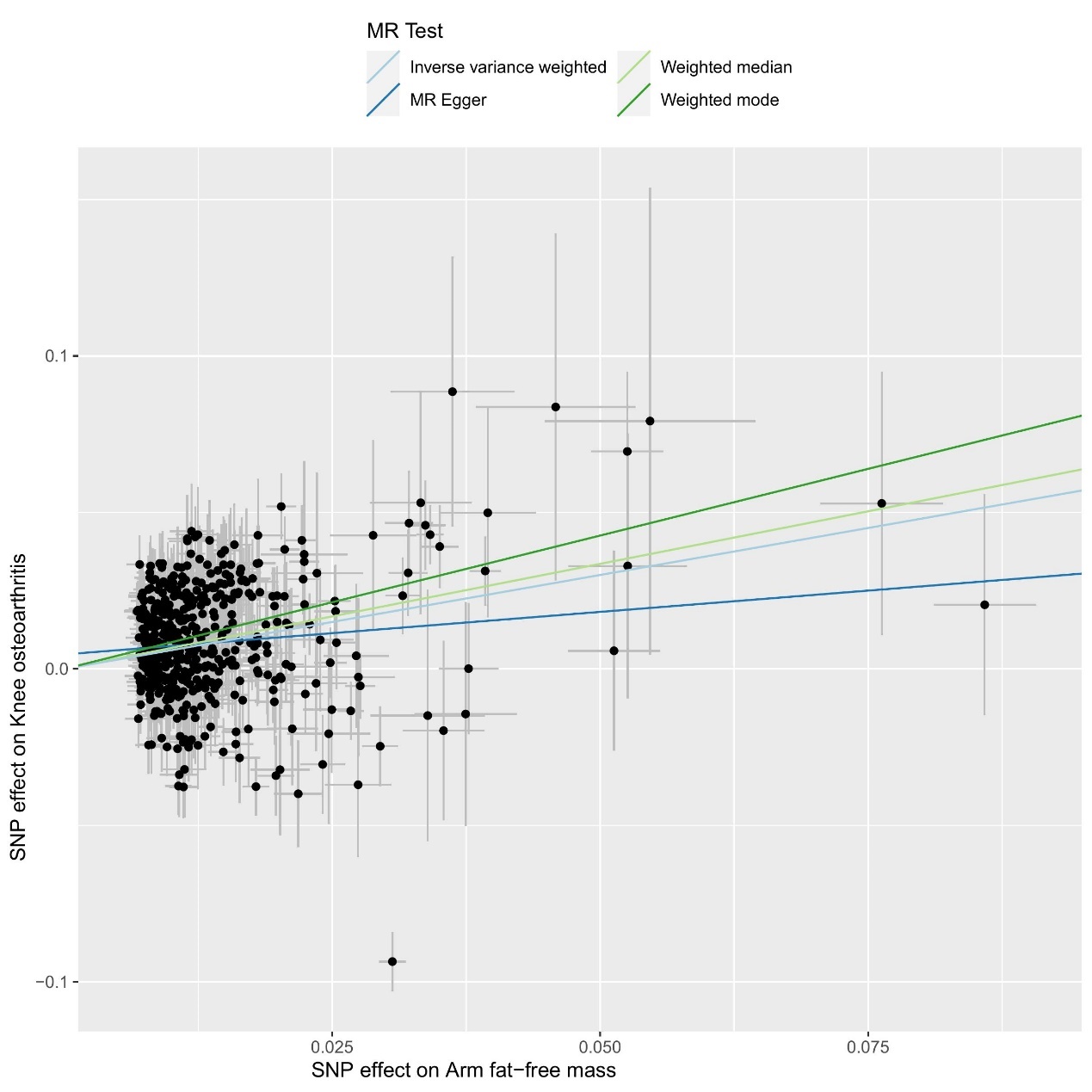


Supplementary Figure 7: Funnel plot of the causal effect of arm fat mass on hip OA.


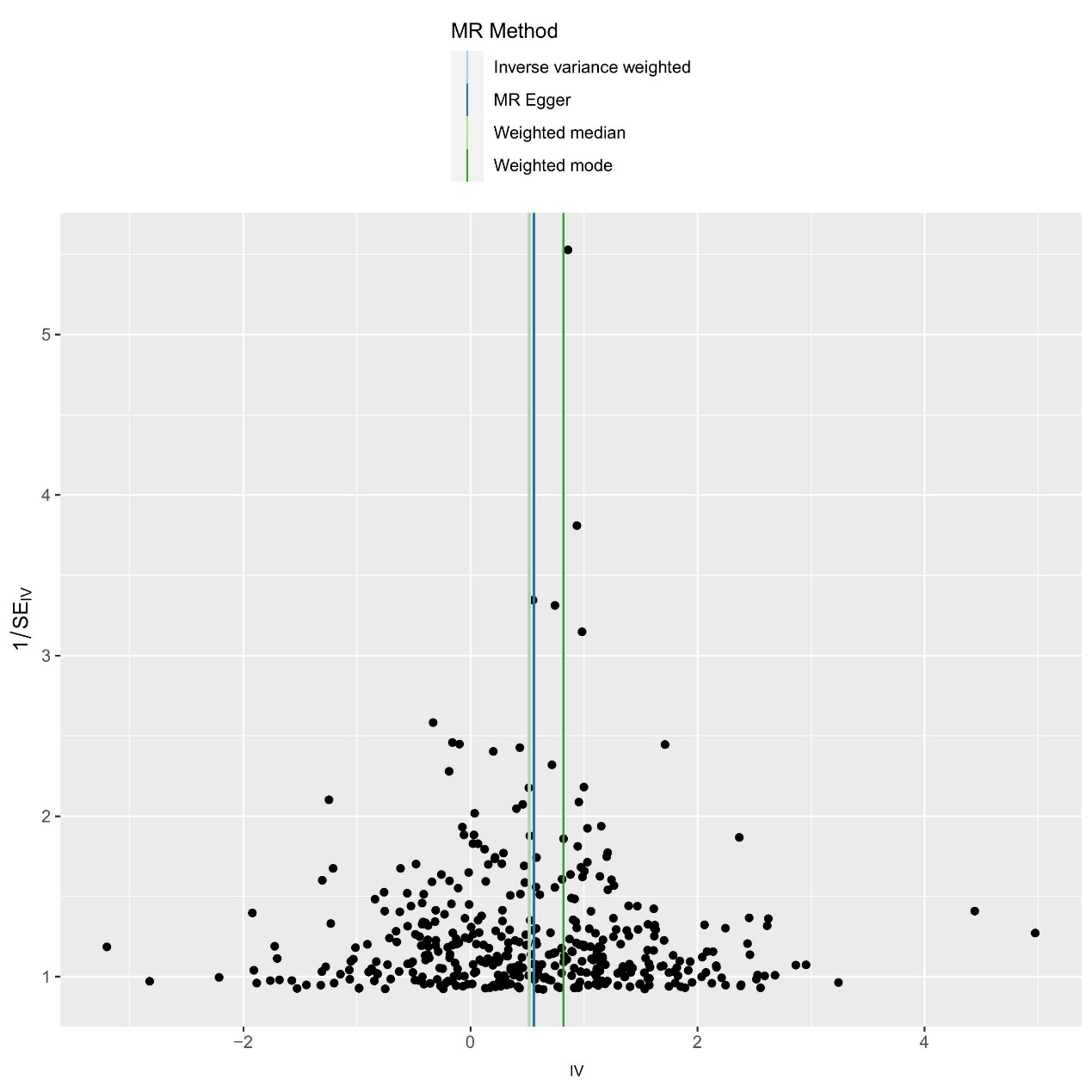


Supplementary Figure 8: Scatter plot of the causal effect of arm fat mass on hip OA.


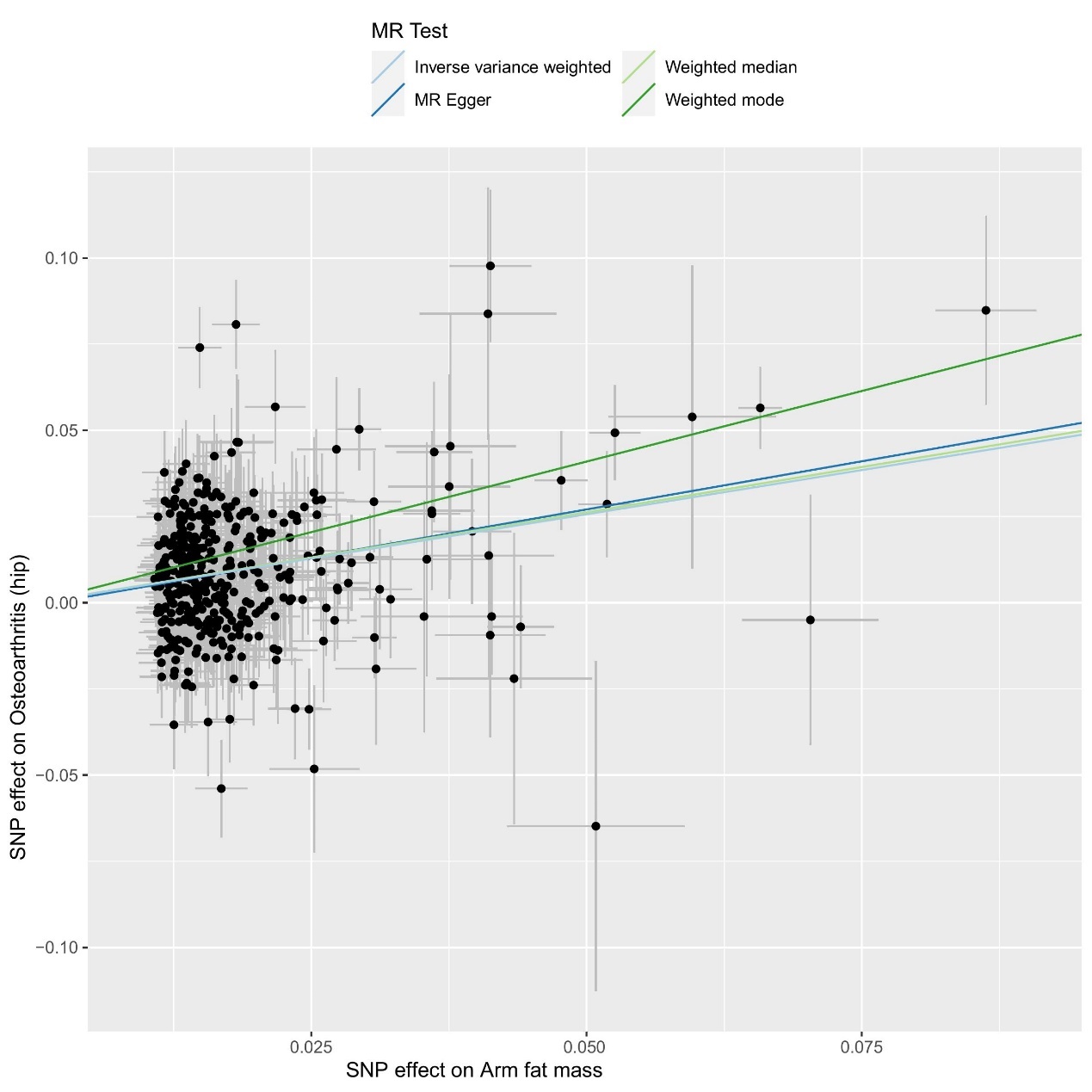


Supplementary Figure 9: Funnel plot of the causal effect of arm fat mass on knee or hip OA.


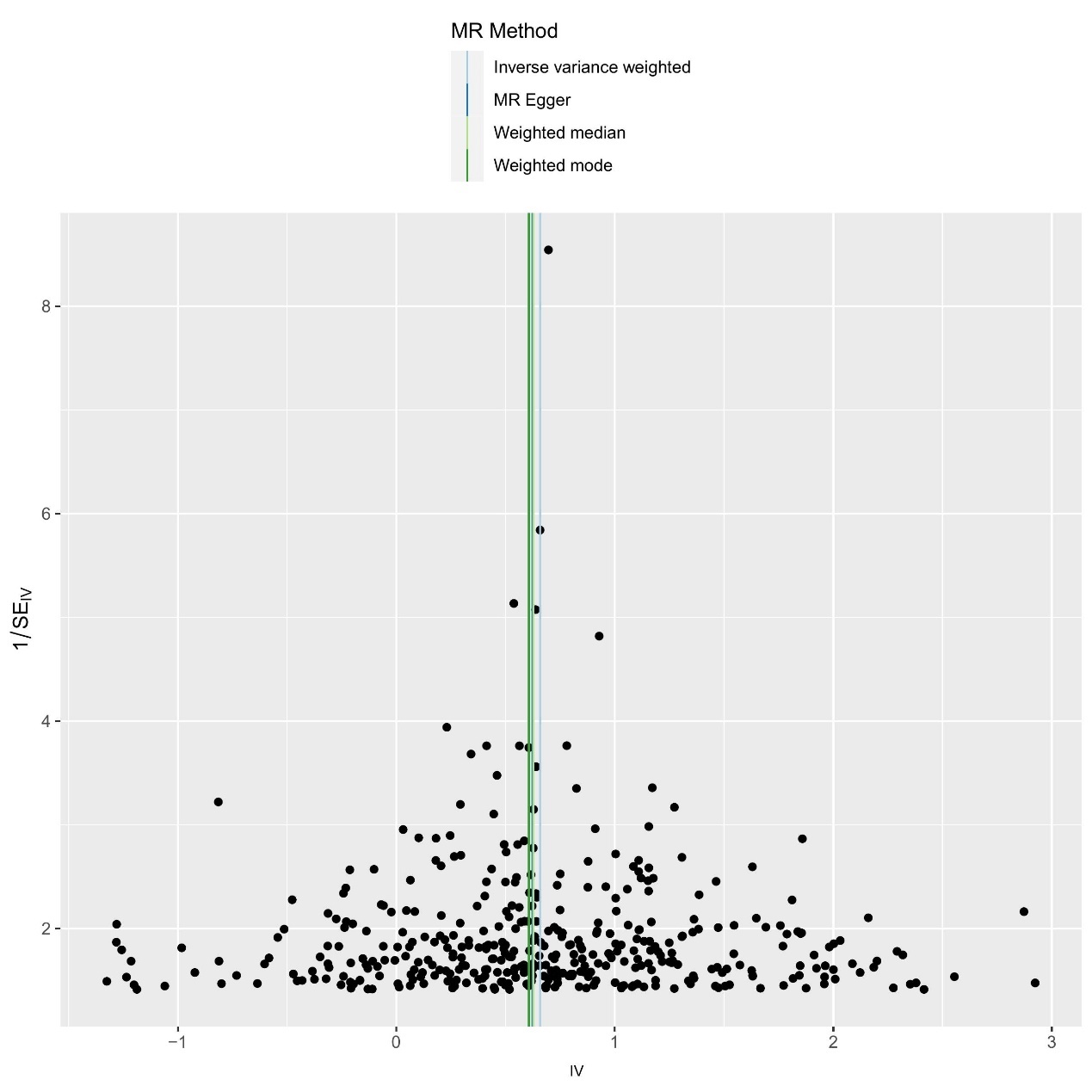


Supplementary Figure 10: Scatter plot of the causal effect of arm fat mass on knee or hip OA.
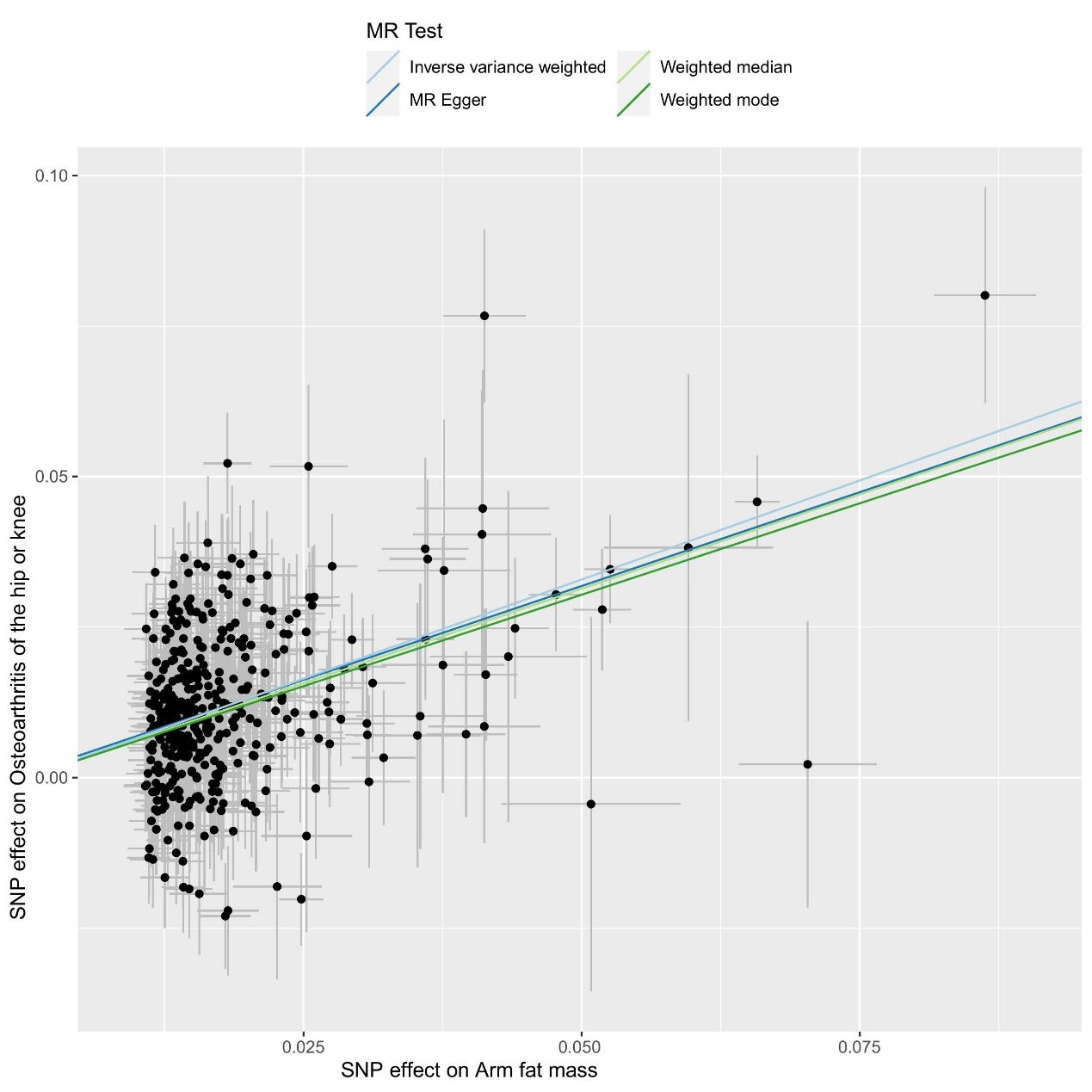


Supplementary Figure 11: Funnel plot of the causal effect of arm fat mass on knee OA.
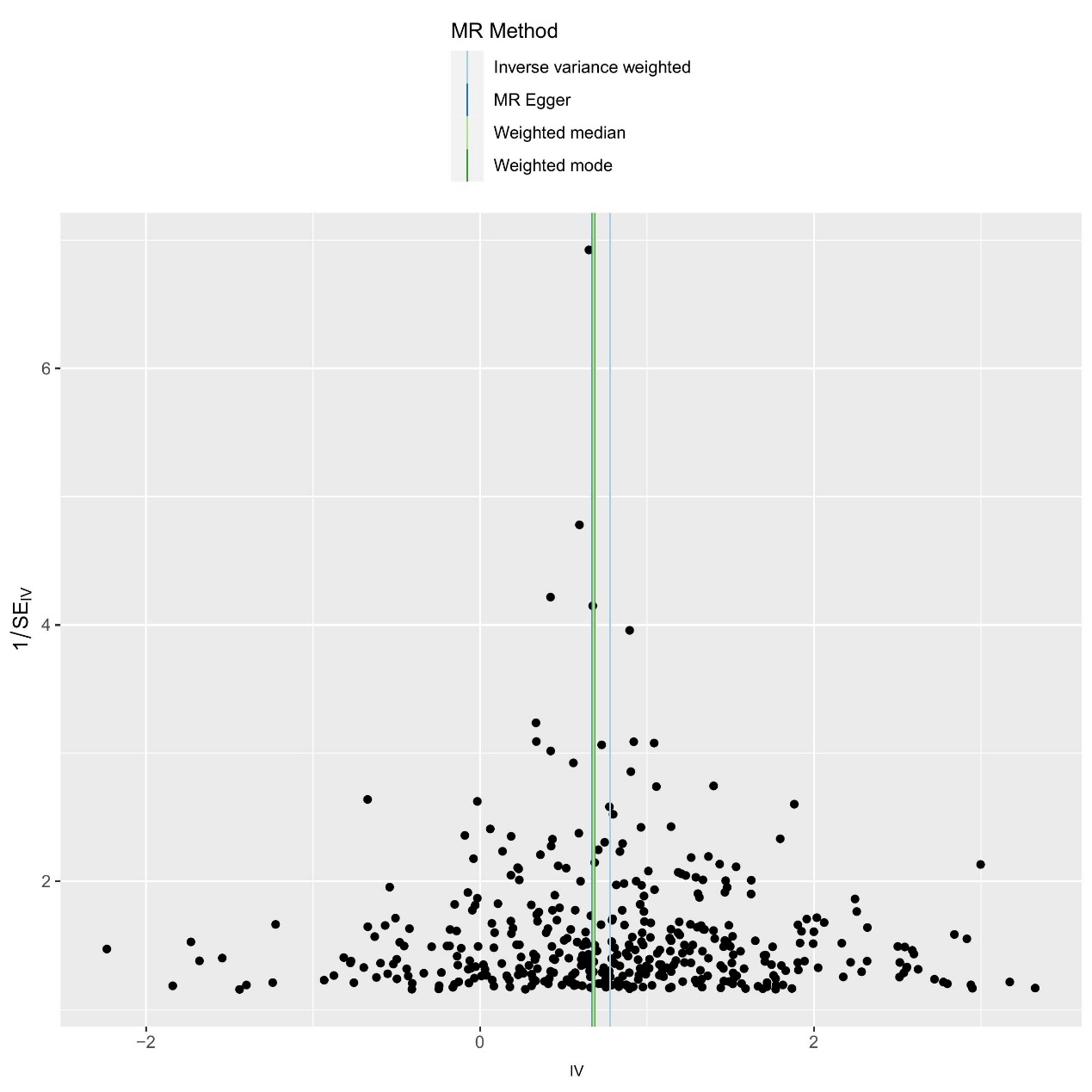


Supplementary Figure 12: Scatter plot of the causal effect of arm fat mass on knee OA.


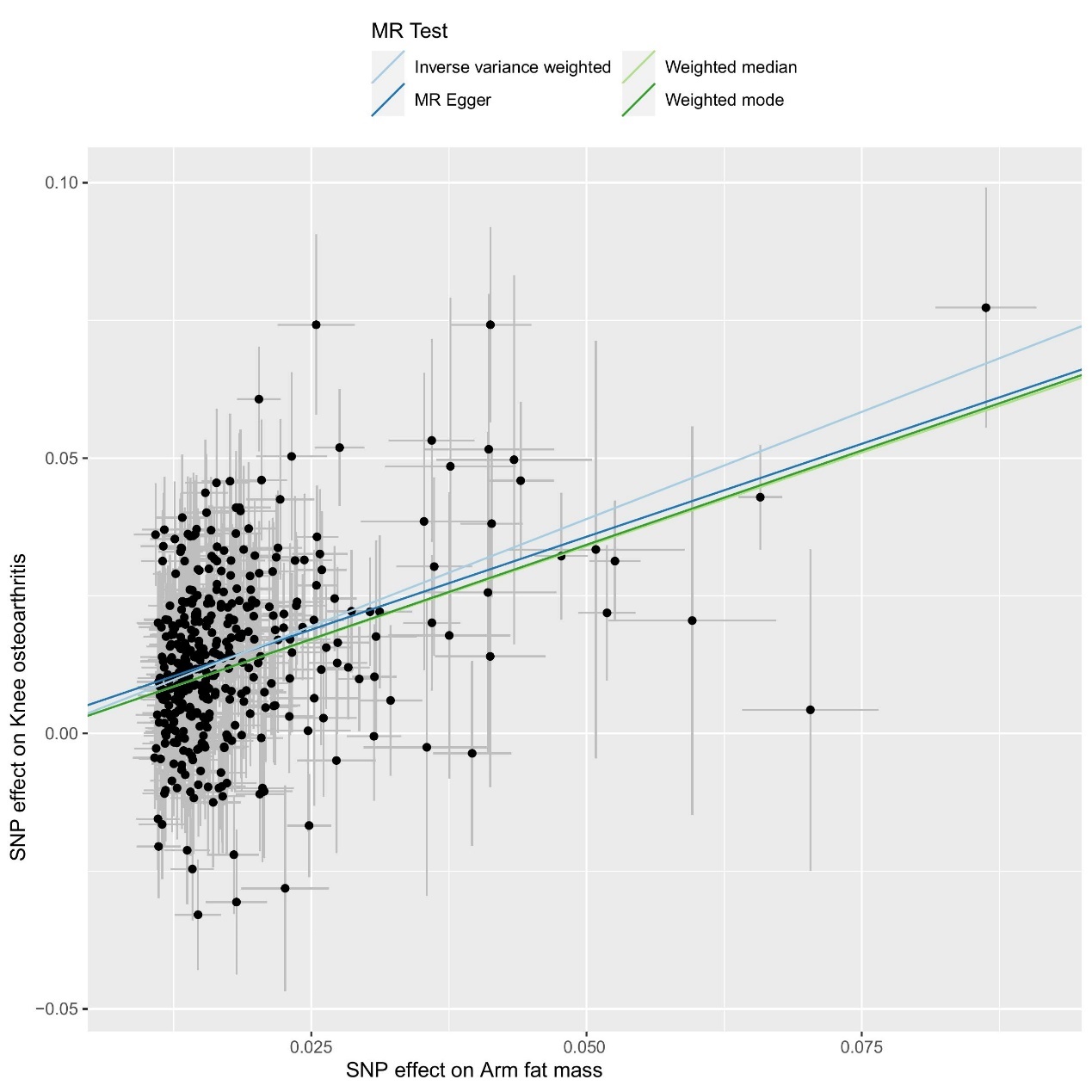


Supplementary Figure 13: Funnel plot of the causal effect of arm fat percentage on hip OA.


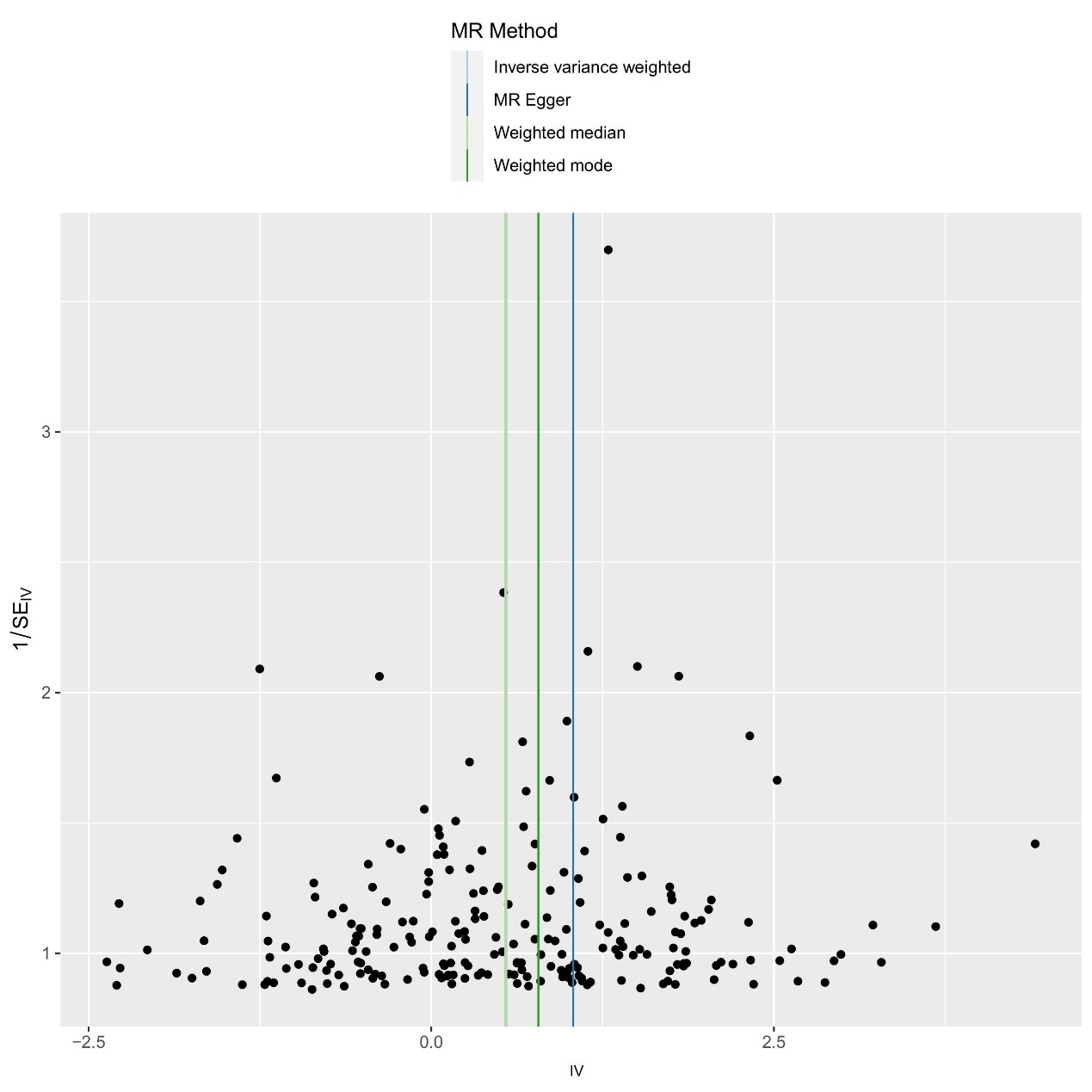


Supplementary Figure 14: Scatter plot of the causal effect of arm fat percentage on hip OA.


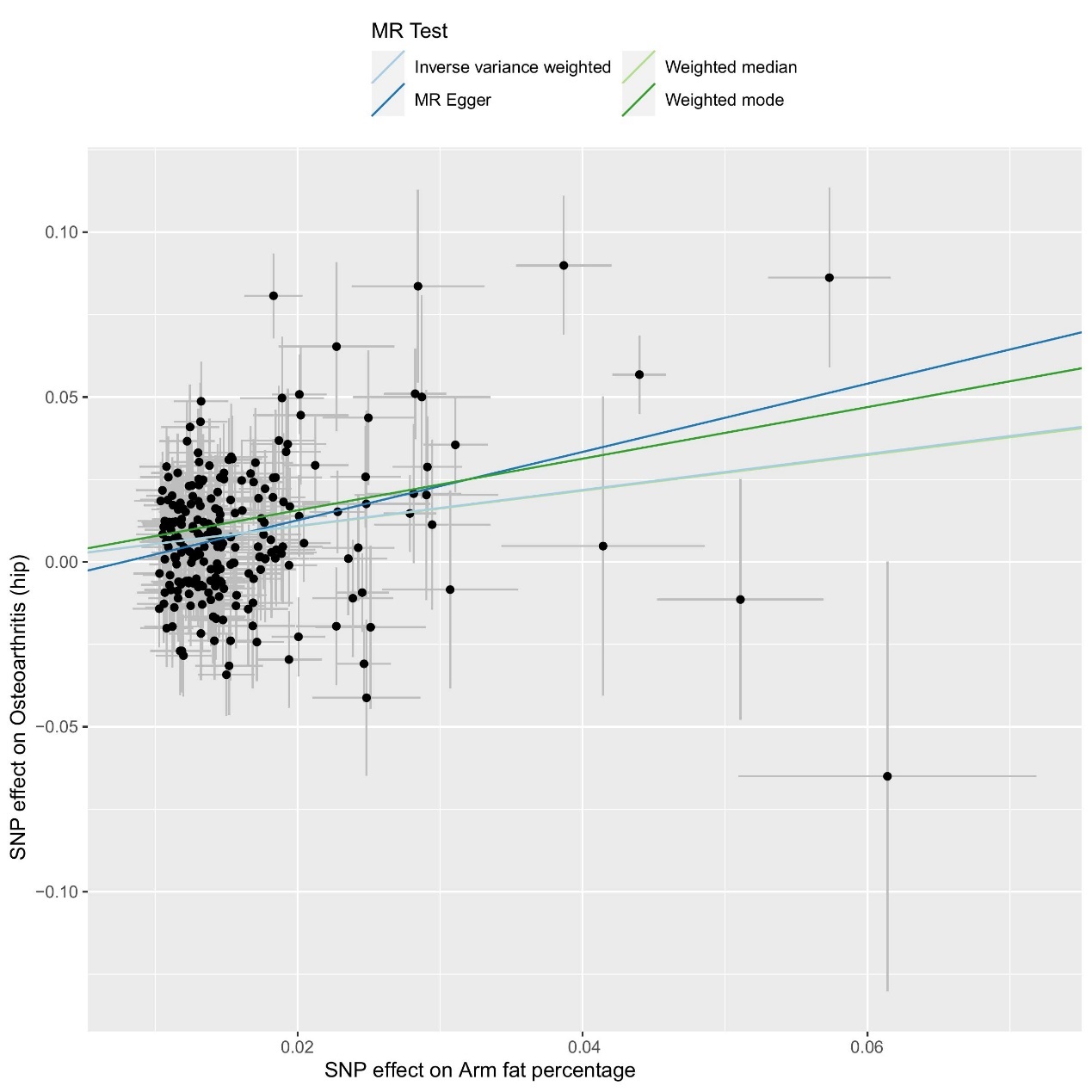


Supplementary Figure 15: Funnel plot of the causal effect of arm fat percentage on knee or hip OA.


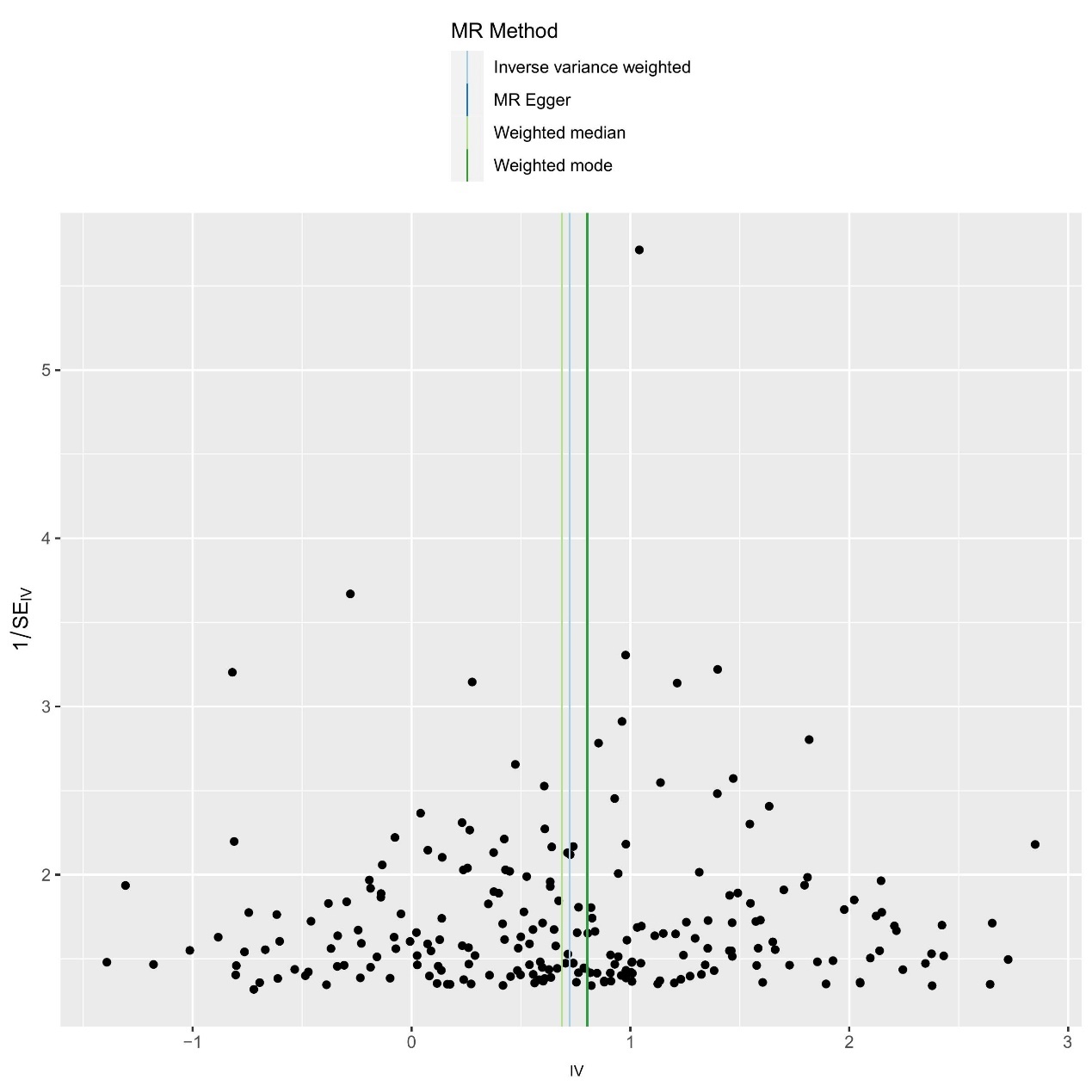


Supplementary Figure 16: Scatter plot of the causal effect of arm fat percentage on knee or hip OA.


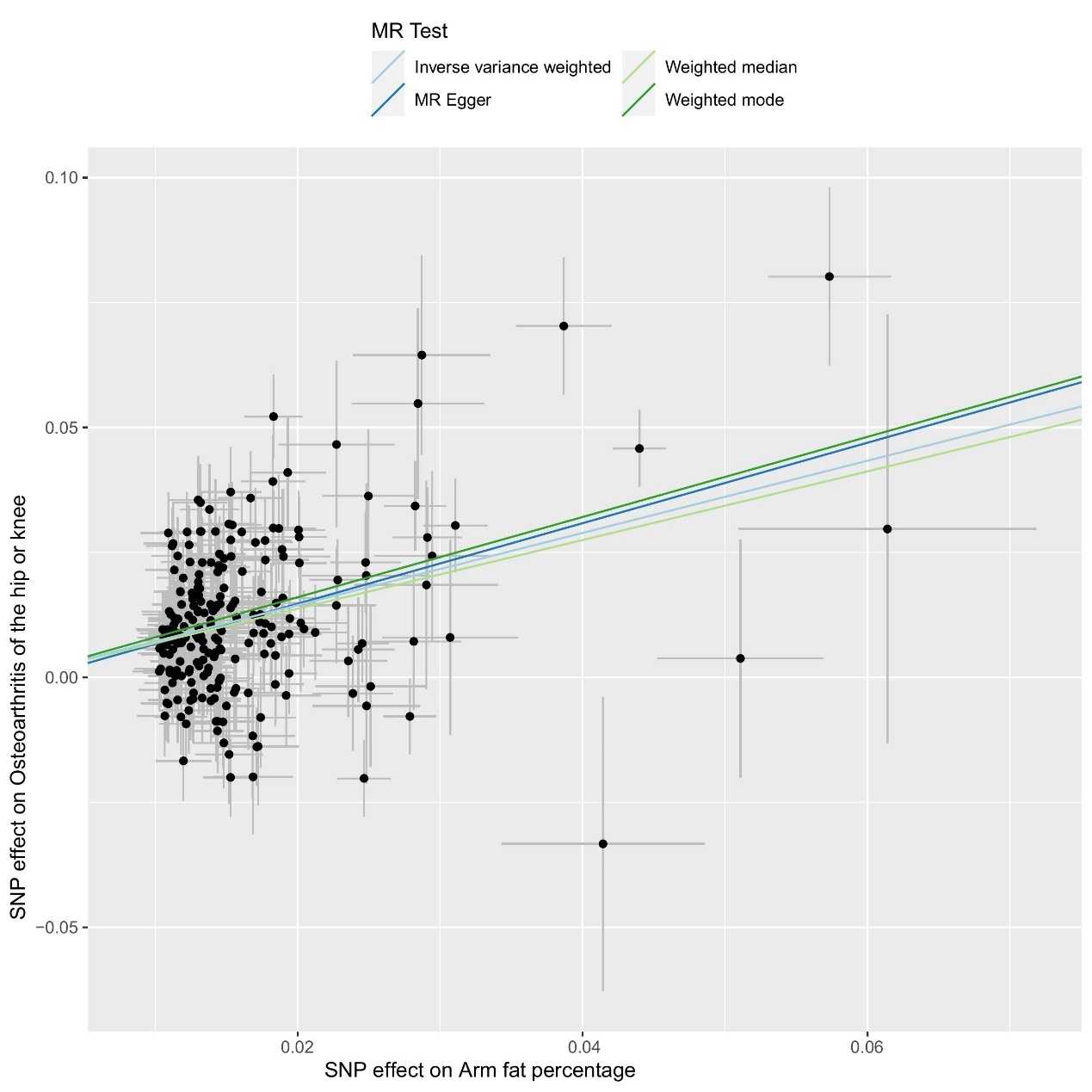


Supplementary Figure 17: Funnel plot of the causal effect of arm fat percentage on knee OA.


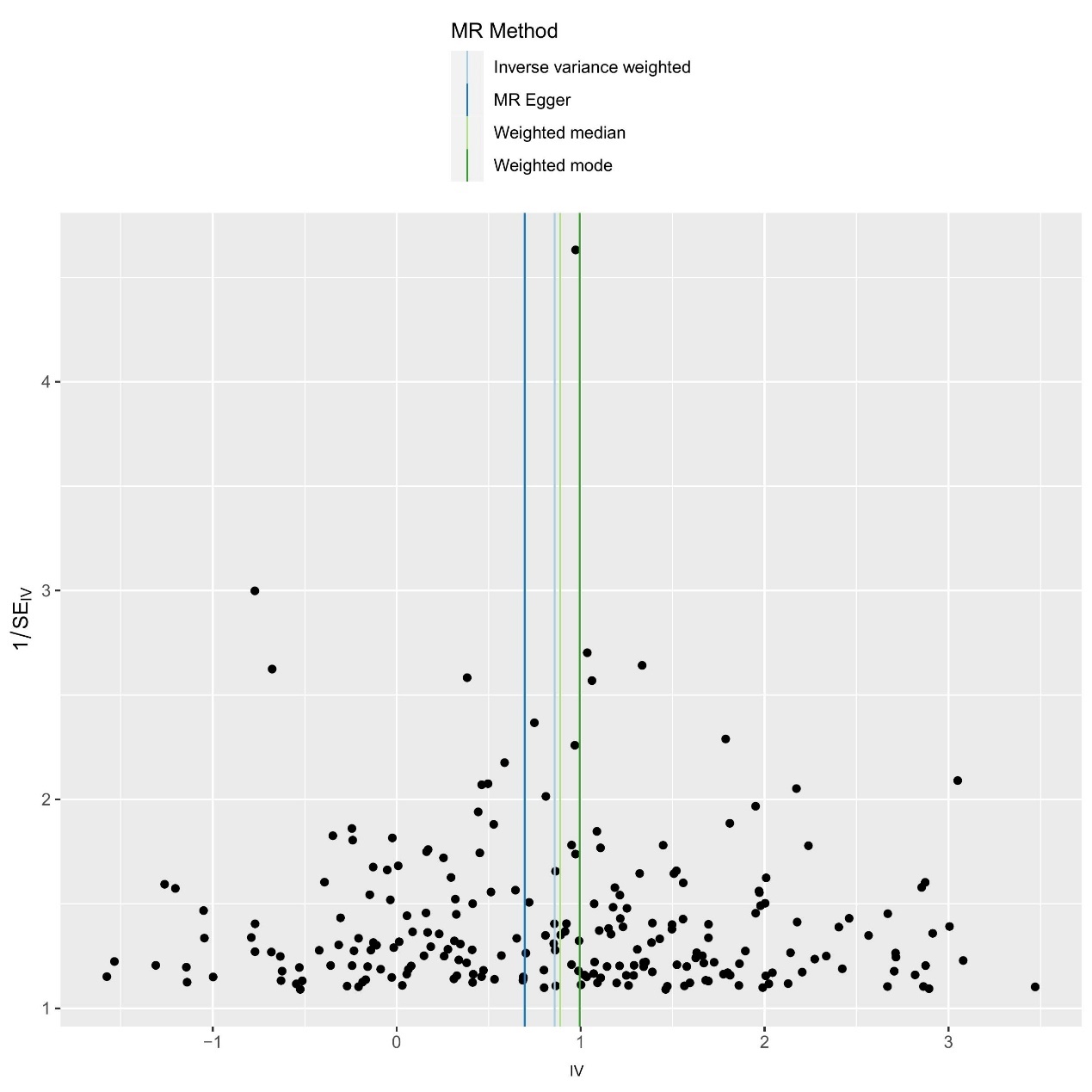


Supplementary Figure 18: Scatter plot of the causal effect of arm fat percentage on knee OA.


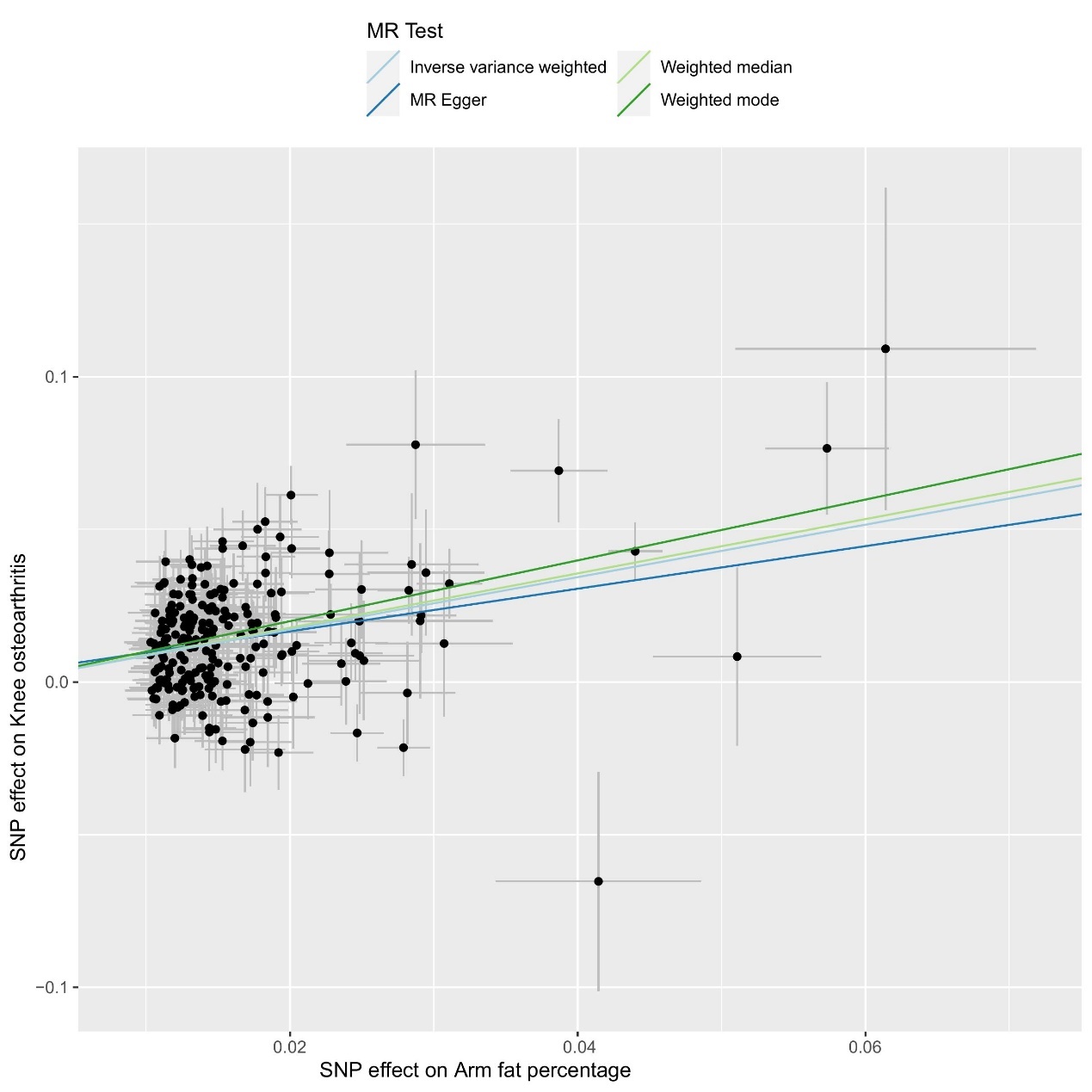


Supplementary Figure 19: Funnel plot of the causal effect of body fat percentage on hip OA.


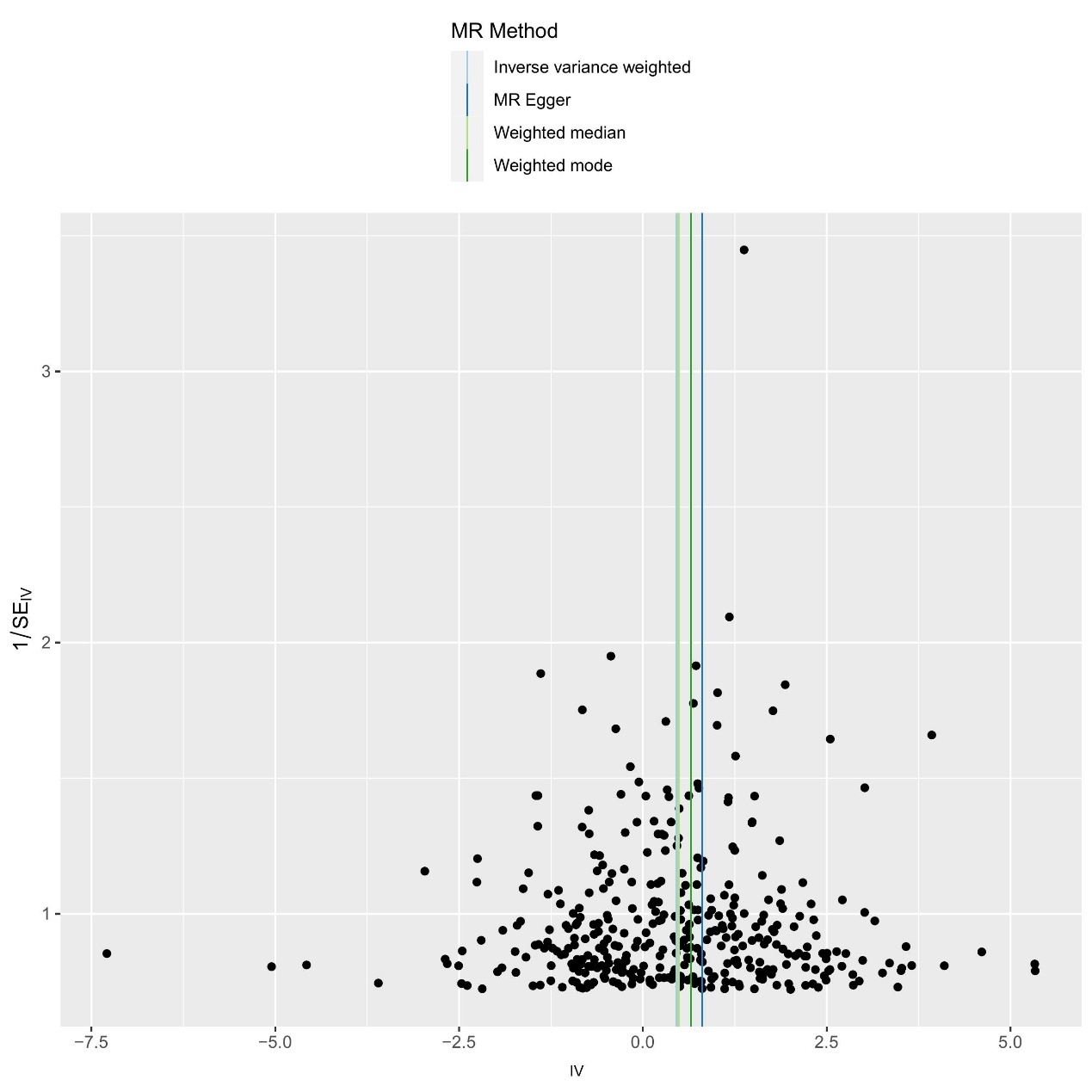


Supplementary Figure 20: Scatter plot of the causal effect of body fat percentage on hip OA.


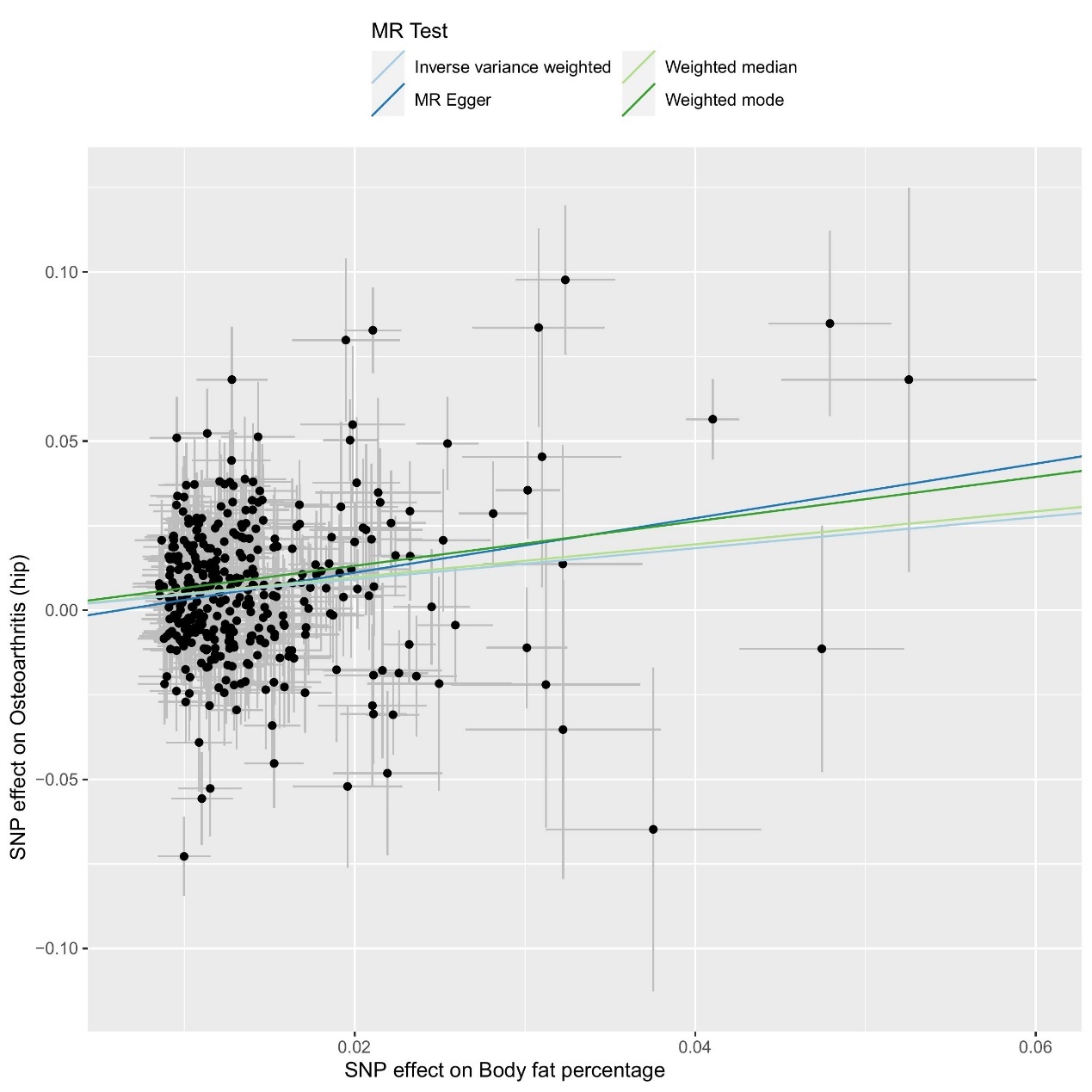


Supplementary Figure 21: Funnel plot of the causal effect of body fat percentage on knee or hip OA.


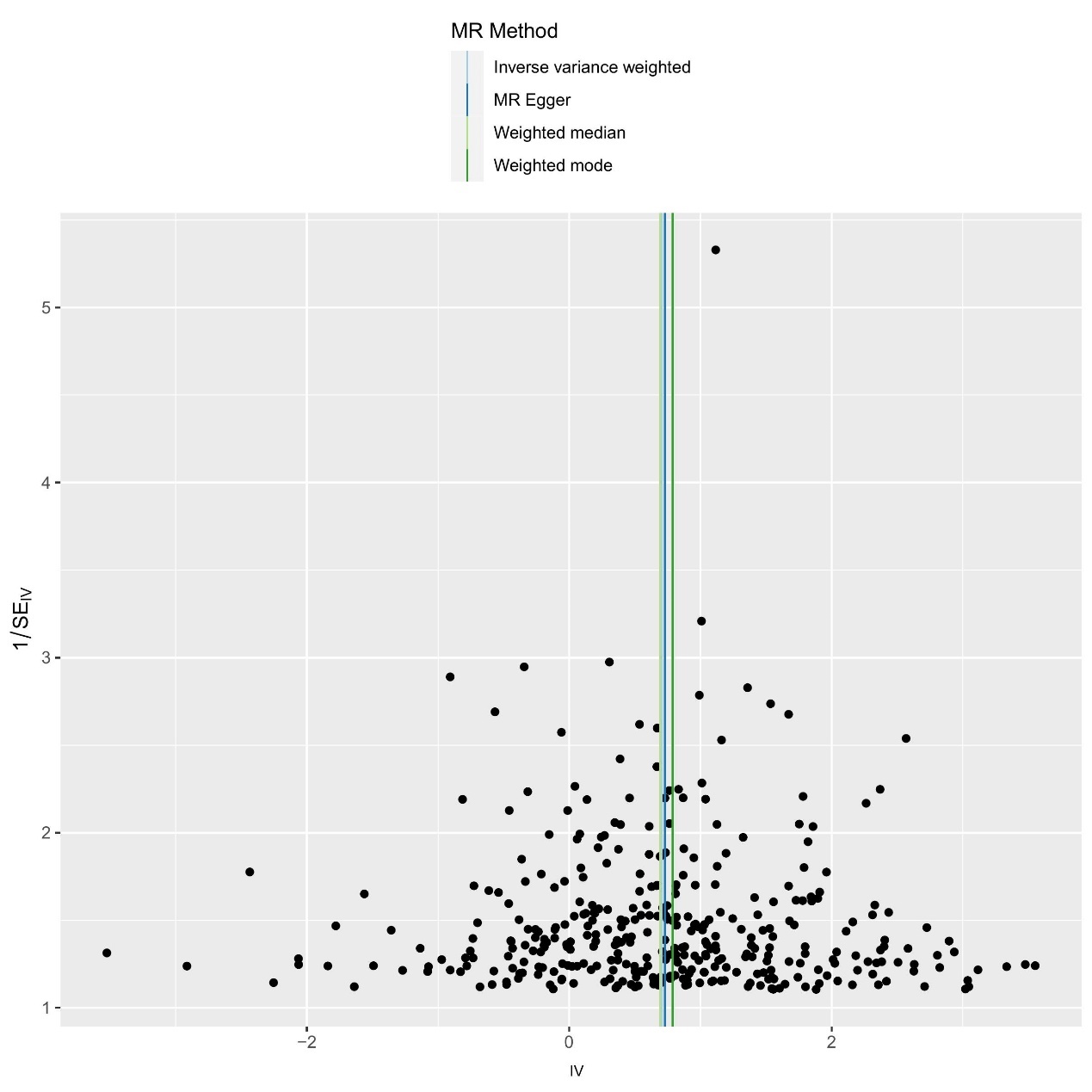


Supplementary Figure 22: Scatter plot of the causal effect of body fat percentage on knee or hip OA.


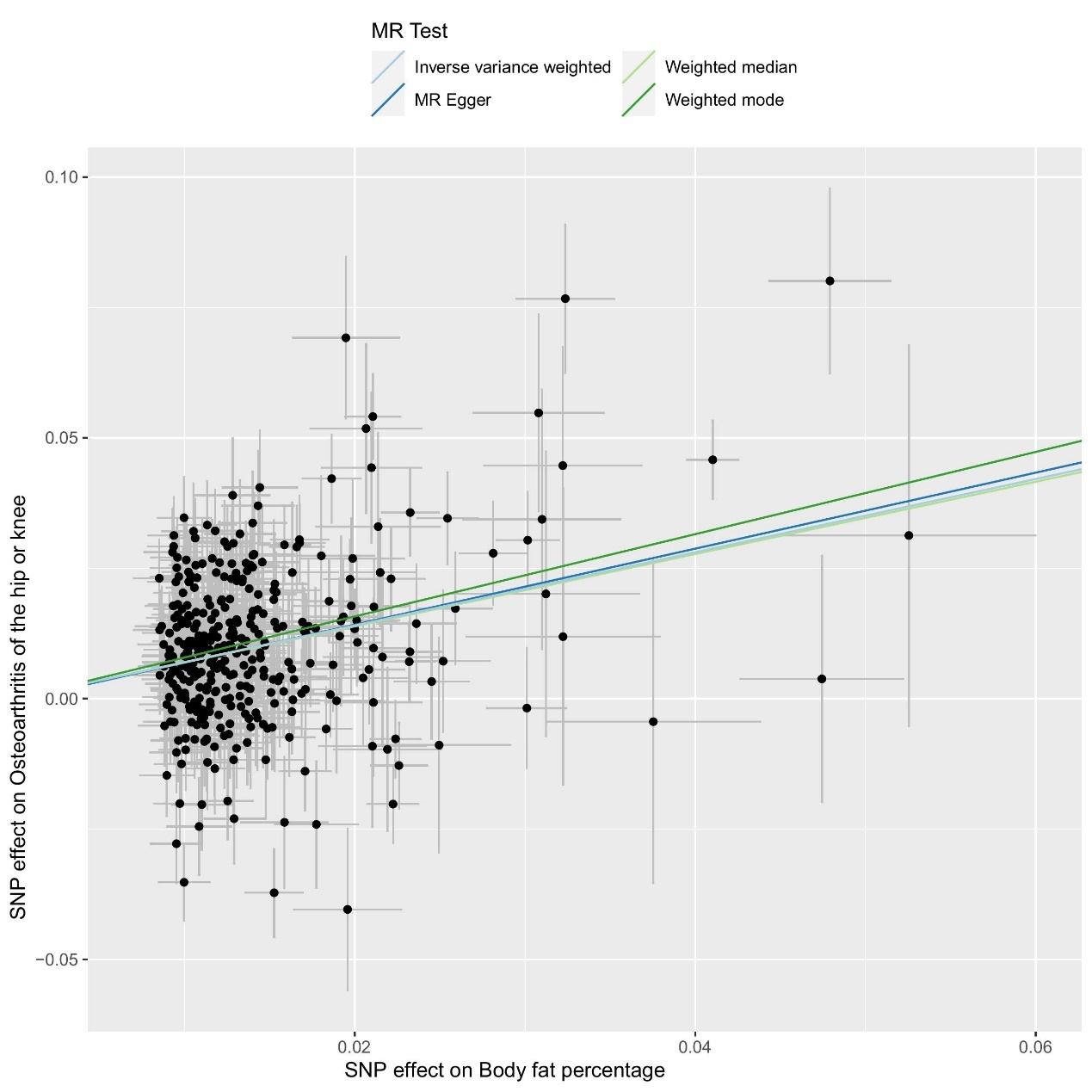


Supplementary Figure 23: Funnel plot of the causal effect of body fat percentage on knee OA.


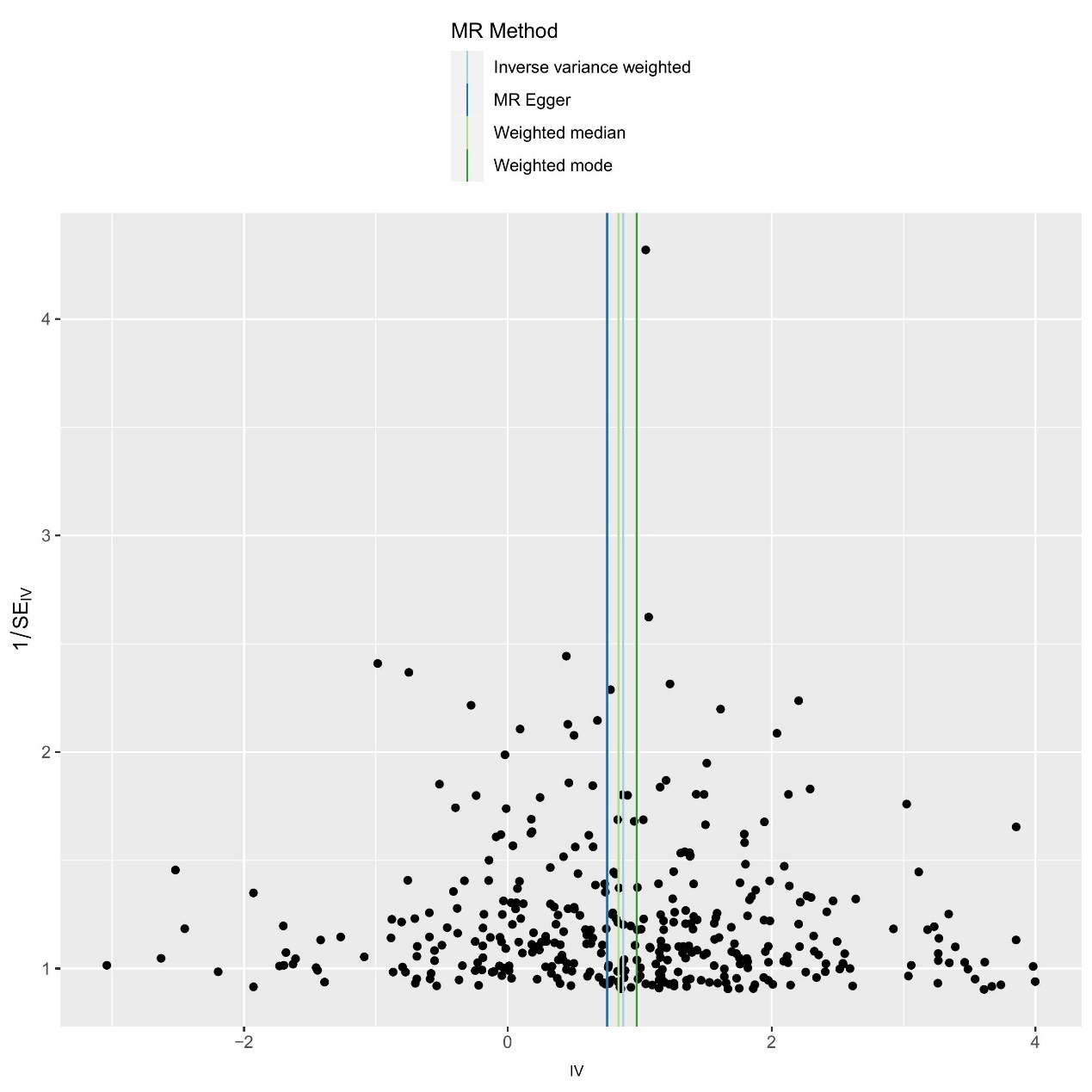


Supplementary Figure 24: Scatter plot of the causal effect of body fat percentage on knee OA.


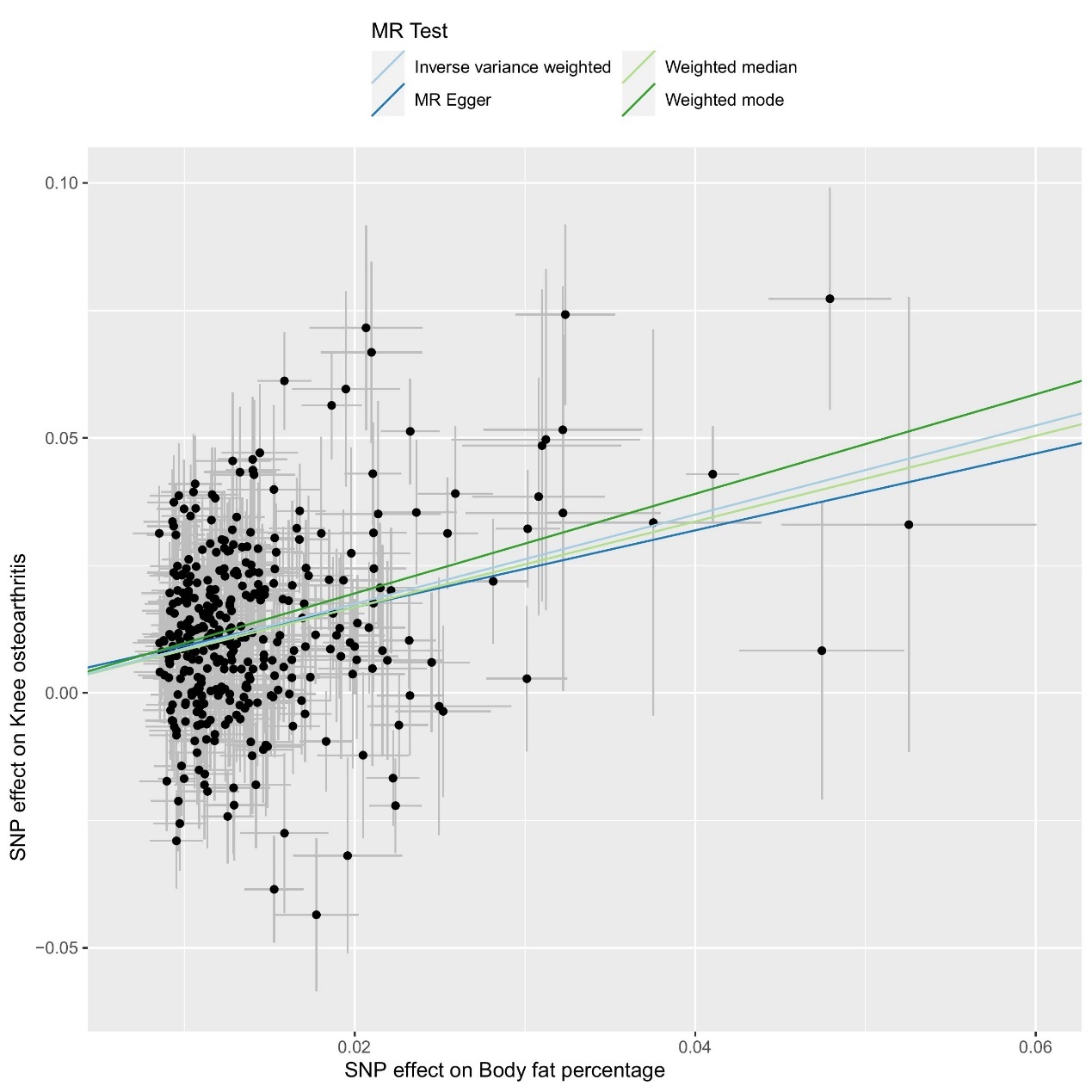


Supplementary Figure 25: Funnel plot of the causal effect of body mass index on hip OA.


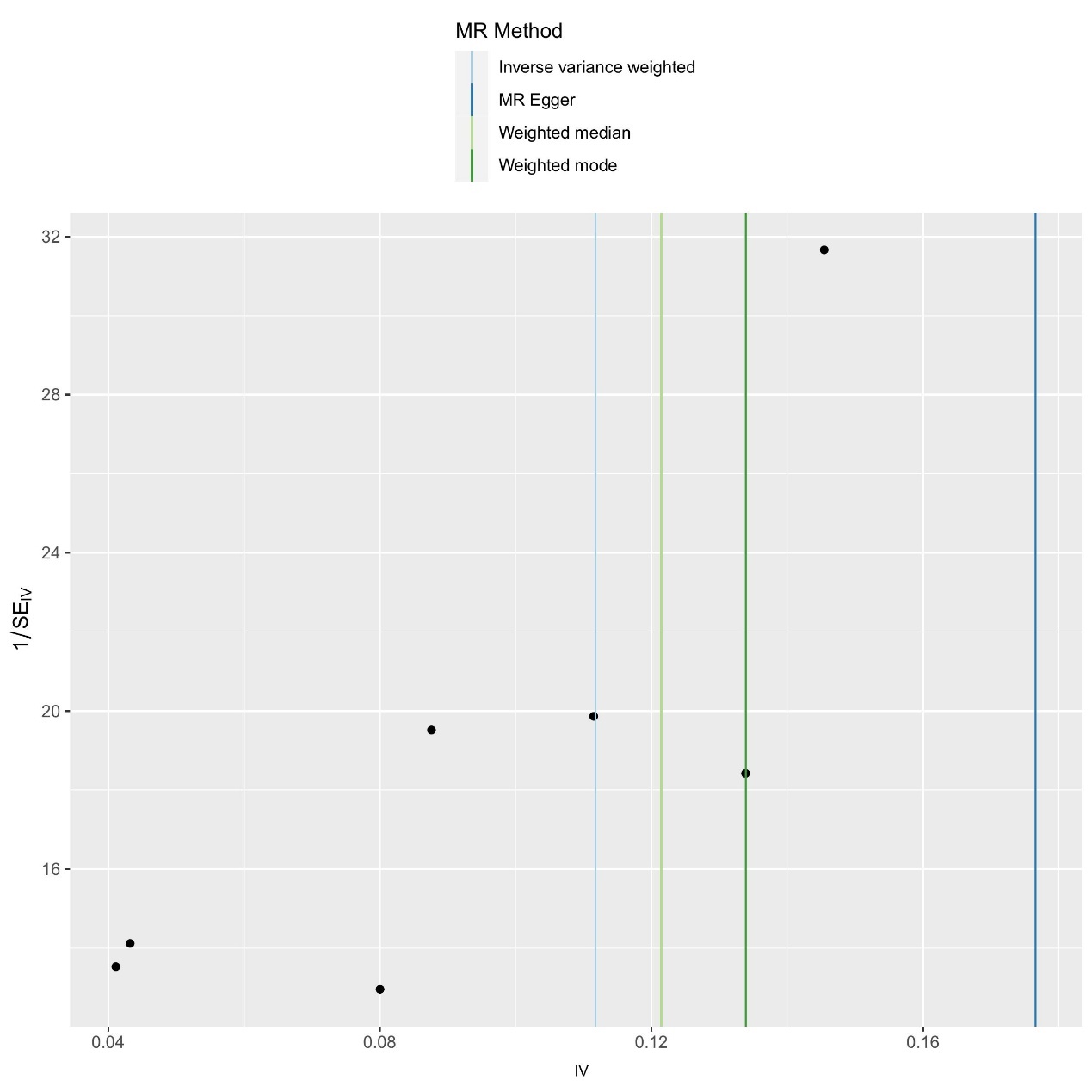


Supplementary Figure 26: Scatter plot of the causal effect of body mass index on hip OA.


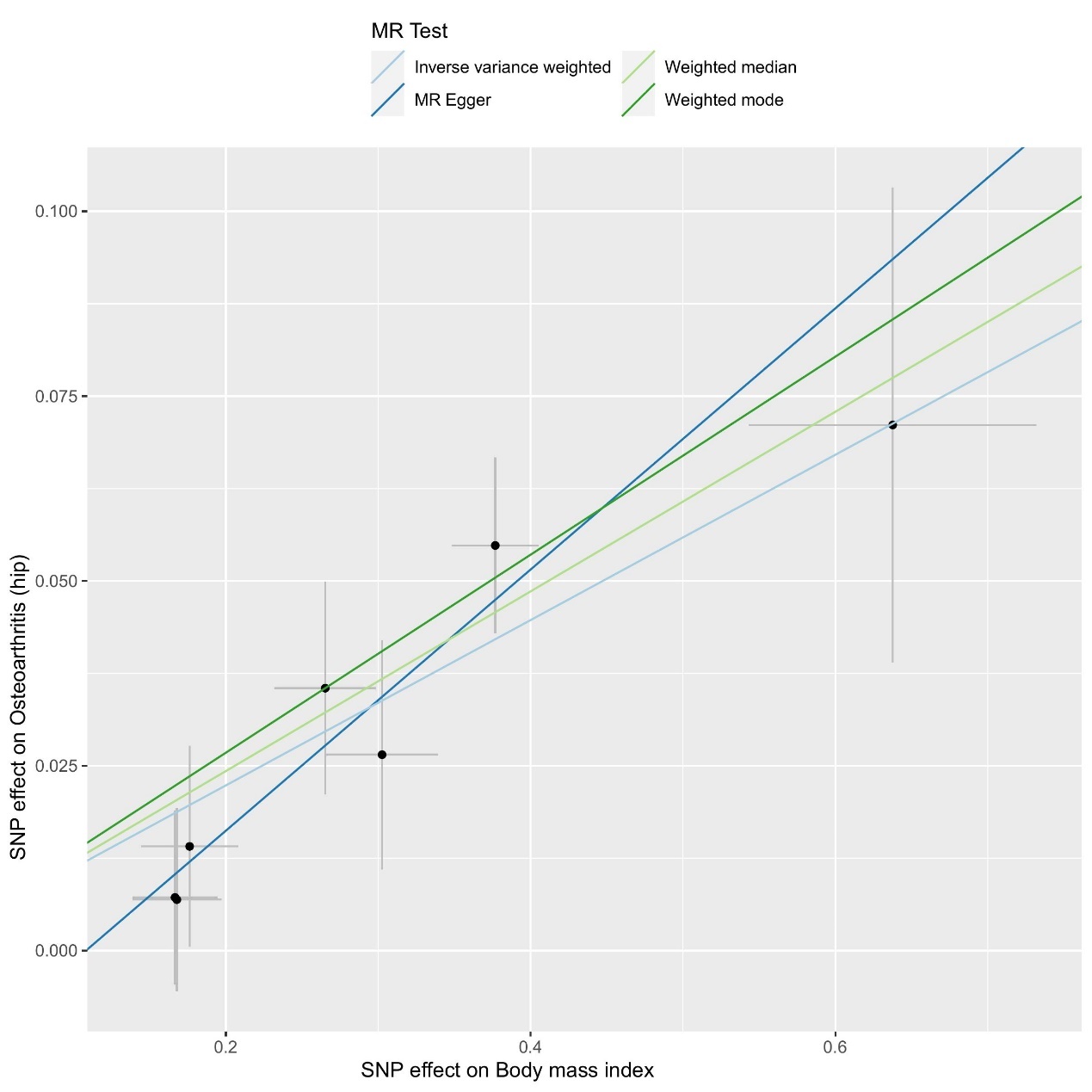


Supplementary Figure 27: Funnel plot of the causal effect of body mass index on knee or hip OA.


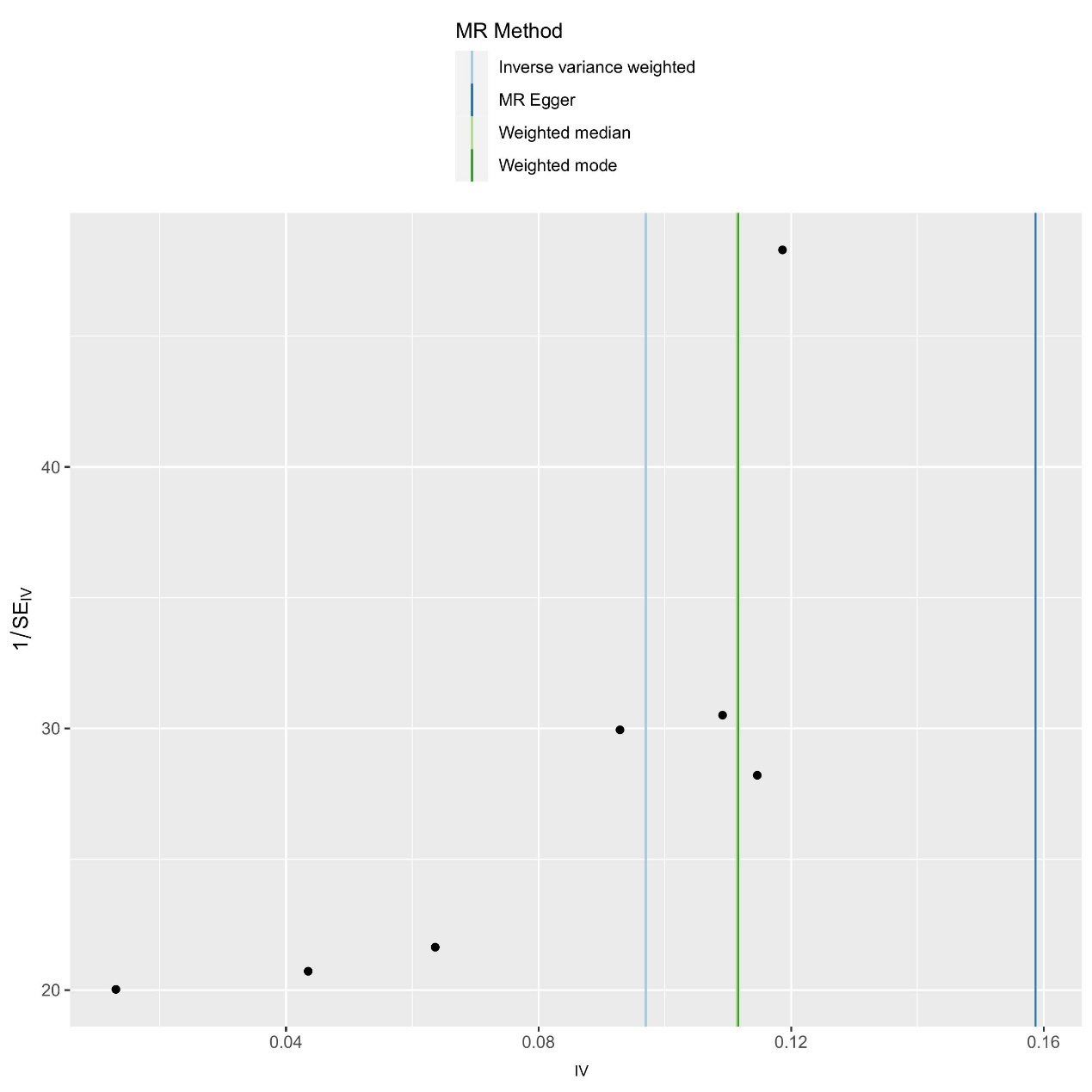


Supplementary Figure 28: Scatter plot of the causal effect of body mass index on knee or hip OA.


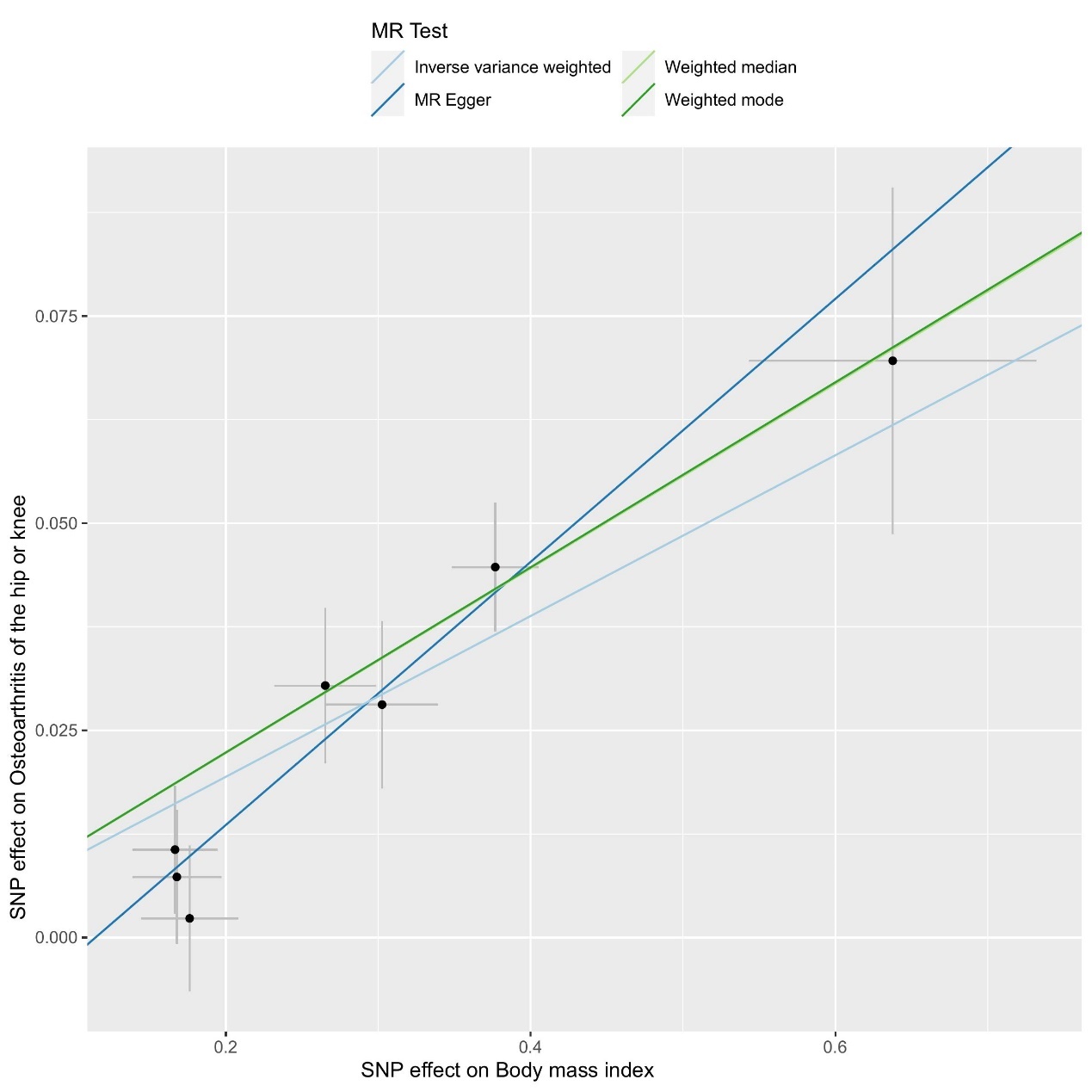


Supplementary Figure 29: Funnel plot of the causal effect of body mass index on knee OA.


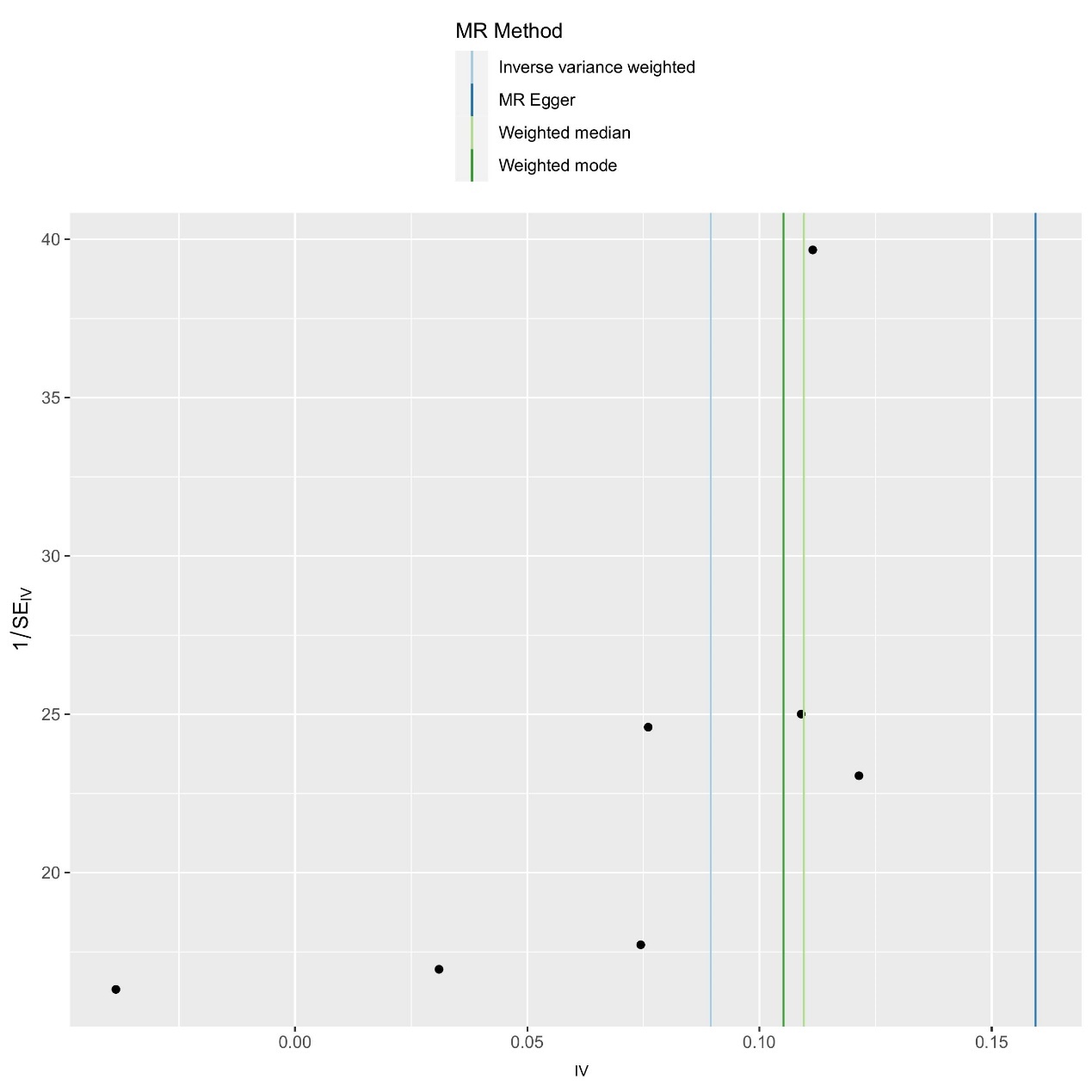


Supplementary Figure 30: Scatter plot of the causal effect of body mass index on knee OA.


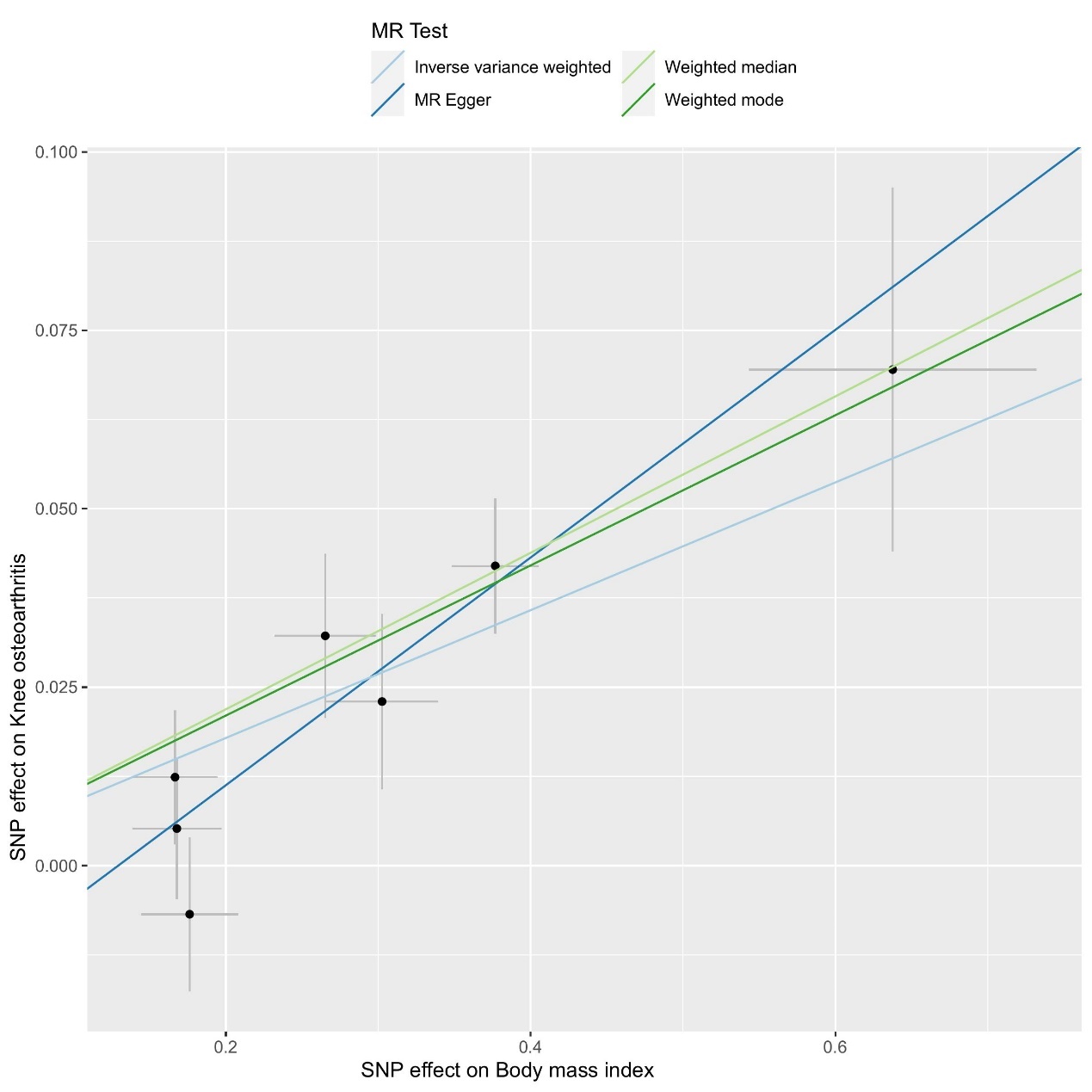


Supplementary Figure 31: Funnel plot of the causal effect of hip circumference on hip OA.


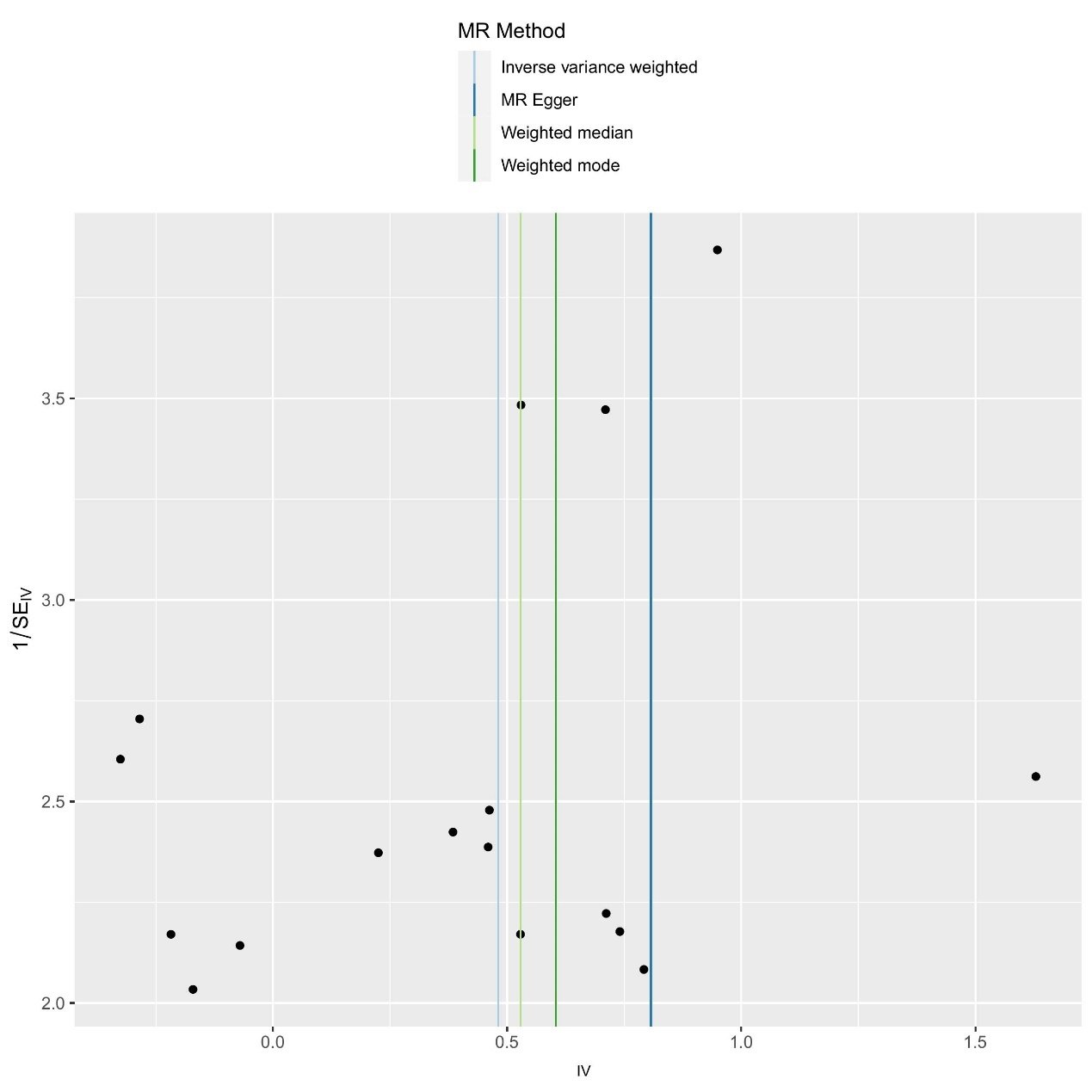


Supplementary Figure 32: Scatter plot of the causal effect of hip circumference on hip OA.


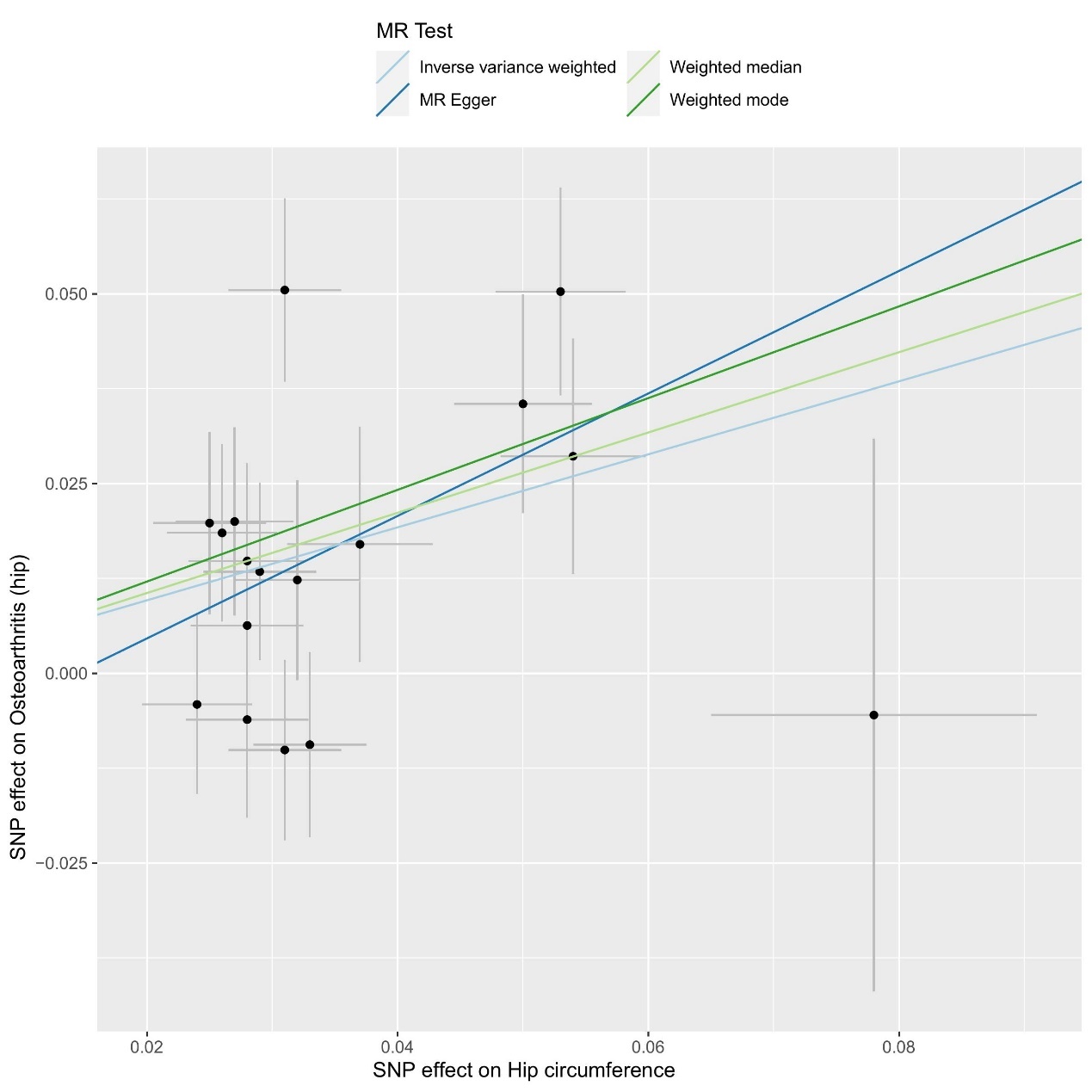


Supplementary Figure 33: Funnel plot of the causal effect of hip circumference on knee or hip OA.


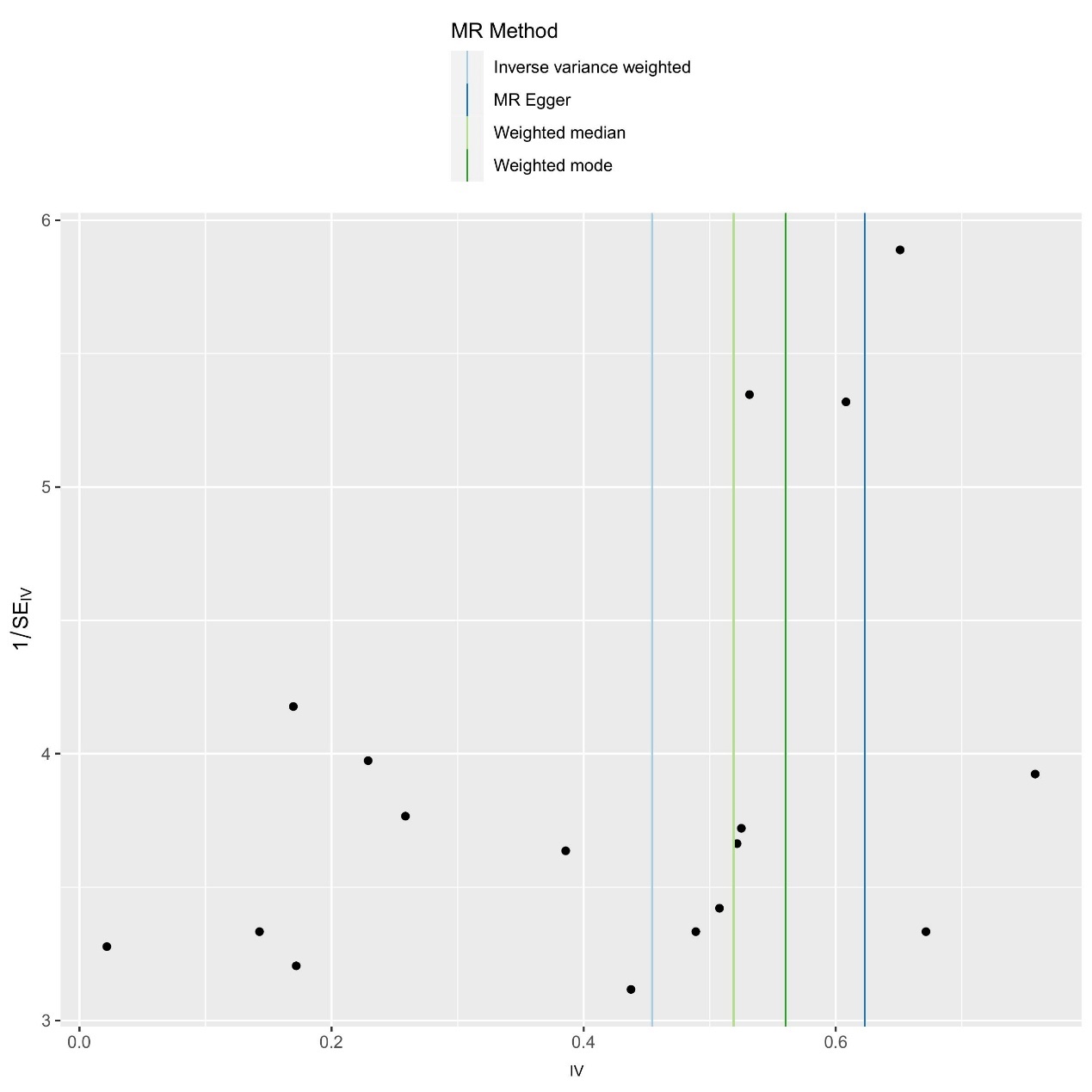


Supplementary Figure 34: Scatter plot of the causal effect of hip circumference on knee or hip OA.


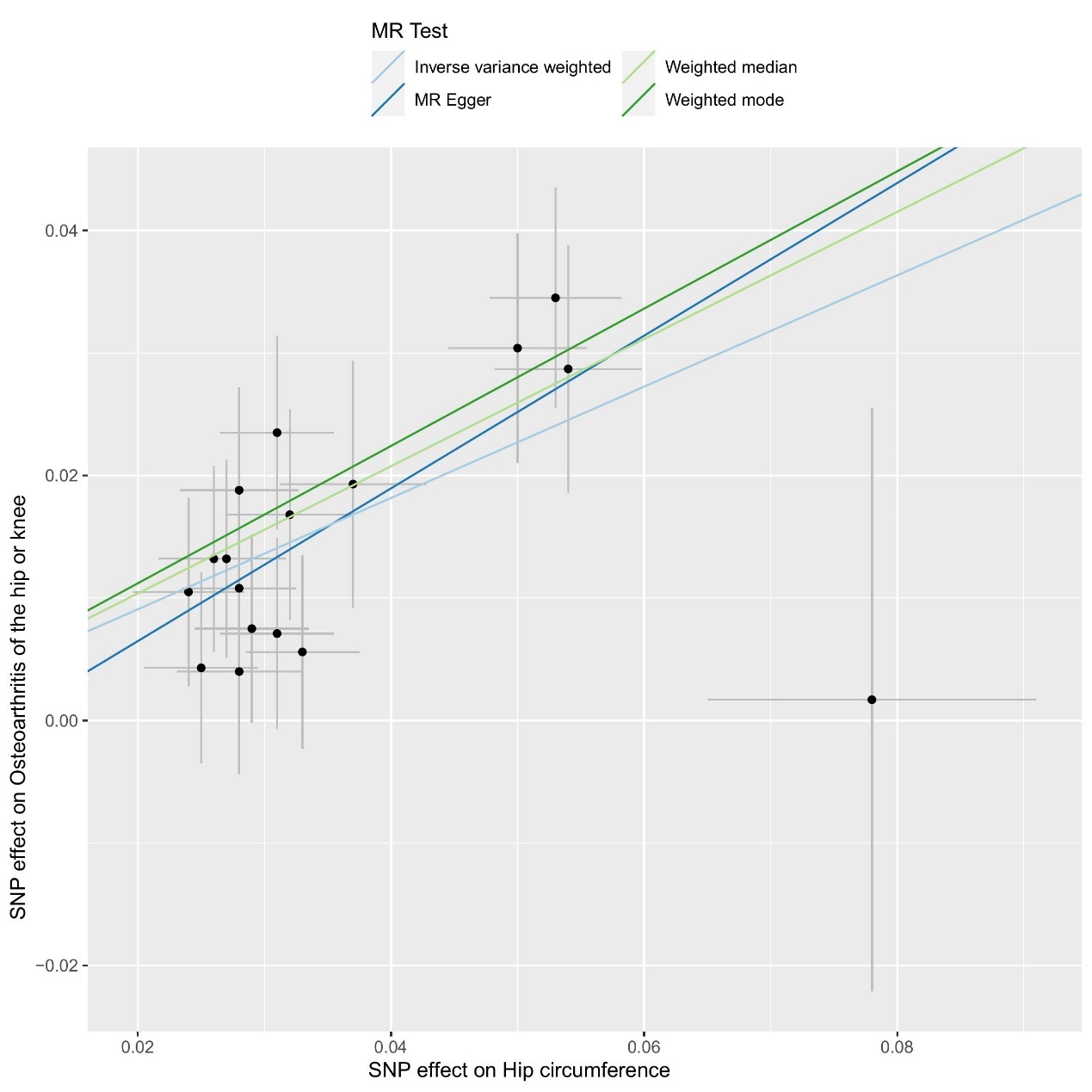


Supplementary Figure 35: Funnel plot of the causal effect of hip circumference on knee OA.


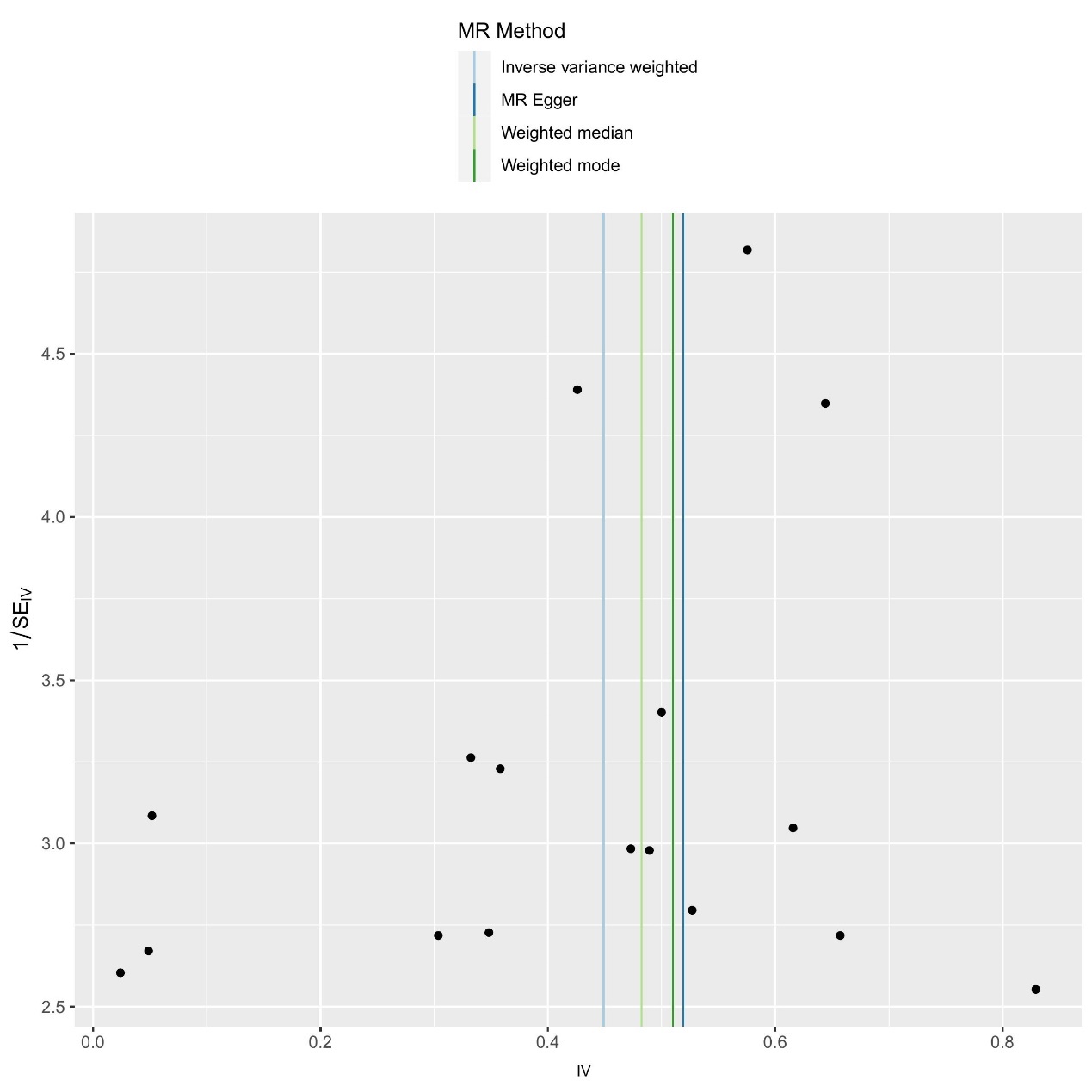


Supplementary Figure 36: Scatter plot of the causal effect of hip circumference on knee OA.


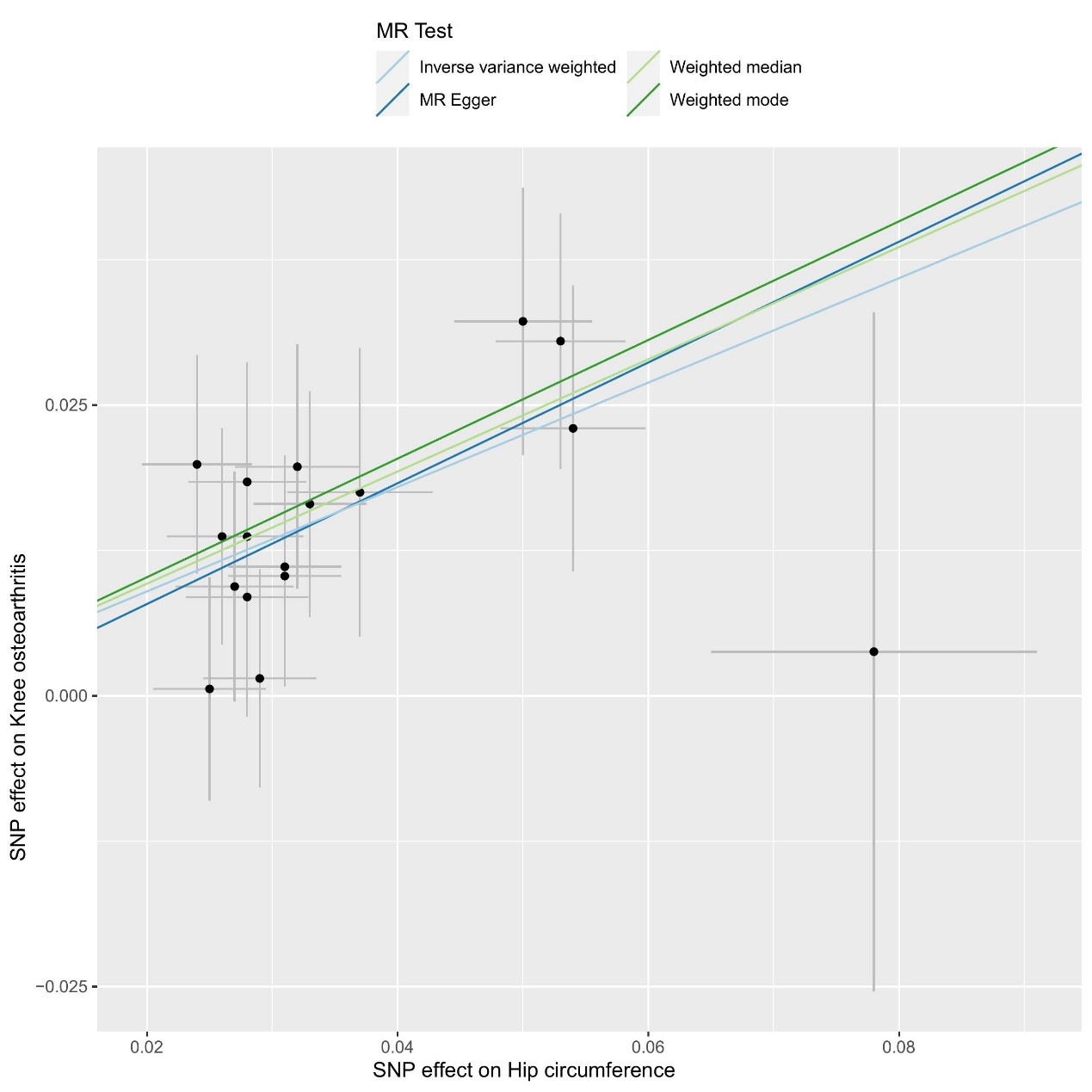


Supplementary Figure 37: Funnel plot of the causal effect of leg fat-free mass on hip OA.


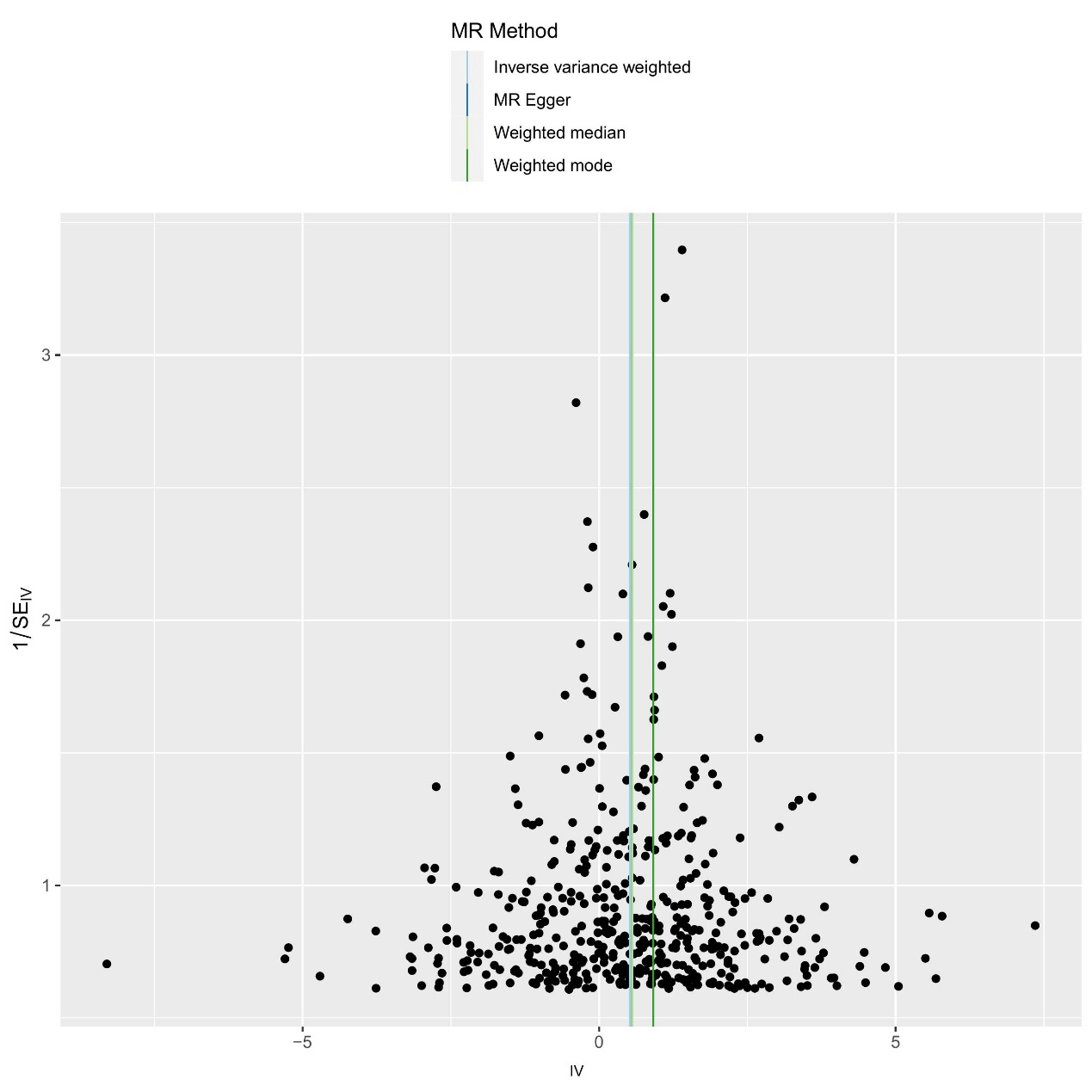


Supplementary Figure 38: Scatter Funnel plot of the causal effect of leg fat-free mass on hip OA.


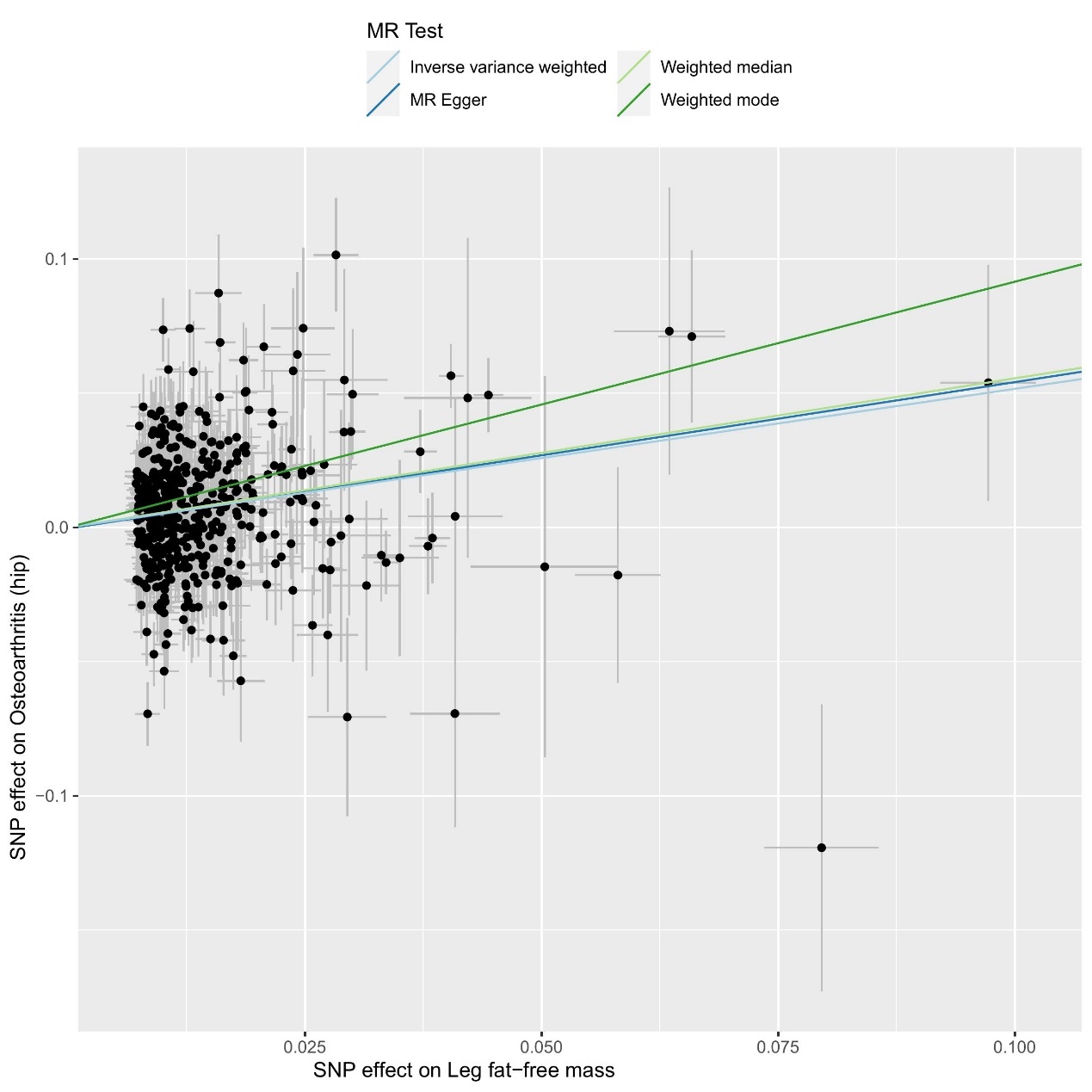


Supplementary Figure 39: Funnel plot of the causal effect of leg fat-free mass on knee or hip OA.


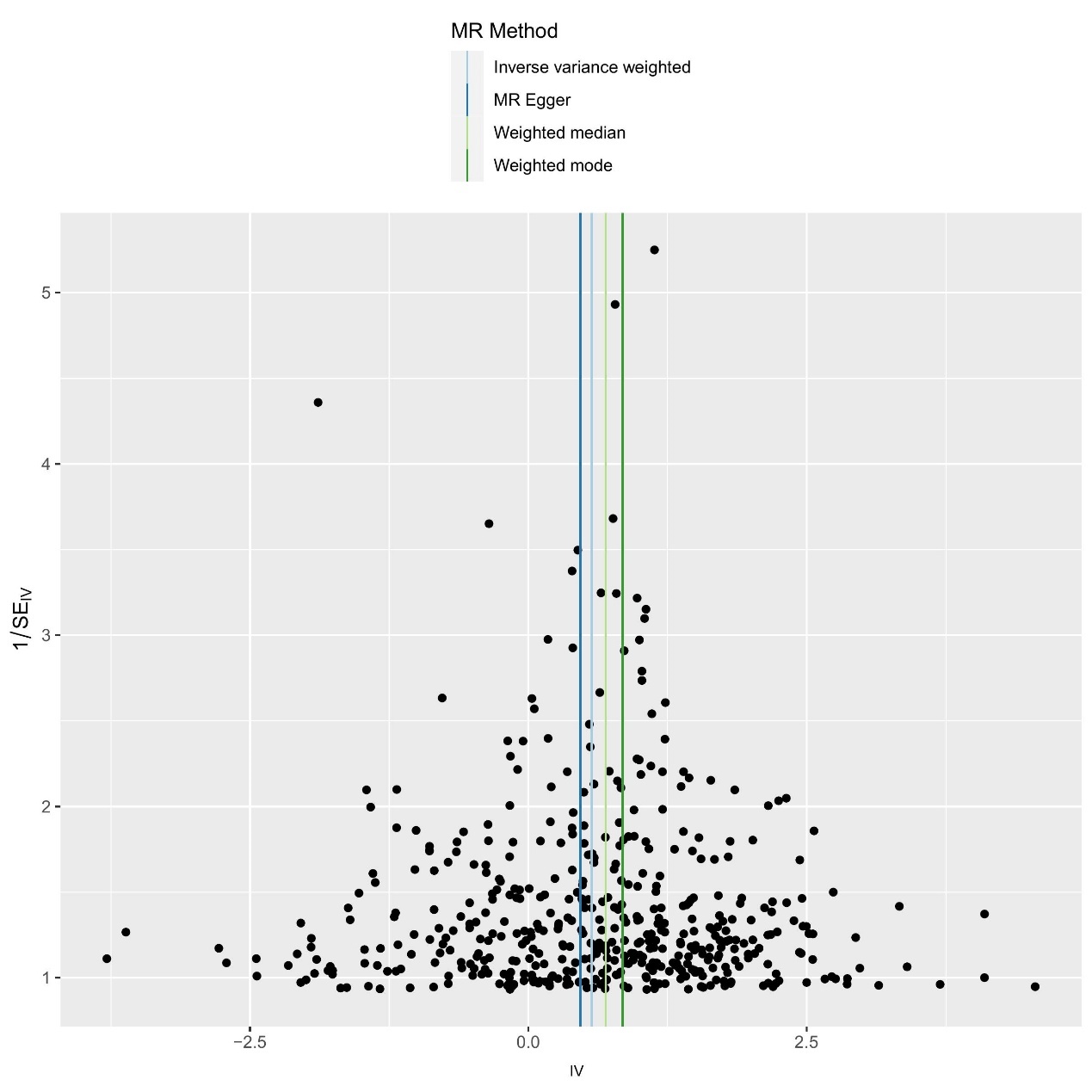


Supplementary Figure 40: Scatter plot of the causal effect of leg fat-free mass on knee or hip OA.


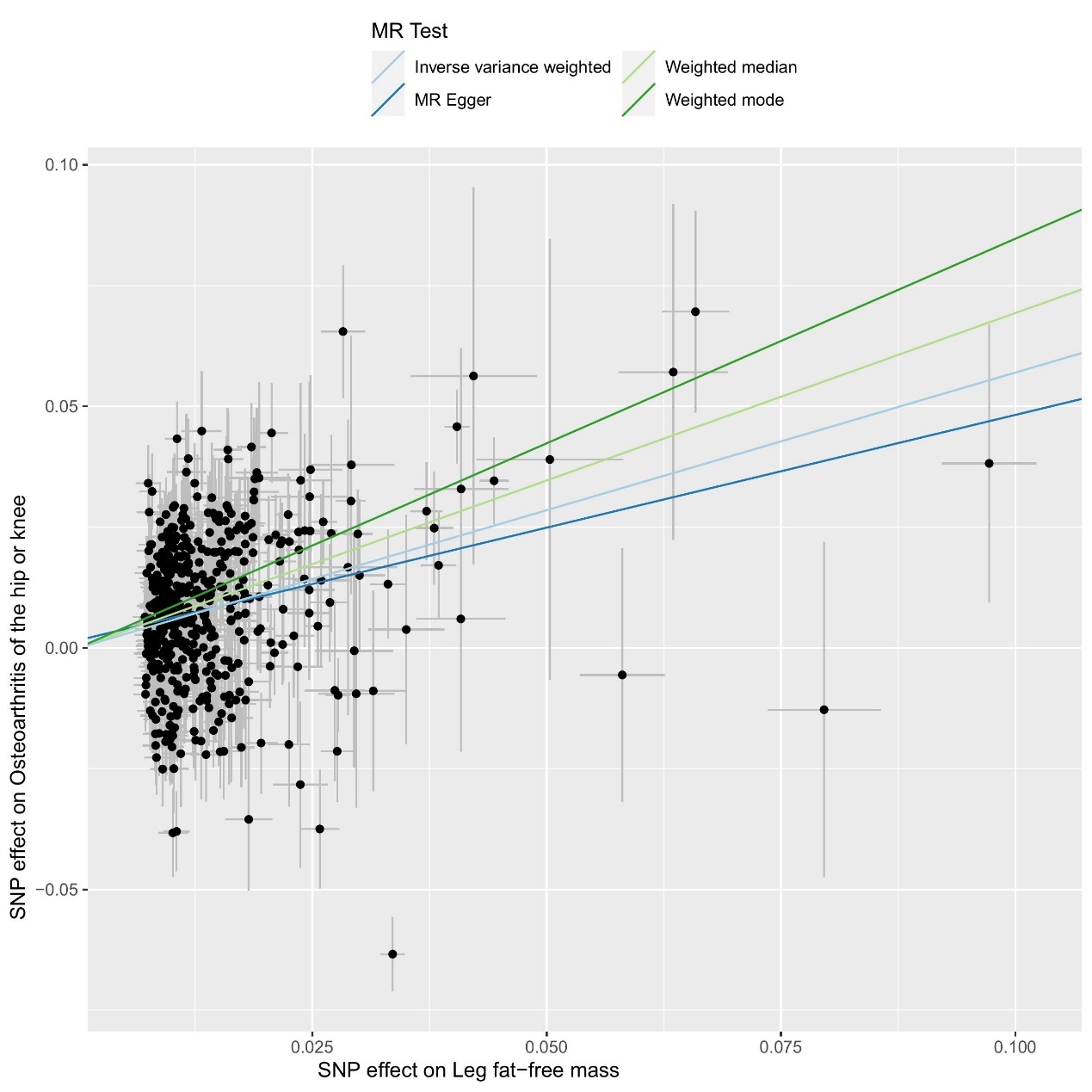


Supplementary Figure 41: Funnel plot of the causal effect of leg fat-free mass on knee OA.


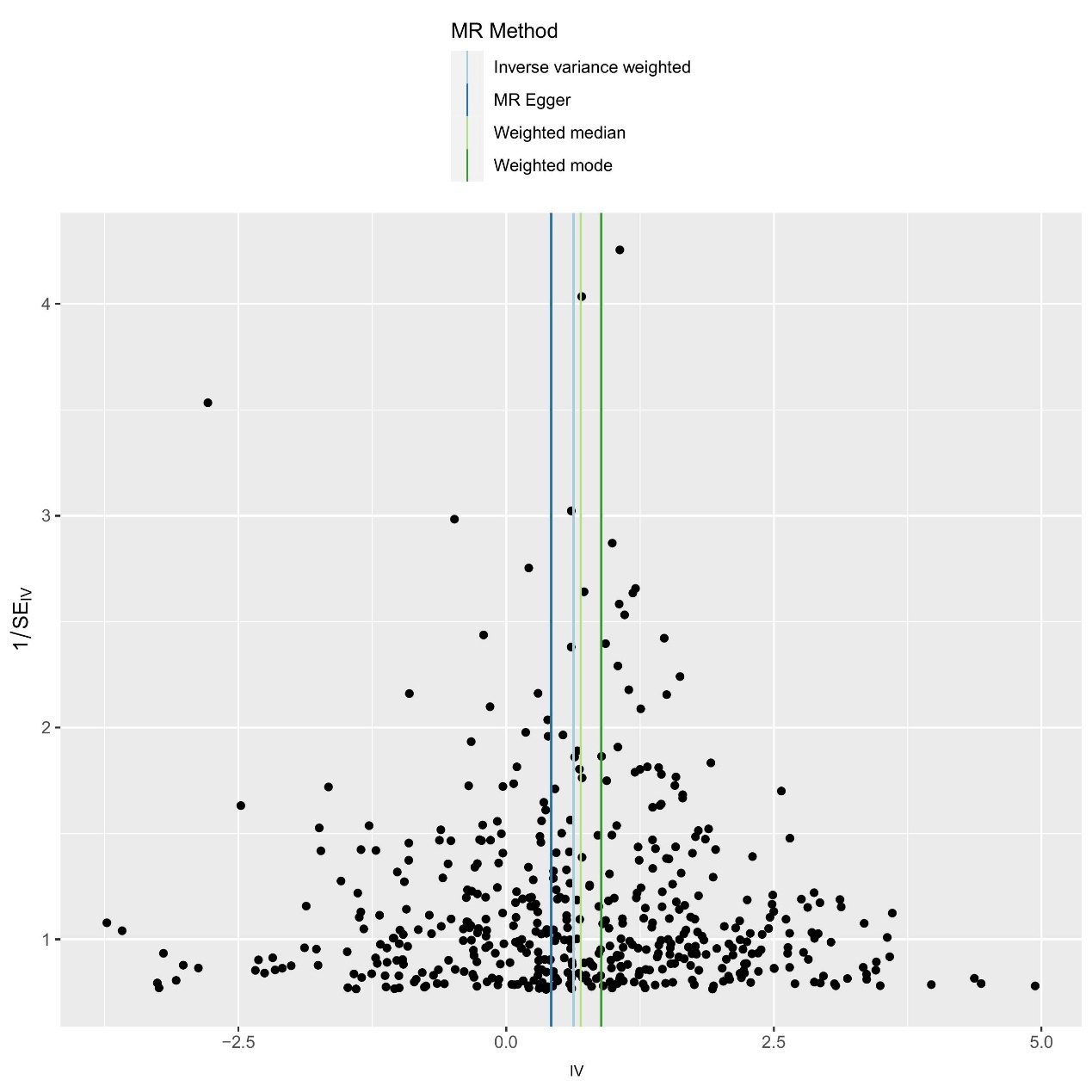


Supplementary Figure 42: Scatter plot of the causal effect of leg fat-free mass on knee OA.


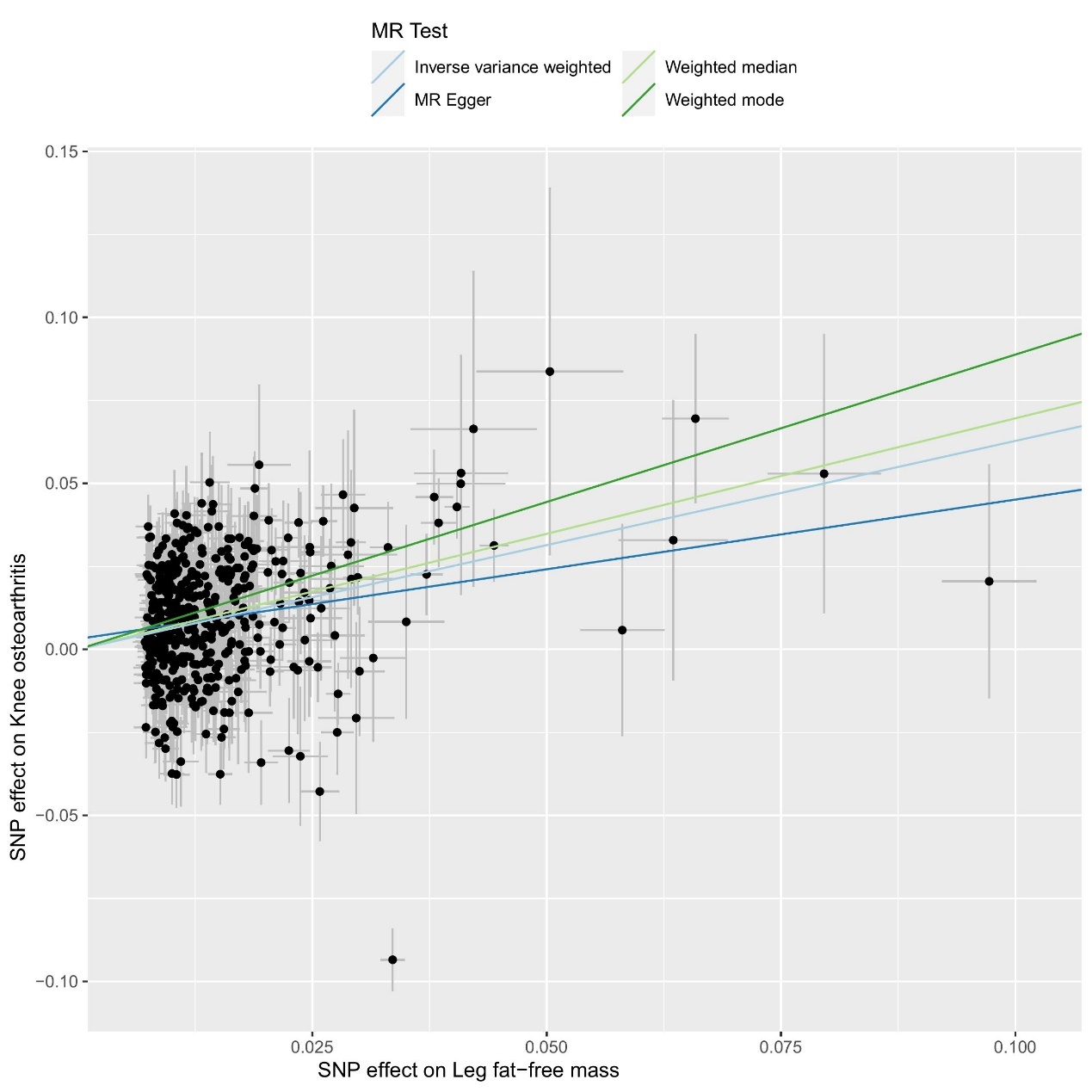


Supplementary Figure 43: Funnel plot of the causal effect of leg fat mass on hip OA.


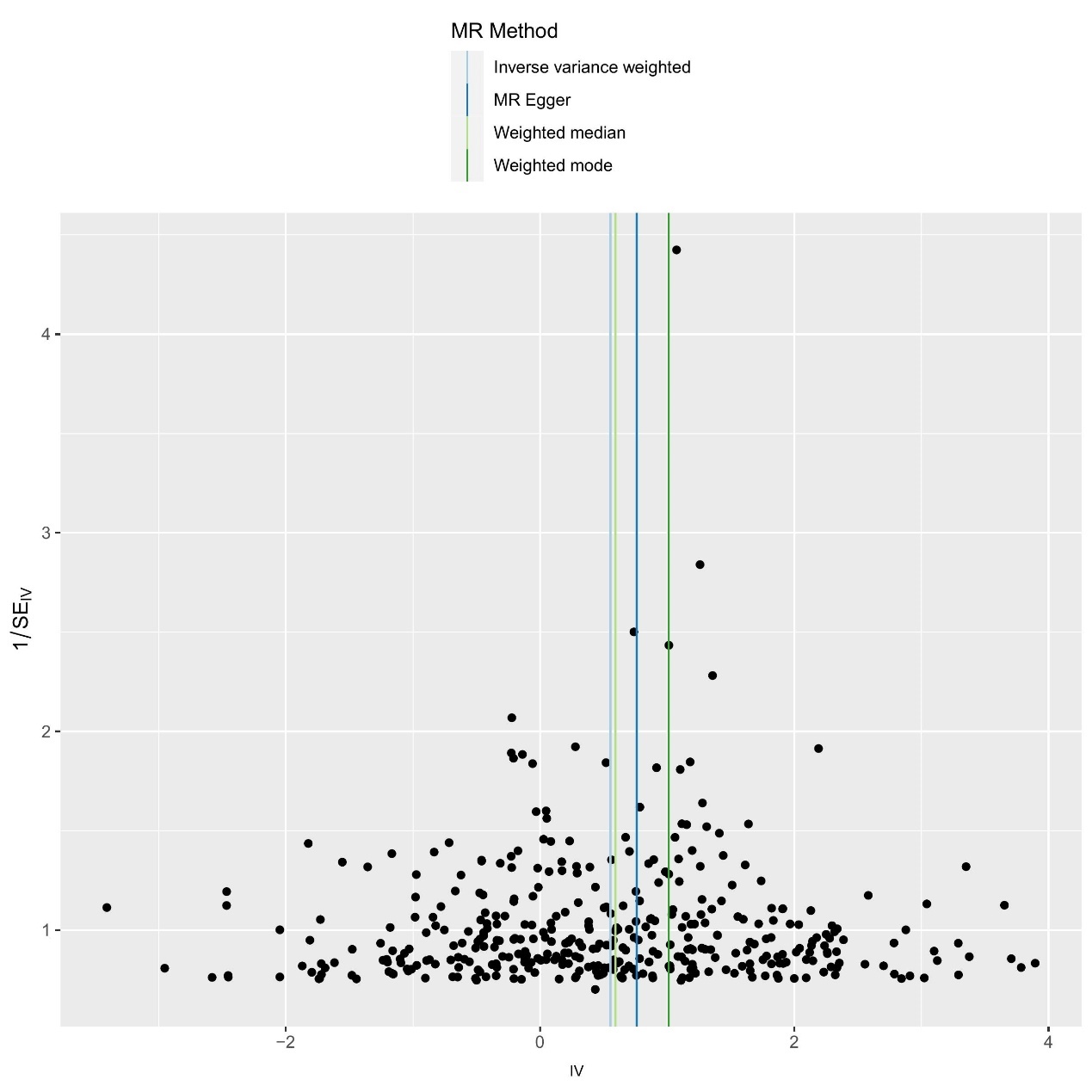


Supplementary Figure 44: Scatter plot of the causal effect of leg fat mass on hip OA.


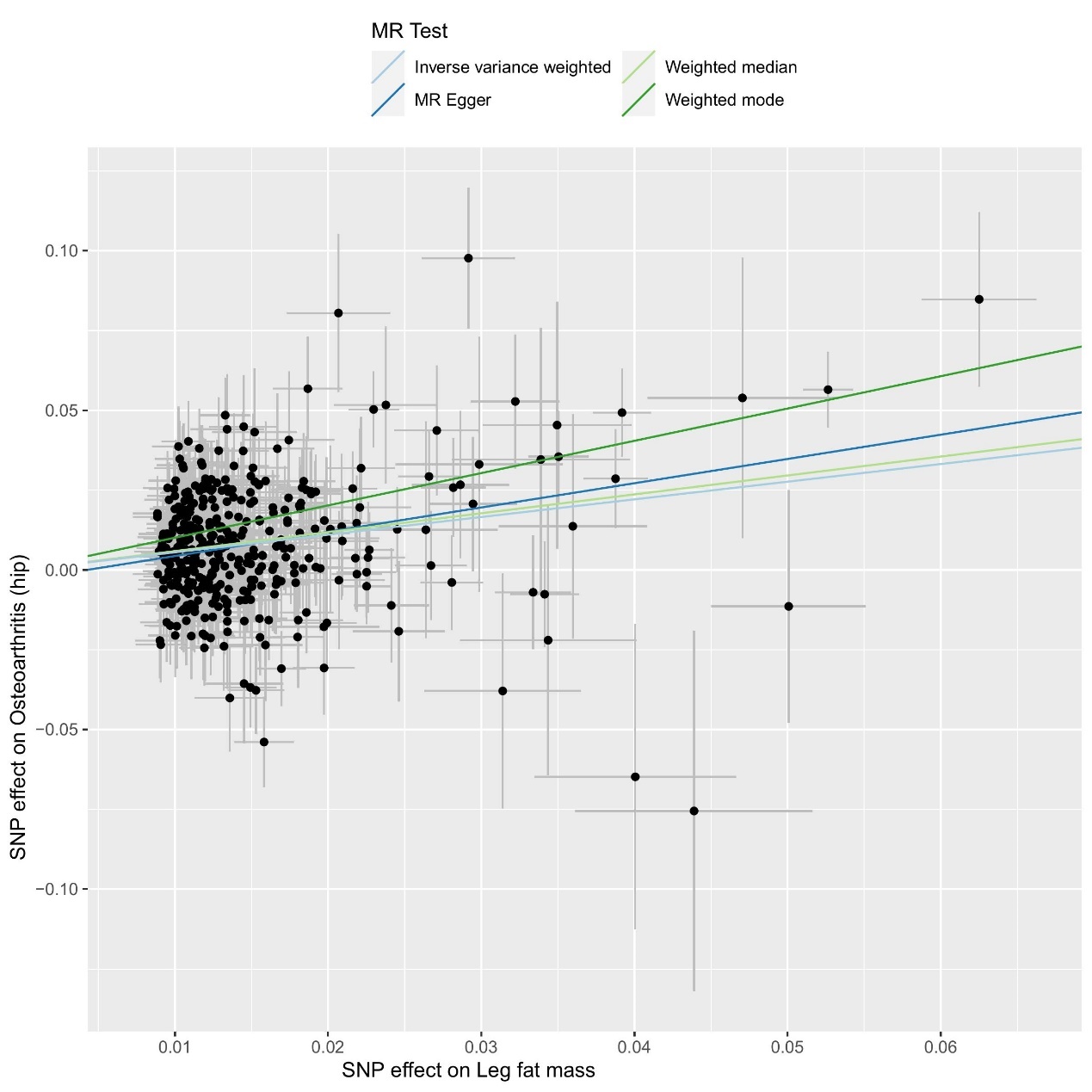


Supplementary Figure 45: Funnel plot of the causal effect of leg fat mass on knee or hip OA.


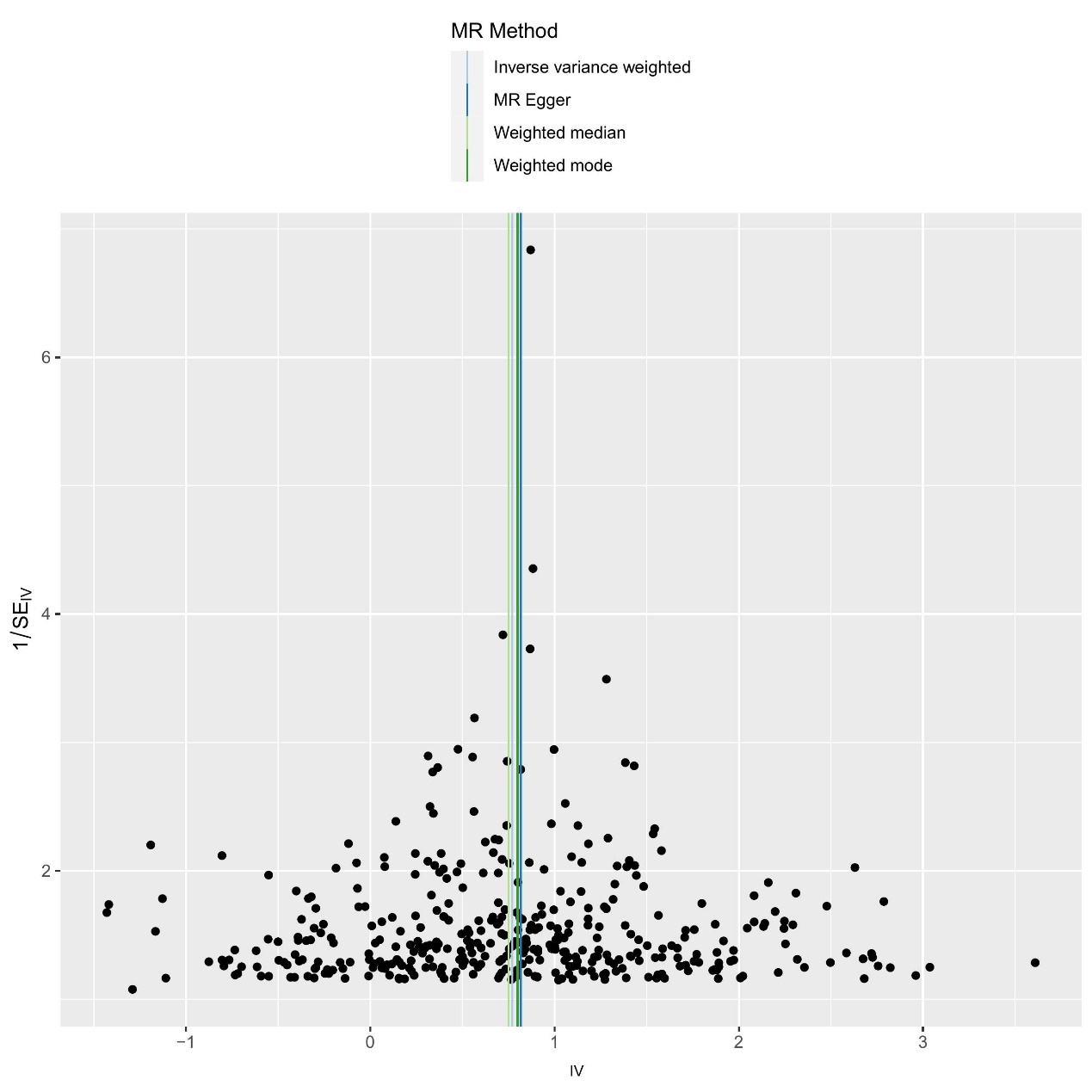


Supplementary Figure 46: Scatter plot of the causal effect of leg fat mass on knee or hip OA.


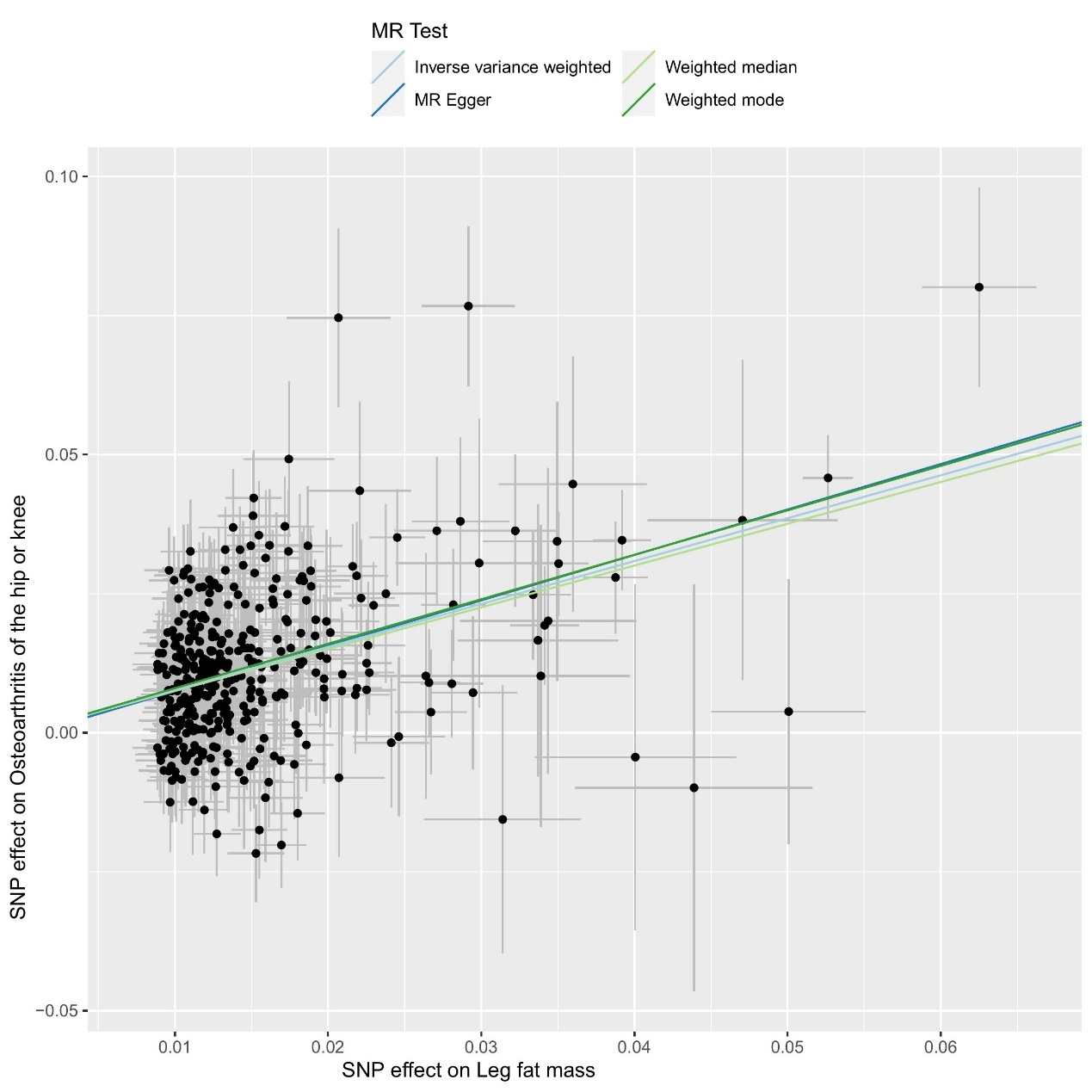


Supplementary Figure 47: Funnel plot of the causal effect of leg fat mass on knee OA.


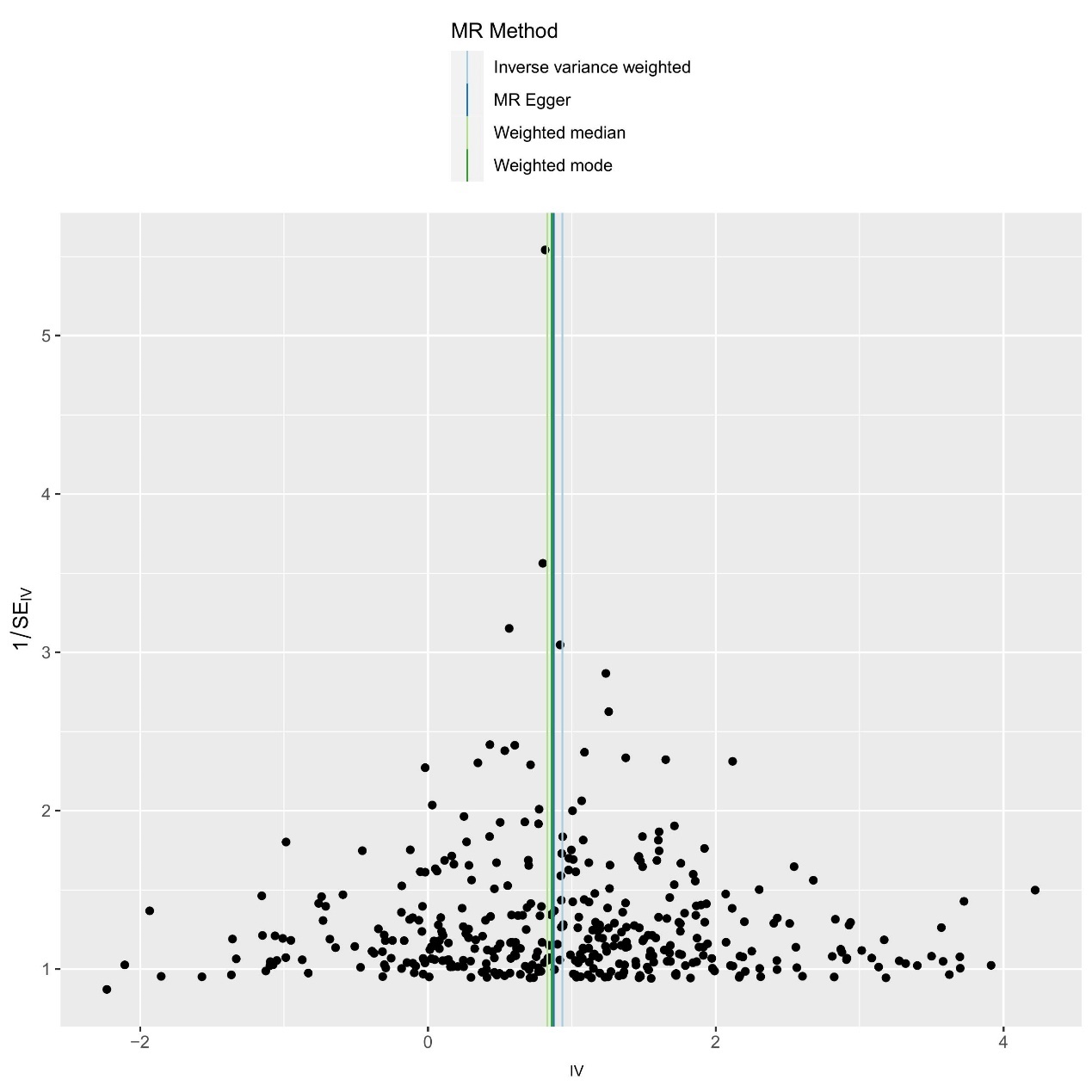


Supplementary Figure 48: Scatter plot of the causal effect of leg fat mass on knee OA.


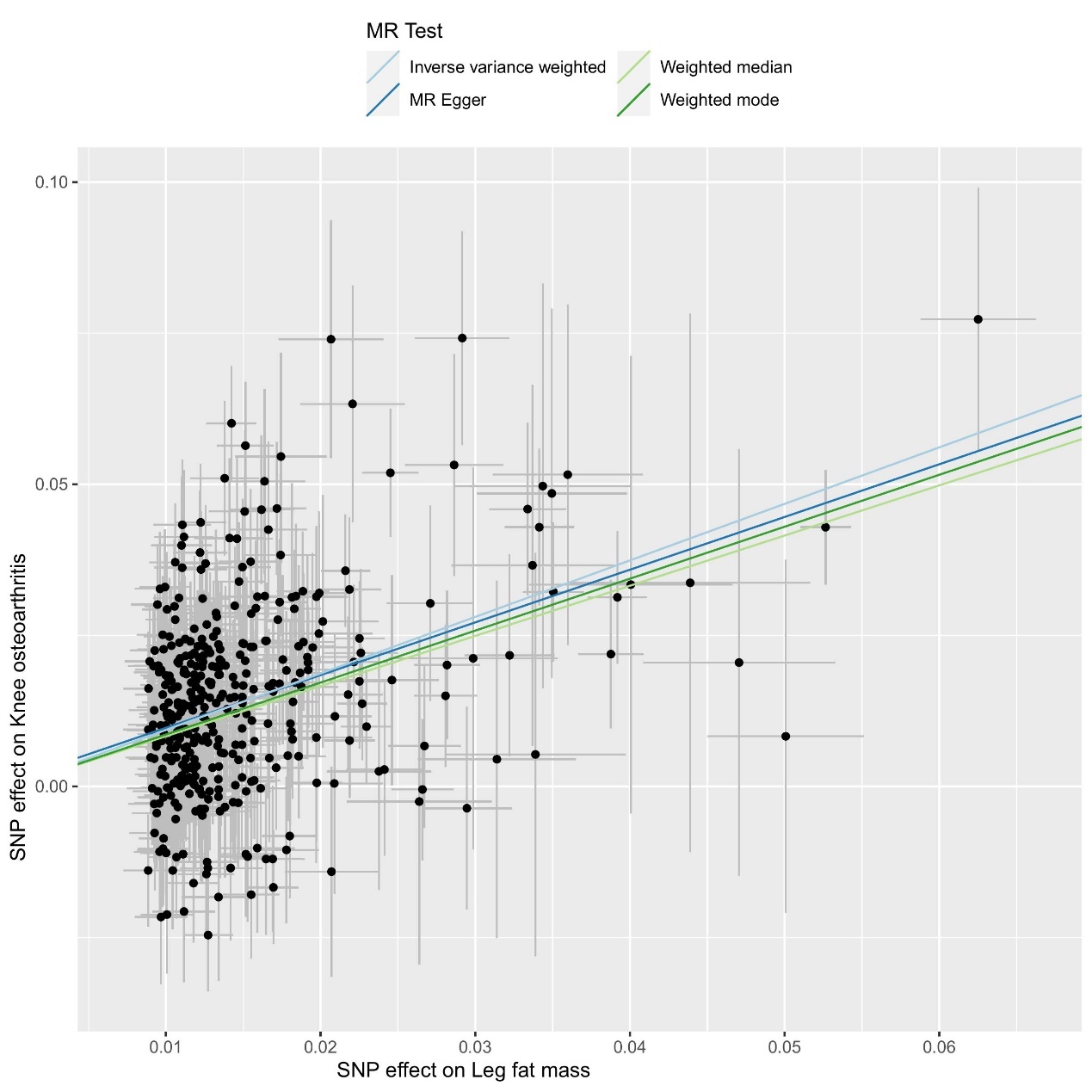


Supplementary Figure 49: Funnel plot of the causal effect of leg fat percentage on hip OA.


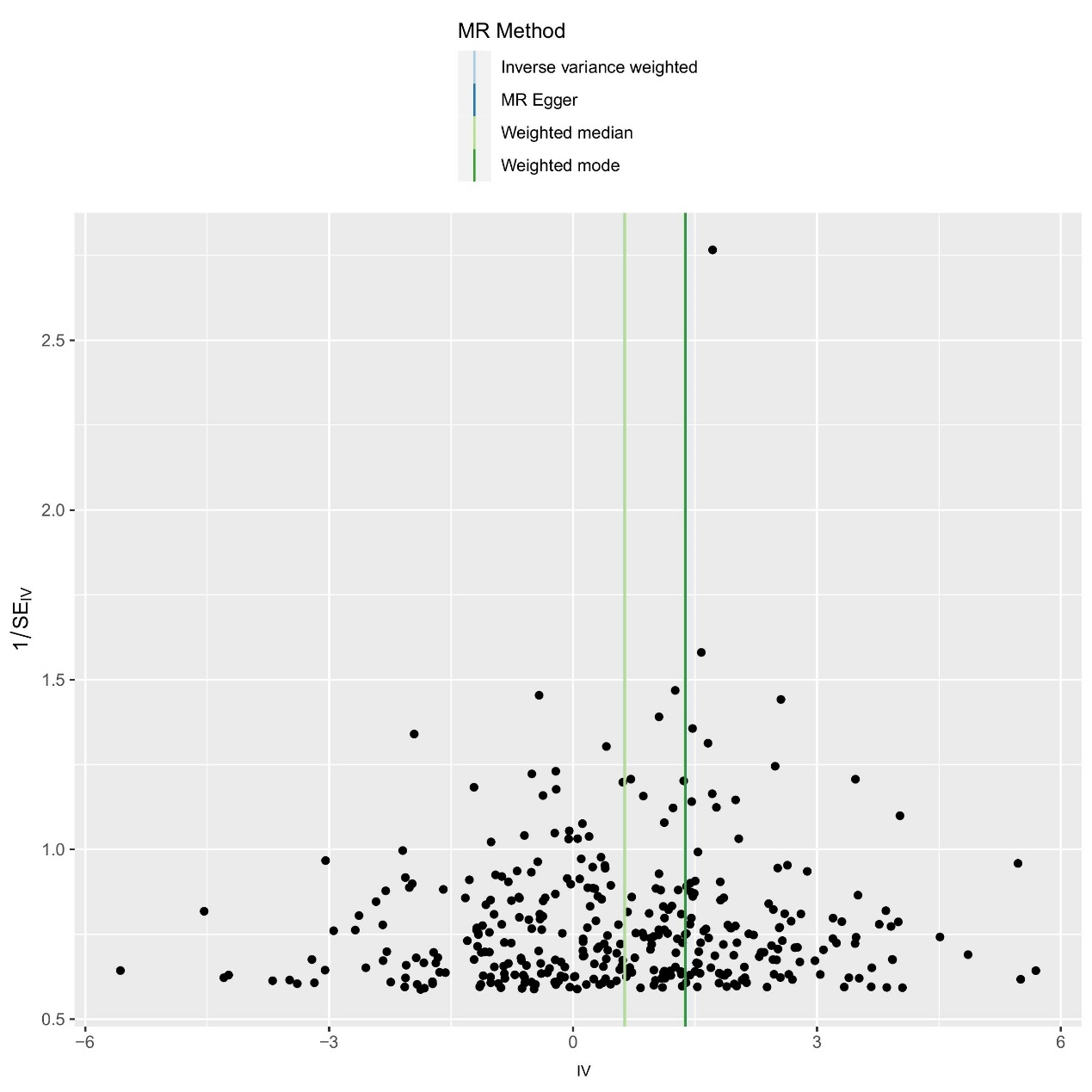


Supplementary Figure 50: Scatter plot of the causal effect of leg fat percentage on hip OA.


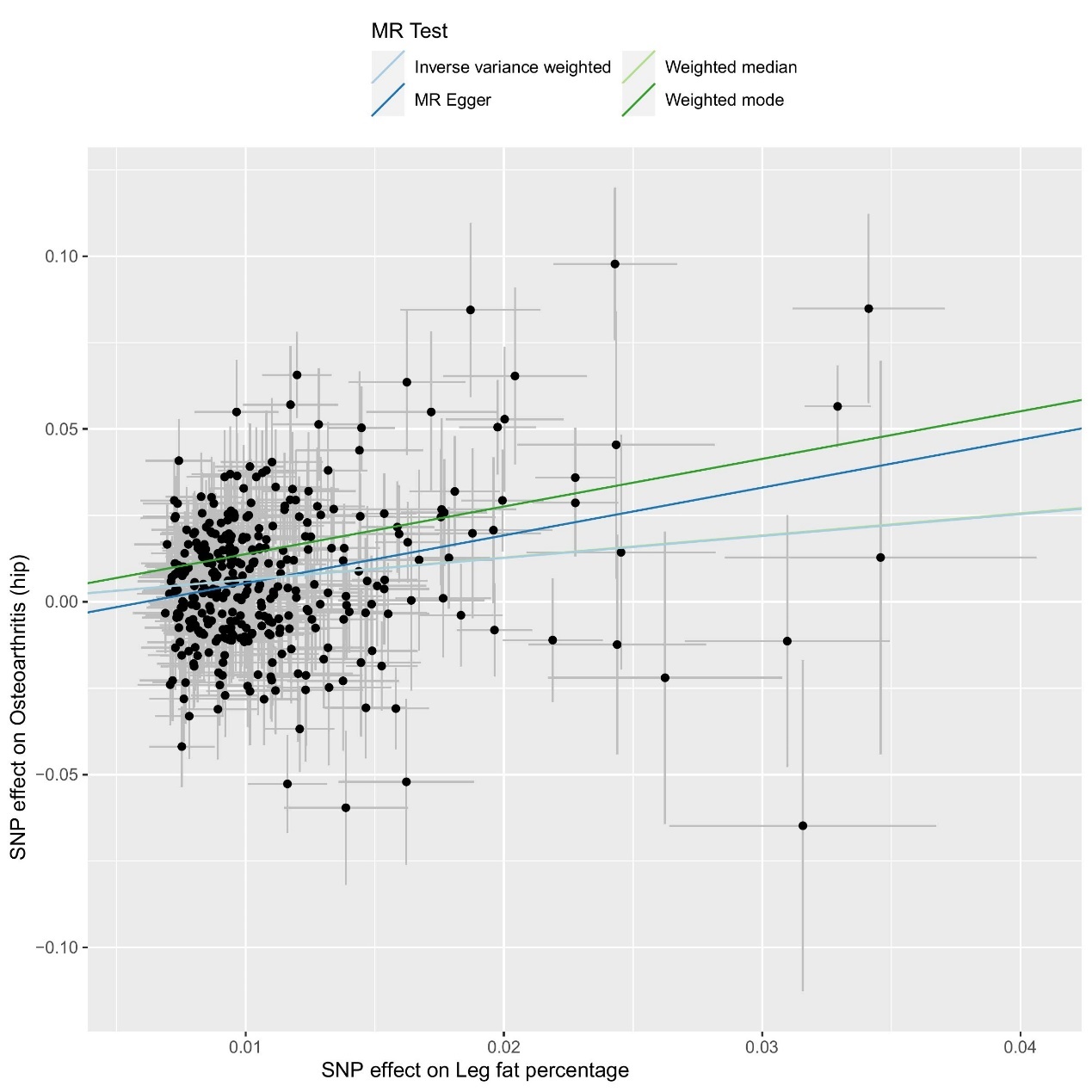


Supplementary Figure 51: Funnel plot of the causal effect of leg fat percentage on knee or hip OA.


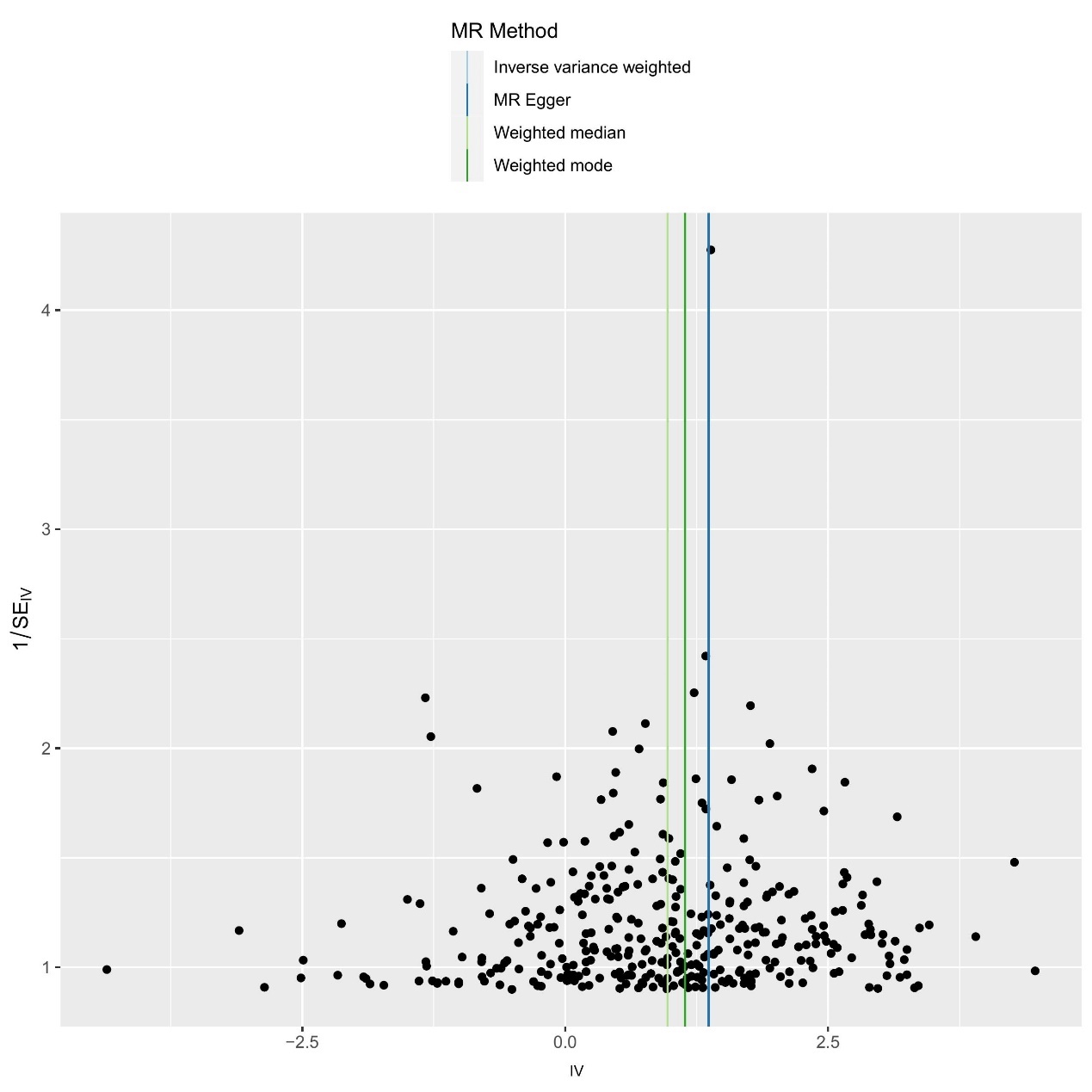


Supplementary Figure 52: Scatter plot of the causal effect of leg fat percentage on knee or hip OA.


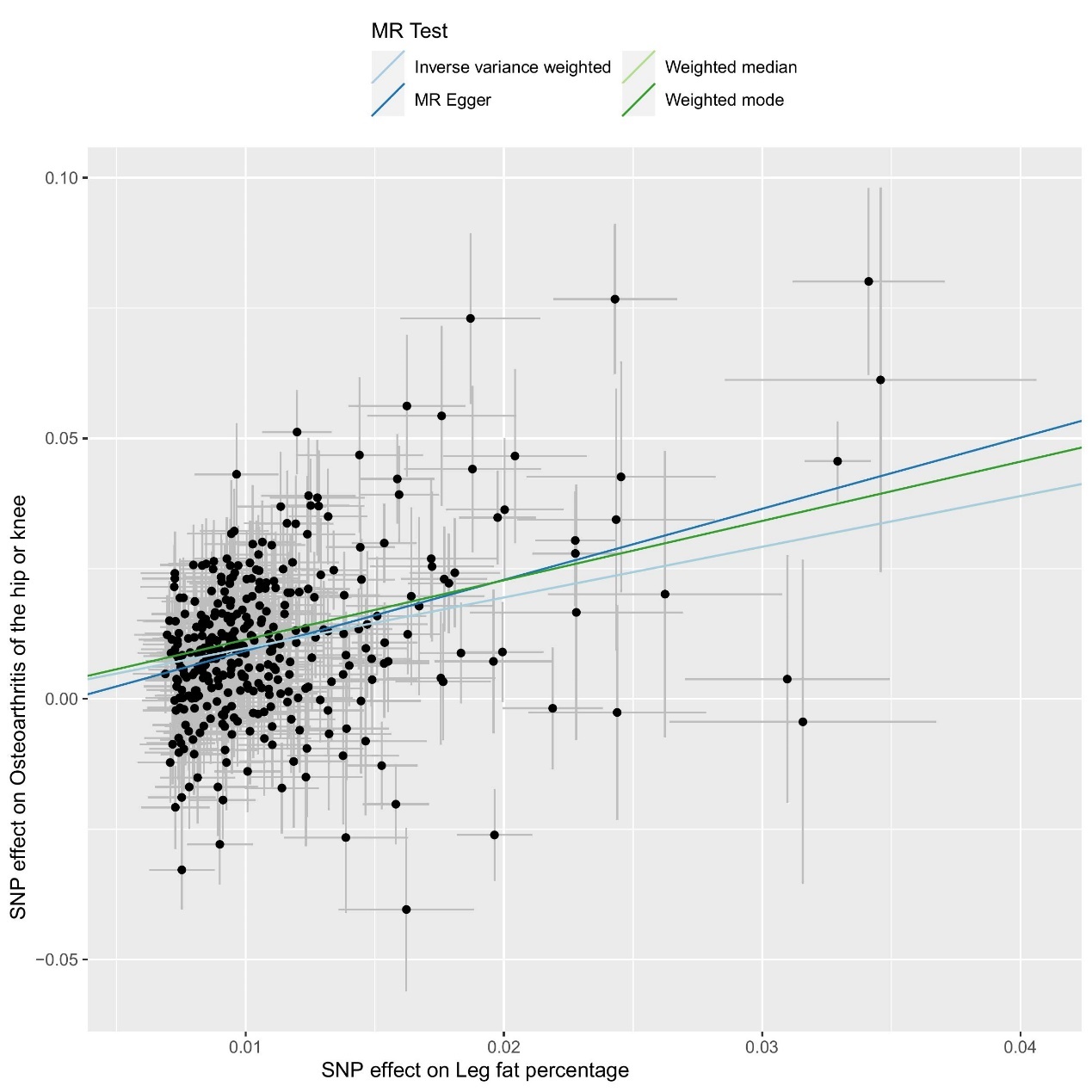


Supplementary Figure 53: Funnel plot of the causal effect of leg fat percentage on knee OA.


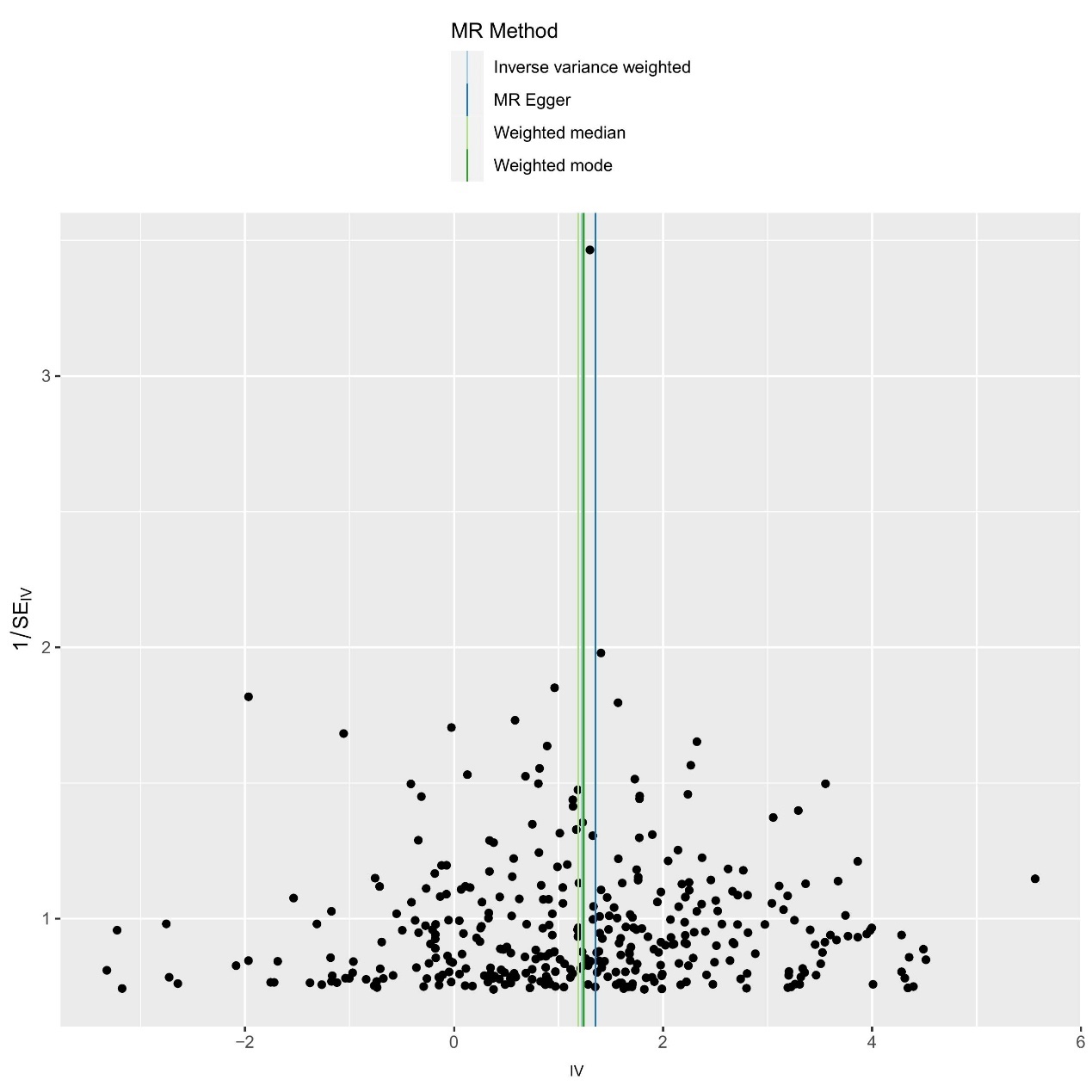


Supplementary Figure 54: Scatter plot of the causal effect of leg fat percentage on knee OA.


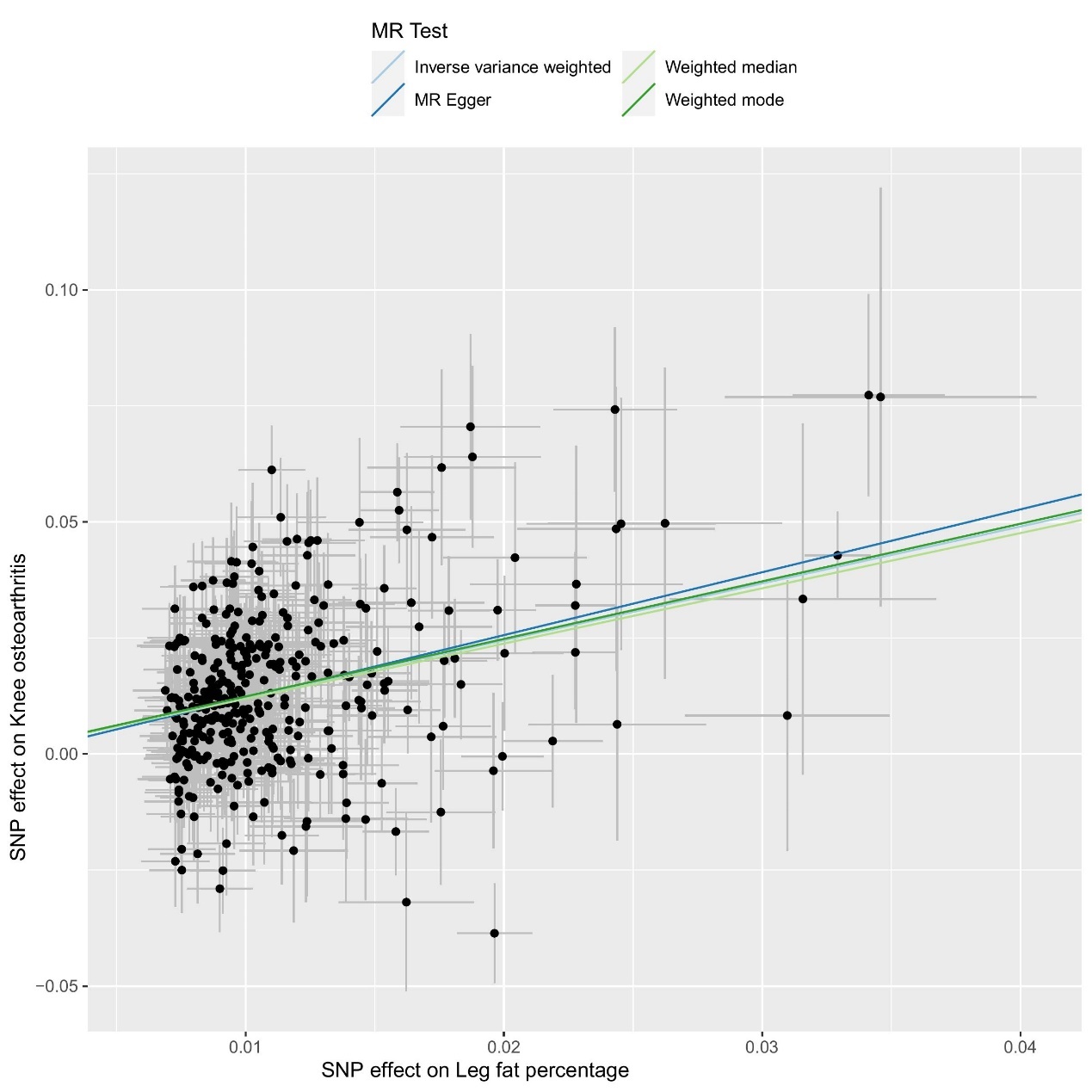


Supplementary Figure 55: Funnel plot of the causal effect of obesity class 1 on hip OA.


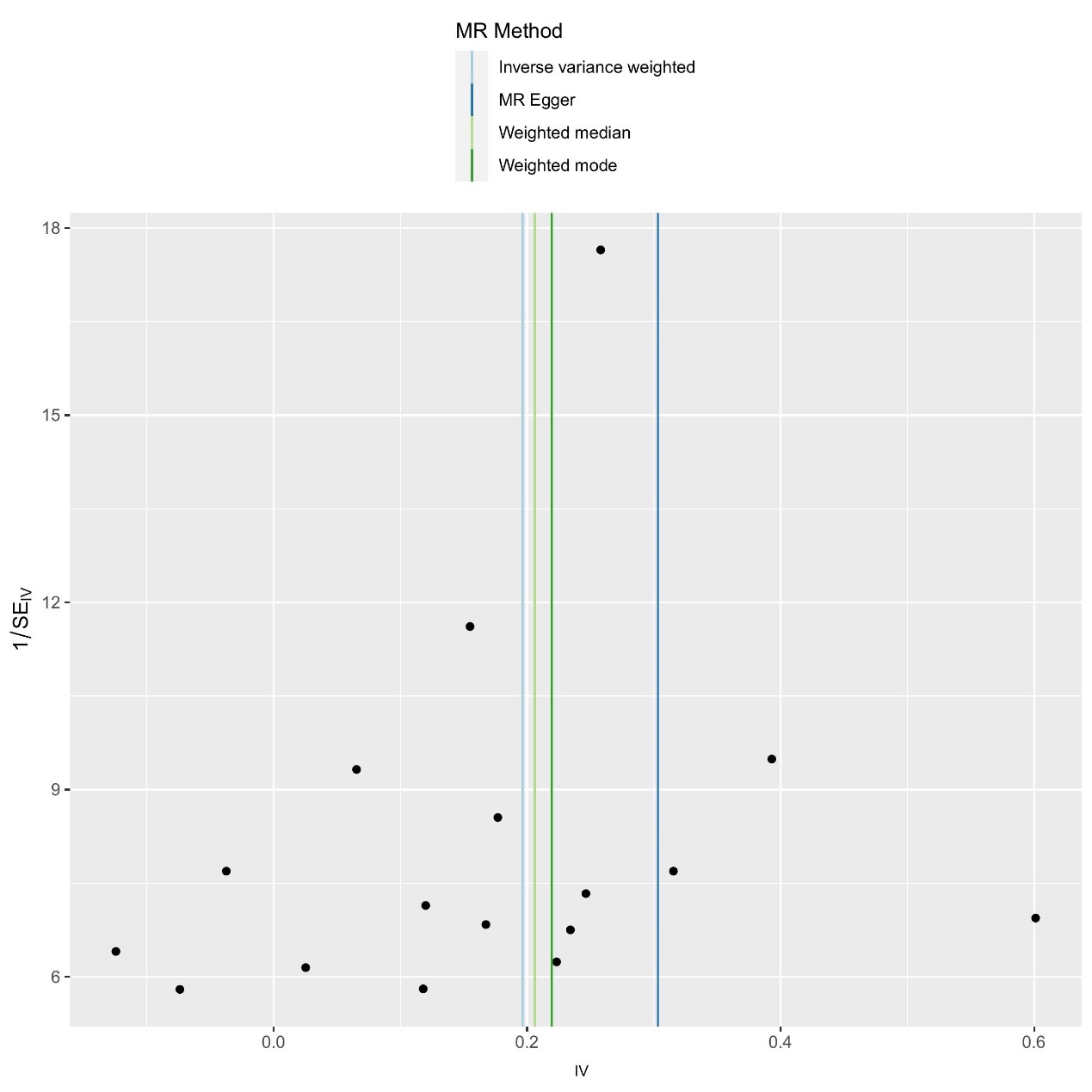


Supplementary Figure 56: Scatter plot of the causal effect of obesity class 1 on hip OA.


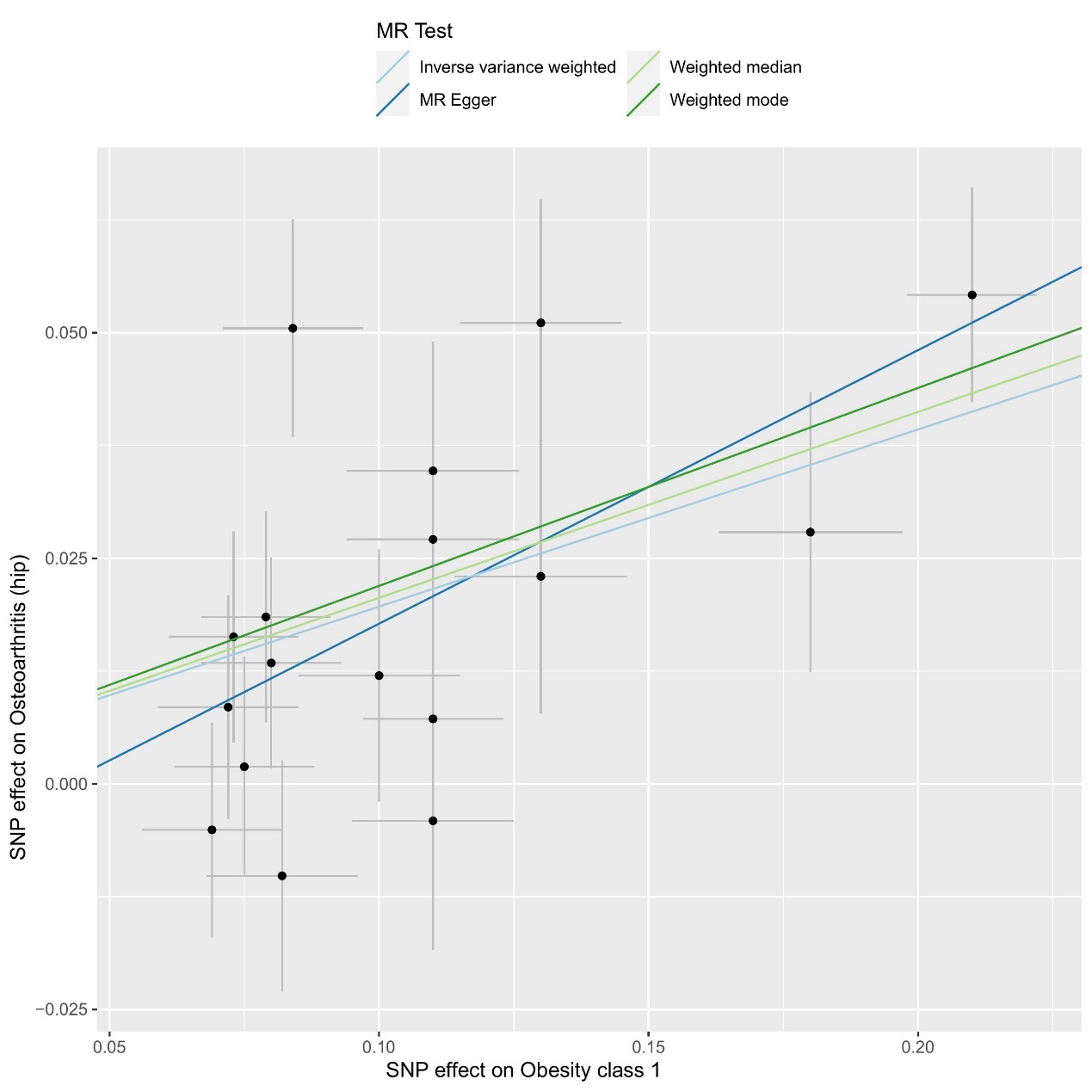


Supplementary Figure 57: Funnel plot of the causal effect of obesity class 1 on knee or hip OA.


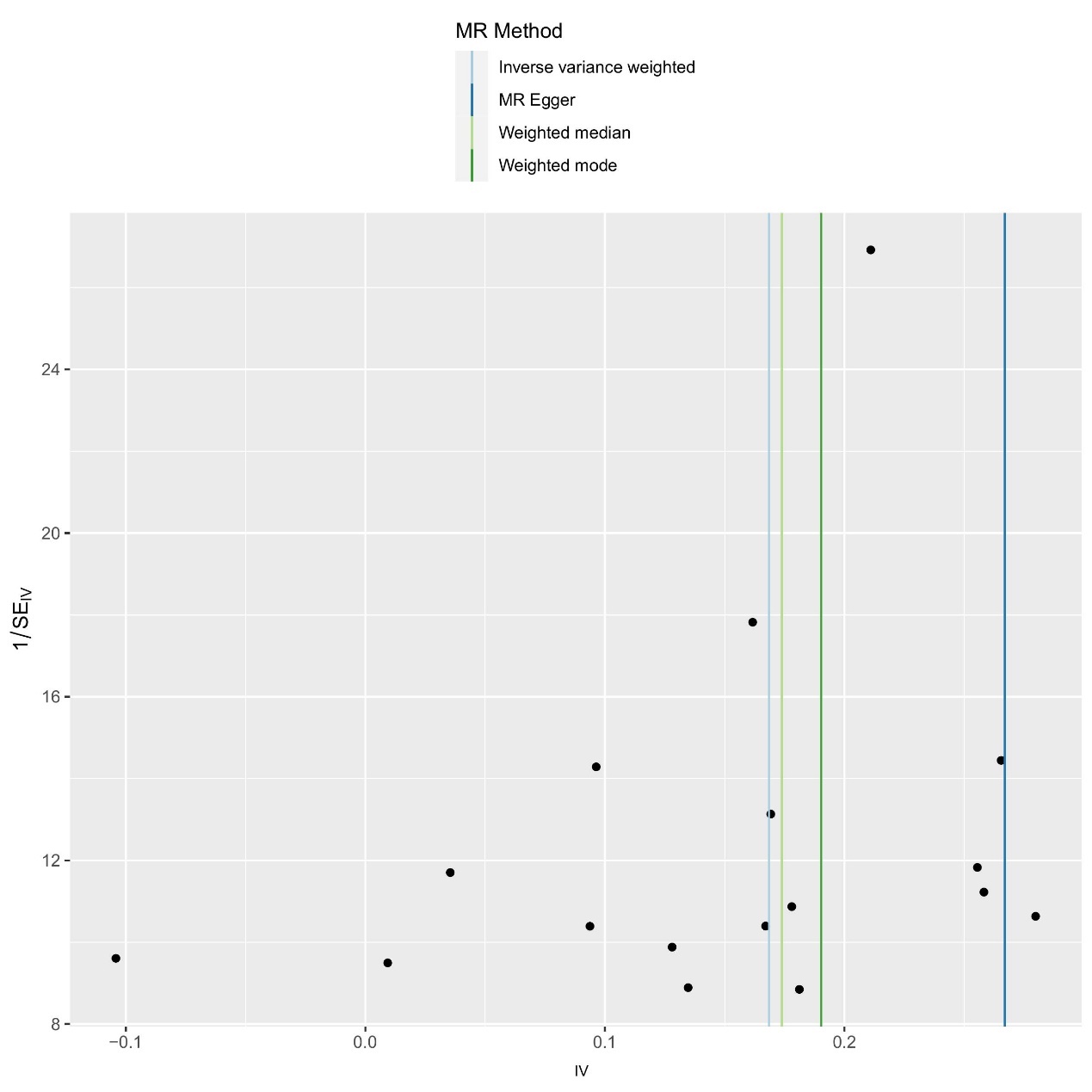


Supplementary Figure 58: Scatter plot of the causal effect of obesity class 1 on knee or hip OA.


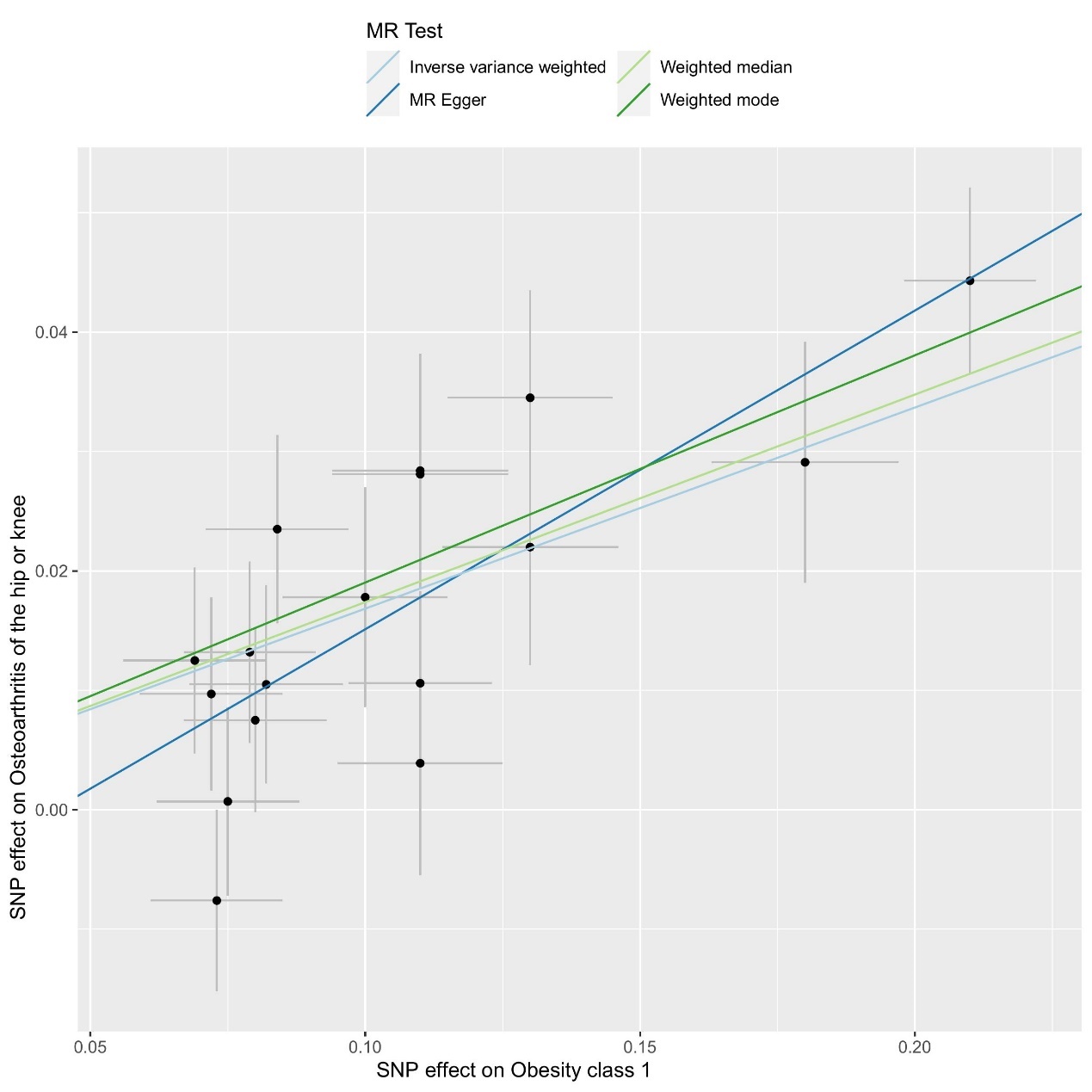


Supplementary Figure 59: Funnel plot of the causal effect of obesity class 1 on knee OA.


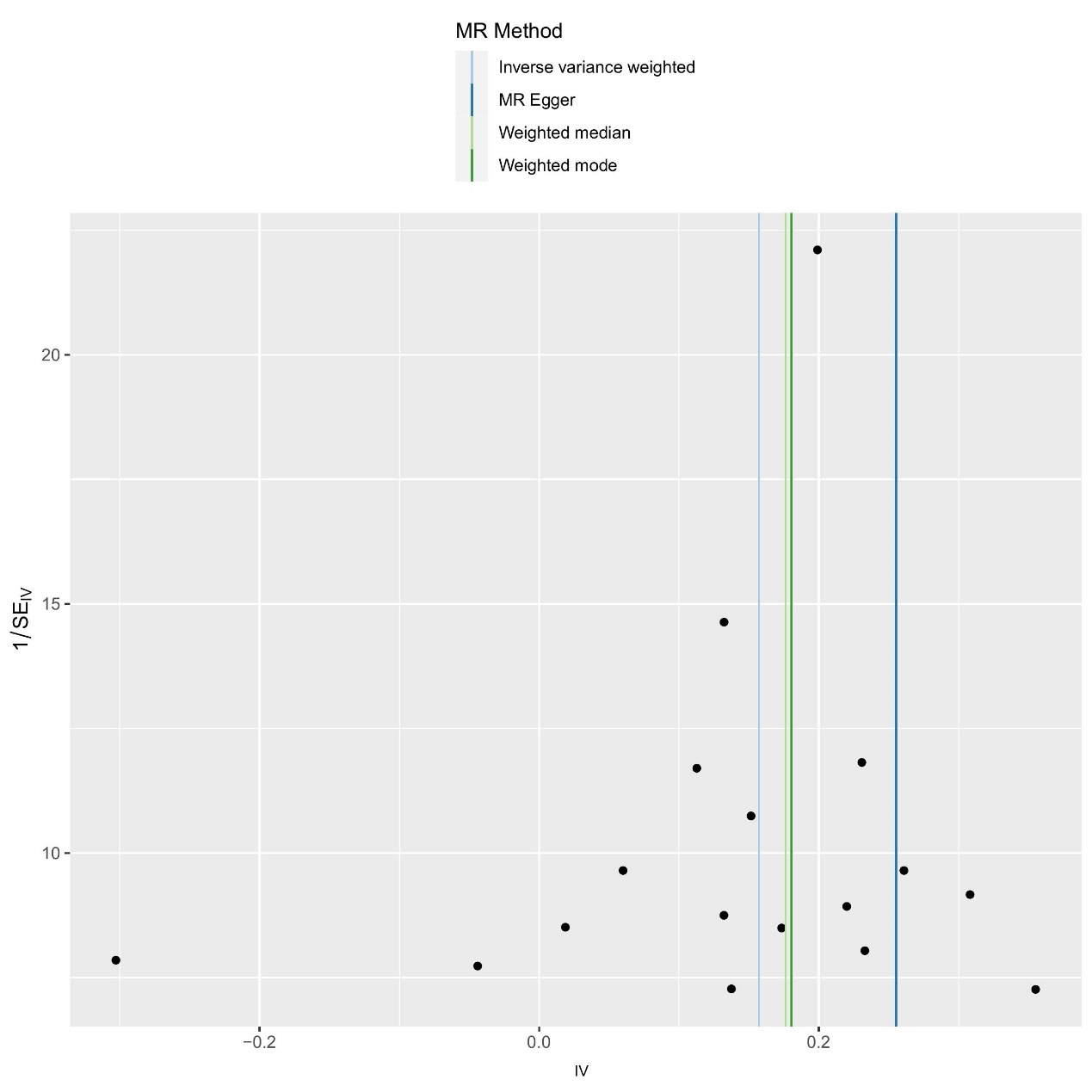


Supplementary Figure 60: Scatter plot of the causal effect of obesity class 1 on knee OA.


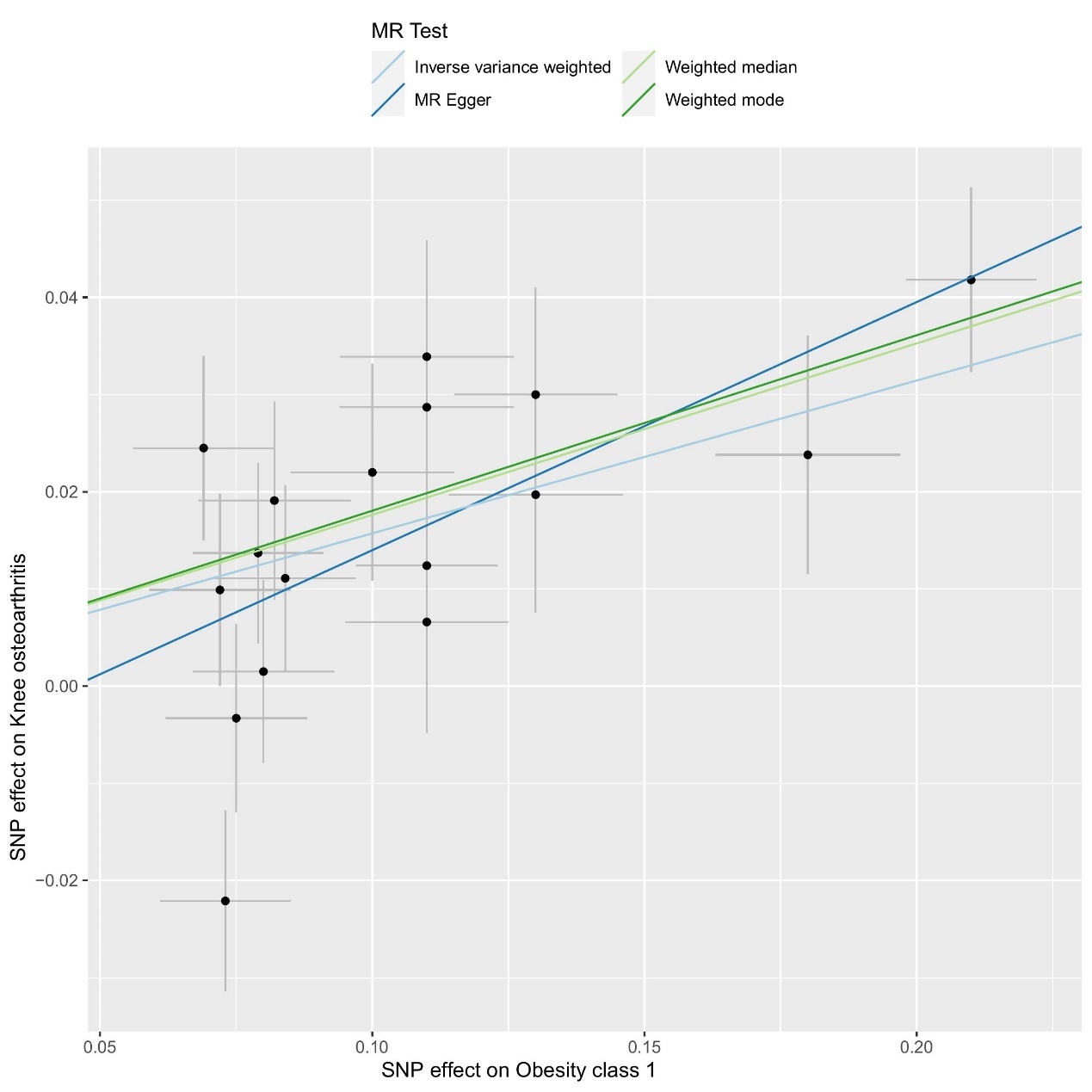


Supplementary Figure 61: Funnel plot of the causal effect of obesity class 2 on hip OA.


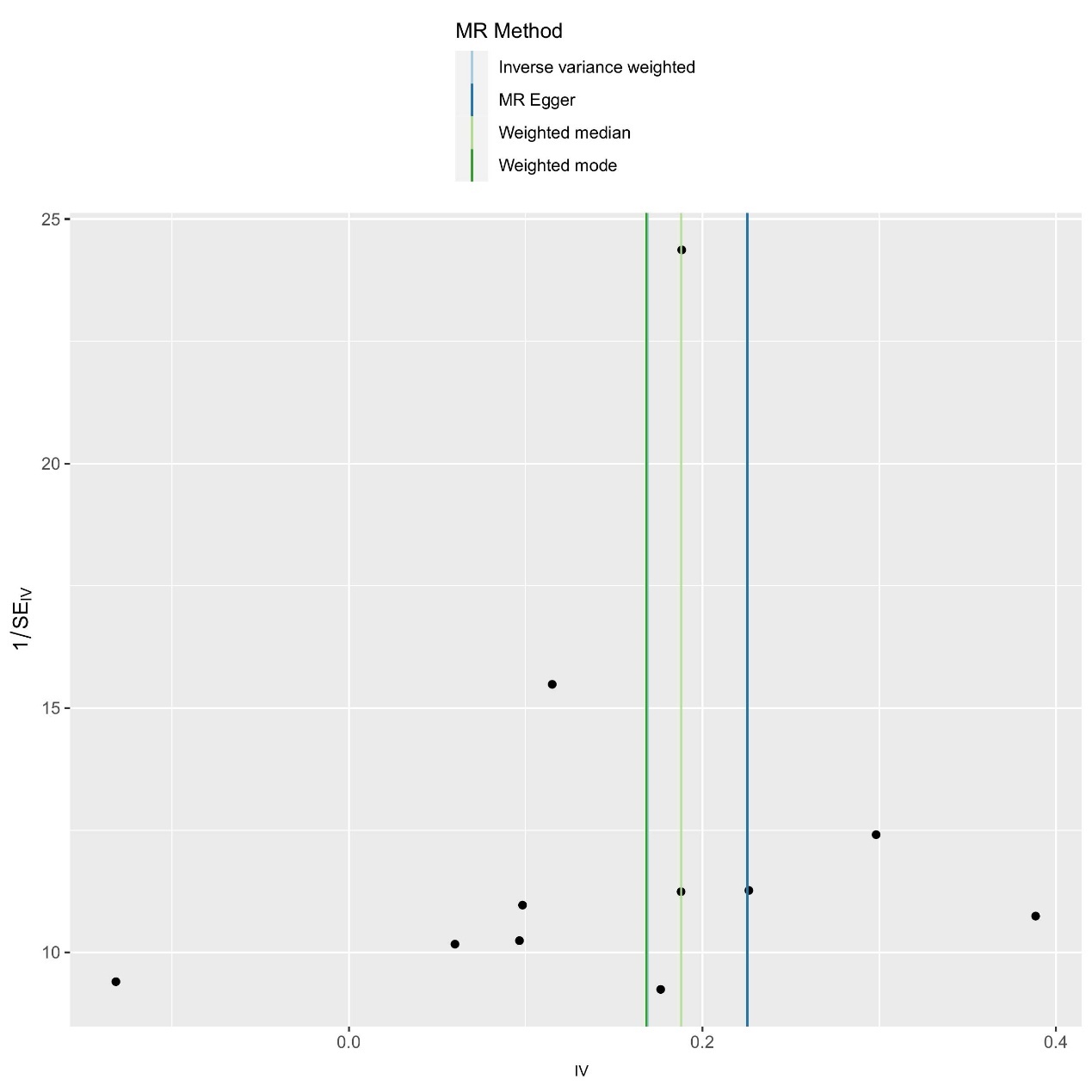


Supplementary Figure 62: Scatter plot of the causal effect of obesity class 2 on hip OA.


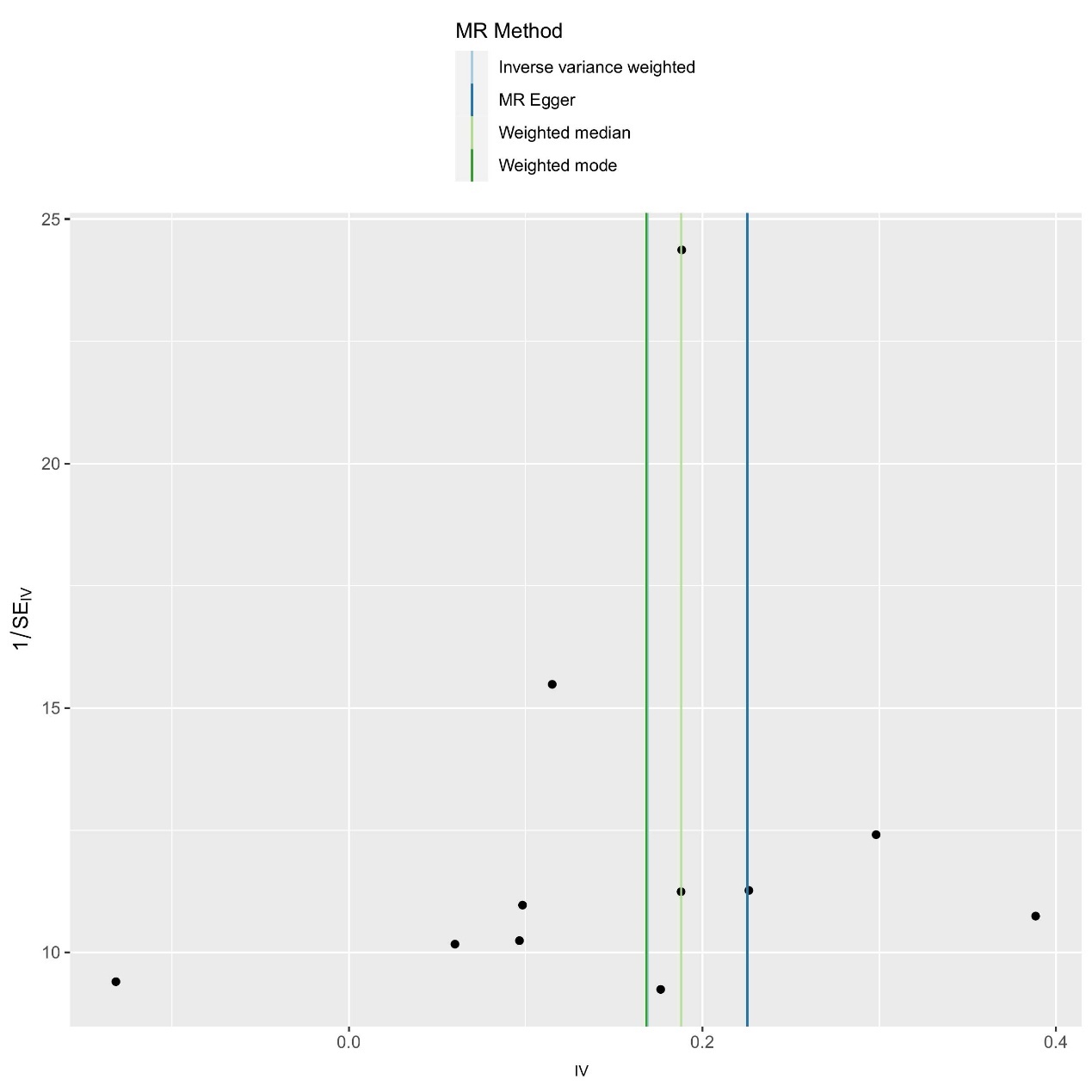


Supplementary Figure 63: Funnel plot of the causal effect of obesity class 2 on knee or hip OA.


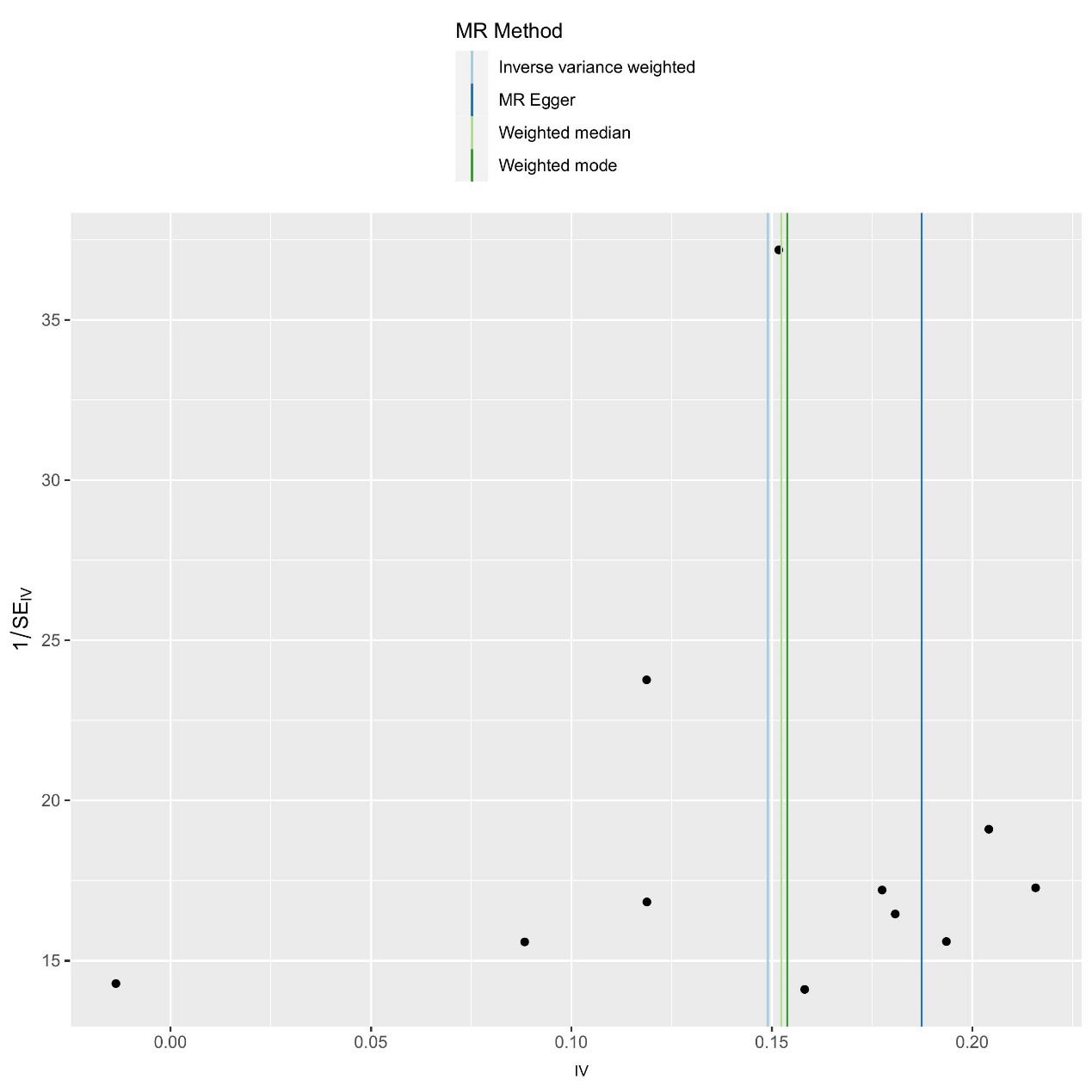


Supplementary Figure 64: Scatter plot of the causal effect of obesity class 2 on knee or hip OA.


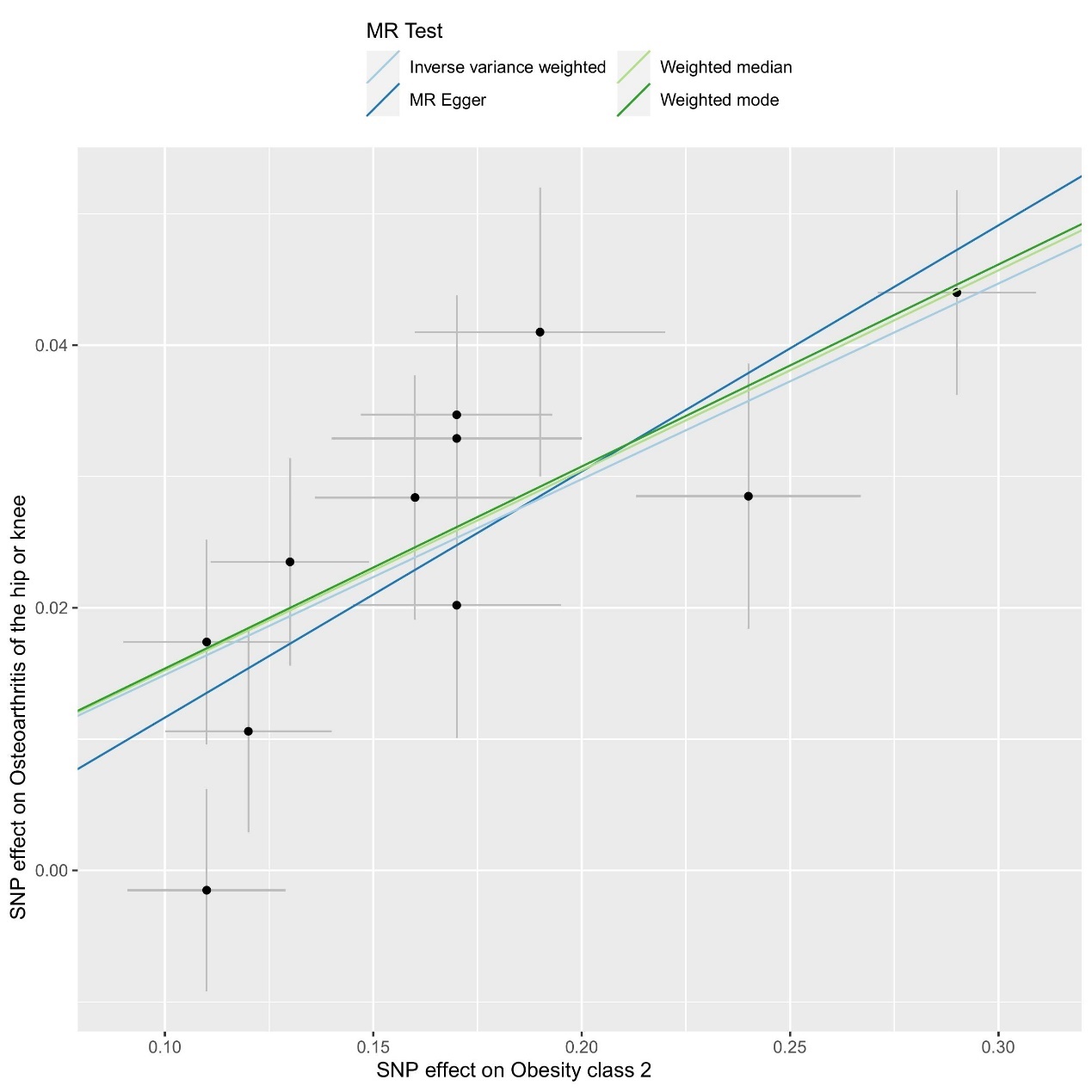


Supplementary Figure 65: Funnel plot of the causal effect of obesity class 2 on knee OA.


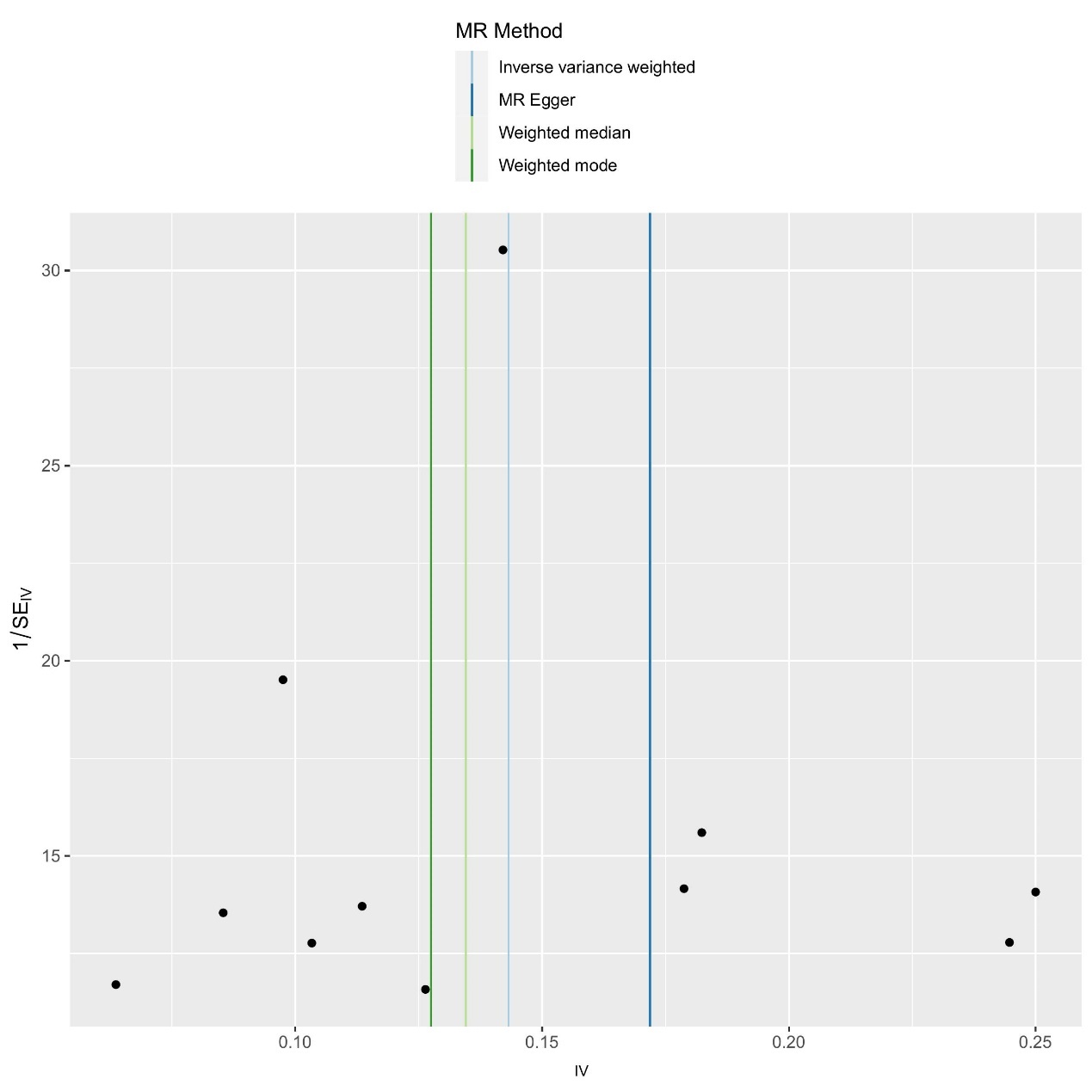


Supplementary Figure 66: Scatter plot of the causal effect of obesity class 2 on knee OA.


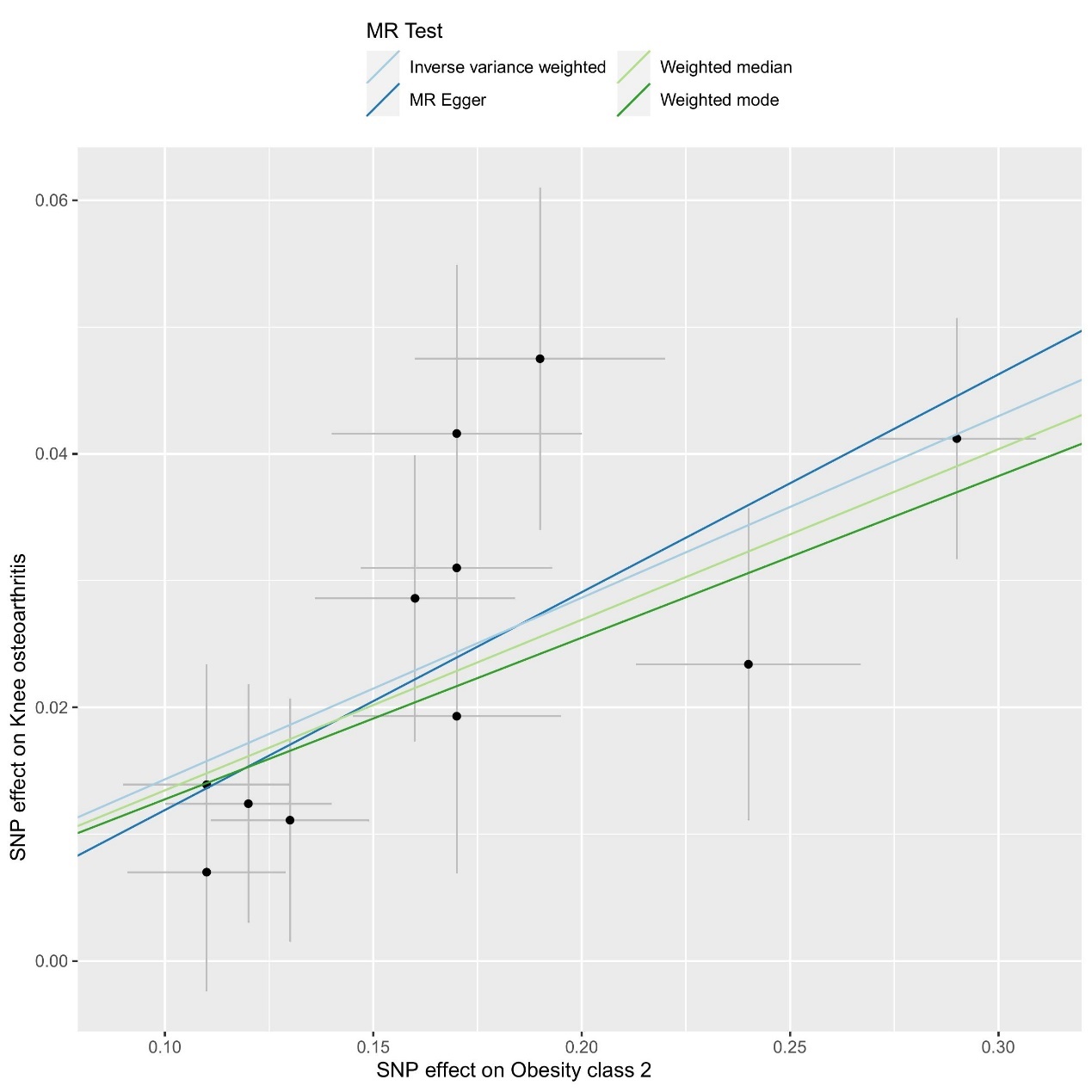


Supplementary Figure 67: Funnel plot of the causal effect of obesity class 3 on hip OA.


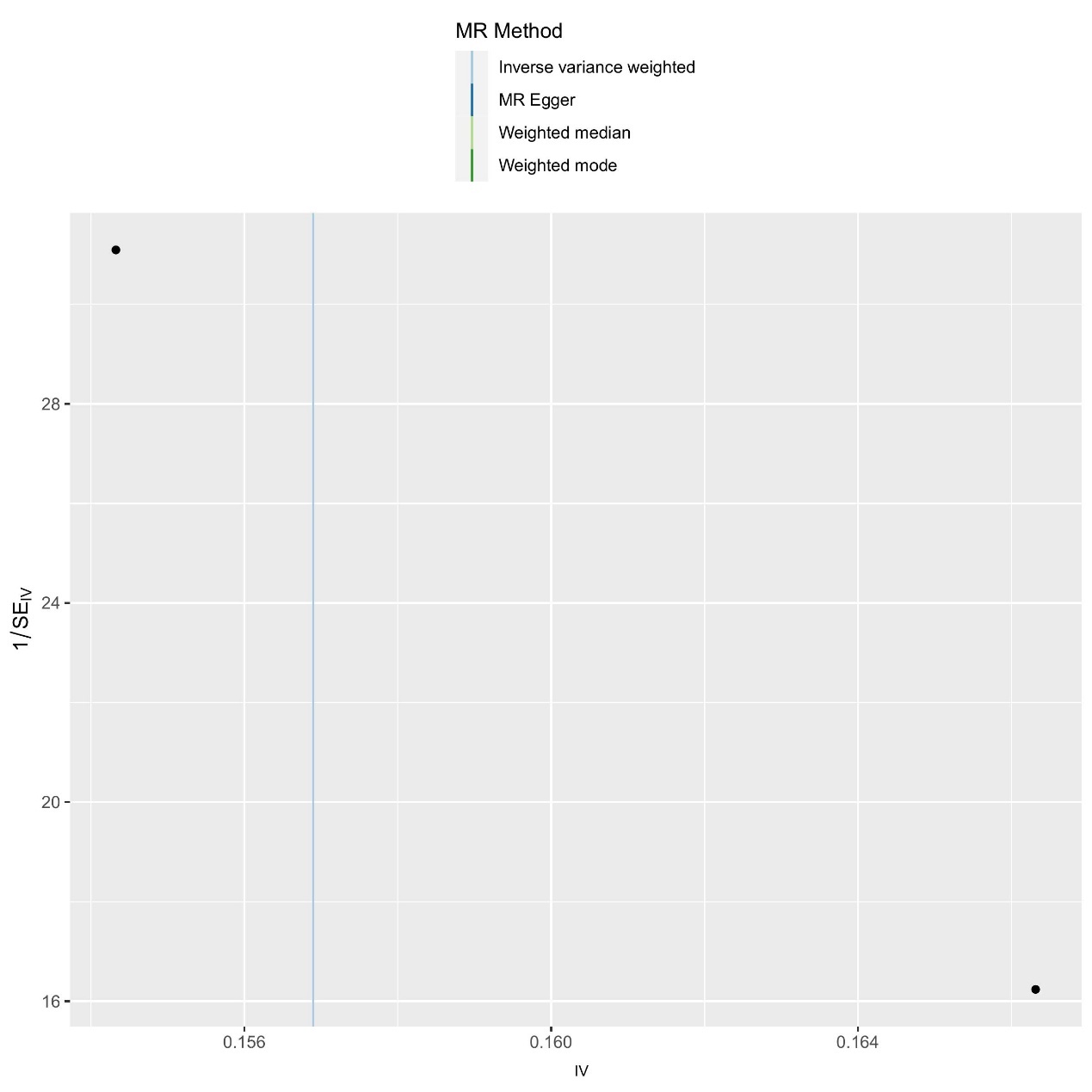


Supplementary Figure 68: Scatter plot of the causal effect of obesity class 3 on hip OA.


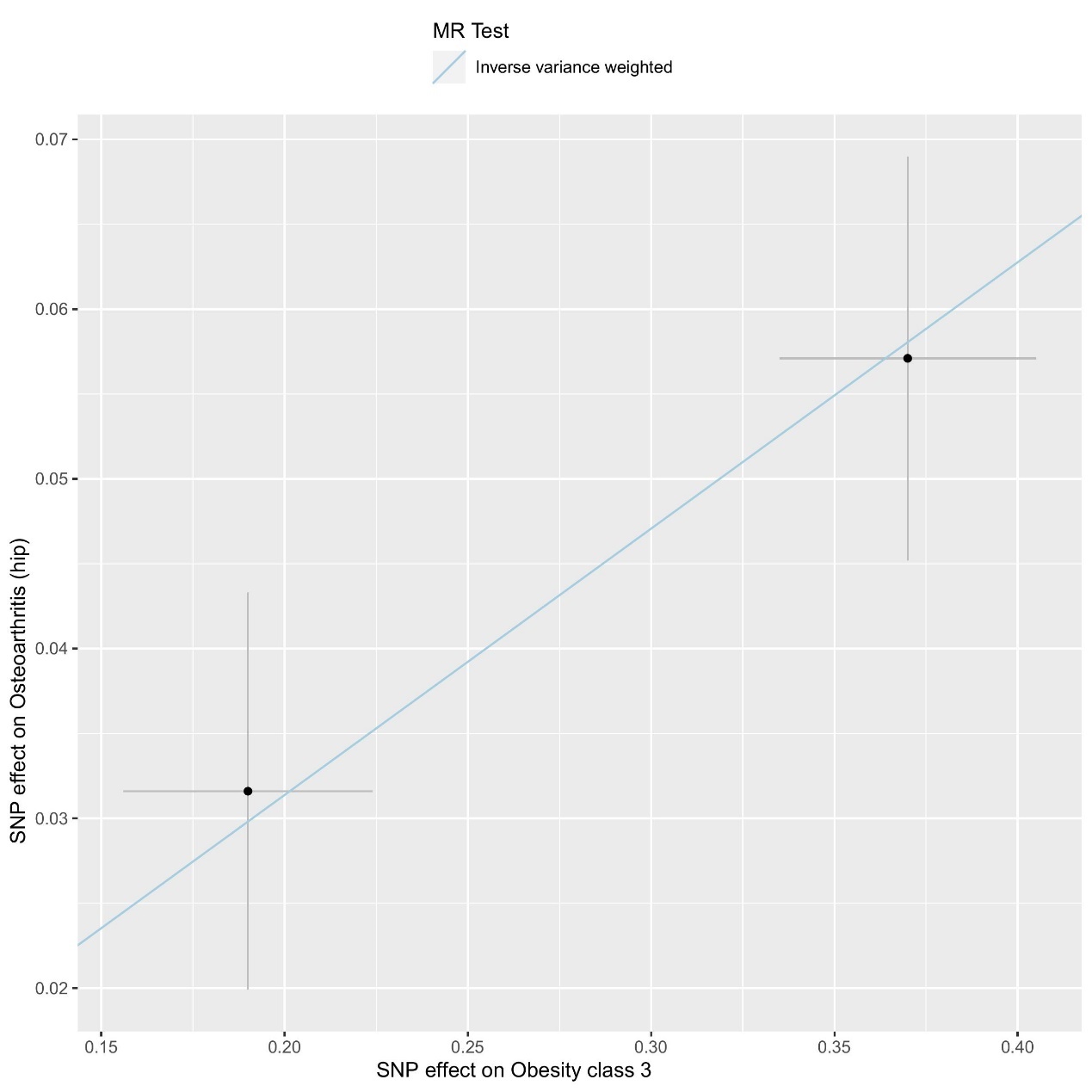


Supplementary Figure 69: Funnel plot of the causal effect of obesity class 3 on knee or hip OA.


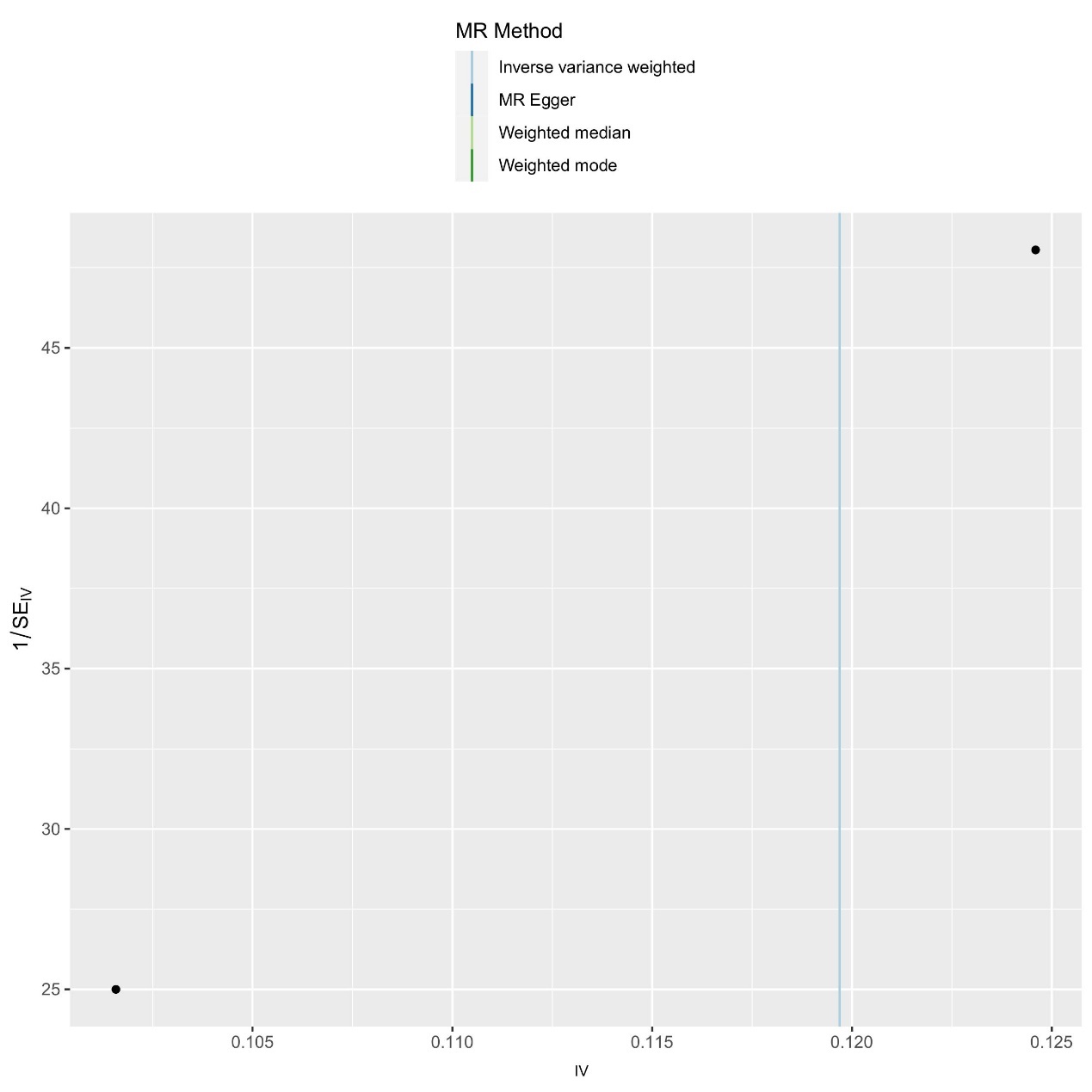


Supplementary Figure 70: Scatter plot of the causal effect of obesity class 3 on knee or hip OA.


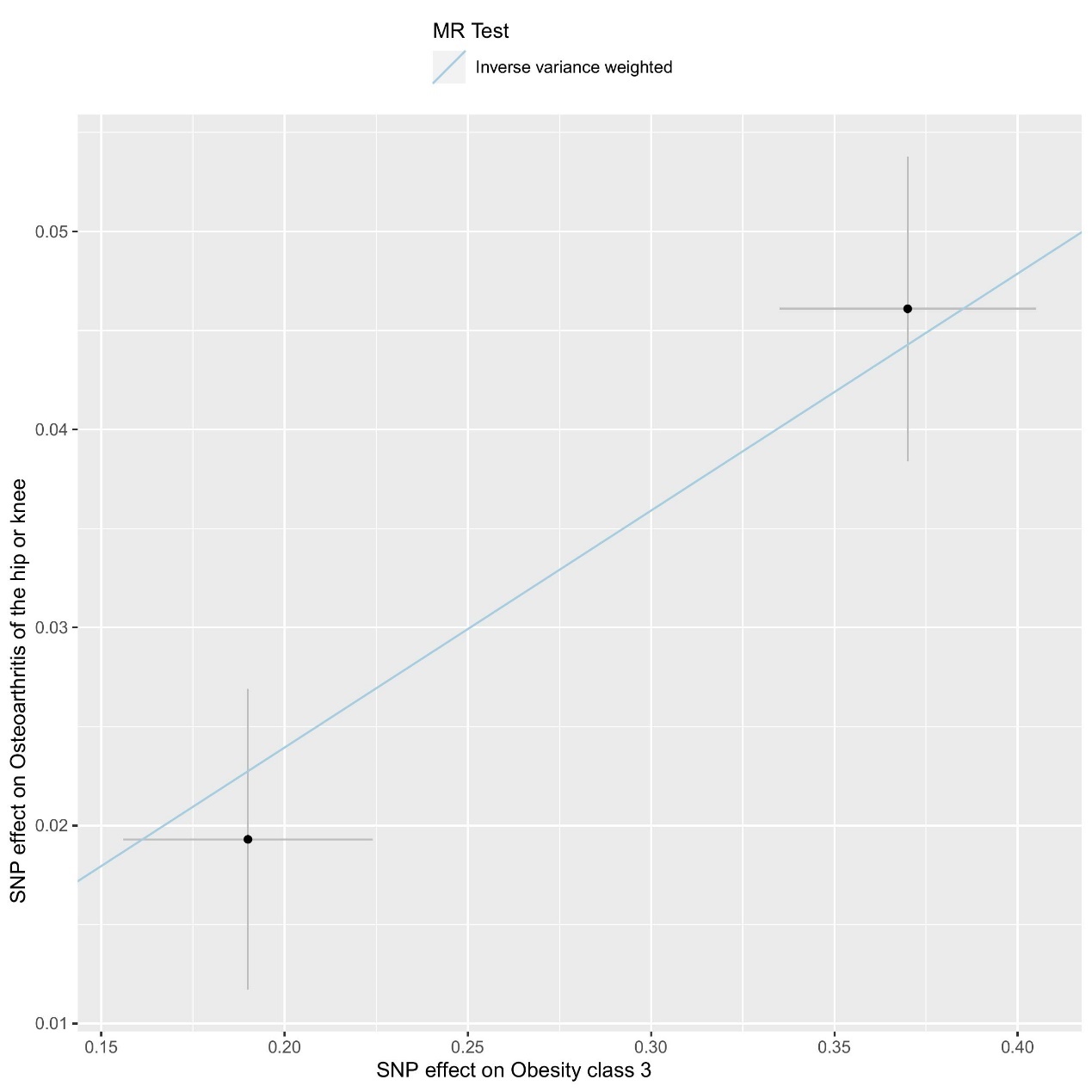


Supplementary Figure 71: Funnel plot of the causal effect of obesity class 3 on knee OA.


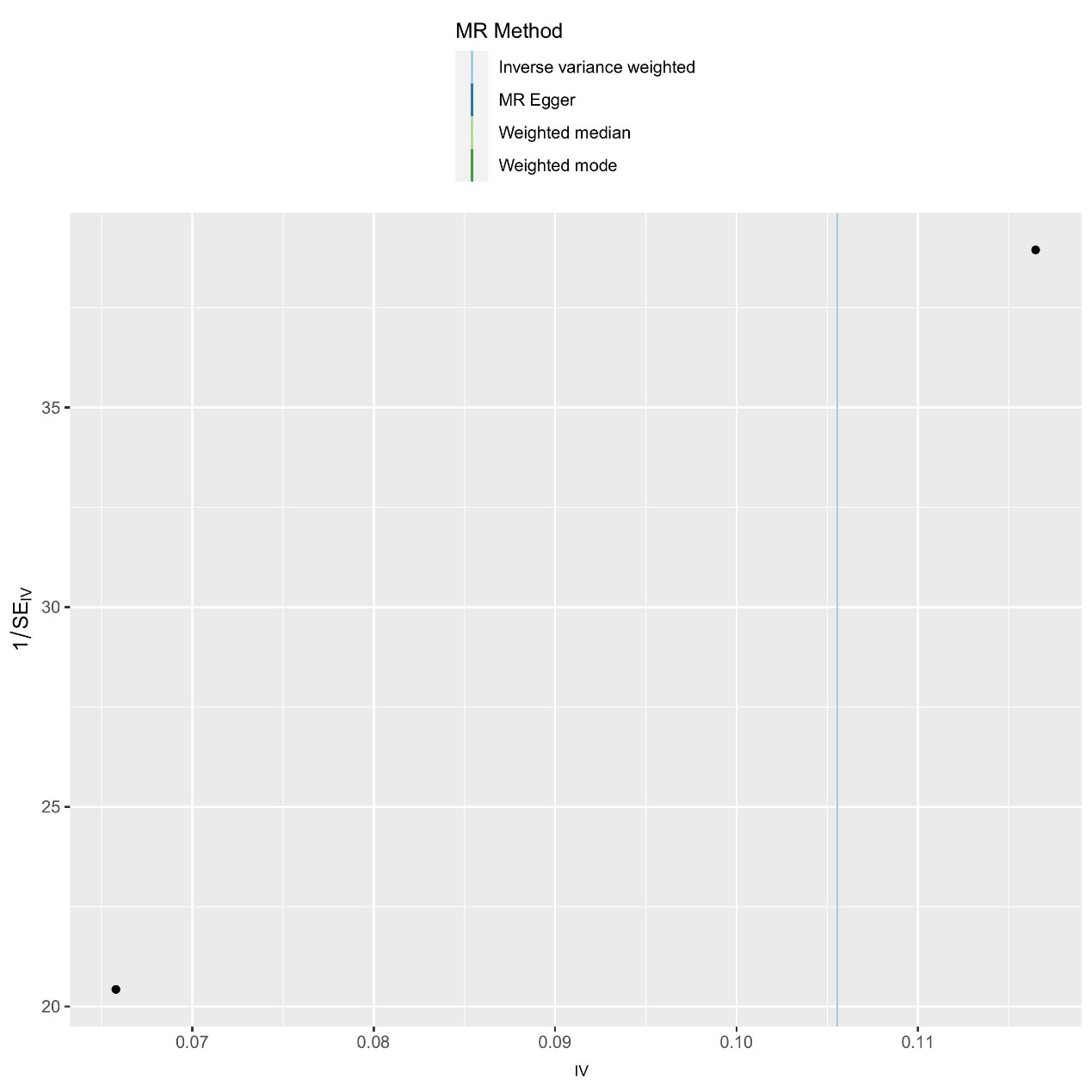


Supplementary Figure 72: Scatter plot of the causal effect of obesity class 3 on knee OA.


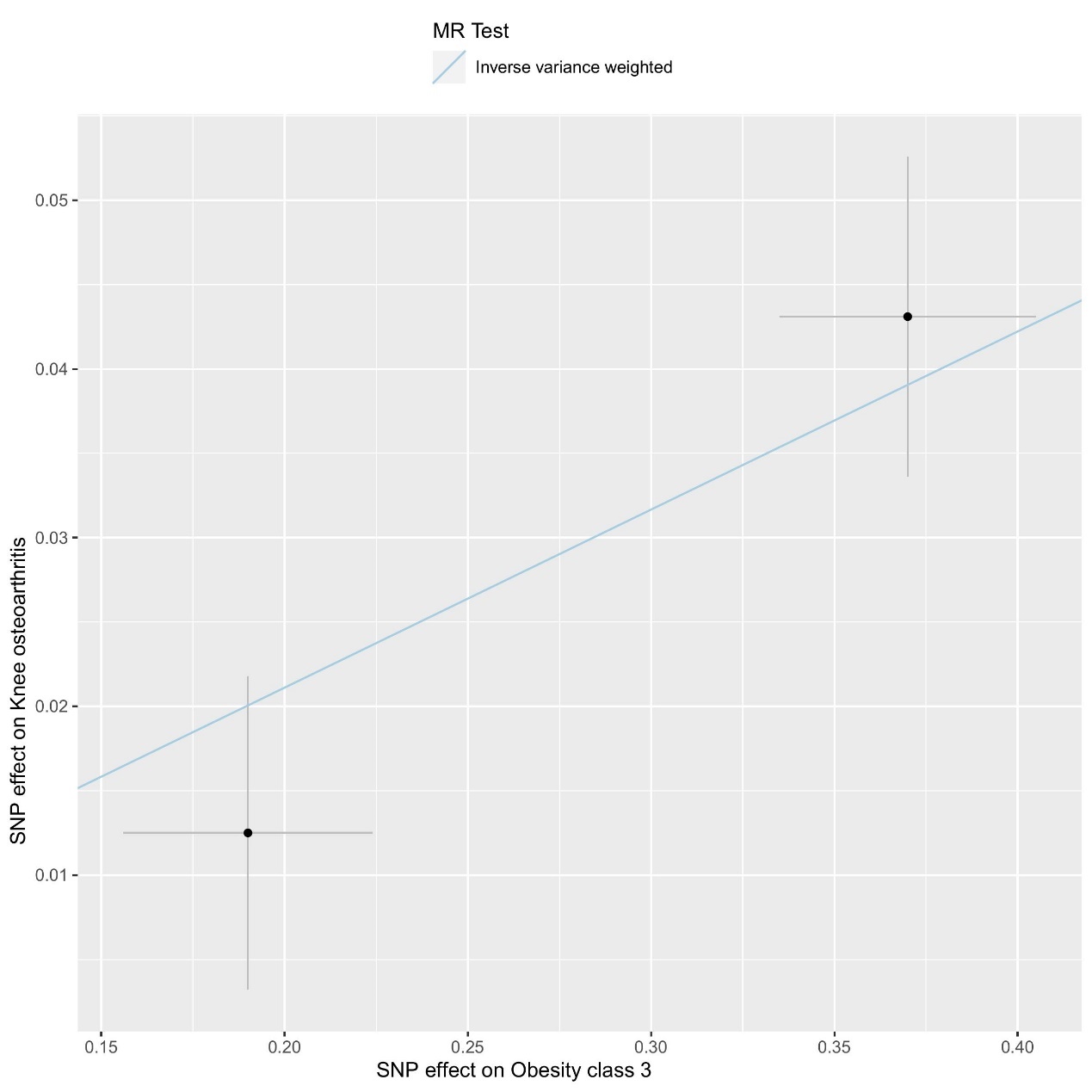


Supplementary Figure 73: Funnel plot of the causal effect of trunk fat-free mass on hip OA.


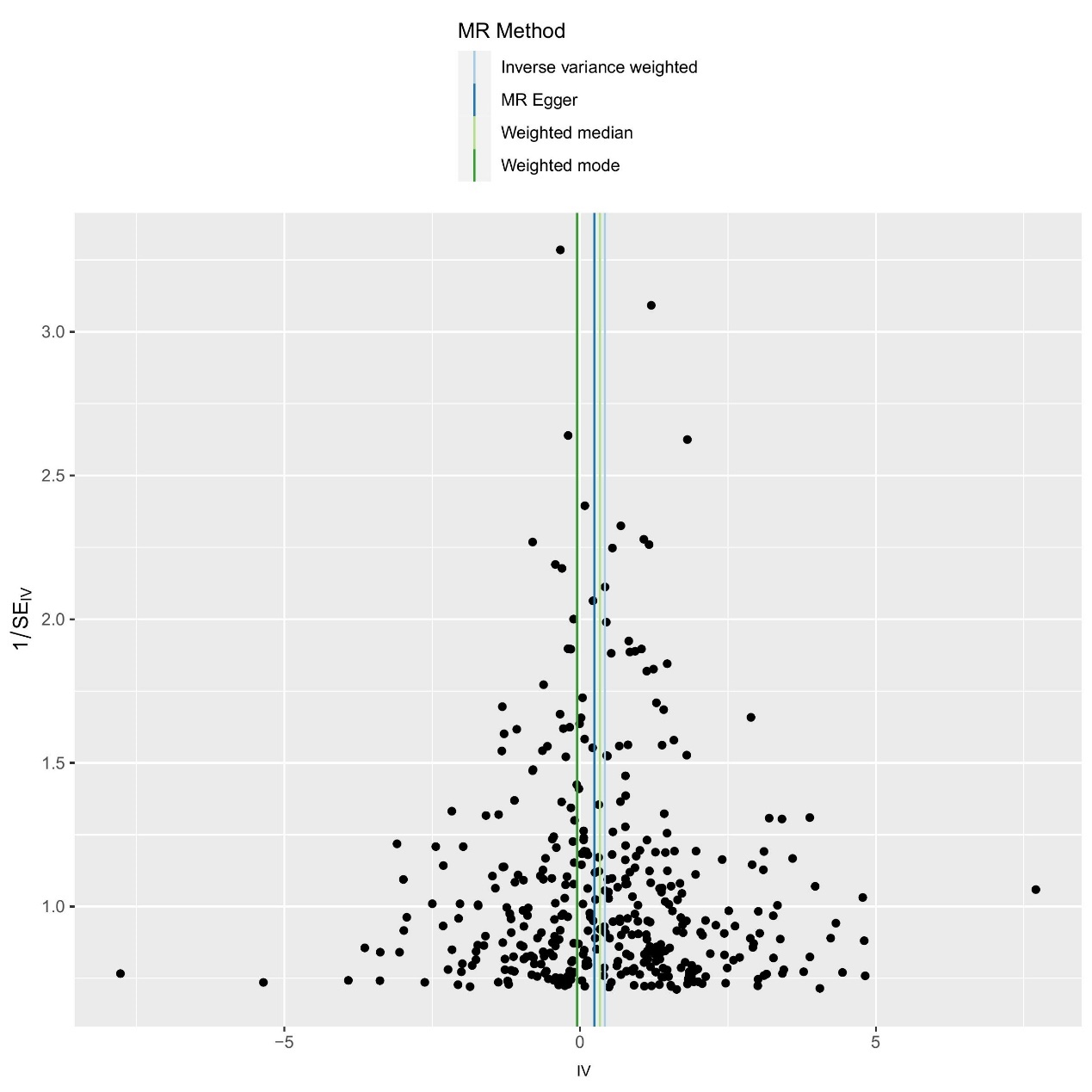


Supplementary Figure 74: Scatter plot of the causal effect of trunk fat-free mass on hip OA.


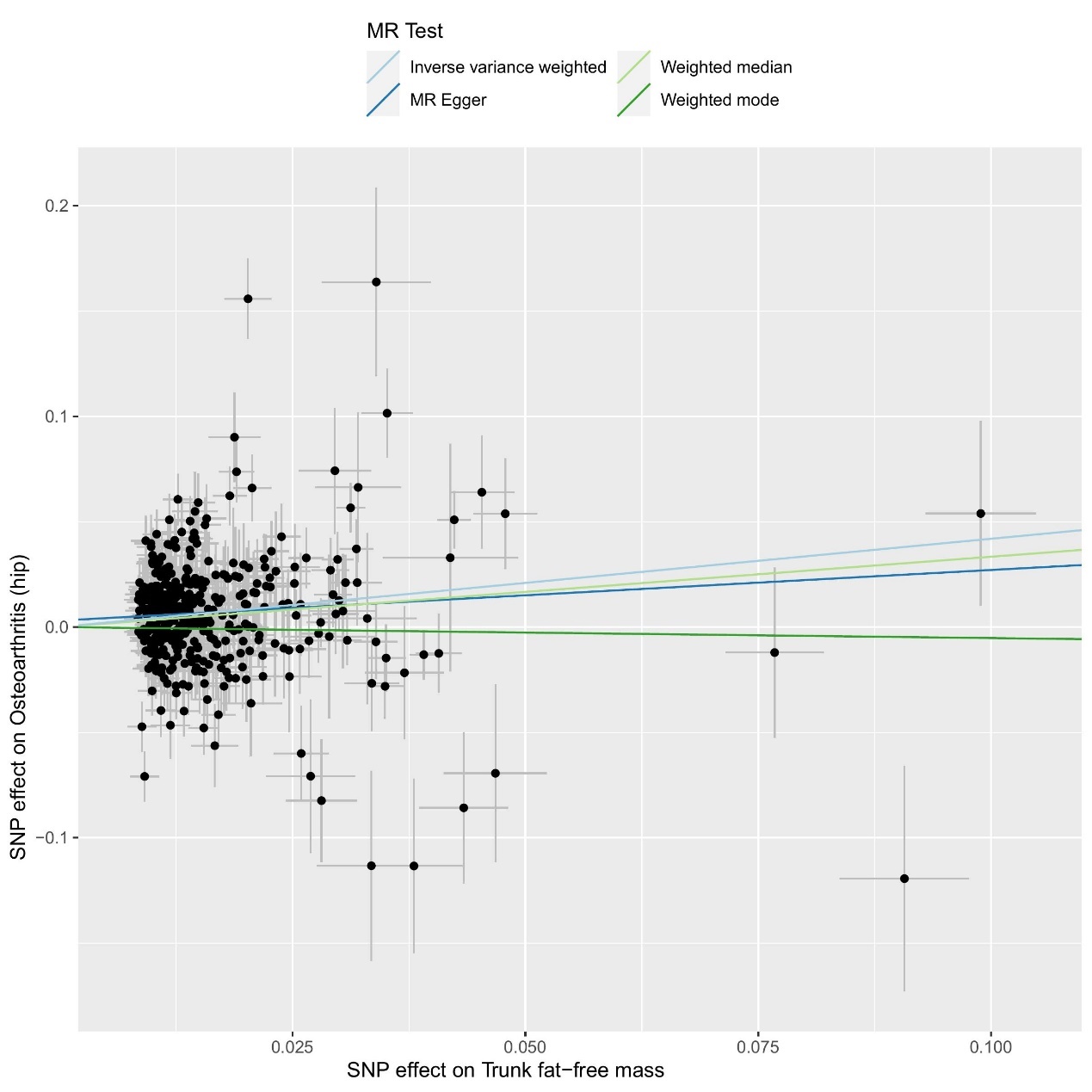


Supplementary Figure 75: Funnel plot of the causal effect of trunk fat-free mass on knee or hip OA.


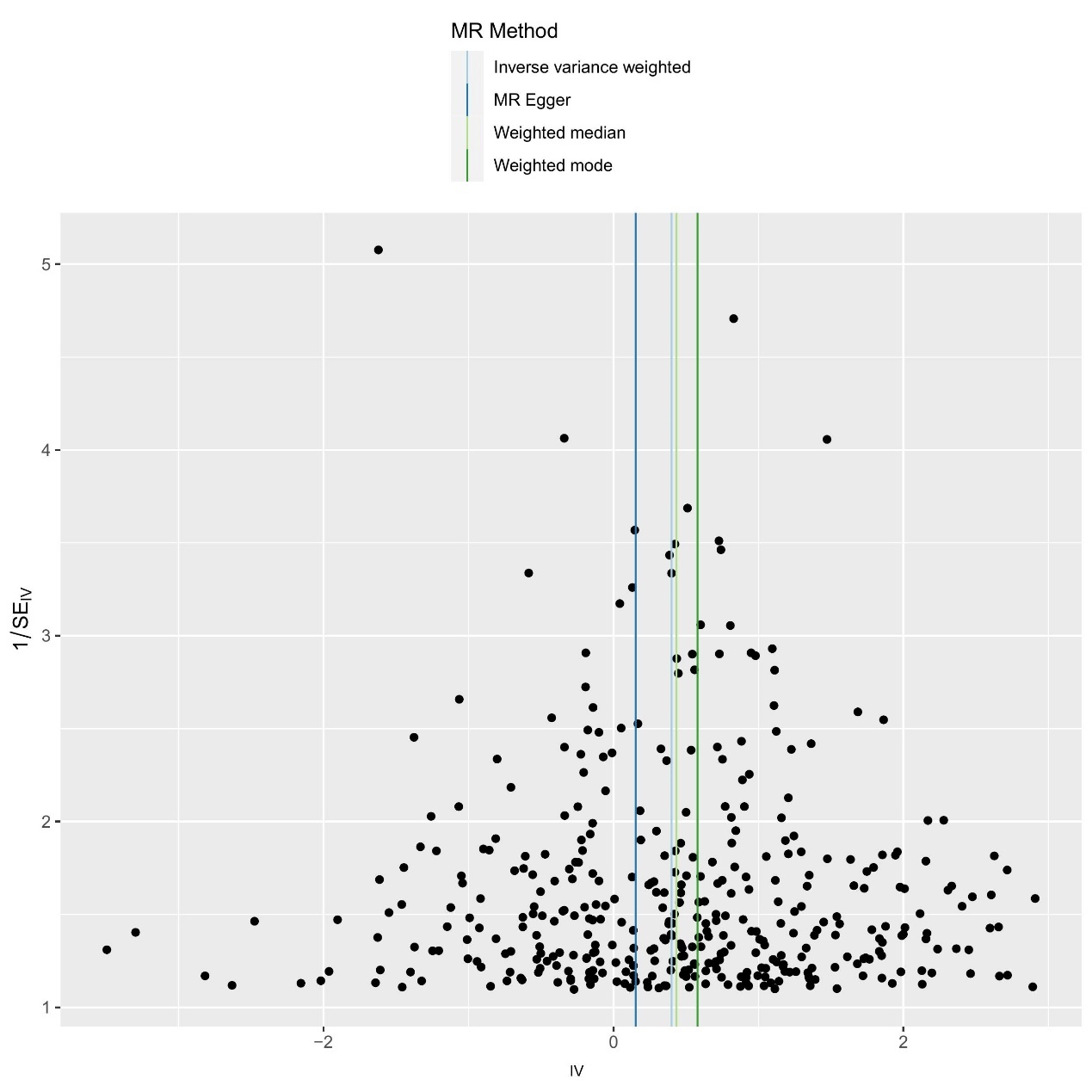


Supplementary Figure 76: Scatter plot of the causal effect of trunk fat-free mass on knee or hip OA.


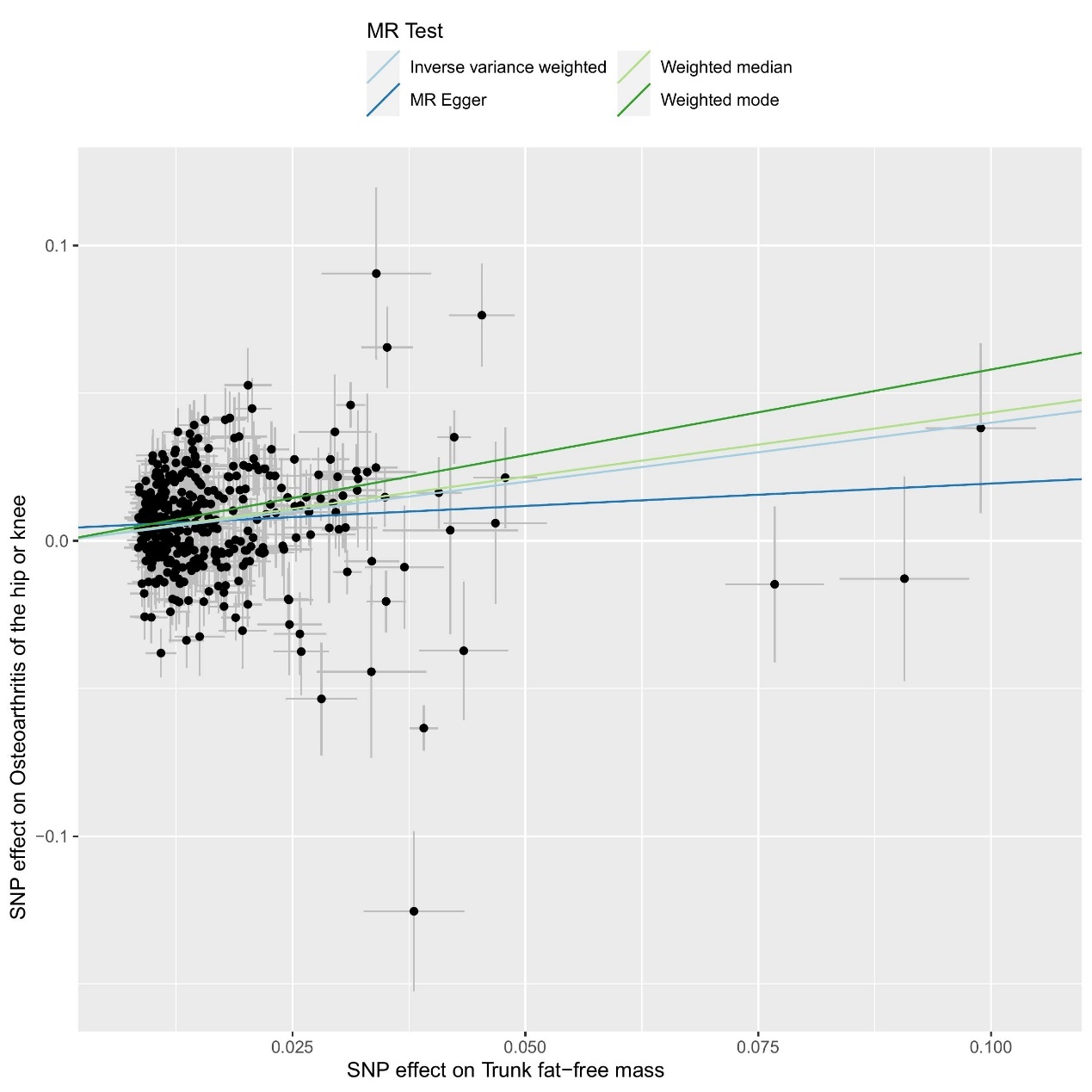


Supplementary Figure 77: Funnel plot of the causal effect of trunk fat-free mass on knee OA.


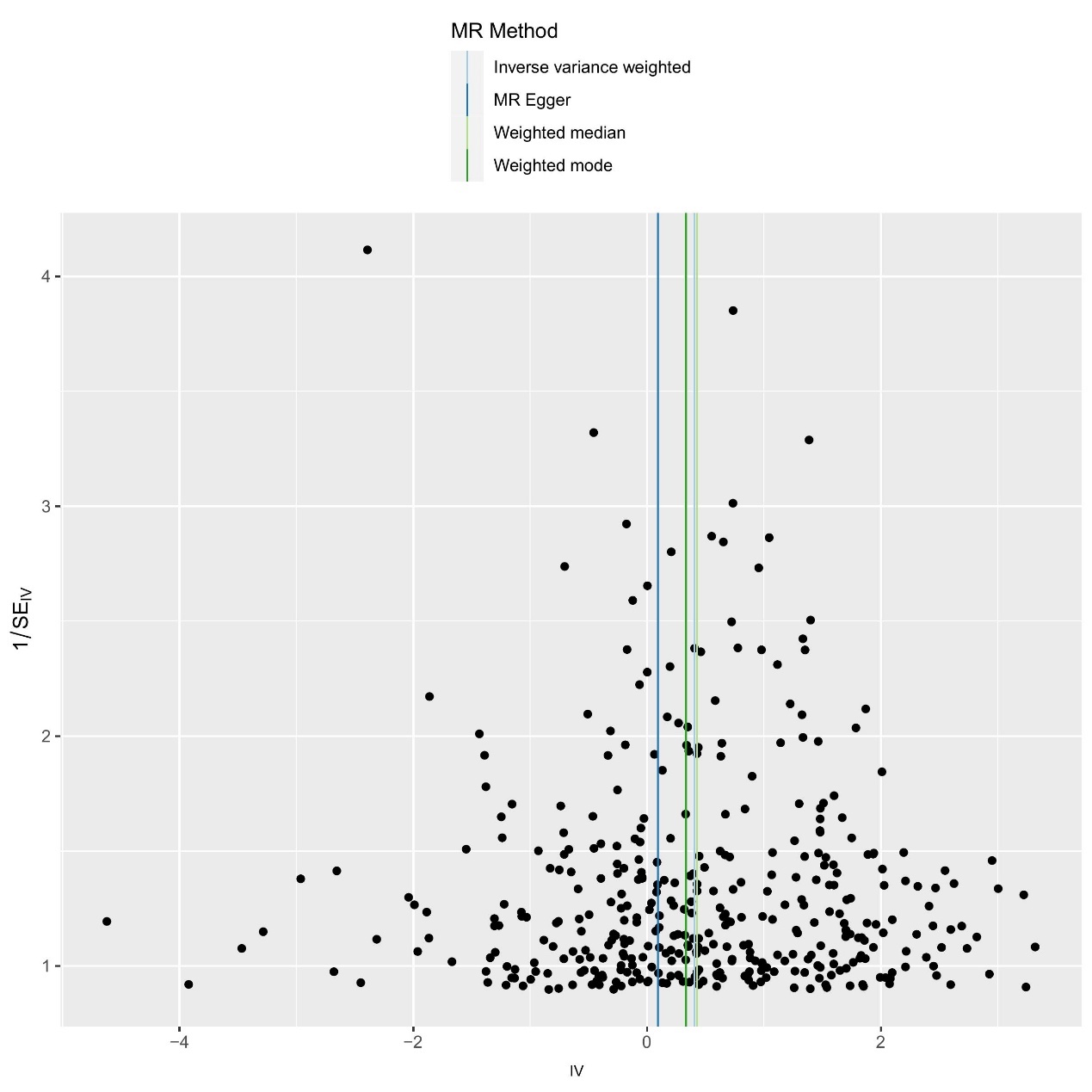


Supplementary Figure 78: Scatter plot of the causal effect of trunk fat-free mass on knee OA.


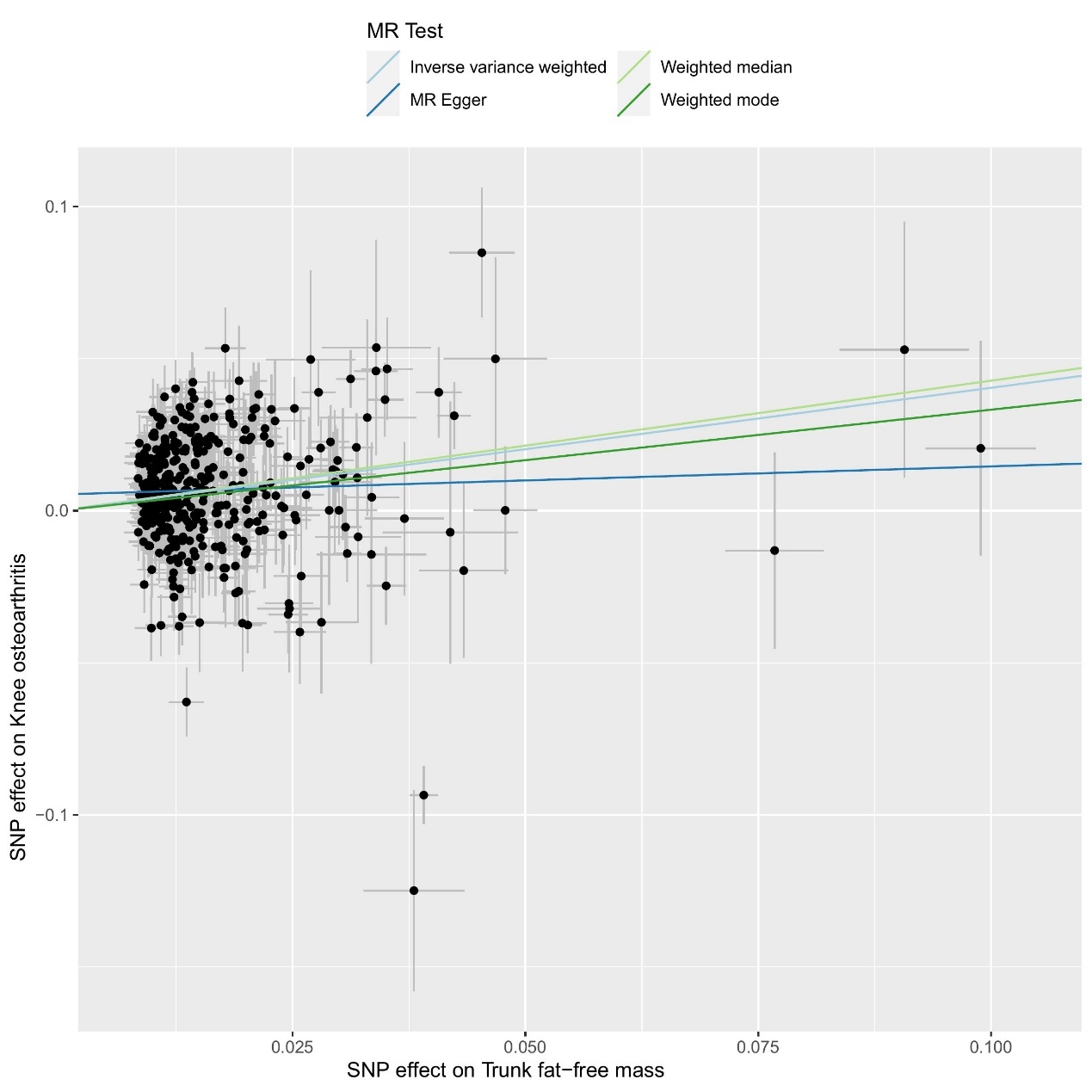


Supplementary Figure 79: Funnel plot of the causal effect of trunk fat mass on hip OA.


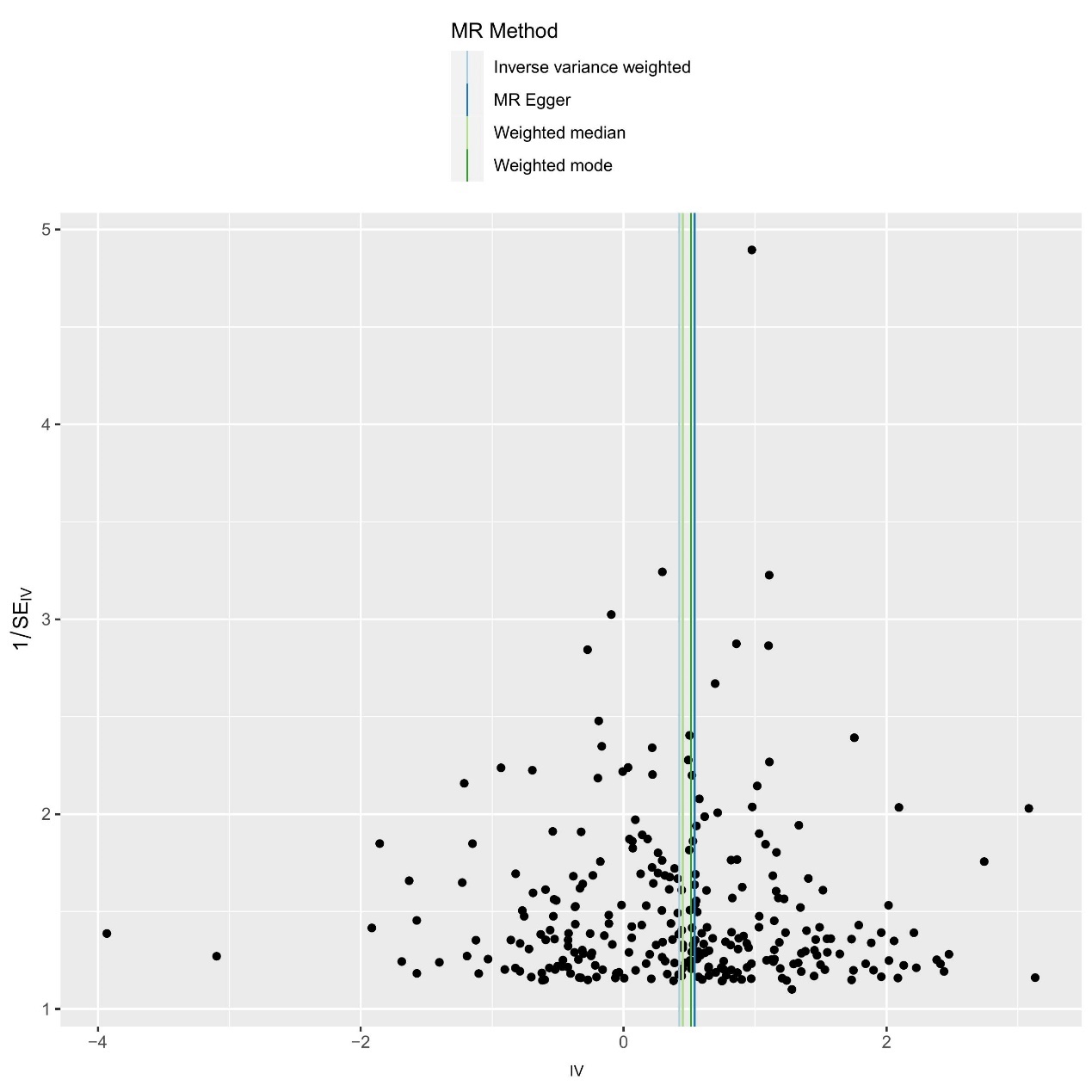


Supplementary Figure 80: Scatter plot of the causal effect of trunk fat mass on hip OA.


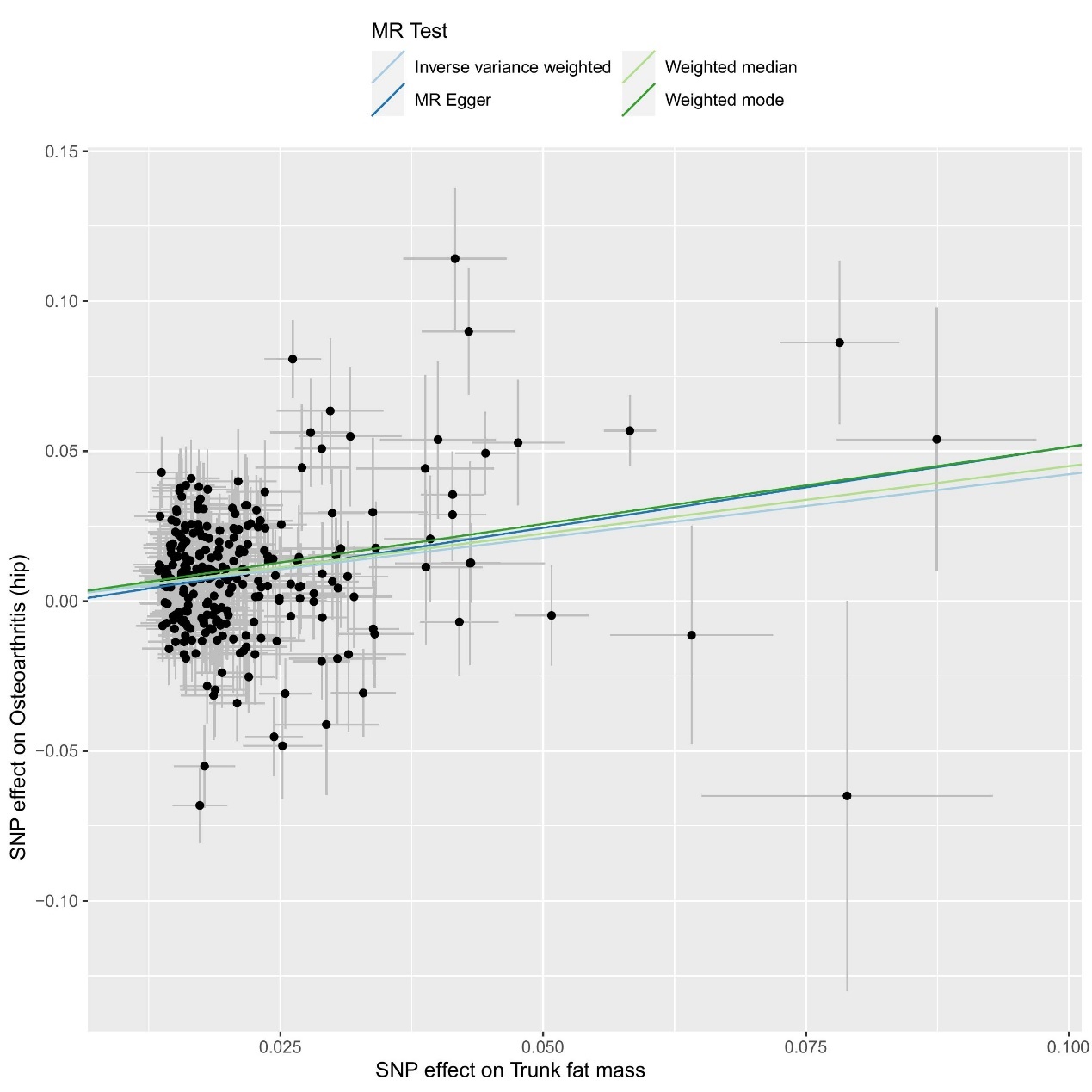


Supplementary Figure 81: Funnel plot of the causal effect of trunk fat mass on knee or hip OA.


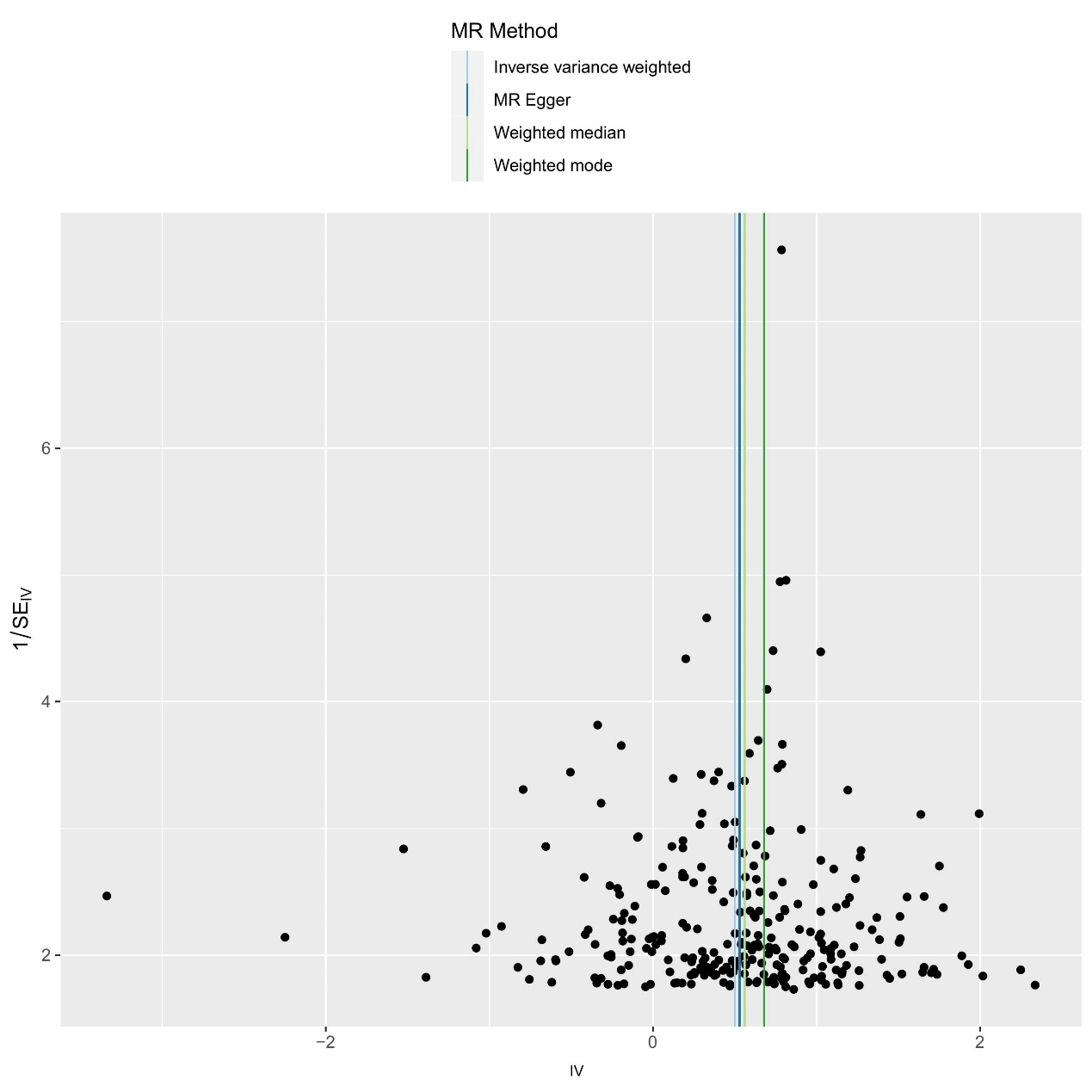


Supplementary Figure 82: Scatter plot of the causal effect of trunk fat mass on knee or hip OA.


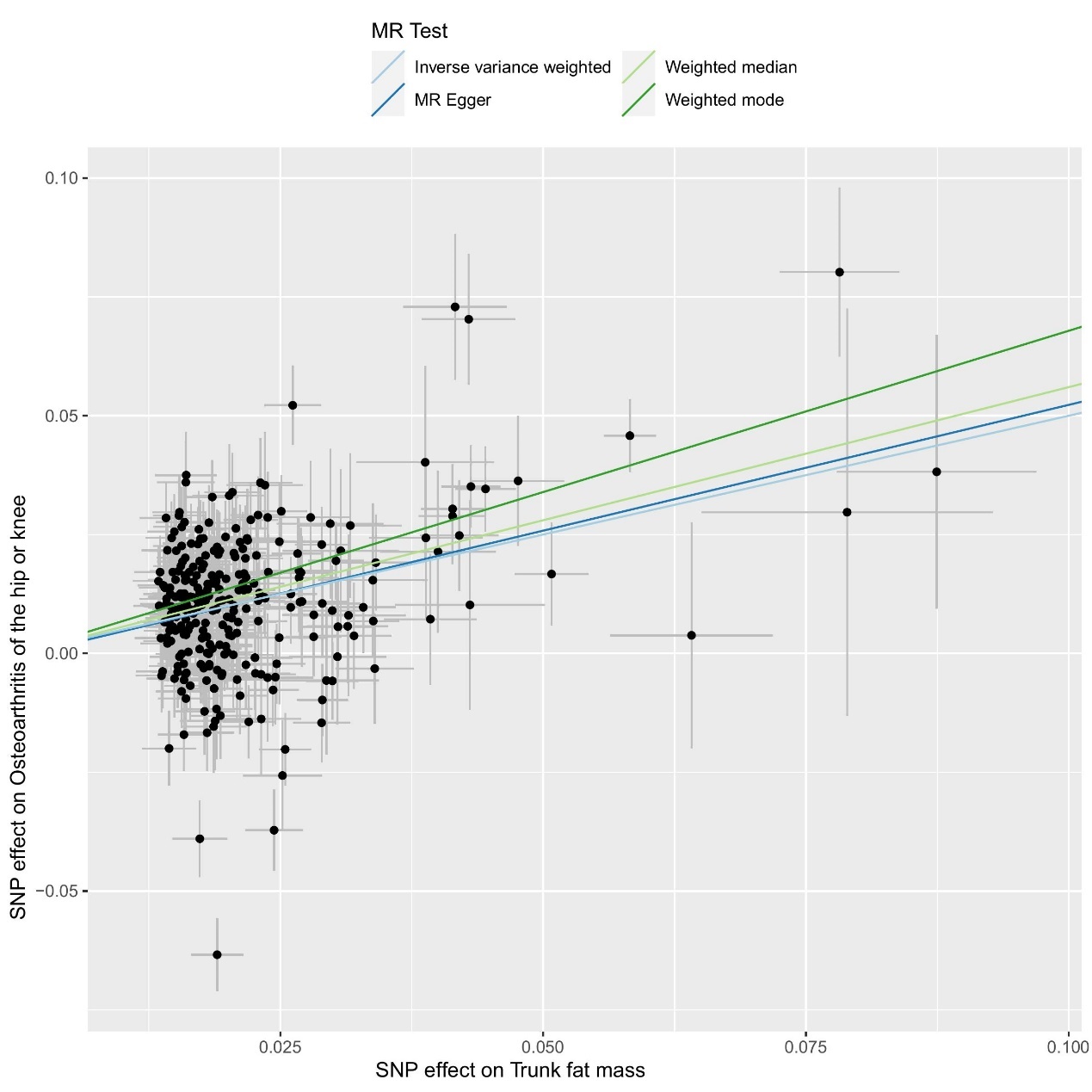


Supplementary Figure 83: Funnel plot of the causal effect of trunk fat mass on knee OA.


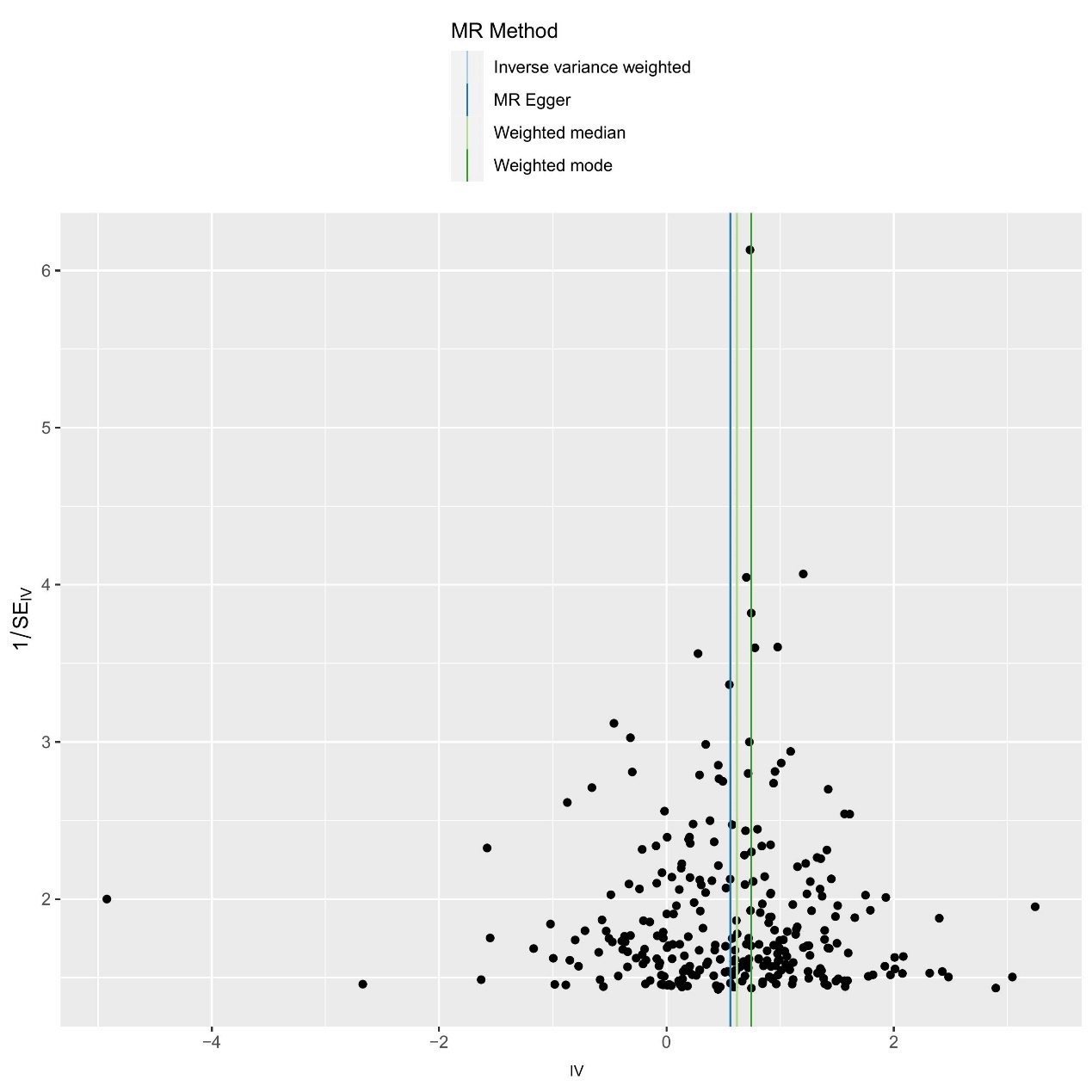


Supplementary Figure 84: Scatter plot of the causal effect of trunk fat mass on knee OA.


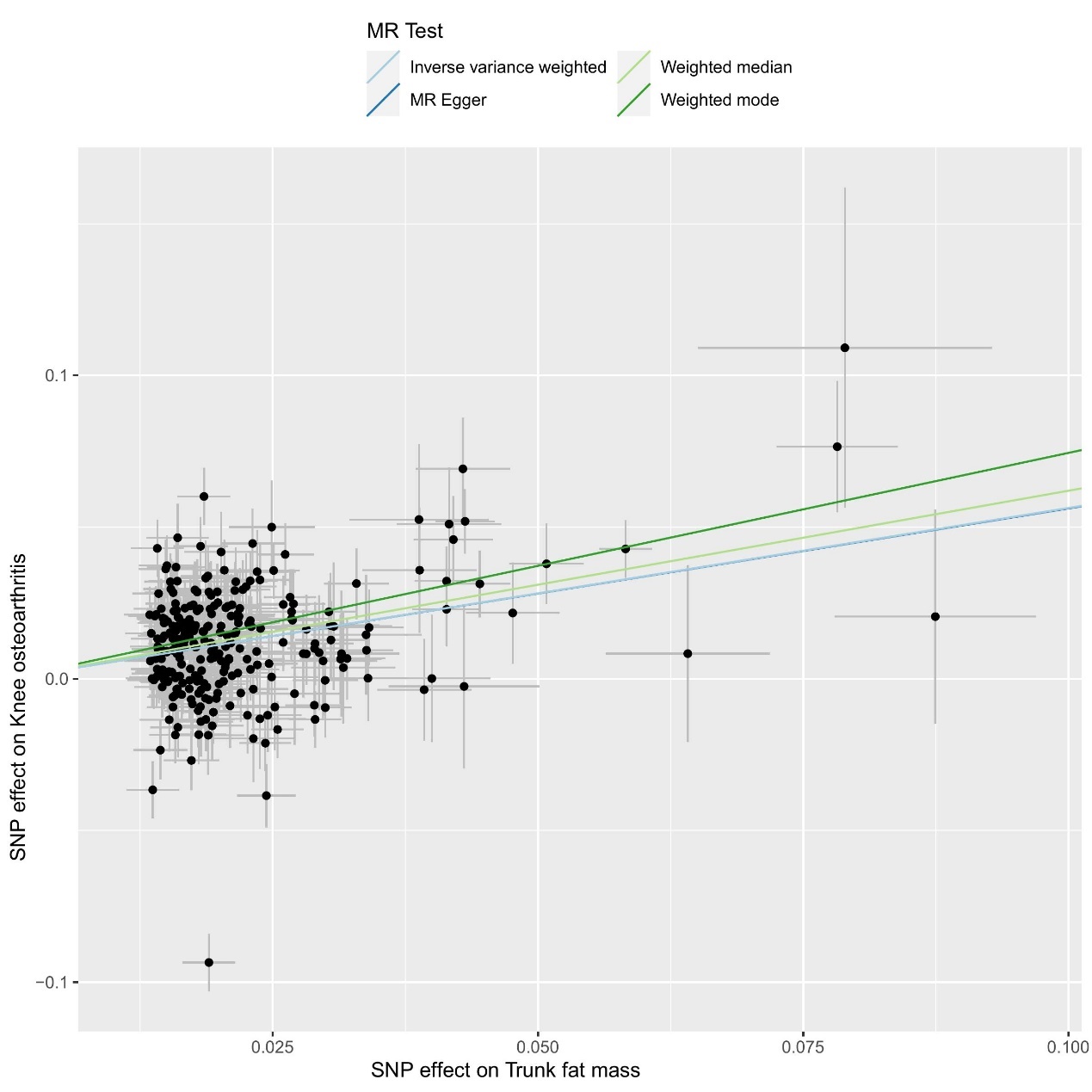


Supplementary Figure 85: Funnel plot of the causal effect of trunk fat percentage on hip OA.


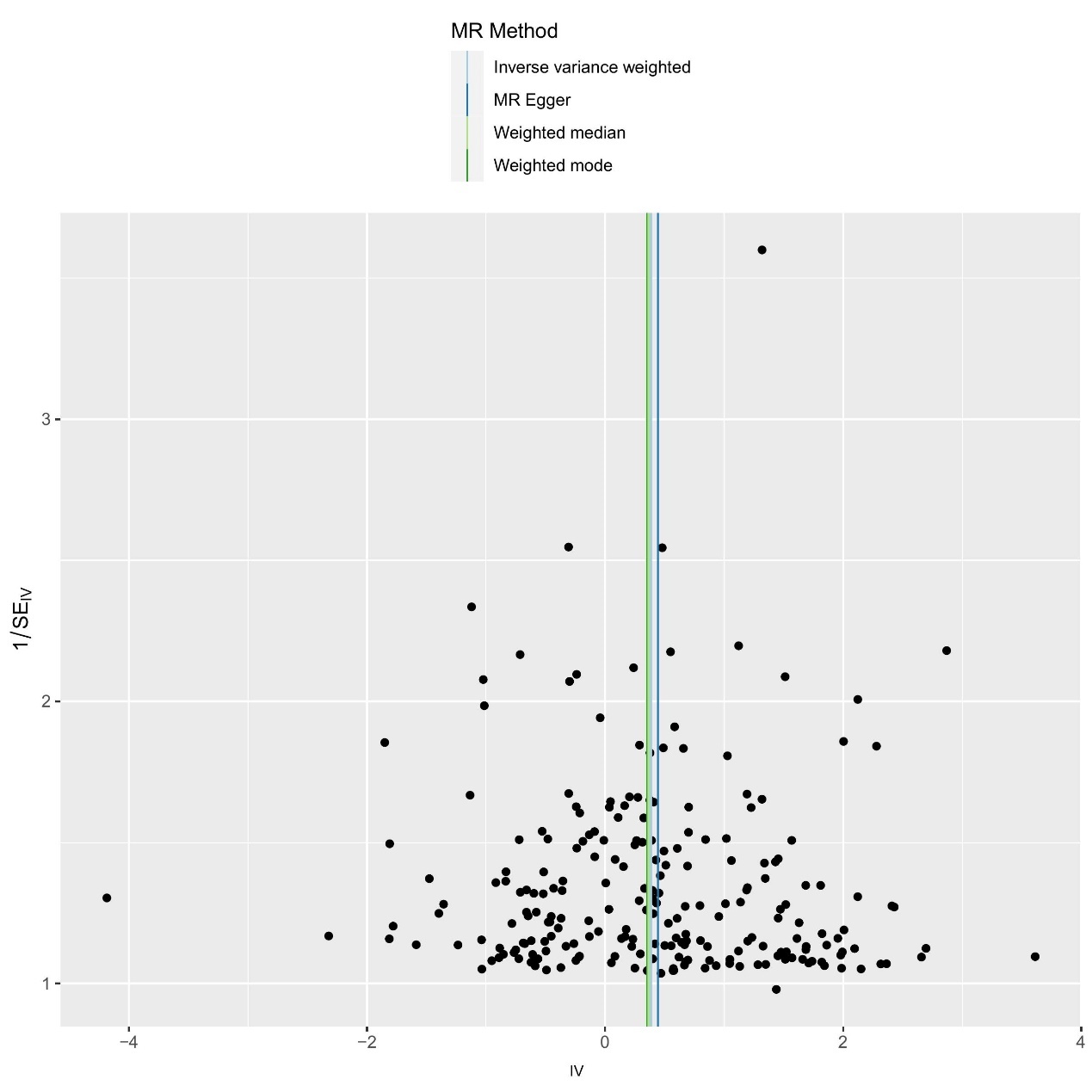


Supplementary Figure 86: Scatter plot of the causal effect of trunk fat percentage on hip OA.


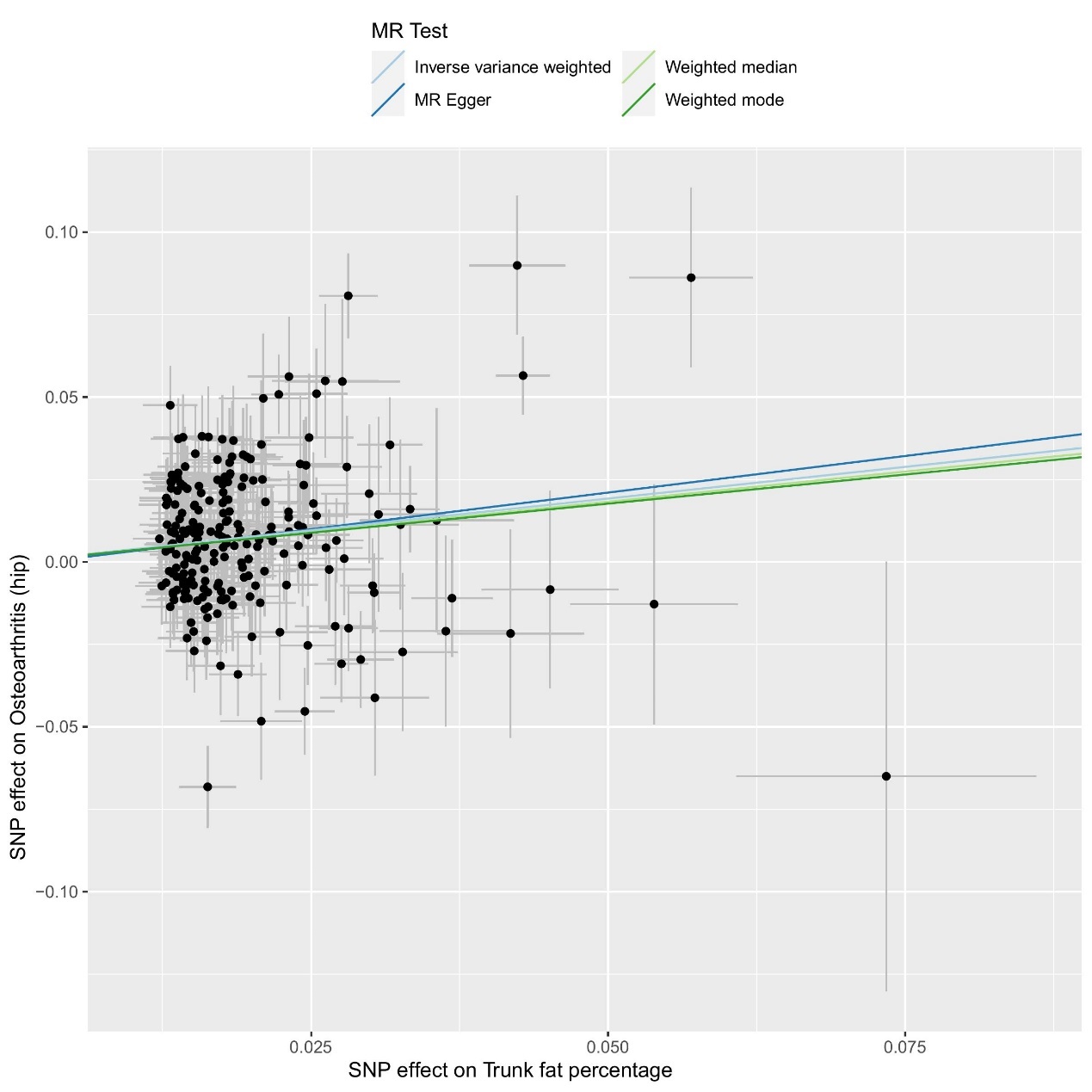


Supplementary Figure 87: Funnel plot of the causal effect of trunk fat percentage on knee or hip OA.


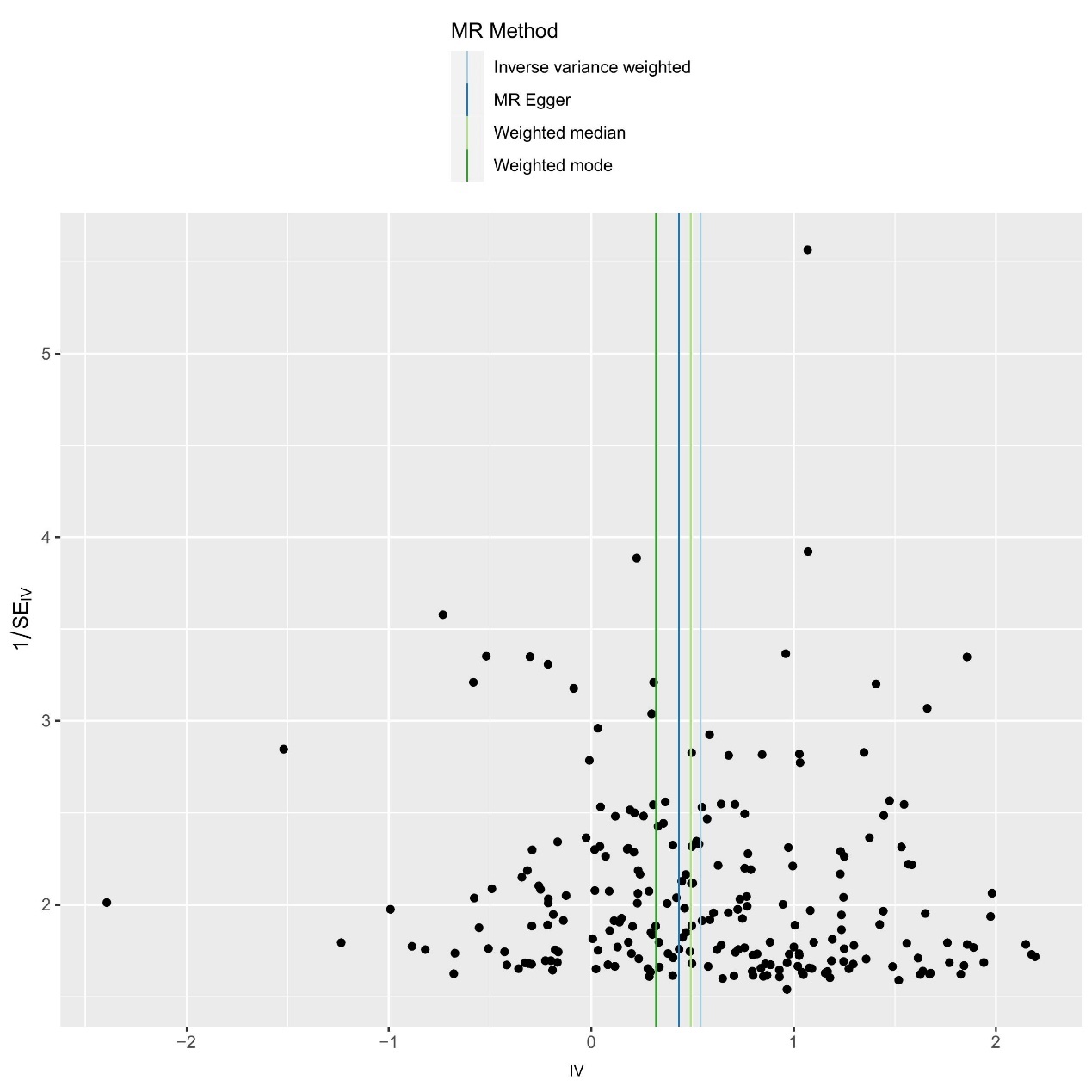


Supplementary Figure 88: Scatter plot of the causal effect of trunk fat percentage on knee or hip OA.


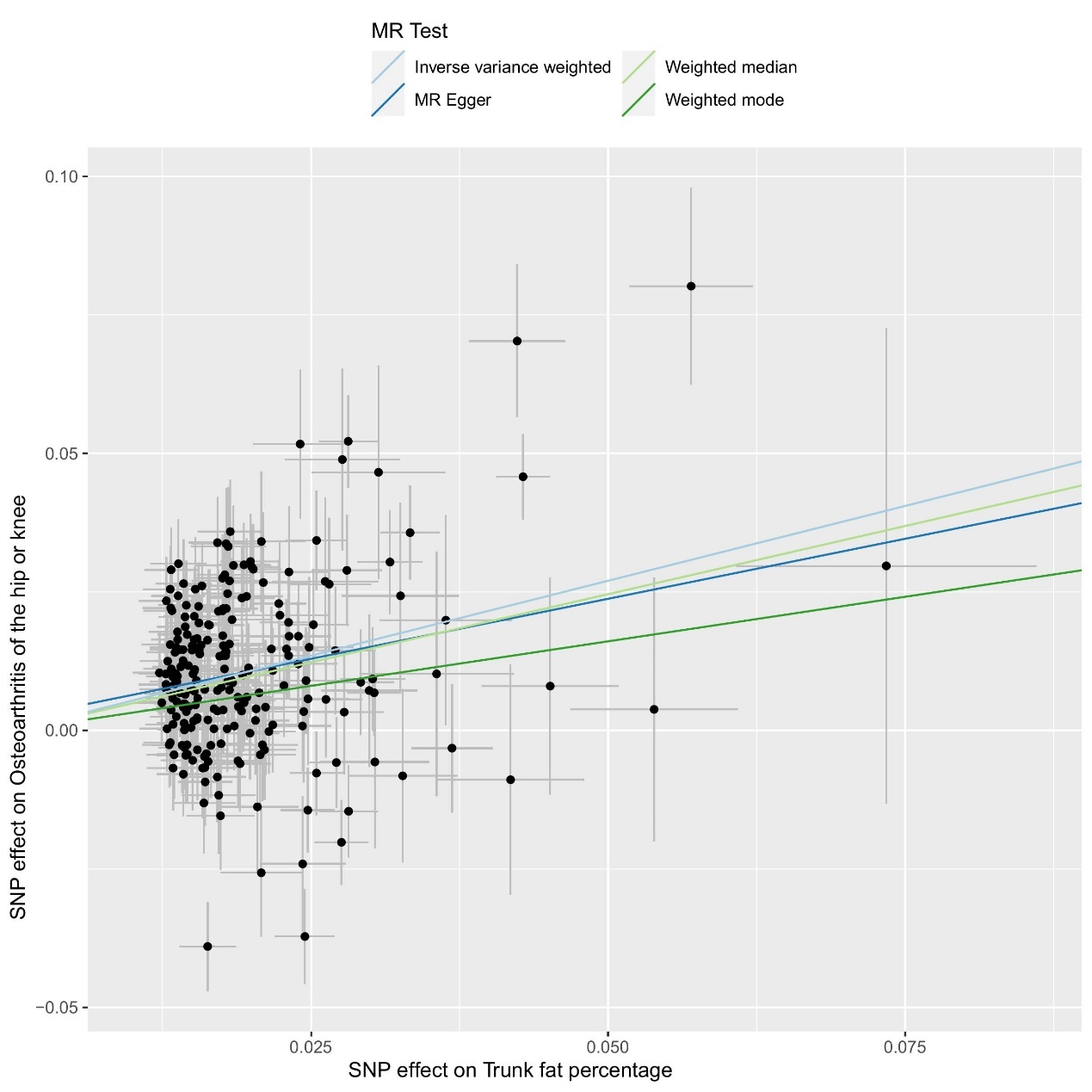


Supplementary Figure 89: Funnel plot of the causal effect of trunk fat percentage on knee OA.


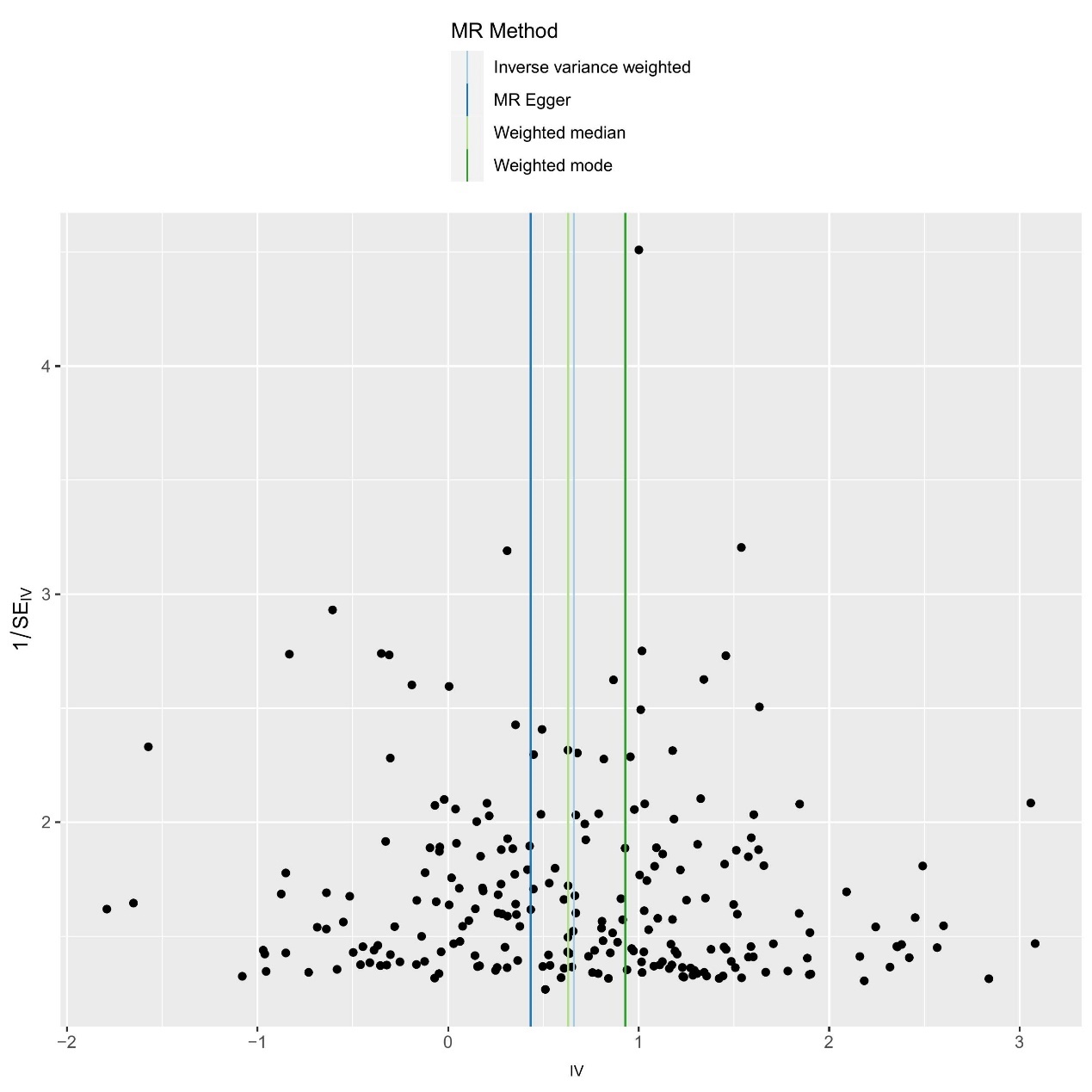


Supplementary Figure 90: Scatter plot of the causal effect of trunk fat percentage on knee OA.


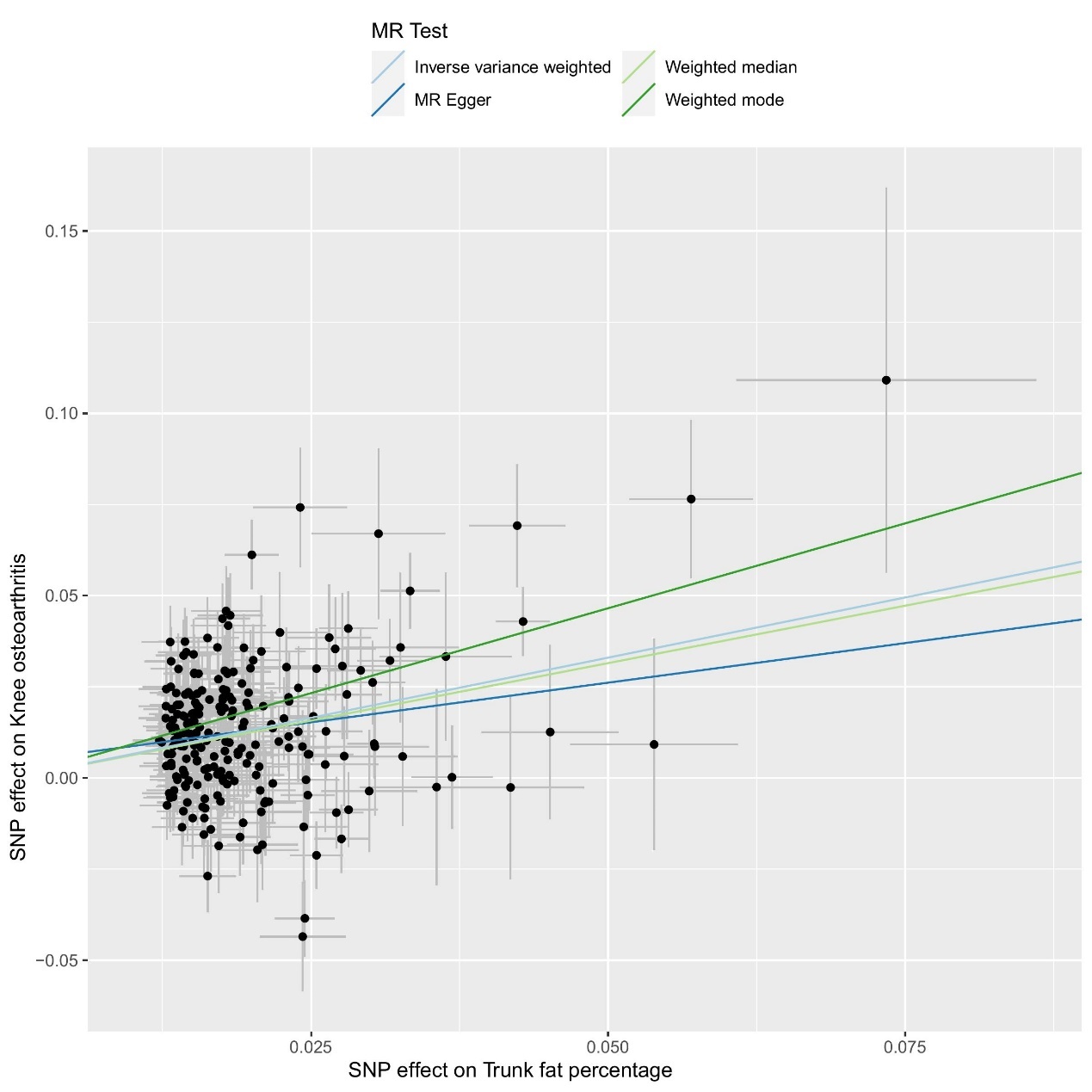


Supplementary Figure 91: Funnel plot of the causal effect of waist circumference on hip OA.


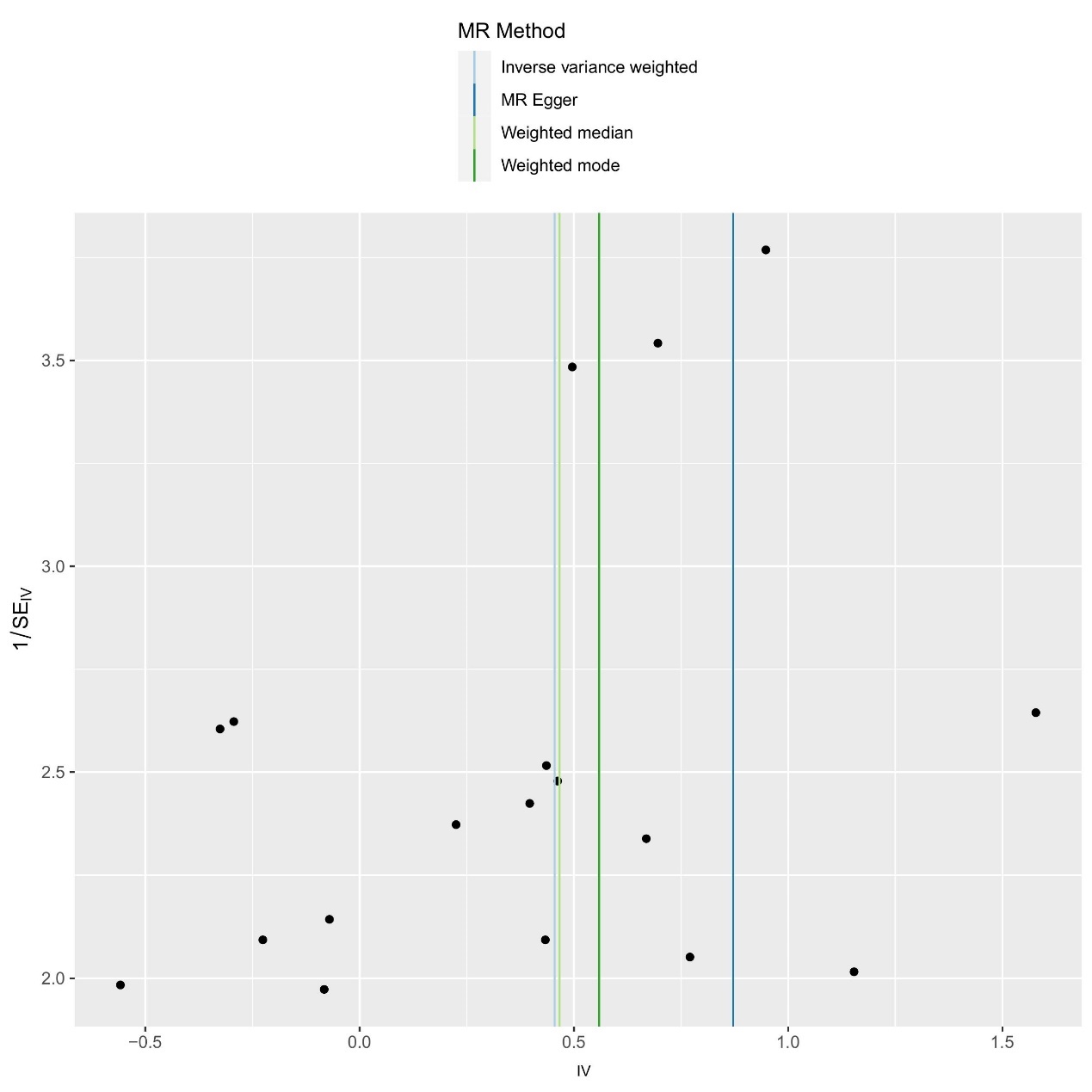


Supplementary Figure 92: Scatter plot of the causal effect of waist circumference on hip OA.


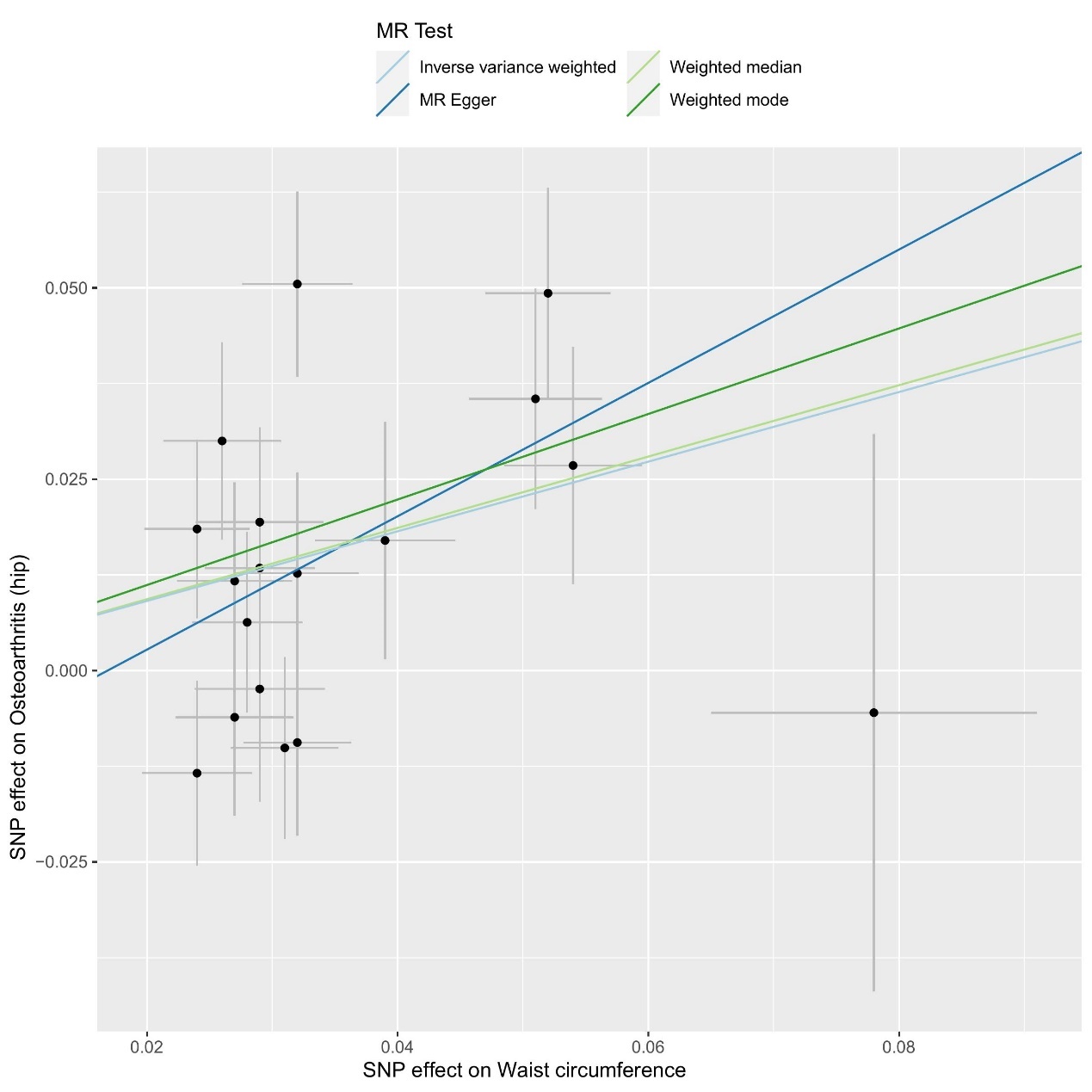


Supplementary Figure 93: Funnel plot of the causal effect of waist circumference on knee or hip OA.


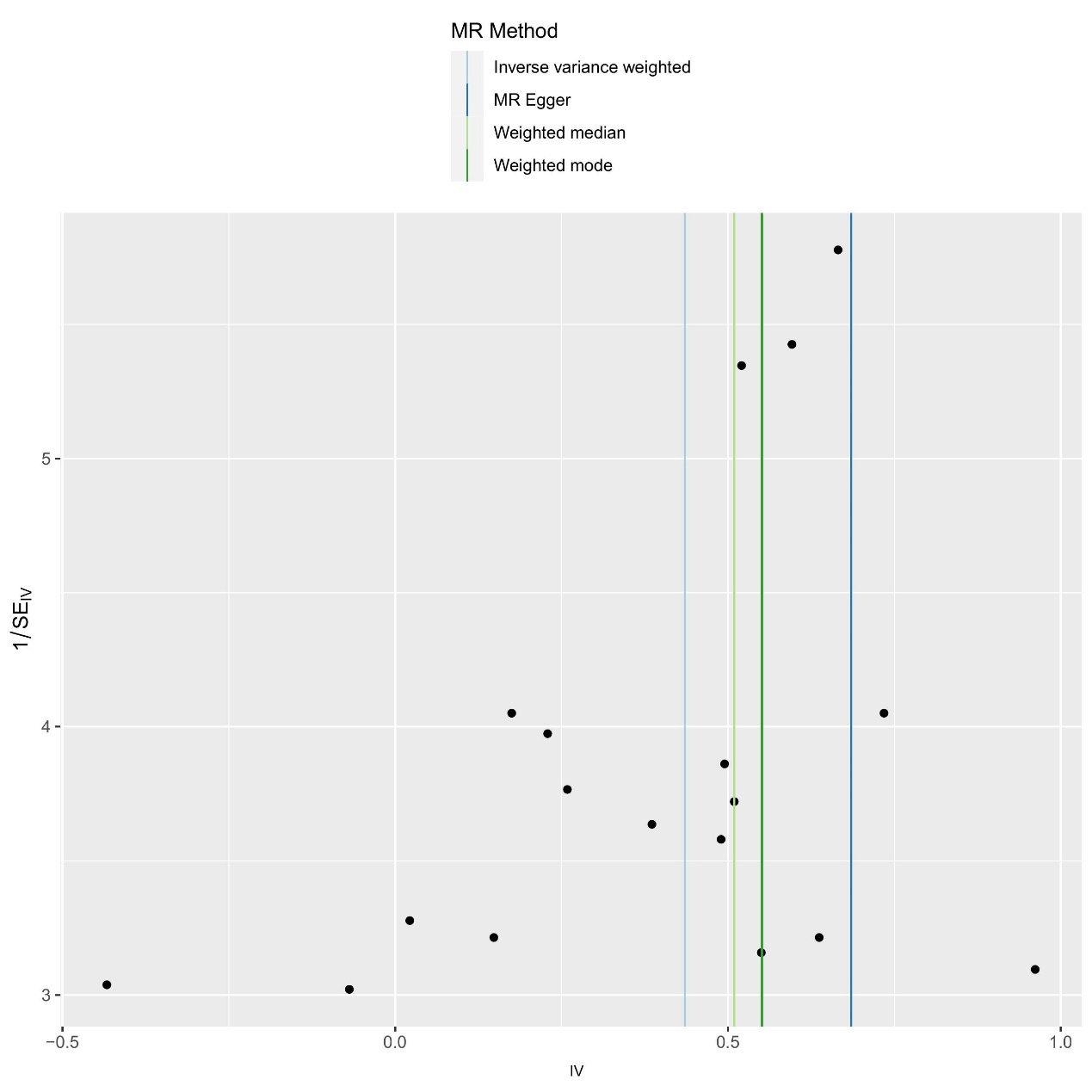


Supplementary Figure 94: Scatter plot of the causal effect of waist circumference on knee or hip OA.


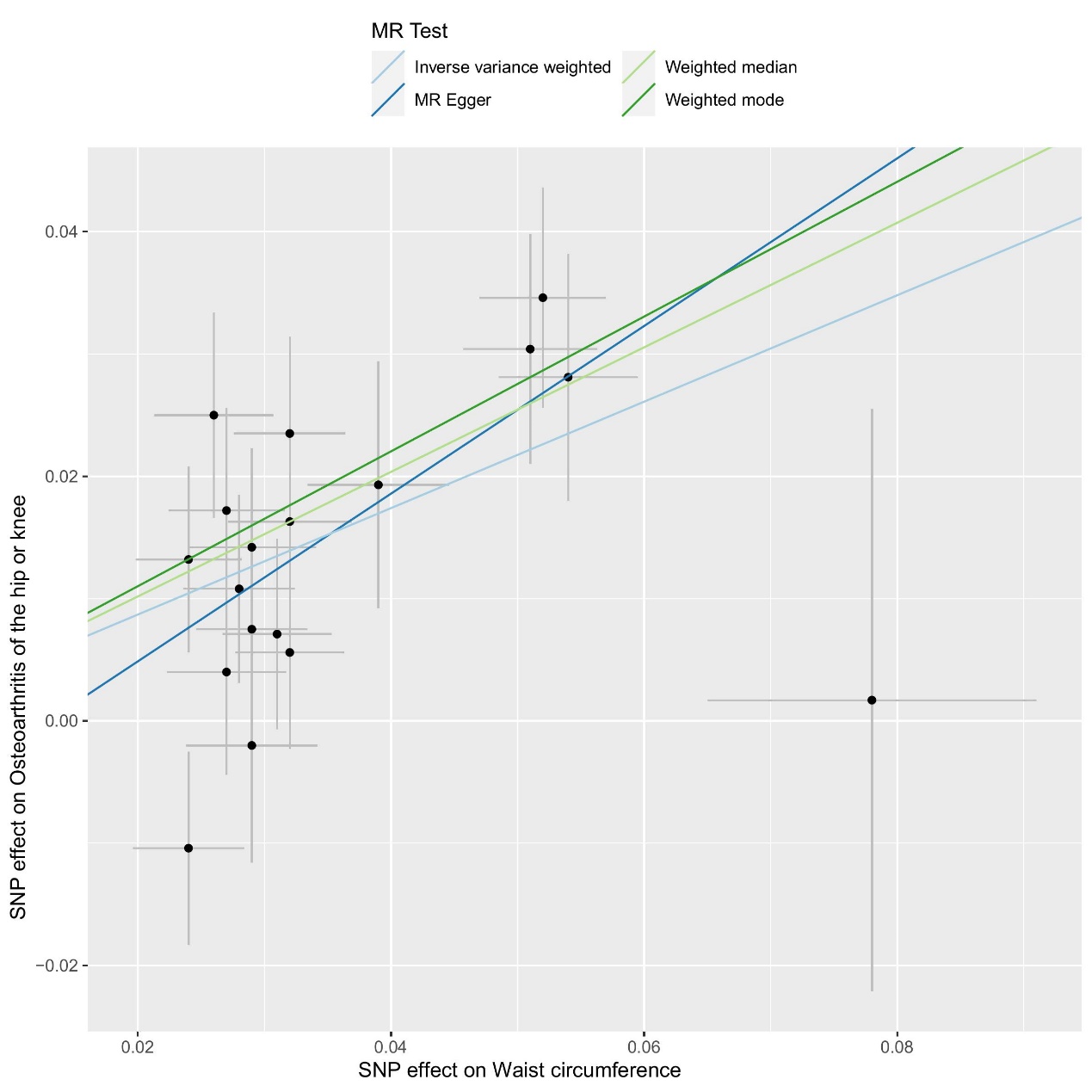


Supplementary Figure 95: Funnel plot of the causal effect of waist circumference on knee OA.


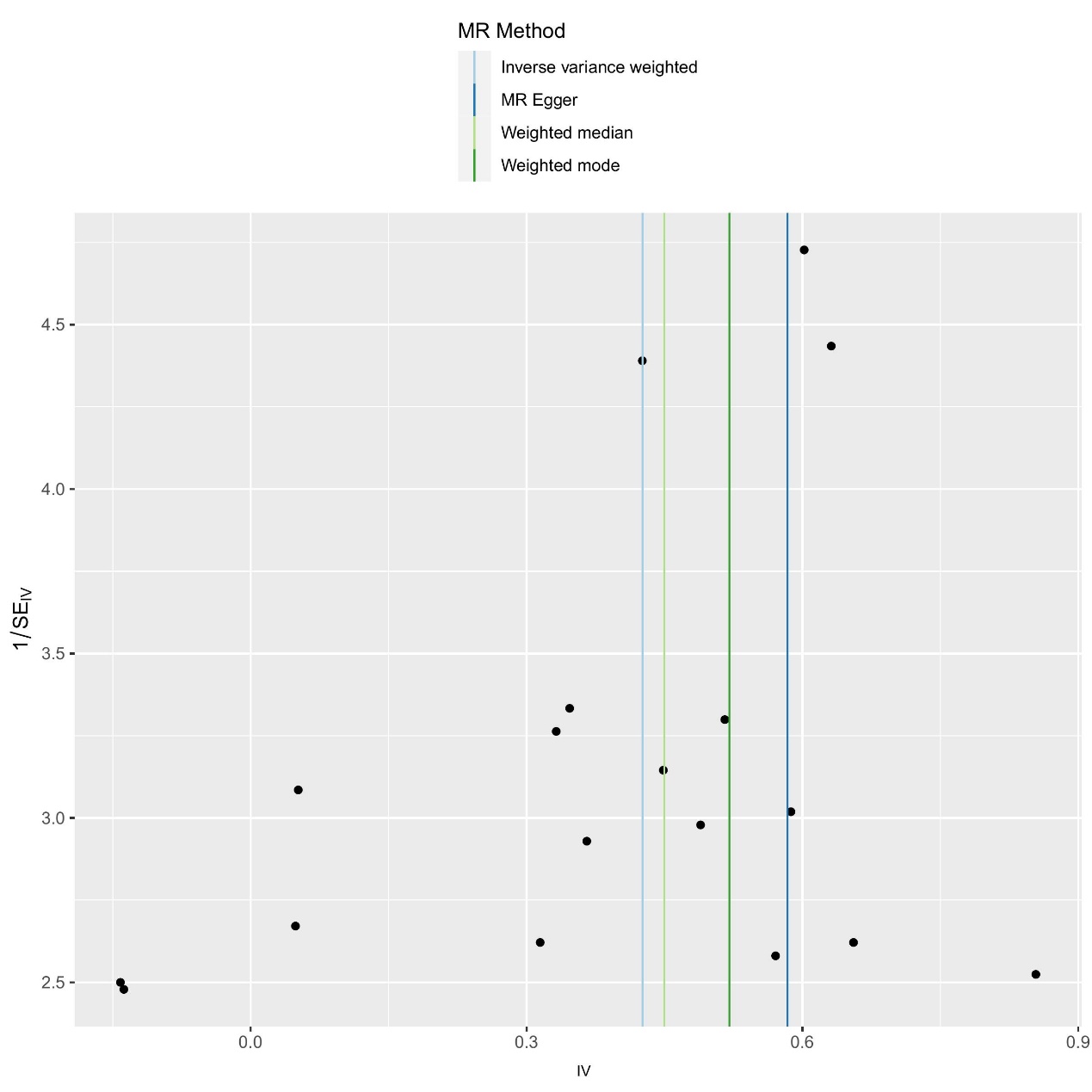


Supplementary Figure 96: Scatter plot of the causal effect of waist circumference on knee OA.


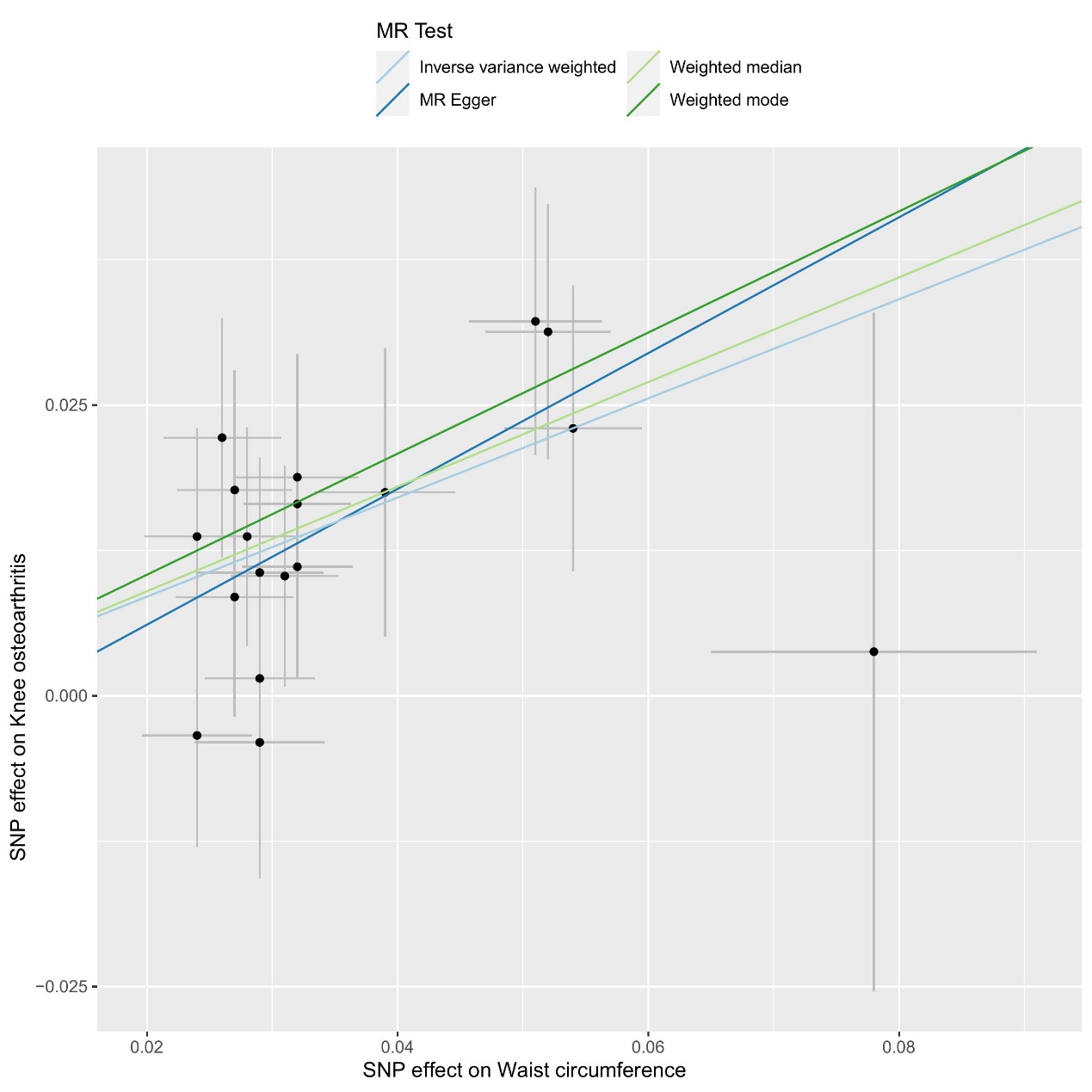


Supplementary Figure 97: Funnel plot of the causal effect of waist-to-hip ratio on hip OA.


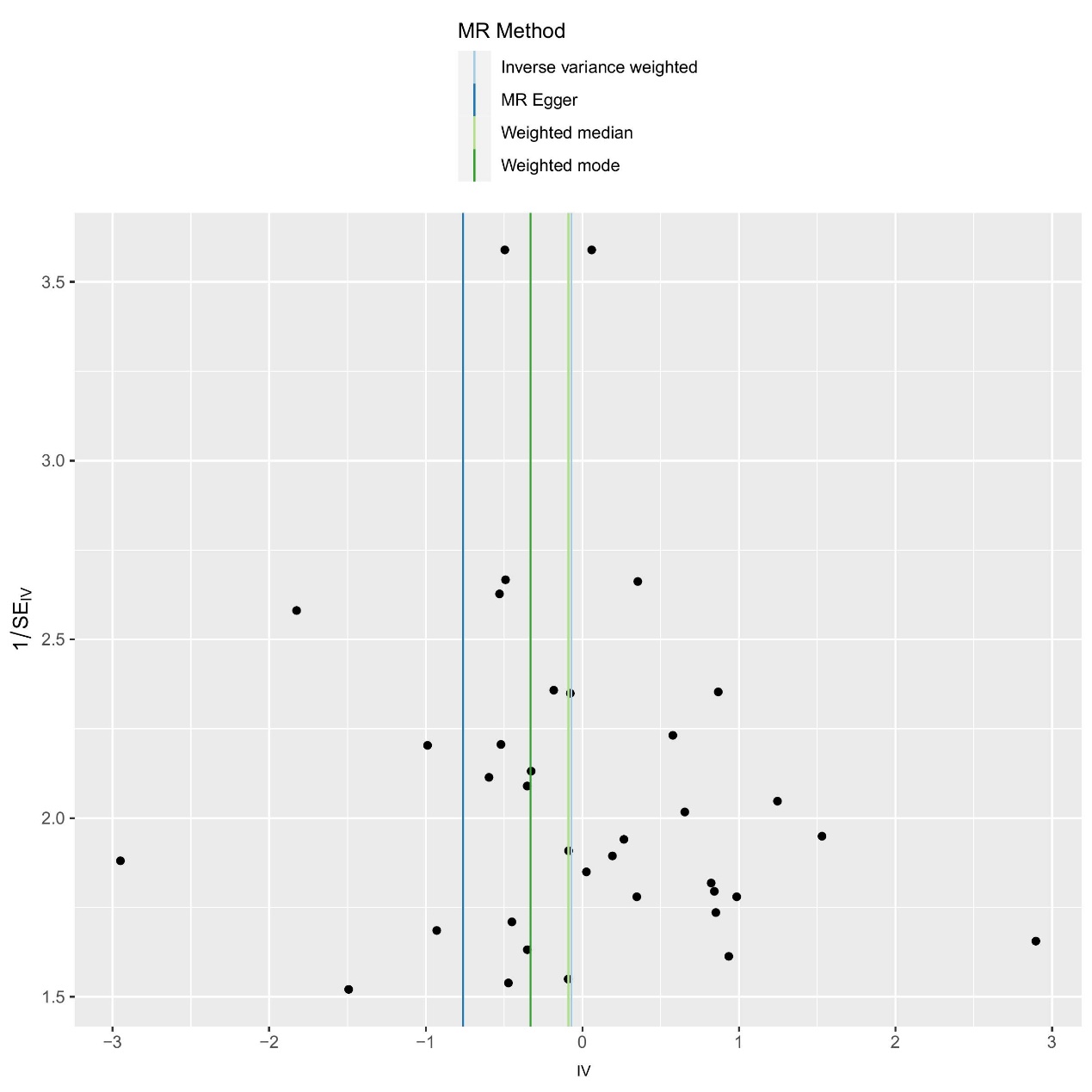


Supplementary Figure 98: Scatter plot of the causal effect of waist-to-hip ratio on hip OA.


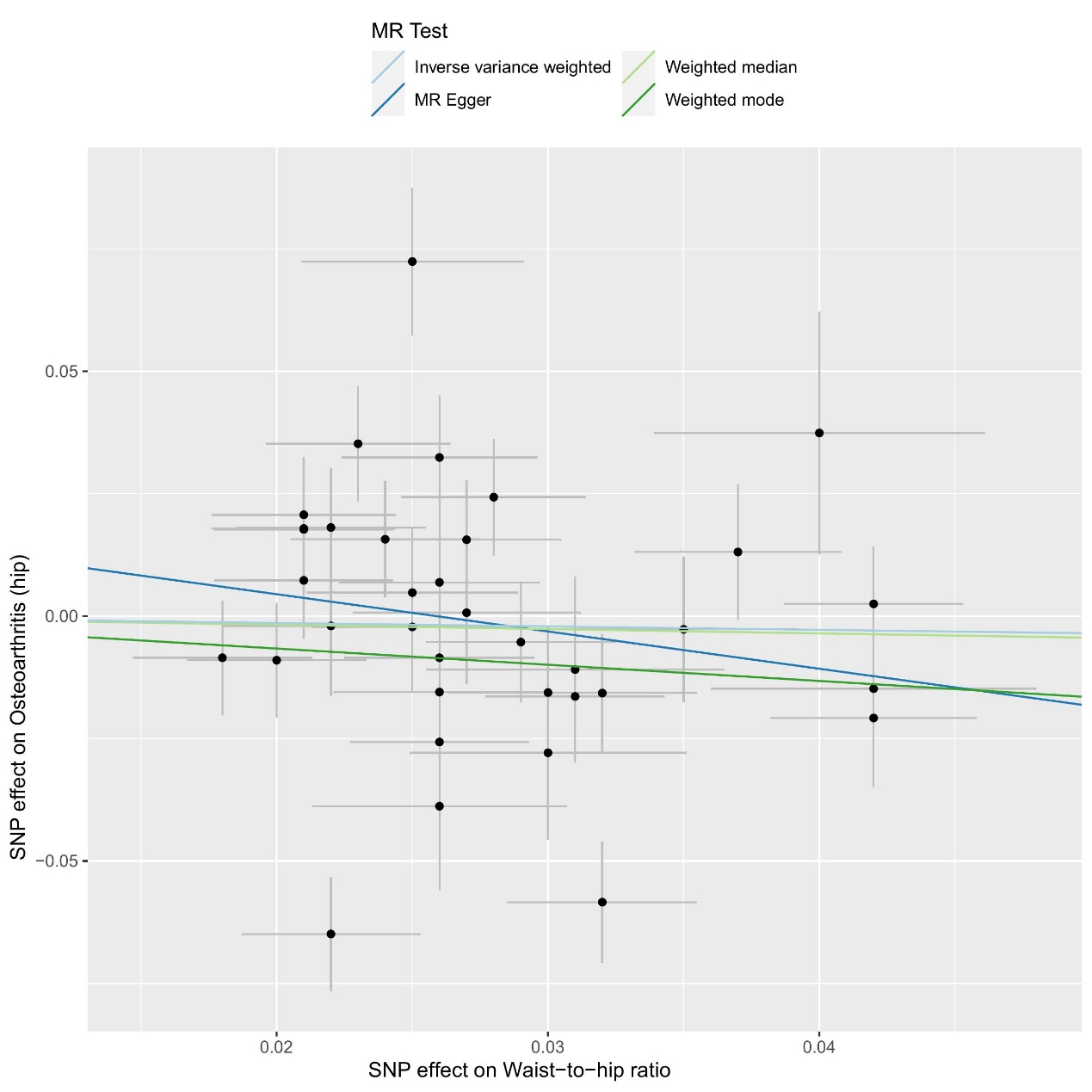


Supplementary Figure 99: Funnel plot of the causal effect of waist-to-hip ratio on knee or hip OA.


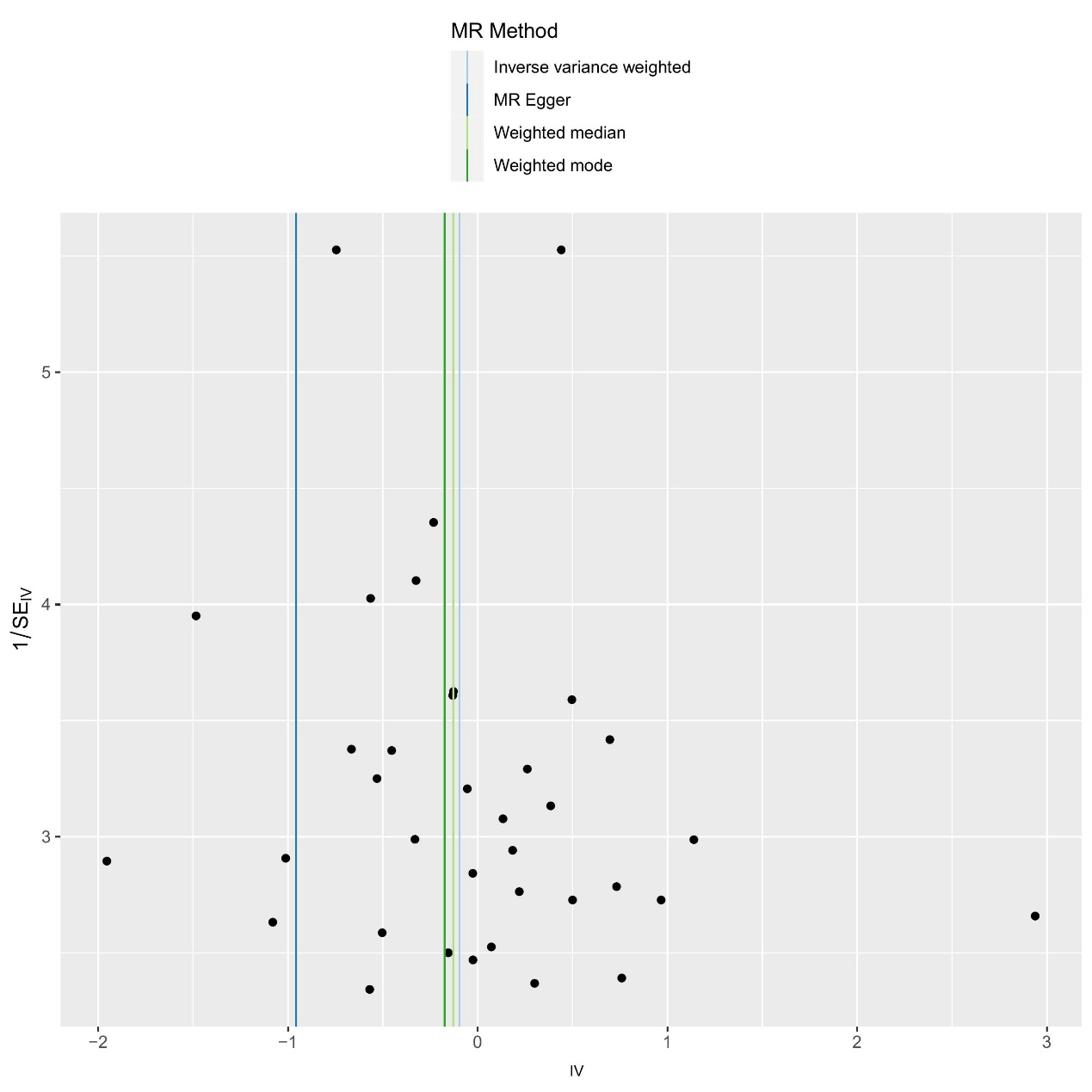


Supplementary Figure 100: Scatter plot of the causal effect of waist-to-hip ratio on knee or hip OA.


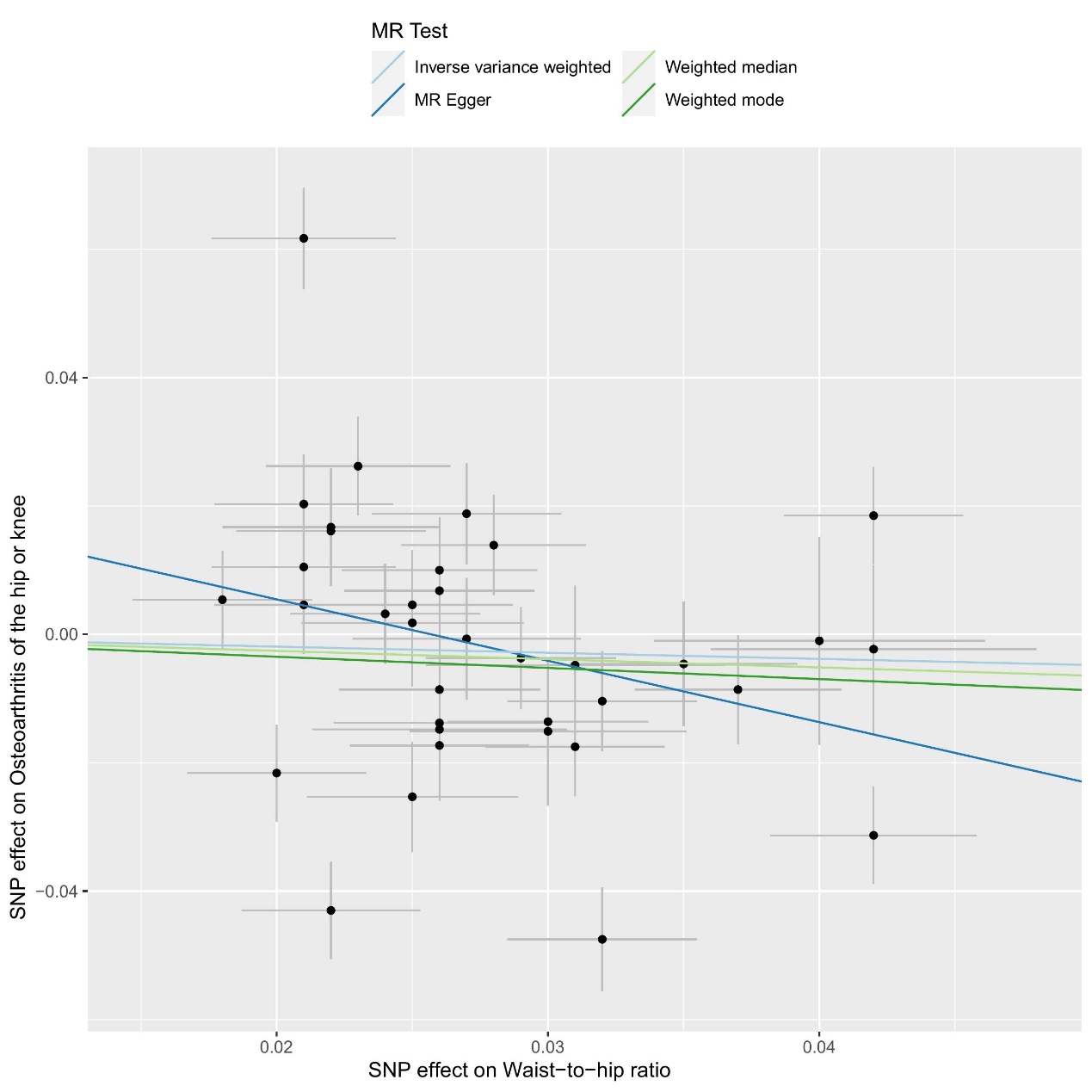


Supplementary Figure 101: Funnel plot of the causal effect of waist-to-hip ratio on knee OA.

Supplementary Figure 102: Scatter plot of the causal effect of waist-to-hip ratio on knee OA.

Supplementary Figure 103: Funnel plot of the causal effect of weight on hip OA.

Supplementary Figure 104: Scatter plot of the causal effect of weight on hip OA.

Supplementary Figure 105: Funnel plot of the causal effect of weight on knee or hip OA.

Supplementary Figure 106: Scatter plot of the causal effect of weight on knee or hip OA.

Supplementary Figure 107: Funnel plot of the causal effect of weight on knee OA.

Supplementary Figure 108: Scatter plot of the causal effect of weight on knee OA.

Supplementary Figure 109: Funnel plot of the causal effect of whole body fat-free mass on hip OA.

Supplementary Figure 110: Scatter plot of the causal effect of whole body fat-free mass on hip OA.

Supplementary Figure 111: Funnel plot of the causal effect of whole body fat-free mass on knee or hip OA.

Supplementary Figure 112: Scatter plot of the causal effect of whole body fat-free mass on knee or hip OA.

Supplementary Figure 113: Funnel plot of the causal effect of whole body fat-free mass on knee OA.

Supplementary Figure 114: Scatter plot of the causal effect of whole body fat-free mass on knee OA.

Supplementary Figure 115: Funnel plot of the causal effect of whole body fat mass on hip OA.

Supplementary Figure 116: Scatter plot of the causal effect of whole body fat mass on hip OA.

Supplementary Figure 117: Funnel plot of the causal effect of whole body fat mass on knee or hip OA.

Supplementary Figure 118: Scatter plot of the causal effect of whole body fat mass on knee or hip OA.

Supplementary Figure 119: Funnel plot of the causal effect of whole body fat mass on knee OA.

Supplementary Figure 120: Scatter plot of the causal effect of whole body fat mass on knee OA.
